# Genome-Wide Analysis of the Cytochrome P450 Monooxygenases in the Lichenized Fungi of the Class *Lecanoromycetes*

**DOI:** 10.3390/microorganisms11102590

**Published:** 2023-10-19

**Authors:** Gugulethu Mlambo, Tiara Padayachee, David R. Nelson, Khajamohiddin Syed

**Affiliations:** 1 Department of Biochemistry and Microbiology, Faculty of Science, Agriculture and Engineering, University of Zululand, Vulindlela, KwaDlangezwa 3886, South Africa; gugulethumlambo75@gmail.com (G.M.); teez07padayachee@gmail.com (T.P.); 2 Department of Microbiology, Immunology and Biochemistry, University of Tennessee Health Science Center, Memphis, TN 38163, USA

**Keywords:** lichens, CYPs, P450s, genome-wide data-mining, phylogenetic analysis, *Pezizomycetes*, *Lecanoromycetes*, depside, depsidone, biosynthetic gene clusters

## Abstract

Lichens are unique organisms that exhibit a permanent symbiosis between fungi and algae or fungi and photosynthetic bacteria. Lichens have been found to produce biotechnologically valuable secondary metabolites. A handful of studies showed that tailoring enzymes such as cytochrome P450 monooxygenases (CYPs/P450s) play a key role in synthesizing these metabolites. Despite the critical role of P450s in the biosynthesis of secondary metabolites, the systematic analysis of P450s in lichens has yet to be reported. This study is aimed to address this research gap. A genome-wide analysis of P450s in five lichens from the fungal class *Lecanoromycetes* revealed the presence of 434 P450s that are grouped into 178 P450 families and 345 P450 subfamilies. The study indicated that none of the P450 families bloomed, and 15 P450 families were conserved in all five *Lecanoromycetes*. *Lecanoromycetes* have more P450s and higher P450 family diversity compared to *Pezizomycetes*. A total of 73 P450s were found to be part of secondary metabolite gene clusters, indicating their potential involvement in the biosynthesis of secondary metabolites. Annotation of P450s revealed that CYP682BG1 and CYP682BG2 from *Cladonia grayi* and *Pseudevernia furfuracea* (physodic acid chemotype) are involved in the synthesis of grayanic acid and physodic acid, CYP65FQ2 from *Stereocaulon alpinum* is involved in the synthesis of atranorin, and CYP6309A2 from *Cladonia uncialis* is involved in the synthesis of usnic acid. This study serves as a reference for future annotation of P450s in lichens.

## 1. Introduction

Lichens are the only organisms on Earth that consist of fungi (the mycobiont) and photosynthetic cyanobacteria or algae (the photobiont) in a permanent symbiotic relationship. The symbiotic relationships between fungi and algae have been found to exist for at least 400 million years [1]. The fungal partner is considered when determining the identity of lichens, and as of now, ascomycetes have been identified as the fungal partner in the majority of lichen species [2,3]. Thus, “lichen-forming fungi (LFF)” refer to fungi that establish a symbiotic relationship and live in a lichen thallus during the entire life cycle.

Lichens can be found in almost all places on Earth, including the harshest environments, such as extreme cold and hot, covering over 10% of the terrestrial surface [4,5,6,7,8]. Lichens’ broad habitats and long lifespans demand a robust defense against harmful environmental elements and predatory species. One line of defense is the biosynthesis of secondary metabolites, chemicals produced by an organism that are not required for its growth, development, and reproduction but have biological activity that can indirectly equip the organism to defend better and survive. It is well known that lichens produce a wide range of secondary metabolites, and harnessing their potential for human benefit has received much attention [9,10,11].

Attempts are often made to find the genes involved in secondary metabolite synthesis [12]. Among the genes identified as key players in the synthesis of metabolites, tailoring enzymes such as cytochrome P450 monooxygenases (CYPs/P450s) were found to be critical in not only synthesis but also in conferring diversity to the metabolites [12]. P450s are heme-thiolate-containing proteins present in almost all organisms, including non-living entities such as viruses [13,14]. P450s are well known in biology due to their stereo- and regio-specific oxidation of diverse compounds, including their synthesis and contributing diversity to the secondary metabolites in organisms [15,16].

For P450s, a distinct nomenclature and classification scheme has been devised [17,18]. The prefix “CYP” stands for cytochrome P450 monooxygenase, and it is followed by an Arabic numeral indicating the family, a capital letter indicating the subfamily, and an Arabic digit representing the individual P450 in a family. According to the annotation/classification criterion, all P450s with >40% identity belong to the same family, and all P450s with >55% identity belong to the same subfamily.

P450s in fungi are involved in primary and secondary metabolism, where, due to their key enzymatic reactions, they serve frequently as a drug target [19,20,21]. Concerning secondary metabolism, fungal P450s are known to be involved in synthesizing many secondary metabolites with potential biotechnological applications [19,22,23]. It is now well-established that P450s play a crucial role in an organism’s adaptation vis à vis the lifestyle of organisms that impact the P450 content in their genome [24]. A recent study published by our laboratory reported this phenomenon in *Pezizomycetes* [25]. Despite belonging to the same class, saprophytes and ectomycorrhizal species were found to have diverse P450 complements characteristic of their lifestyles [25].

A handful of studies on lichens reported the involvement of P450s in synthesizing secondary metabolites, where P450s were found to carry out a critical enzymatic reaction [26,27,28,29]. Despite the importance of P450s in the biosynthesis of secondary metabolites in lichens, to date, a systematic analysis of P450s in these organisms has yet to be reported. Thus, this study is aimed to address this research gap.

In this study, we have selected five lichens from the fungal class *Lecanoromycetes* (Table 1). General information on these five *Lecanoromycetes* is presented in Table 1. The criteria for selecting these five *Lecanoromycetes* is that their genomes were well annotated, published, and available for public use at the Joint Genome Institute’s MycoCosm site [30]. Furthermore, detailed information on the secondary metabolite biosynthetic gene clusters (BGCs) and the genes forming the BGCs is also available. This allows us to easily identify P450s, carry out their annotation and phylogenetic analysis, and identify their role in secondary metabolism.

As part of this study, we also named characterized lichen P450s reported in the literature to enable researchers to use the proper P450 names. Last, we also compared P450s between *Lecanoromycetes* and *Pezizomycetes* to see any commonalities or diversity as they belong to the same phylum, *Ascomycota* [36].

## 2. Materials and Methods

### 2.1. Species and Databases

The study employed five *Lecanoromycetes* (Table 2). All *Lecanoromycetes* genomes used in the study have been published and are freely accessible to the public via the Joint Genome Institute’s MycoCosm site [30].

### 2.2. Genome Data Mining and Identification of P450s

Genome data mining and identification of P450s in *Lecanoromycetes* was carried out following the method described elsewhere [25]. Each set of annotated proteins from *Lecanoromycetes* was briefly searched for P450s using the InterPro code “IPR001128”. The hit protein sequences were downloaded and screened for P450 motifs, such as EXXR and CXG [37,38].

Proteins containing one of these motifs or short in length (fewer than 350 amino acids) were classified as P450 fragments. In comparison, proteins containing fewer than 350 amino acids but showing a similarity to P450s were classified as partial P450s. Proteins with all of the P450 characteristic motifs were considered P450s. P450 family and subfamily analyses were performed on proteins grouped as P450s. The P450 fragments that were <350 amino acids long were considered incomplete. These sequences were produced by annotation pipelines that are in use by the genome producers. We rely on that software for the accurate assembly of the protein sequences. When the sequences are short, there is a high probability that they come from pseudogenes as the same software accurately predicted full-length P450 proteins from the same genome. Furthermore, the DNA sequence was manually copied and pasted such that 5000 bp upstream and downstream were taken, and genes were predicted using GENSCAN [39].

### 2.3. Assigning P450 Family and Subfamily

We used a BLAST (Basic Local Alignment Search Tool) analysis of *Lecanoromycetes* P450s against all fungal sequences listed on the Cytochrome P450 Homepage [18] to estimate the percentage identity with named homolog P450s in order to determine P450 families and subfamilies. The proteins were then classified into distinct P450 families and subfamilies following the International P450 Nomenclature standards [17,18]. Proteins with >40% and >55% amino acid identity were classified as belonging to the same P450 family and subfamily. New P450 families were assigned to proteins that had less than 40% similarity with the closest P450 homologs. The P450s are included in Supplementary Dataset S1, together with the names given to them and the P450 fragment sequences.

### 2.4. Phylogenetic Analysis

Phylogenetic analysis of P450s was carried out following the method described elsewhere [25]. The P450 protein sequences were aligned using the MAFFT v6.864 [40] program with an automatically optimized model option available at the T-REX web server [41]. The alignments were then automatically subjected to inference and optimization of the tree by the Trex web server [41] with its embedded weighting procedure. Finally, the best-inferred tree was visualized, colored, and generated by the Interactive Tree Of Life (iTOL) [42].

### 2.5. P450 Family Conservation Analysis

Heat maps were created from the P450 family data using the method described in the literature [25]. The data were shown as −3 for the absence of a gene (green) and +3 for the presence of a gene (red). A tab-delimited file was imported into the multi-experiment viewer (MeV) [43]. The data were clustered using a Euclidean distance metric and hierarchical clustering. The vertical axis was made up of *Lecanoromycetes*, and the horizontal axis was made up of P450 families.

### 2.6. Identification of P450s That Are Part of Secondary Metabolite Biosynthetic Gene Clusters

P450s that are part of secondary metabolite biosynthetic gene clusters (BGCs), were identified following the method described elsewhere [25] with slight modification. Each of the BGCs listed in the Joint Genome Institute’s MycoCosm site [30] for each of the species was manually searched for gene/protein sequences in that cluster. If a P450 was listed as a part of the cluster, the P450 protein ID and its sequence were noted and matched with its assigned name. The cluster ID, cluster type, scaffold information (genomic location), and P450s part of the cluster were presented in table format as a standard practice.

### 2.7. Assigning the P450 Family and Subfamilies to the Functionally Characterized Lichen P450s

A handful of P450s from lichens are functionally characterized and shown to be involved in synthesizing different secondary metabolites [26,27,28,29]. The P450 sequences from these lichens were retrieved from the literature, and P450 families and P450 subfamilies were assigned to these P450s, as indicated in Section 2.3.

## 3. Results and Discussion

### 3.1. Lecanoromycetes Have More P450s than Pezizomycetes

**Figure 1:**
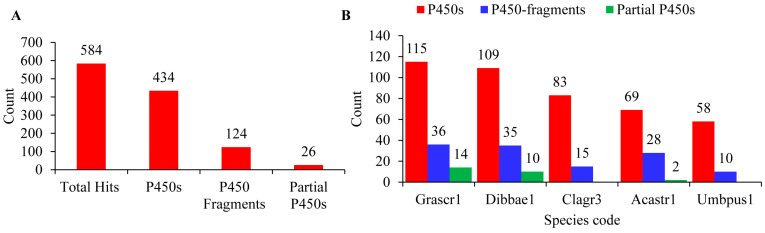
Analysis of P450s in the five *Lecanoromycetes*. Identification of P450s (**A**) and species level comparison of P450s (**B**) in the five *Lecanoromycetes*. Species codes: Grascr1, Graphis scripta CBS 132367; Dibbae1, *Dibaeis baeomyces*; Clagr3, *Cladonia grayi* Cgr/DA2myc/ss; Acastr1, *Acarospora Strigata* CBS 132363; Umbpus1, *Umbilicaria pustulata*.

A genome-wide analysis of P450s in the five *Lecanoromycetes* yielded 584 hit proteins (Figure 1A). Further examination of hit proteins for typical P450 motifs (as indicated in Section 2.2) revealed that not all hit proteins are P450s. Among the hits, 434 have all the typical P450 motifs and are considered P450s; of the rest, 124 hit proteins were identified as P450 fragments and 26 hit proteins as partial P450s (Figure 1A). A species-level analysis indicated that *Cladonia grayi* Cgr/DA2myc/ss and *Umbilicaria pustulata* have no partial P450s (Figure 1B).

The number of P450s in the five *Lecanoromycetes* ranged from 58 to 115 P450s, with an average of 87 P450s. Among *Lecanoromycetes*, *Graphis scripta* CBS 132367 has the highest number of P450s (115), and *U. pustulata* has the lowest number of P450s (58) in their genome (Figure 1B). The average number of P450s in *Lecanoromycetes* is 2.5 times higher than the *Pezizomycetes* [25], suggesting that Lecanoromycetes have more P450s in their genome. A list of P450s identified in *Lecanoromycetes*, along with their assigned name and protein IDs, are presented in Supplementary Dataset S1.

### 3.2. Lecanoromycetes Have Higher P450 Family Diversity than Pezizomycetes

**Figure 2:**
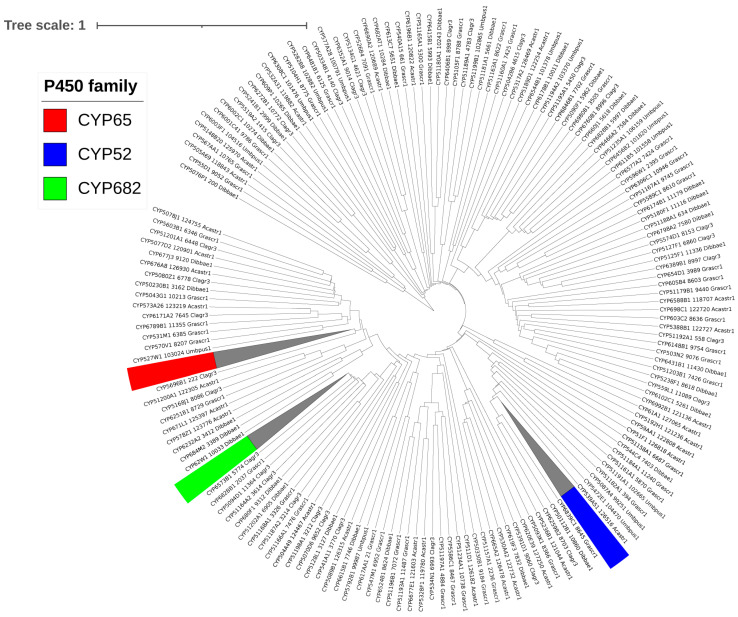
Phylogenetic analysis of *Lecanoromycetes* P450s. A single P450 from each of the 178 P450 families was used to construct the tree, except for P450 families with ten or more members highlighted in different colors; all members were included, and the branch collapsed. A high-quality figure with all 434 P450s is presented in Supplementary Figure S1.

The 434 P450s identified in the five *Lecanoromycetes* were categorized into 178 P450 families and 345 P450 subfamilies following the International P450 Nomenclature Committee’s criteria [17] and the phylogenetic analysis (Figure 2 and Table 3). As stated in Section 2.3, P450s were allocated to various P450 families and subfamilies based on the percent sequence identity; however, phylogenetic analysis is crucial in determining which subfamilies belong to P450s that are borderline with the identified homolog P450s and fall into the range of about 55% identical. These borderline P450s were assigned to the correct subfamilies based on alignment on the evolutionary tree. Additionally, phylogenetic analysis can be used to determine evolutionary links, such as how closely two species’ P450 genes are related.

Only 6 P450 families, CYP65, CYP59, CYP52, CYP682, CYP584, and CYP6001, have ten or more members among the 178 identified families in the five *Lecanoromycetes*. As a result, it is safe to conclude that P450 families in *Lecanoromycetes* have not bloomed (a single P450 family with many genes) (Table 3). It is worth noting that the same phenomenon was found in *Pezizomycetes*, where no P450 family bloomed [25]. In contrast to what was found for Lecanoromycetes and *Pezizomycetes*, P450 family blooming was observed in several fungal species [21,44]. P450 subfamily-level blooming was also not observed for *Lecanoromycetes*, whereas the blooming of two P450 subfamilies was observed for *Pezizomycetes* [25]. Compared to the 19 *Pezizomycetes*, the 5 *Lecanoromycetes* have more P450 families (153 vs. 178), indicating higher P450 family diversity in the *Lecanoromycetes*.

**Figure 3:**
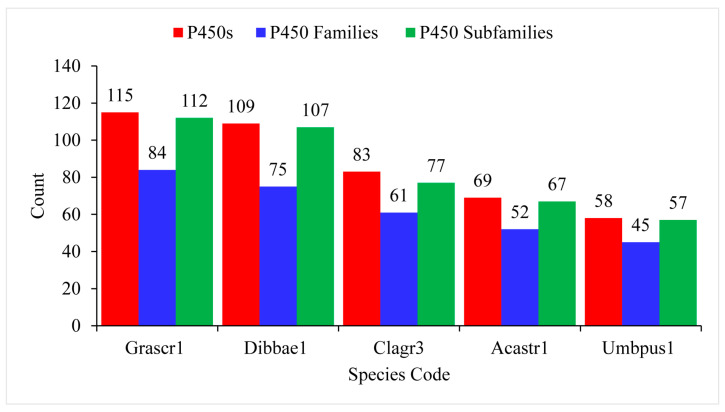
Comparative analysis of P450s, P450 families, and P450 subfamilies in five the *Lecanoromycetes*. Detailed information is presented in Table S1. Species codes: Grascr1, *Graphis scripta* CBS 132367; Dibbae1, Dibaeis baeomyces; Clagr3, *Cladonia grayi* Cgr/DA2myc/ss; Acastr1, *Acarospora Strigata* CBS 132363; Umbpus1, *Umbilicaria pustulata*.

The number of P450 families ranged from 45 to 84, with an average of 63 P450 families, and the number of P450 subfamilies ranged from 112 to 57, with an average of 84 P450 families (Figure 3 and Table S1). *G. scripta CBS 132367* and *U. pustulata* had the highest and lowest number of P450 families and P450 subfamilies in their genomes (Figure 3 and Table S1). *Lecanoromycetes* have a higher number of P450 families and P450 subfamilies per species compared to *Pezizomycetes* [25], indicating that species belonging to the former group have the highest P450 family and subfamily diversity.

**Figure 4:**
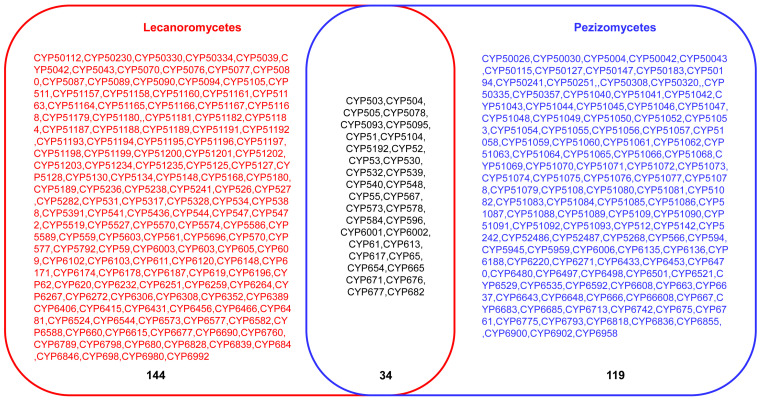
Comparative analysis of P450 families between *Lecanoromycetes* and *Pezizomycetes*. The number indicates the total number of P450 families.

A comparative analysis of P450s profiles between Lecanoromycetes and *Pezizomycetes* revealed that species belonging to these classes have different P450 profiles with few similarities (Figure 4). Despite both classes belonging to the same phylum, *Ascomycota* [36], and subphylum, *Pezizomycotina*, only 34 P450 families are common, and quite a large number of P450 families were found to be unique to *Lecanoromycetes* (144 P450 families) and *Pezizomycetes* (119 P450 families) (Figure 4). This indicates that after speciation, the lifestyle or ecological niches played a key role in shaping the P450 content in these species, as observed for bacterial [24,45] and some fungal species [21,44,46].

### 3.3. Fifteen P450 Families Are Conserved in the Five Lecanoromycetes

**Figure 5:**
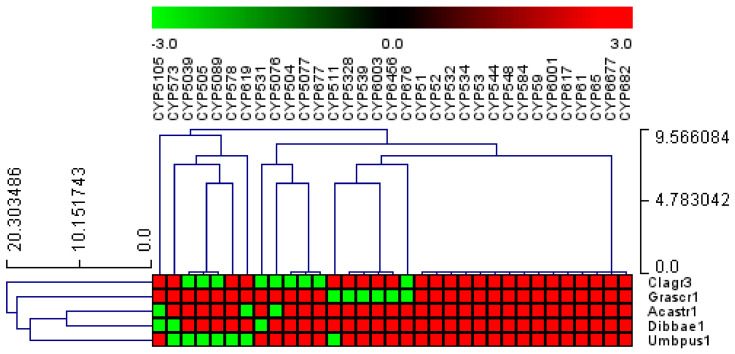
Analysis of P450 family conservation in the five Lecanoromycetes. P450 families that are conserved in three or more species are presented. The heat map shows that the P450 family is either present (red) or absent (green) in *Lecanoromycetes*. The horizontal axis is made up of P450 families, and the vertical axis is made up of *Lecanoromycetes*. Table S2 provides a thorough examination of P450 family conservation in *Lecanoromycetes*.

P450 family conservation analysis revealed that only 15 out of 178 families, namely, CYP51, CYP52, CYP532, CYP534, CYP53, CYP544, CYP548, CYP584, CYP59, CYP6001, CYP617, CYP61, CYP65, CYP6677, and CYP682, are conserved in five Lecanoromycetes (Figure 5). The conservation of these P450 families across the five Lecanoromycetes suggests that these P450 families may play important roles. It is well established that CYP51 and CYP61 are involved in sterol biosynthesis [47,48,49], which is required for cell wall membranes, and that CYP6001 members are involved in fatty acid oxidation [50]. CYP53 members play a key role in detoxifying toxic molecules in fungi; thus, CYP53 has been proposed as a common alternative anti-fungal drug target [21]. CYP52 members are involved in the oxidation of alkanes and fatty acids and the biosynthesis of sophorolipids [51,52]. CYP65 members are involved in the biosynthesis of sesquiterpenoid mycotoxins [53]. From the characterized homolog P450s functions, it is clear that members belonging to these P450 families play an important role in fungi and, thus, one can expect the conservation of these P450 family members in Lecanoromycetes.

### 3.4. Fifty-Five P450s from the Five Lecanoromycetes Are Involved in the Biosynthesis of Secondary Metabolites

A biosynthetic gene cluster (BGC) analysis revealed that 73 P450s are part of different BGCs, indicating their possible role in synthesizing secondary metabolites (Table 4). Among the 73 P450s, 18 were P450-fragments/partial P450s, and thus only 55 P450s can be assumed to be involved in the biosynthesis of secondary metabolites (Table 4). Among the five Lecanoromycetes, *G. scripta* CBS 132367 have the highest number of P450s that are part of BGCs (19 P450s), followed by *C. grayi* Cgr/DA2myc/ss v2.0 (13 P450s), *Dibaeis baeomyces* (12 P450s), *A. strigata* CBS 132363 (9 P450s), and *U. pustulata* (2 P450s).

The analysis of P450 families that are part of BGCs revealed that the CYP65 family has the highest number of members (seven members) followed by CYP5039 (five members) and CYP5077 (three members). Eight P450 families have only two members, and twenty-four P450 families have only a single member as part of BGCs (Table 4).

**Figure 6:**
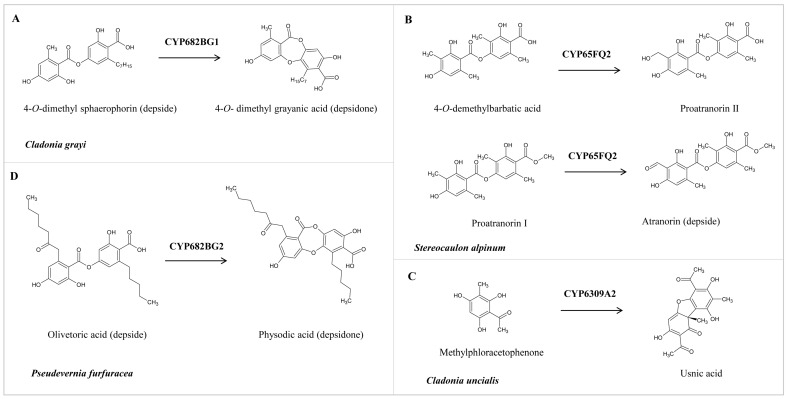
Role of lichen P450s in the biosynthesis of different secondary metabolites. (**A**,**B**) Conversion of depside to depsidone by CYP682BG1 and CYP682BG2. (**C**) Oxidation of 4-O-demethylbarbatic acid and proatranorin I by CYP65Q2. (**D**) Oxidative dimerization of methylphloracetophenone to usnic acid by CYP6309A2.

As reported in the literature, the functional characterization of a handful of P450s from lichens revealed that P450s perform a critical catalytic reaction in synthesizing secondary metabolites [26,27,28,29] (Figure 6). These P450 sequences were retrieved from the literature and annotated in this study to enable their correct identification. These P450s and their assigned names and protein sequences are presented in Supplementary Dataset S1.

To enable researchers to identify the P450 and its functions, we provide detailed information on lichen P450s and their elucidated functions. CYP682BCG1 from *C. grayi* performs the oxidative coupling of 4-O-demethyl sphaerophorin (depside) to 4-O-demethyl grayanic acid (depsidone) via ether bond formation (Figure 6A) [26]. CYP65FQ2 from *Stereocaulon alpinum* catalyzes the oxidation of C-9 of 4-O-demethylbarbatic acid and proatranorin I to generate proatranorin II and atranorin (ß-orcinol depside), respectively (Figure 6B) [27]. CYP6309A2, named methylphloracetophenone oxidase, from *Cladonia uncialis*, performs the oxidative dimerization of methylphloracetophenone to usnic acid (dibezofurane derivative) (Figure 6C) [28]. CYP682BG1 from *Pseudevernia furfuracea* (physodic acid chemotype) performs the oxidative coupling of olivetoric acid (depside), leading to physodic acid (depsidone) synthesis via ether bond formation (Figure 6D) [29]. It is interesting to note that CYP682BG in *C. grayi* and *P. furfuracea* converts depside to depsidone irrespective of the side chains in depside.

## 4. Conclusions

The quest for novel compounds with potential biotechnological value is increasing, and researchers are probing previously unexplored venues. Due to their adaptability to practically all climatic conditions, lichens generate a broad spectrum of natural compounds that may help them protect themselves against infections or predators. Several studies have indicated that these compounds have potential biotechnological values. A few have been shown to act as anti-bacterial, anti-fungal, and anti-cancer agents. A handful of studies have tried to outline the biosynthetic pathway of these compounds and shed light on genes involved in synthesizing these compounds. Among these genes, tailoring enzymes such as P450s have been found to play a key role in synthesizing these compounds in lichens. The results from this study provide important information on these enzymes in lichens. Also, this study indicates that speciation and adaptation to diverse ecological niches led to the development of a diverse P450 complement in *Lecanoromycetes* and *Pezizomycetes*. More lichen genomes are under investigation for P450 profiles to understand evolutionary aspects and assess their role in secondary metabolism. Considering the unique reactions performed by lichen P450s, one can predict that a great potential for lichen P450s is waiting to be discovered.

## Figures and Tables

**Table 1:** Information about the five *Lecanoromycetes* used in the study and their general characteristics.

Species Name	General Information	Reference
* Acarospora strigata * CBS 132363	* A. strigata * is a crustose lichen that grows in dry conditions, primarily on rocks, and is prevalent in North America’s southwestern deserts.	[31]
*Cladonia grayi* Cgr/DA2myc/ss	It is a well-studied lichen that can be grown easily in the laboratory. This lichen is used to understand the development of symbiosis between fungus and algae as the symbionts of this lichen can be separated and propagated, and symbiotic relationships can be regenerated.	[32]
*Dibaeis baeomyces*	*D. baeomyces* is a fruticose lichen known as pink earth lichen because it produces pink apothecia 1–4 mm in diameter and 6 mm tall. It can be found anywhere, from North America to the Arctic Circle. It grows in full sun and on unstable soils such as loose sand or dry clay. It also likes acidic soil over neutral or alkaline soil. It may spread swiftly on disturbed ground, a preferred habitat for this lichen.	[31]
*Graphis scripta* CBS 132367	*G. scripta* is a lichen with a growth pattern that looks like writing. It is common throughout the British Isles, especially in unpolluted zones. It occurs on moderately shaded, smooth bark of various trees.	[31,33]
*Umbilicaria pustulata*	*U. pustulata* forms a symbiotic connection with green algae of the genus *Trebouxia* and may live in various latitudes and altitudes across continental Europe. This lichen lives on rocks.	[34,35]

**Table 2:** The table lists the *Lecanoromycetes* used in this study and their genome database links and reference articles. The genome database of *Lecanoromycetes* was last accessed on 31 July 2023.

Species Name	Genome Database Link	Reference
* Acarospora Strigata * CBS 132363	https://mycocosm.jgi.doe.gov/Acastr1/Acastr1.home.html	[31]
*Cladonia grayi* Cgr/DA2myc/ss	https://mycocosm.jgi.doe.gov/Clagr3/Clagr3.home.html	[32]
*Dibaeis baeomyces*	https://mycocosm.jgi.doe.gov/Dibbae1/Dibbae1.home.html	[31]
*Graphis scripta* CBS 132367	https://mycocosm.jgi.doe.gov/Grascr1/Grascr1.home.html	[31]
*Umbilicaria pustulata*	https://mycocosm.jgi.doe.gov/Umbpus1/Umbpus1.home.html	[35]

**Table 3:** Analysis of P450 family and subfamily count in *Lecanoromycetes*. The name of P450 families (F), the subfamilies (SF), and the number of members in each subfamily (C) are presented in the table.

F	SF	C	F	SF	C	F	SF	C	F	SF	C
CYP51	F	8	CYP561	Q	1		B	1		B	1
CYP52	AA	2	CYP567	AA	1		C	3	CYP6306	C	1
	AG	1		E	1		D	1	CYP6308	C	1
	AJ	3	CYP570	V	1	CYP5078	E	1		D	1
	AZ	1	CYP573	A	2	CYP5080	J	2	CYP6352	A	1
	BA	1		D	1	CYP5087	Z	1		B	1
	K	4	CYP577	A	1	CYP5089	A	2	CYP6389	B	1
	P	2	CYP578	AA	1		B	1	CYP6406	B	1
	Z	1		AB	2		C	1	CYP6415	B	1
CYP53	A	5		AC	1	CYP5090	D	1	CYP6431	B	1
CYP55	D	1		Y	1	CYP5093	F	1	CYP6456	B	3
CYP59	AA	1		Z	1	CYP5094	K	1		D	1
	AB	1	CYP584	AA	1	CYP5095	D	2	CYP6466	A	1
	AC	1		AP	1	CYP5104	N	1		B	1
	AD	1		AQ	1		B	1	CYP6481	B	1
	AE	2		AR	1	CYP5105	H	1	CYP6524	B	1
	U	4		AS	2		A	1		C	1
	V	1		AT	1		E	3	CYP6544	B	1
	W	1		AU	1	CYP5125	F	1	CYP6573	B	1
	X	3		AV	2	CYP5127	F	1	CYP6577	A	1
	Y	1		AX	1	CYP5128	F	1	CYP6582	B	1
	Z	1		E	1		B	1	CYP6588	B	1
CYP61	A	6		J	1	CYP5130	L	1	CYP6615	B	1
	B	1		L	2	CYP5134	A	1	CYP6677	A	1
CYP62	W	1	CYP596	W	1	CYP5148	G	1		B	1
CYP65	AU	1	CYP603	C	1	CYP5168	B	1		C	1
	DY	1	CYP605	B	1		J	1		D	1
	EP	1	CYP609	P	1		K	1		E	1
	FC	1		Q	1	CYP5180	L	1		F	1
	FD	1	CYP611	B	1		E	1	CYP6690	A	1
	FE	1		C	1	CYP5189	F	1	CYP6760	B	1
	FF	1	CYP613	C	1	CYP5192	D	1	CYP6789	B	1
	FG	1		T	1		G	1	CYP6798	A	1
	FH	1		U	1	CYP5236	H	1	CYP6828	B	1
	FJ	2	CYP617	AA	1	CYP5238	B	1	CYP6839	B	1
	FK	1		AB	1		E	1		C	1
	FL	1		AC	1	CYP5241	F	1	CYP6846	B	1
	FM	1		AD	1	CYP5282	B	1	CYP6980	B	1
	FN	1		G	3	CYP5317	B	1	CYP6992	B	1
	FP	1		Z	1	CYP5328	A	1	CYP50112	B	1
	FQ	1	CYP619	C	1		E	2	CYP50230	B	1
	FR	2		F	1		F	1		C	1
	FS	1		N	1		G	1	CYP50330	B	1
	FT	1	CYP620	E	1		H	1	CYP50334	B	1
	FU	1	CYP654	D	1		J	1	CYP51157	A	1
	FV	1	CYP660	J	1		K	1	CYP51158	A	1
CYP503	N	1	CYP665	A	1	CYP5388	L	1	CYP51160	A	1
CYP504	A	4	CYP671	L	1	CYP5391	B	1	CYP51161	A	1
CYP505	A	2	CYP676	A	2	CYP5436	D	1	CYP51163	A	1
	AL	1		C	1		C	1	CYP51164	A	1
	AM	1		E	1	CYP5472	D	1	CYP51165	A	1
CYP511	A	1	CYP677	A	1	CYP5519	E	1	CYP51166	A	1
	D	1		H	1		A	1		B	1
	E	1		J	3	CYP5527	B	1	CYP51167	A	1
CYP526	AB	1	CYP680	C	1	CYP5570	B	1	CYP51168	A	1
	B	1		F	1	CYP5574	C	1	CYP51179	A	1
CYP527	R	1	CYP682	AT	1	CYP5586	D	1		B	1
	W	1		AU	1	CYP5589	C	1	CYP51180	A	1
	X	1		AV	1	CYP5603	C	2	CYP51181	A	1
CYP530	A	3		AW	1	CYP5696	B	1		B	1
CYP531	M	1		AX	1	CYP5792	B	1	CYP51182	A	1
	N	1		AY	1	CYP6001	B	1	CYP51184	A	1
	P	1		AZ	1		A	3	CYP51187	A	1
	Q	1		B	2		C	5	CYP51188	A	1
CYP532	A	5		BA	1	CYP6002	J	3	CYP51189	A	1
	U	1		BB	1	CYP6003	C	1	CYP51191	A	1
	V	1		BC	1		A	1	CYP51192	A	1
	W	1		BD	1		E	1	CYP51193	A	1
	X	1		BG	1		D	1	CYP51194	A	1
CYP534	D	1		BE	1	CYP6102	F	1	CYP51195	A	1
	M	1		BF	1		B	1	CYP51196	A	1
	N	1	CYP684	P	1	CYP6103	C	1		B	1
	P	1		M	1	CYP6120	B	1	CYP51197	A	1
	Q	1	CYP698	N	1	CYP6148	A	1	CYP51198	A	1
CYP539	A	4	CYP5039	C	1	CYP6171	B	1	CYP51199	A	1
CYP540	A	1		D	3	CYP6174	A	2		B	1
	G	1	CYP5042	E	2	CYP6178	B	1	CYP51200	A	1
CYP541	A	1		B	2	CYP6187	B	1		B	1
	B	1		D	1		B	1	CYP51201	A	1
CYP544	C	4	CYP5043	E	1		C	1	CYP51202	A	1
	D	1	CYP5070	G	1	CYP6196	D	1	CYP51203	A	1
CYP547	M	1	CYP5076	D	2	CYP6232	B	2		B	1
CYP548	A	4		L	1	CYP6251	A	1	CYP51234	A	1
	AJ	2		M	1	CYP6259	B	1	CYP51235	A	1
	BF	1		N	1	CYP6264	B	2			
	BG	1	CYP5077	P	1	CYP6267	A	1			
CYP559	L	1		A	1	CYP6272	A	1			

**Table 4:** Comparative analysis of secondary metabolite gene clusters (BGCs) P450s in the five Lecanoromycetes. BGCs information is retrieved from the Joint Genome Institute’s MycoCosm site [30]. P450s that are part of BGCs were identified and are presented with their assigned names.

*Acarospora strigata* CBS 132363						
Cluster Id	Cluster Type	Scaffold	Size (bp)	P450(s)		
Acastr1.5	NRPS	scaffold_2955:14024-25341	11,317	CYP527G2-fragment2	CYP527G2-fragment1	
Acastr1.7	PKS	scaffold_1025:541-17362	16,821	CYP6588B1		
Acastr1.9	DMAT	scaffold_8982:48845-71243	22,398	CYP5082-fragment1	CYP50105-fragment1	
Acastr1.11	PKS	scaffold_1754:2324-28832	26,508	CYP65FT-fragment1		
Acastr1.13	PKS	scaffold_11468:19115-30956	11,841	CYP548W-fragment1		
Acastr1.17	PKS	scaffold_541:14065-35374	21,309	CYP65FP1	CYP65FM1	
Acastr1.18	NRPS	scaffold_5370:9699-29989	20,290	CYP5078J1		
Acastr1.21	PKS	scaffold_3284:7180-23443	16,263	CYP5077C4		
Acastr1.22	PKS	scaffold_16232:29-22526	22,497	CYP676-fragment2	CYP5039D2	
Acastr1.28	PKS-Like	scaffold_8394:131532-167701	36,169	CYP5317A2	CYP665A2	
Acastr1.29	NRPS	scaffold_14549:3269-14411	11,142	CYP613-fragment1		
Acastr1.31	PKS	scaffold_7007:269-35975	35,706	CYP584E29		
***Cladonia grayi* Cgr/DA2myc/ss**						
Clagr3.4	PKS	scaffold_00063:22905-52715	29,810	CYP6171A2		
Clagr3.7	NRPS-Like	scaffold_00142:753-20882	20,129	CYP5436C1		
Clagr3.10	NRPS	scaffold_00016:35094-74834	39,740	CYP51198A1	CYP51187A2	
Clagr3.12	PKS	scaffold_00015:30884-46489	15,605	CYP532X1		
Clagr3.22	PKS	scaffold_00006:61108-75768	14,660	CYP5519A2		
Clagr3.28	NRPS-Like	scaffold_00088:9809-27658	17,849	CYP5042-fragment1		
Clagr3.35	PKS	scaffold_00038:232070-257829	25,759	CYP619C8	CYP6573B1	
Clagr3.37	PKS-Like	scaffold_00139:36187-66034	29,847	CYP532W1	CYP5436D1	
Clagr3.39	DMAT	scaffold_00027:92959-116616	23,657	CYP5042B6	CYP5134G1	
Clagr3.43	PKS	scaffold_00126:23596-42042	18,446	CYP65-fragment5	CYP6171A1	
***Dibaeis baeomyces***						
Dibbae1.2	PKS	scaffold_20:120953-140362	19,409	CYP5472C-fragment2	CYP5472C-fragment1	
Dibbae1.5	NRPS	scaffold_83:62571-93764	31,193	CYP6845B1-partial	CYP5589B2-partial	
Dibbae1.9	TC	scaffold_66:13303-22596	9293	CYP511A4		
Dibbae1.14	PKS	scaffold_2:20594-91671	71,077	CYP684M2	CYP6232A2	
Dibbae1.16	PKS	scaffold_7:187972-207957	19,985	CYP5077B6		
Dibbae1.20	PKS	scaffold_12:35918-72399	36,481	CYP65FJ1	CYP5039E1	CYP5039D4
Dibbae1.25	NRPS-Like	scaffold_141:42837-63865	21,028	CYP676C1	CYP6001C40	
Dibbae1.26	PKS	scaffold_54:22464-49793	27,329	CYP5571B1-fragment1		
Dibbae1.27	TC	scaffold_545:4754-14401	9647	CYP682BF1		
Dibbae1.39	NRPS-Like	scaffold_48:15983-27357	11,374	CYP677J3		
Dibbae1.40	PKS	scaffold_48:43672-64451	20,779	CYP6267B1-partial		
Dibbae1.47	PKS-Like	scaffold_166:20983-59475	38,492	CYP682AU1		
***Graphis scripta* CBS 132367**						
Grascr1.2	NRPS	scaffold_405:10641-23912	13,271	CYP5589C1	CYP613T1	
Grascr1.3	HYBRID	scaffold_404:1464-19085	17,621	CYP605B4		
Grascr1.5	NRPS-Like	scaffold_24:156299-161893	5594	CYP65FV1		
Grascr1.6	NRPS	scaffold_27:55736-86594	30,858	CYP617Z1		
Grascr1.12	PKS	scaffold_3:286280-316117	29,837	CYP65FL1		
Grascr1.15	NRPS	scaffold_227:20834-45351	24,517	CYP5589C2	CYP613U1	
Grascr1.18	NRPS-Like	scaffold_11:112852-134952	22,100	CYP540A15		
Grascr1.21	DMAT	scaffold_293:15177-27918	12,741	CYP5603B1		
Grascr1.30	PKS	scaffold_104:57527-90656	33,129	CYP65FJ2	CYP5039E2	CYP5039D3
Grascr1.33	NRPS-Like	scaffold_217:4559-13643	9084	CYP684N1		
Grascr1.34	NRPS	scaffold_191:8643-34575	25,932	CYP53A65		
Grascr1.38	PKS	scaffold_44:15391-39442	24,051	CYP65FT1		
Grascr1.41	PKS	scaffold_49:26297-55939	29,642	CYP5295A6- partial		
Grascr1.44	T1PKS	scaffold_324:9928-31442	21,514	CYP526B4	CYP552-fragment1	
Grascr1.46	NRPS-Like	scaffold_39:77314-95864	18,550	CYP5042E1		
Grascr1.49	TC	scaffold_200:1362-8058	6696	CYP654D1		
***Umbilicaria pustulata***						
Umbpus1.4	PKS	scaffold_2941:3803-26170	22,367	CYP5077A4		
Umbpus1.6	HYBRID (NRPS, T1PKS)	scaffold_1372:129382-152252	22,870	CYP5792B1		

## Data Availability

Not applicable.

## Supplementary Materials

The following supporting information can be downloaded at: https://www.mdpi.com/article/10.3390/microorganisms11102590/s1, Figure S1: Phylogenetic analysis of *Lecanoromycetes* P450s. P450 families populated in *Lecanoromycetes* are highlighted in different colors; Table S1: Comparative analysis of P450 families and subfamilies in Lecanoromycetes; Table S2: P450 family conservation analysis in 5 *Lecanoromycetes*; Supplementary Dataset S1: P450s identified and annotated in *Lecanoromycetes* are presented with their assigned name, followed by protein ID from the Joint Genome Institute MycoCosm database and species code. P450 fragments and partials identified in *Lecanoromycetes* are also listed.
