# Supplementary material for: Genome-Wide Analysis of the Cytochrome P450 Monooxygenases in the Lichenized Fungi of the Class Lecanoromycetes
Source: Microorganisms. 2023 Oct 19;11(10):2590. doi: 10.3390/microorganisms11102590 (PMC10608907; doi:10.3390/microorganisms11102590)
Supplement: Supplementary file 1 [file microorganisms-11-02590-s001.zip › Figure S1.pdf]

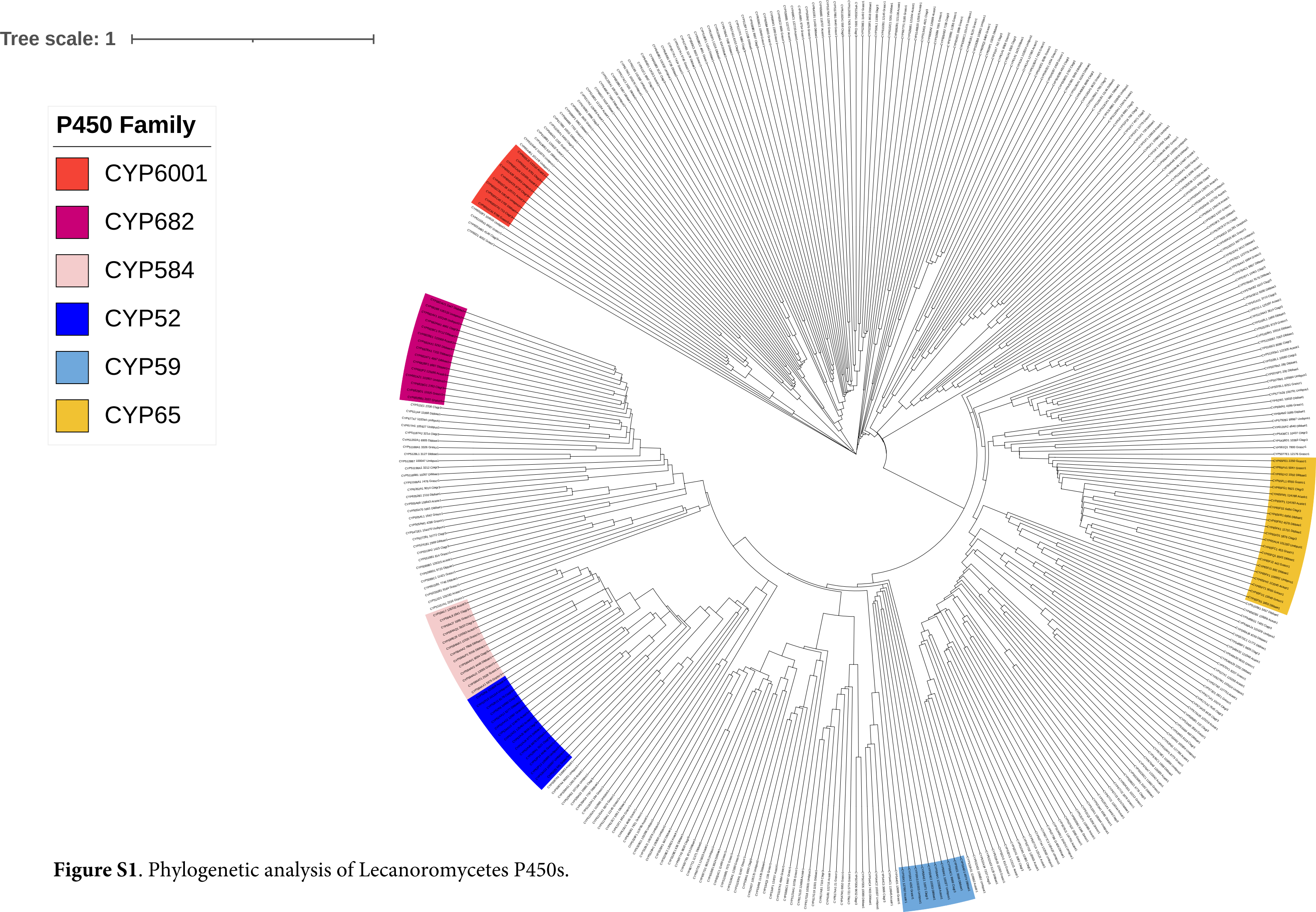

Figure S1. Phylogenetic analysis of Lecanoromycetes P450s.
