# Supplementary material for: Genome-Wide Analysis of the Cytochrome P450 Monooxygenases in the Lichenized Fungi of the Class Lecanoromycetes
Source: Microorganisms. 2023 Oct 19;11(10):2590. doi: 10.3390/microorganisms11102590 (PMC10608907; doi:10.3390/microorganisms11102590)
Supplement: Supplementary file 1 [file microorganisms-11-02590-s001.zip › Supplementary Dataset S1.pdf]

Genome-Wide Analysis of the Cytochrome P450 Monooxygenases in the Lichenized Fungi of the Class *Lecanoromycetes*

Gugulethu Mlambo [1], Tiara Padayachee [1], David R. Nelson [2,*] and Khajamohiddin Syed [1,*]

[1] Department of Biochemistry and Microbiology, Faculty of Science, Agriculture and Engineering, University of Zululand, Vulindlela, KwaDlangezwa 3886, South Africa; gugulethumlambo75@gmail.com (G.M); teez07padayachee@gmail.com(T.P.)

[2] Department of Microbiology, Immunology and Biochemistry, University of Tennessee Health Science Center, Memphis, TN 38163, USA

* Correspondence: drnelson1@gmail.com (D.R.N.); syedk@unizulu.ac.za (K.S.); Tel.: +1-901-448-8303 (D.R.N.); +27-0359026857 (K.S.)

Supplementary Dataset S1

P450s identified and annotated in *Lecanoromycetes* are presented with their assigned name, followed by protein ID from the Joint Genome Institute MycoCosm database and species code. P450 fragments and partials identified in *Lecanoromycetes* are also listed.

Acarospora strigata CBS 132363

>CYP531P1|119065|Acastr1
MAILGLLFQALLGVTALFITNLLWTYYNSPLKPIPGPFLAKFSNLWRFFDVWDGRSELTLRMLHAKY
GSAVRIGPNVVSLSDPRLIKTIYSTRGEYLKSEFYKVNDVKSGNAIVSNMFSTLSNDYHAMQLKPIQR
HYTLNALMDFEPLVDKTIDTFRQEMEKRFVGPGKVCIIGDWLLYCATPTLGVKEIAWDVIGEITFSQ
PMGFLKTGADVEHILSGADTSLDYFSVIGQIPRLDFLFDKNPIHRIGPPSFGTVVTMSIQRTIARINGT
DQHSAGTQKDFLDQFIEKKKEAAWIDDNQVVGWLCINMFAGSDTTAITLRAIVYFVLGSDTTAITL
RAIVYFVLKNPHVHRKLHQELDAANLPMRISYKAAQSLPYLGAVIEEAMRMHPAVGLLLERVVPSS
GLTLPDGRFIAPDTIVGLNAWVVHQDKTVFGQDADSFNPERWLQGPDETVTEFQNRRSRMREANL
TFGAGNRVCLGKSLSMLEIYKFIATLFLNYQMELVDPAKEWHVQNSWFVRESGMDVTIKRREELSG
*
>CYP677J1|119344|Acastr1
MLILNIYLIQIAVTAISIYLVGWIIYSRYFHPLHSIPGPFLASISRAWIIFKTMTGDMEHTQRALHKKHG
YLVRIAPNEVACSDPEAIKIIYGTKIVFTKTDFYDAWAPPNNGYVGHFPARDEKEHSERRRIVNNVY
SMSSVLESEKAIDSCTQVFCETMRDFAKQKSVVDLGLWINMYAFDVLGVLFYGKTFGFMNERTDV
GNYMKAIDSLLPAFTIGGTVPSYLTKLYLISTILFSPSIRGALGAVKRLESASEAAVKRRKQEMEENKD
DKRDLLRKMLEIDADRGQKINFTFPHICVESHSSIFAGADTTAIAINSILYHLMRNTAAYEKLTAEID
TAVADGTLSMPVAYAEAVKLPYLKACINEGMRLHPSVGLTMPRLVPAGGATISGFDFPEGYRIGIN
GAVVQYDKDVFGPDADNFNPNRWIEGDSVRMDKTMIQFGGGPRTCIGKNLSAGLCIDFRMYANIS
LSEIYKLIPQIVRVFHIRLVDLSKEWKTHNYWFNKQTGVYIYIKERAHESRYRDRDPSRAG*
>CYP6588B1|118707|Acastr1
MDTALTSLPLWQLQIGGFVAVLAAFLALRVVQYRYQLTQTRALPVADVKEGNWKEAISCASAKYP
DAPWILPTDPPRVVLPNSVMDEVRFLPENQLSLRKEVYKKMHGRYTDLGKDHPVGMEAIKTDLTN
NVGRMLPDLQEETIYALEREIGRCPGWTKVALYDKLLQLSALVNGRMFVGLPLCRDQTWIDMSIKY
TLDMVYGIRAVQKVNPLLRPLKAPWLSEIRNLAGYKKRAAQMLRPHIDEAVEARKASYNLITWML
NQMKDVDYELLASEQIFASFGAIHATCITVTNALYDLAAYPQYVAELRAEINDVVALEPDYRLRKA
ALPKLMKLDSFLRESQRMNPGNITSIQRIVTARNGIKLSNDFTIPHGTSIGFLHPLAPFVHPAPNLVTP
VDRIPNQPPLSEFYPFRHSEVRSLPGQMNYHQFAMTAQDNITFGHGPGSCPGRFFAGAEVKVILVEI
LRRYDVALGPNGEGETDGGGEKGFKRPTNIILPGSLQCVPDFTKMIYFKELKVVLPTTPKSES*
>CYP6582B1|119656|Acastr1
MPFLILGPIVRFAPNSLSFNTASAVESIYSSRKANSAKSDWYQCVRDSAGGFVSTFTVIDPVRHATKR
RLLSHAFSSEALRSMEPCIVGHIWTWIECLGLKLESASRHEWGSELDIGTLTNMLIFDILGDLCFGKSF
DMMKSPEHRFLVPLIPQATRGWYTVSRFSLPYLALNGLLVALCVLTYCPTHQLGYHPYTHLLRYILF
KTPLGPRYILFKTPLGPLVGGKTFEDNRIFRAFCTKALKARRQAENCTNGNLTSASSKGVRKDIFHY
LLHGKDPETGRGYAVGELECESVLLMVAGSESTSTAVAASIYYLIHNSDALEKLTLEVREAFASRDEI
RYEAGGKLMNLPYLRACLDESLRLTPSTPGHLPREVLRGGMLVDGEWIPSGTNVGASAYALHRNE
AYYPHADQFRPERWTCEEVRSGKGEAVTAREAFWAFSSGPTGCIGKHLAYMELSLAVAALSWSFDI
RAAESTRAMEQDSTARNVKHNASEFQVKDVFVGVGEGPVVQLRRR*
>CYP544C1|119945|Acastr1
KPPNTLPILGNGLLFLQARHKLFQWFTQCECRFGLETFEISVPTLPPGVVINDPKNLEFVLRRDDVFT
KGEFFRRRSWDLFGYGIINADGDLWKVQRKAGQRFFSPANLKSLVDVVLPLHLADTRSYLFQQARE
RRRVDLQHVFLDFTTRLMGKLAYDMDMQASSTFSEAFDFASGAIGERFQNPFWRLTDLFFGARLRR
AVSEVKRFGRSIVAAAVQRRSGKILEGDKDNSTYSNSISGSLINFLMDGISDHEIVADAALNYLSAGR
DTTAQSLTWTFYLLMRHPDIAQNVRNELRTLFPGQKVIHLSSANLPQPSSLPYTMAVFYESLRLHPP
VPFEIKQCETATTLPDGTFLPQGSVIVSCIWAMNRSKTSWGPDADCFRPERWLEEDFDNTLENHPE

GGKSEEKRWKVTSKTASEFPVFHGGPRSCLGRKMAELLGVYVIASLFWEFDFEEDKDKEEARERVS
KNSLTLPMEGGLPCYVAVREQIF*
>CYP5039D2|120184|Acastr1
MSSITEISGHLPLSSIPSLNLIPTIALACTVYLVILGIYRVYLSPLSKFPGPKLAGSDVVSQALTTLYQAY
YDMYHYGQFFKKLDQLHQEYGPIVRVNPHELHVCDPEFIDVLFTGNSQRRDKYKWIGRSILLPDST
VATISHELHRKRRAALNPYFSKASIRRLEPVIQQTLANLLDRMTQCGRDREVIPMSVVYKATTSDIIT
GYSFGESTEYLKREDYNEPFFSAVDANFAMAWPMTYIPWLGPLLSVIPPSVMGLVYPGLKSLWDMH
GQWSKQIDEIRNSKDISSDATVFHGLLNSSLPPCEKEPGRMRQEAQLVVLAGQDTTAQTLSATTYEL
LANPDKLKKLQVELAEALPDPDMVPTSAIVEKLPYLSAVIQEGLRLHPGALTRMTRVAPDKAMIYD
DKNGRKQWVIPAGVPVTMTARTVQMNATYFPDPDKFEPERWLDNARLDKYLLAFSKGTRICLGF
HLAYAELYLILAGIFRKYELYDGTGNQQSPTLALYDTVRERDIDPVIDLVVPYPAKGSHGLRLAVHA
*
>CYP6196B1|120822|Acastr1
MLYIVTAPKDVAEVYKKADILAWDGHLNQLFLNFGFNAESLKRAWLKPAHIDSRYKTVNPHQLSL
IHLVESIYAKQLLPGVHMDEMGKGFVAALQAALRLPNLKVCSIDKNIISNLLAFSENAWMLFYGLP
SFFASAVLTPQSAVLATFQQFADLPESLRNDQSWSVQQILIAQECVGIDLRSRACMLLMILWAANS
NVNNTAFWVLTHILFDEQLRKAVQQEVNAAWKSEQLDVKFLCANATVLDRTFHECLRLKAGAM
MGRKVLAPTRIGNKMLKPGGTVLIPSRQLHSNPNVWGPDHEHFAETRFAKNEGAETRFAKNEGLL
KHSSYRPFGGGRRLFALVAILIHRFDVKLNQDLHQEFPRLNVSTPALGVTGPAKGMDILVELSHVEK
NT*
>CYP5328E2|120823|Acastr1
MSAPLVDGRTDPDVQVMKVQEPVFLGLIWGGLALAIICCSLRFLARIRTFKKLLIDDYFVLLALAFLL
ANAIIWQIYARQMYYIMTVAAGLEMPGEGFAPLAESYAPLAESYFKATVAVIILFYSALWSIKVSFLL
FFKRLGTNVRGQKVIWWPVLGITLATYFACVGTIQYSCLVSSFEYLAANCRTPAATSFQQITLKLNC
AWDVTTDCLIMFIPFSMLRGVQMKWRRKAALSGIFSLVIITMVFAIVRTALVGSSNTDQPDSSWLYM
WSAIEASIAIIVACLATFRNLFSRETTRHKLEKPAVPASSNLFLGGNGRRNKIRDILDSLASMPDHAH
SGYQQQNENTSDAQSISNHHNHVVTIGVLESCSLYVVSCPHPLLSVLLPHFSPYYPLIFPRPRHKVLM
PYPLFVLVPLALSLGTVLYVLYGLLVNYNAARKTGLPLIVLPFDCGNPLWLIIDRKIVQLVRRIPFCSG
TFTRFNWRGWEIWDRYRAHQQLGDAILFVTPGKNYLQLCDAEAVSELCDAEAVSEIFQRRADFPR
PPESTGKGLAHTQPPESTGKGLAHTQLKDIHNGLLEMLNIFGPNVGTTDGRQWQRHRRITASSFNE
HVHQRVWIESILQSTGLIHYWSRKSSVDSVATDTRTVSLHVMSGAIFGKSYPFRGADEDNSSTKEDSS
SYGEALRIILDRCIPLVVLGRKNLKHMTQAYEGEKRAMMRNDKLENNLMTSLVRASQANVDQKG
STTESHQEGLTEEEVYGNVFVFNFAGHDATANSLTIGICLLATRPDIQDWIAEEINAVLAGFDSKESS
YEAAFPRLPRCLAVVFETVRLYTAVAIAKSTGSSPQPLKLGASTVLIPKNTVIIPNYSALHTHPRYWG
HDSLEFEPSRWIIPDPHISTTNGYQASSSHPVEHLKELPTRNSPFVGWSGGARSCPGRKFAQVEFVGV
LVGLFRDFKVKPVALKGEDDGMARARLLDQIRTDTGMRLLLQMLHPERAVLEWSRR*
>CYP5077D2|120901|Acastr1
MSSSYSLLHSGDVDLTKAALLVTFLTVLYGLYHVSRSYLKLAHVPGPFLASLSNLSRVSWVLSKRAH
EIHIELHEKYGDLVRFGPNMVSVADPAEIPTIYPMRPGFVKSDFYKVLLPYSKGKSLPSLFGTADENI
HKALKRPIAPIFSLSNVVSFEGYVDSTIRVLFEQLDNRHVQTGDVCDFGAWLQMFAFDVMGELTFS
KRLGFLESGKDVDGVMRMIWEHFKAAAPVSQMPWIDRFWNKNPLLAYLRPSSASPILKFASTRAK
ERMDLEKDPDQEKPELNNRDFLSRFIEAQSKDPQVPEWAVTVWTFSNITAGSDSTAVVLRTVFYNL
LKHPTTLRRLQAELQDTARQGRLSLPCTWKETRDLPYLDACVKEGLRMHPPFGLPFERVVPSGGAT
ICGQRFEEGTVVGMSAWVANRHKGTFGEDADVWRPERWLCEENPSIKNYS*
>CYP6992B1|121136|Acastr1
MAFEYFSQLTLFVPLSLLVVYFVYTKLTSKPLIPEHVPWVGRRSEFFSKGRANLRSFTRGRELLDDAY
YKFSKHGLNCVLPTFQGGEVLIPPAQIPWLLSQPEHVLNTKEVHRDSLEADYTMLNEKIVRSPVHE
DVIRRDLTRQLGNLTMDIMQELVAMDIMQELGSGFDEYWGYDTENWKEACVYENMMKIIARTSN
RVIVGLPLCRNEDFLRNVAKFSQDIPLSGQVIKLFPKFLKPLIGPIAVLPNRYHFRQCAKHTVPLVKQ
RQADMERKRQNPEYKYEEPNNFLTWQIRDAMRRDDPSEQTPEMISHRVLVVNFAAIHTSTFTVTN
TLIDLFSAAPEKGYLEGIREEAQRVLNEYDGVWTKAGLAKMIRVDSAIRESMRLSGFSSRGLVRKVIS

DGGVTLEDGLHLNKGTKVGLSAYSVHHDETIFEDPMTYDAFRFSRPREAFATSQDIPIGDLIGDEKP
LANGTASGTEKGENLTEVLKHKNLSMASTSETFLAFGHGRHACPGRFFAANELKLLIAYMVLNYD
VKPLPVRPRNTWLGDTVLPPMKATVQVRRRKTEVSG*
>CYP676A7|122042|Acastr1
MASILPWDALKLLGYLPFVLFIVLLYVVVIAVQRRYFSPISDIPGPFLASVSIFWQIWQIAKGATAKSTI
KLHEKHGPFVRISHKEVSINHPDAIRAVLLAPLRKGEFYGIMALPDHHHPTPMSERDPKRCVQRKK
NVASGYALSNIMTSEKFIDDTIGLLEERLDQLSQANEPVAFDKWFNFMAFDIIGEVTFSRRFGFLEKG
IDIGGAIANTRILTAYYVSVMAHLQWLHRLTLGPIISYLGLEPNQHIFDTAKAAVAARQANEFARTD
MIEQWKISRRNHPDRMAEQEILDVATATIGAGADTISTTLQAFFYFLLKHPEYLARLQGEIDAANAR
GELSHVVSYGEAQKLPFLQACIKETFRCHPAVPTGLARIVPEEGLVVAGHRFERGITVSVNSWAIHR
NPLCFGKTAAHYDPTRWLDPEQASRMDKYMIQFGAGYNSCPGRNLAYLEINKVAATLVRDFDVR
QVEPGREWRYEEWFTAVPYGWDCWVRRRVEKGVVTQYGALQVGRGLGR*
>CYP65FH1|122045|Acastr1
MASAIDTNKLISLTPRTLVISSVILQAIYIVYLIGKAVYNVYFHPLSAFPPGPKSFAATRVSHVRSLLRG
QLSQTVKELHDEYGEVVRIGPDELSFTAAQAWRDIYGHRQGHKSFQKDPVFYGLPPEGVHNIVSTP
SDADHSRMRRLLAHAFSEKALREQEPLIMSYVDTLVDKLRQHIHGPEGGKMDLVRWYNFTTFDIIG
DLAFGESFHCLKNKEYHSWVSTIFKSIKFLAYLQVGRRFPPLTEAINFVLPRKLREQRRKRMQYNKD
RVDKRLELGTDVDRPDFISYILRHNDEKGMSRSEIQACSAVLTTAPDFISYILRHNDEKGMSRSEIQA
CSAVLTTAGSETTATLLSGVTFHLLSNPDAYKKLVDEVRTTFKDEEEINSIAVGQLPYLNAVLEEGFR
MYPPTPAAIPRVTPPEGDEICGQWIPGNTIVGINHYAAYRSPQNFALPDIFIPERWLPSTASVSSTAHP
NIDFSSDSKHVLQPFSFGPRNCIGRNLAYMEIRIIMARMLWTFDLELCEESKGWERQKVWIIWEKKP
LVVRLREVVRA*
>CYP6001J2|122110|Acastr1
MFGHTKALFFSGQEQEDTSNYGDDSFTEAKVKQSGLLNDLRALGPNIGQDALTLLEKVASKGEPY
DDRTFLMERIIALTASLPQSSKMRAKLSQTLVGTLWDSLQHPPLSYYGDLHQYRTADGSYNNIMFP
DFGKAGMPYAKSVRSVKALHGSKPDPGLLFDLLMRRRDGKFAKNPAGINSMLFYHATIIIHDIFRT
NRRDSNISDTSSYLDLSPLYGKDQEAQNTVRTFKDGKLKPDTFAEERLIGQPPGVCVMLVMYSRFH
NYVAENLAAINENNRFTEPRKGDPDYDIKVQKRDNDLFQTARLVVGGMYINISLHDYIRGITNIHH
SNSTWTLDPRVEVTASEGGPAVERGVGNMVSAEFNLLYRFHSSISQRDDKWTADFFKSIFGDKEPEK
IGLEEFYQGVAKYERGIPKEPSERVFGGLTRDPKTGKFNDGDLVQILKESIEDPAGIFPNIYEPQTASL
NEFRQFFGLKRHDTFQSINPDPEIADLLRKLYDSPDMVELYPGLFVEDGKPRMDPGSGFAAPYTVGR
AVLSDAVTLVRGDRFYTLDYTSATMTTWGMAEVQQNYETLGGSMLYRLIHRSFPKWFKFNSIYVM
QPMYLPSMNQEIAKQFGTIDQYCLDPPAQPPKVTILSSHAAISSLLNDQKNFKVKYGLQLPDLVFPG
YMLQGDGAANRDNHKFVAQRMLNGQGGLDLYARSFEGAMRKVLAREAYKLGDCYQVDLSKDV
ARPVTVQTHADFLNLSLRVKGKDDEGLSEEELYKYTVSYLNYANIDSDPAESWNLRRKARQSYMK
LKTSTEDMVKKSAPKGSLVGHFFPAAPAATGSLREVGQNLTRDLLDAGYSVEKTATTLFTTAAGGI
ANIPTTFVQILDWFLQEENAQHWAAVQKLAAKDTPASFETLKKYVLEACRFSSGLALVRICIPEQGE
TASVKNDQGESIMLKKGEVVLCNVTAAFRDPAVYPDPNEVKLDRPMDLYQMWSMGPHGCAGRA
IAITALTSMTKVCAQIRNLRRAPGAQGYIKSVPRPLGGRNYLSDDWSRYQPFAGVMVSLRTLNTAT
KATLLLLSLTGFWTTWGIGKRTGFLALIADRLKANVKLLPAAEKPLKTTFTGIPPVDQLLRRLTVFL
WPAIDGTWPGLSLVAFEFSGQFSGSWMVAGLEGLRVGNRRNILSFTTIVGVLSQVITYATILPLYLFI
HLLTSPTNLDPEQKSPGDPTTDLLVDPSEAAAWTIGFTLSYLIPTILVALPSPTYTSFQTHQTFMAFWE
LYPIPFKIFQILLVRWFSSNLLFDKGSKSPKERKASALRALRYVYLFSAAVGVITHIVTLTLSFSSIFFPTL
FNPGIRAGLNPSAIFLGVSPLSTTPVKDMGEGLWHFLVWNMNVSGLAPIIWAALQYRNAHEGRAN
FDGWGVLILKIVAGTMVGGSASSVTALMWARDVMVLGQTERSKLK*

>CYP5189D1|122254|Acastr1
MATMSDIWVGLPWSVQLIATCFVLTATLLITTRLNSLSHSWSAAAGKGHEPPIAPYWLPGIQHLAA
FLLNPARTFRTTQSKYKDSPYTLLMGNVKFHVFGSPSAATHVFARSRTFAYEPVTMSMLENGLDLP
VADRVYFQISLDRANSAEHAKGFVLQNHNVWLRYLSGEPLDDLMQLFTRHFHQVLEQHLDMKTR
GWQTVDLYQFLRKVIFNTSVLTFFGPHLAQIWGPTMWKDFCLFNDATYIGVRTNLAYVLQPRAGR

ARERMLRAFEQWLTHDSEGDWPDKDKYWNEAWGAKMNWERDHLARQFGLSLRGRATMQASF
LFAIVLNAAPMGSWFTWVAASNSDRLAKCRAESSKFLRPSKSSRFDHELDIDATQLRGSNFMQAL
WKEALRIGTAASPARVVMEDTELEGYVVRKGSVILMPTALLHHSDLFPRADQVDTERWTGEGAIV
QQRSKSIRTFGGGMGMCSGRHAAEQEVIGLVSKLITLFDIEFEDSWKDKFHFDPRSLGIMHPAGPPM
VRLKRRVL*
>CYP65EP1|122262|Acastr1
MLSSNRTQYALALWLTPLVLYVLCAWLKVITGAFFGPLSKIPGPKLWAISRLPRMYMEWQGEEAA
GVAALHKKYGPVVRLAPNEVSFAGGAQAWKDIYGYRKNVGQEKHPYRSPASYWTPANGAPSMI
QAIRDEDHQRLRKSLAVSFSEKSMKQMEPRVKGWARLLQTKLAERGDAAVDMVELFRCATFDAM
GDLALGDDLQMLRNGRLSDYVHAAFSGIRTIIRLRVLSTYNSFTRWLVQDCFFQSAFVRKAAMENL
RYSADRVDRRLQEPVRDPPDLWTKPLTGIDGKMSLDEYHSTAQLLMTAGTDTSAIGLSGTLYYLLA
NPDCLAKVTDEVRSAFSSMDDVTYETLAQQKYLDAALQEGLRLYPPVPTGAPRVTPMPGWSICGY
WIPGGVNVHAPHYATSRLPAHFTAPESFHPERWLGDEKFQNDRLDAVQPFSHGPQNCLGQAFAM
NEMRLFMAAVLLQFDLQLCEDPQAWLDQRVFSMWETKPLMCVATKTS*
>CYP698C1|122720|Acastr1
MRVNLSIMAMKRPYKGYAFQIPTLSRWEIFICDEAMITEYRNLDSNLMSANAVTAELFEAKYALPG
AAEGIHKIQIPILAKGITWLRMRASTKSDYYFSDFYDEFLAAFAEEVPVKDESWTTVPCFQAATRMV
SRLTAKALLGRPLCRDPDLIALFCNYGNAVPTSAFFIAKFPAPLKPLVALLCSAPKMSRRIQRIVLQIA
TQRRANRPKTPNDLTDWMMNWVDQNGSSKYNDKDVALQIANIVFGAIHTTSQLLVHSLYEVATR
PEYRTPLSQEVIECVAEHGGWTKDALEAMRKLDSFIRESQRWNPLDAGSMARRVCKDFTFSTGLHI
PAGNWIFAANSPALRDPENYSNPEKFDGFRYSRLREQEGQSANHTLVTTSLKNYDPGRYMAADEI
KLMLAHMIVNYDIAMEDHGPRPPNLILGKILFPNLNAKIMLRKIRV*
>CYP5388B1|122727|Acastr1
MEPVFVFSFVAAIALYLVSLFLIPGKKQPRPSEIPYFDWEGGSTTKSNERFFADARGILKEAYQKFGN
NVYKLKTPNGDHVFLSSSAAEALKSMSKKAFNIQEASNEFLAAKYLTIDRPPEWSHDAVRMDLTQS
LDWTALAVHPKMIRIIALLTGRVFVGPQLNRNEEWIETTINFTTDVFNGSQMIKKWHPALRPIAQYF
IPEIQRIHAHHAKIHQLLLPTLKARAEAEAKPGYEKPDDMLQWLKDRAAKNNVRDFKVQASTQLV
ISLAAIHTTAMAATHAVYDLAAYPKYIEPLREEVVRVLQEEDGIFTKRATSKMEKMDSFLKESQRLN
PFSMTNFVRKVVKPISLPNGTPLPVGTIFSVPNSVLDREEGIWSNPDEFDGFRYYILRRSSAEESMKHL
LSECSADSMSFGYGAHTCSGRFFASDTLKLILGHLVKNFDLELKDKKKDRPANMESAFNILPDPTVE
IMFKRIG*
>CYP59AA1|122808|Acastr1
MEPLLDTLQIALAVVTGLVTVFVIKLYRVRSFIQQHQKQGLPMPPHHWLLGHIPLIVSTIRSMPPSA
HGLYVGDQIRQQYPHLDSAFYLDTWPFATPILVVLKPDMMYQLTQANQIPKDKGLRRFLEPLTGKE
DLVTLEGSAWKRWRAIFNPGFSNNHITSLIPGMIEEVEVFKIILNEHARNGDILYLEAATLNLTIDIIG
RIVMDHRFQSQTRYNDMTAALRKQLEWCTTGMNIDPLEYVNIFRPFVHAYNKHRMNRYLSRELDS
RYSIIHGKADNKNKSSSSFSIFAGHDTTSAGAIFTYHLLAQHPDILSKVRAEHDTVFGSNAADTASVL
CSKPQLLNRLPYTLAVVKETLRMYPTVAALRDGQPDSTCLATTASAFRRITPEEYLPQRWLVPEGDP
LYPPKNAWRPFERGPRNCIGQEVALTEIKVMLVLTVRHDDLNNGVAPTRKPHLGRFGAEYHSCAT
QNITTPWRAPCALASGPATFRYVFVFAFLFPTPFIVNYAIIWVMFRHTHLMEKVGKIPPTLPHLVPLL
GSTISFVWDGANLVNFKEHLTPPVLEALYGQALLSANPTFVRDIWAYDAGVQDLARRLPRFWVPA
AYRLRDKVLASVKRWHALARSSIHASMIYADGDGEPYWGSELDATLLSRLRNSWRGTVEQTPGLG
LDMKMVEKDPLLILFYAETLRQLLAIHITRCAPDHDIKVNNWLLPRNKVM*
>CYP5077C4|123121|Acastr1
MSLPDHAPGTPVYDQKLMLLISLAALSLCLVTLGRTYARLVHVPGPLLAAFSNIPRFLWVWTGRAH
EIHIALHARYGHLVRLGPNMVSVGDPAEISQIYGIGSNFVKVMNPKRLSEFYPVLQPYSKGKLMPGL
FNTTDDELHRVMKRPVAGIYSMSNLVYFEPYVDSTMKVFFQELDKQLVRTGNVCDLGTWLQWFA
FDVMGEITFSRRLGFLERAEDVDGIMGSIWKKFGYSALVGQMTWLDVLWEKNPIRERLWPSTTSPV
VAFAVARANERLTLSASSEQEKSNLNSRDFLSRFIDAKSQDSKIPDWFLTAWTTSNVLAGSDTTAILL
RAILYFLLKHPETLHRLQLELEEAANGGRLSNPVTWKESRSLPYLDACVKEAGRLHPAVGLPLERV

VPAKGAEICGKRFEAGTIVGINAWVVHRDKDAFGEDAAAWRPERWLCAEQHRHQMERALLTFG
AGHRTCIGKNISYLEIYKLIPTILQTYEASRPRQ*
>CYP573A26|123219|Acastr1
MASLDSSSLSSLLSKVTLVYLLIGGLVYLTACVLYQIIHYRFFHPLSQFPGPFWASVTRLWIAYHSAKK
DEIAVMEALHQKYGPAVRVSPSLIIVNDPKRLPDIYHRQANKSNFYINGLFGKTENVFNMRDWKQ
HAHFRKFIAGPFWITENLEVREIVNLDAYIYGWQYSFTHVKKMEPLVDTRVYDWLDKIDERFAKTG
KPIEFSRWAVDFQSFMAYDVISEIAFGAPFGFVETGTDVGSLIQGFHDGLPFFAIMGRLHPFTTWIKE
TWVGENFFMAKPEHQSGMGALMRFRDKLLAQRLKDIEAGTAGGRVDLLQNFLEARQEDGKPLD
MEYIKAEISLILTAGADTTGTTFQAIVHYLMLDPAAYSKVMAEIDEATRAGHLSAMPQFNEVLRHC
PYYVACVKETLRLCPPAEGMLPRTVSKGGIMLEGKFAPEGTEISCVPRIVQRNRELFGKDANDFRPE
RWLESEENANEYSKYSMAFGYGARGCLGKDIALMELYKAPVQASSSTSLMQYRRVPSFITFVLMSG
LVPSHLPPPTCGREEARQSCEFRWFSLLEGCLAADRKAYPGRLERHVKNQTGVY*
>CYP532U1|123221|Acastr1
MHEKYGDVVRYGPNLVSFANPQAINDIYGIGKPLSKSPYYLPSQAMSKGRAFASLFTLLENKAHSD
LKRTLATAFSGSSIVSLEPLVDPVIYAWVEQTKKVYVEGNRVCDIGWWMQLFAFDVVTNLTYSKTH
GMVEQHKDVDGIVAWLGWMFSYTSVVGQMPILDQFLNKNPIQRFLEWAEISVPTFPTVIFAKKRIV
ERLSTDPSVLPTKQGMRPDILTQLMESGKKHPNVLTMKMITSQAVSIAFAGSDNTAVTLTAIIYYLL
KNRRCYERLMAELDDAAANGRIPANPGEILAWEQSQGLTYFNAVVKESQRLYPSVGGLLERMTPP
QGAVILGKWYPGNTLVGCNAWVIHQRKERKEVFGDDCAEFKPERWIEGDPDHIKMMNQSFFAFG
AGTRACIGRSISLLEIGKAVPVWLRLFDIKLVDPEKEWTVHNSFVVKQHGVNATFSMRKYSEKETLT
MS*
>CYP680C2|123308|Acastr1
MPISVVALAVQYKWQLVAAVILAYCATLCVYRRFFHPLAKIPGPFLPAVTTLYQSAYNKQFYKQVE
RLHERYGPVVRITPDEIHLSDPENYDKIYYQASKYGKSPNFYNALCVPASTFGSLPNTVHRIRRGAIN
PMFSRKMVQQLESVVQDKAGKVCKLMQEGIRKRLPVDLHHAFRAVSMDVISDFAFNQSYNFLERE
DLGAYFFRMARGIGPALWVFQQLPSFQAAALKIPPWLAPYLSEPLGAVTSLQQRCVKHVEDVKRD
MAVGKDSERPTIFSTLLTDTDKPDGYRVPSTMELKDDAYSILAAASDTTGNAMTVATYHVLSNPQI
YQTLVQELKNAFPDPNAELPYAELEKLPYLTGVIKEALRLSFGVIGRLPRVVPESGATFNGYYVPSGT
IVGMSSWIMHRNPNIFPDPMKFQPERWLDPENYKRLDRYMVPFGRGNRMCVGMPLAYCELYVVL
GTFSRRFHDLKVYETTPADMEYDDFFSSFHVNGNKVFKAASSMIACG*
>CYP6001A24|123335|Acastr1
MSESDAVMGSLPNRIEGAFTGIKELIHAALQPLPTQTGDGSYIEEKVPTHLFKDLHDMGFDGIDALI
DTLKADVSGDPTDDRTYLMENLASRLQPTSKRGTNLTNTFVNKLWKDLLHPPVTSLGEQYRYRQA
DGSHNNIMFPRLGAANTPYARTVRPQTLQPANLPEPEVLFDSIMVRKKFEEHPNKISSVLFYLASIII
HDLFRTDHNDFNNSMTSSYLDLAPLYGSNQDEQNMMRTGQDGKIKADCFSEKRLLGFPPGVGVL
LIMFNRFHNHVVEELARINESGRFTKPKEGLPASAAKNAVDPYAKYDNDLFQTGRLITCGLYINCIL
RDYVRTILNLNRTSSTWDLDPRVQQGKNIFVEGAPEGVGNQVSAEFNLVYRWHAAISQRDDEWTQ
QAYRNMFPGKKPSEVSLPELMAGLKKWEKSIPADPQERPFADLERKNGSLNDDGLVKIITESVDDV
AGAFGANKVPEVLRAVEILGIKQARSWNLATLNEFRGYFNLKKHEKFTDINPDPQVAEQLKHLYD
HPDFVELYPGLVVEEAKRPEKPGSGLCPSYTVSRAVLSDAVALVRGDRFYTLDYTPKNLTNWGYNE
VNYNVGVDQGHVFYKLFLRAFPKHFKPNSIYAHFPLVIPPENEKILKQLGIADRYSWDKPSPIPPLTF
ITSYAAAKSILDNQIDFKVTWGEAIKFLMNHNGKPYGADFMLAGDNEVNTNSRQMMKTALYREK
WHQEVKLFYENITLKLLREKSYKLAGVNQVDIVRDVGNLAQAHFCANIFSLPLKTKENPFGIYTESQ
LYGIMALVFTCIFFDAEPANSFPLRQAARQLTQQLGQLTMANVETVKVTGFLASLAERLHHHTALS
DYGVHMIRRLLDSGLDAKDIVWSHILPTLDAKDIVWSHILPTAGGMVANQAQLFAQTLDYYLSEE
GKVHLPDIRKLAEADTPEADERILRYFMEGSRMKATVGLYRDAVTSTTIRDGDRDIHVKPGQRLMI
TASMDKNAFPEPEKINLDRDMDSYIHYGMGPHICLGYGLSKVAMTAMLKTVARLDNLRRAPGVQ
GQMKKIPGPGGFTLYMLADQSSYFPFPTTMKVQWDGDLPAPK*
>CYP548A32|123348|Acastr1
MMSFITTERLAVTVLYATALGCLLYVLGVIIYRLTLHPLARYPGPLLAKITDWYNVYHAWKGDRHL
ELWRIHQVYGDVVRFGPNSVSINTRTALRDIYGFKANVKKSDFYSVFPATKAAFSTHTAIDKAVHA

RKRRVLSQAFSDSAMRAMEVHILDNIQAWCGHLGDDQARTRDEVSRPMNGFGGPNPASDSKGW
SAPKNMGDWTNYLSFDVLGDLCFGKPFGVMEREENRFVLDLIPTAAYFHNVNGQMPIIKKLGLDA
LLFGQIKKKRERYMAYSKQQMAERAKLGINGQRRDFFHHLLTAKDPETGKGFGPQELWGESNVLL
IAGNDRYSNSQSPILIPGKMPAATLIAPAPGSDTTSTALSSTFFSLTHNPHTLHTLTSHIRRTFPTLASIR
SGPLLTQSTYLRACLDESLRLSPPVGGLLPREVLPGGIVIDGHHFPAGTVVGVPHYALHHKEEYFPD
PWAFKPGRWIVTGDGGEEGGEGTAESVALAREAFCPFSVGPRGCVGKGMAYLEYLELSVALARVV
WLFDFRLATGGSGDVGLGEGRPDAEWGRHRTGEYQLFDAFVSMKDGPLVEFRARVD*
>CYP51199A1|123476|Acastr1
MLVSFAAFITLIMLLEEHGTLRGLWVGATTMPWWALCLVFIFVCCLTRVVTGFTPVRSNEVSSSMV
ARRVAVLPYWIPYLGHVPFLFSLSSSLRAARDQVKAGVFALRLGPSKHYIIHTPSLTKAIFHSPPDTVI
KDPILWRVLENMFGARRQSKETYVPLFKALHKSSHEWLLRRTALAKIVDVTARGIERNVAQLITRA
FSPIDAQPWERAAVPTLVRDSKVTAAEVSLFTLIRAFVGIIATPSIFGNSLIEQEGLFEDIWTLDSGFRF
LVMGLPRWLPIRSLSRAYAARDRLLKHLTRYSIALDQYARGDEPSGEWGDMSDVGELIQSRMKVM
REAGISPQSRASFDLAYLWAMNANANNLVFWIILRICATPGLLERVRAEIAPYAIVVTSPQGTRFLEP
KSLRLDAEGISRSCPLLKACYLEALRLHSASVTIKRVQKDVVVTESATDNPDGQARSYVLDPGSYVEI
CHQLHQSDSRYFERPEQFLPERFLISGKDGLKVKDHTLTPYGGGSSICPGRLYAATETVIFAAAILTC
WDIEPLGSKGWQIPANEAVTGVSRPAKDIRVTMSNGRRI*
>CYP6456D1|124313|Acastr1
MEERSYTATLVLLVAFAAVCYLLDYFYAPKNAPNEPPVLPHPIPYVGHIIGLLRHGLTYFEATSARSK
SPIHTLHVLNSKIYVVTSPDLVNAVSRNSSSIAFNPFIARLAQRLTGADEATMAIVGDNLNGEKGHW
GLVLQTHDDTIAAMAPGESLDHMNRTMLQQATEHLQALEQDADGTVINLYAWIKHVLTVCSTR
AVYGPTNPFTLEPGLEEVFWDFDRETDLLLVNIFPSIMAPKGYRAREKLVHAFKHYFDNNPRGRSS
GLTNARYNANTKYGVTSVNMGRLEAGALIGILVNTVPSLFYMLVHIYSDEALLHALRDELQACVST
DLGKTTTSPPTRHLNVSAVKENCPLFHSTFHEVLRLHSLGATSRLVLKDTLLNDTYLLKAGSVVQM
PTSVIHSDPTVWGPTPKSFNPHRFLKQDPAGKEPKRTPAAAFRPWGGGSTLCPGRHFASTEIMSLA
AMLVLRFDMVPLQGRWVVPPPYQYGYGGDMVPLQGRWVVPPPYQVSMATAVFPPKTDISVRVVR
RREVGDGAWDFVIG*
>CYP504A49|124467|Acastr1
MGYQSVGIAVVAVLFFAIRYLNRTDVPKIKNLPEVPGVPIFGNLLQLGNEHARKAGEWAKTYGPVF
QVRMGNKRIIFANTFDSVKHLWINNQSALISRPTLHTFHTVVSSSQGYTIGTSPWDESCKLRRKAAA
TALNRPAVQSYMPIIDLESNISIKELLADSKDGQIDIDPNPYFQRFALNTSLTLNYGIRIDGSIDNELLK
EVVHVERVVSNLRRENGERGGQAVYNWEHPKRPRPGEAKLSEAEIKSICLTMVSAGLDTVPGNLI
MGIAYLSSPHGQEVQKRAYEEIMKVYPNGDAWEKCLTEEKVPYVTALVKELLRFWTVIPICLPRVSI
KDIQYKNATIPAGSTFYMNAWAADYDATHFKDPNSFLPERYLDNAEGTGTPHYGYGAGSRMCVG
SHLANRELYTAFVRLISAFHILPPKDKRDEPILDALECNAIKTSLTTEPKKFKVGFRARDPETLREWIR
GSDERTRDL*

>CYP534P1|124707|Acastr1
MWKRLYAPFKKLGTDTFLTVSPGGNMLWCAEPNVISQITTRRNDFPKPIEIYGSLNLYGKNVISTEG
AAQCMVQSWLGPDGAGDKTVDRVSDDTMRLSLHIISRAGFGVRLLWPGVEDRNMDNTEAEPTG
GGEQSSSDIPPGHTMRYTDALSSFLHNILLVLLVPRWILITLPYKKTQKAYESYLEWGKYMREIYAAK
KAEVLAGEEREGMDLMGALVKGAGITPETLAASGPFEKSQPAPKQTFTDEDVLGNAFVFIVAGHET
TANSIHFSLLYLAMNPASQRRLQKDLDSNFNGRPISEWDYDRDLPHLFGGMAGAVLNEQLRLVPP
VVSIPKKVSETSPQTFSVDGKKAHLPAGTGISLLAVAVHRNPKYWPHGPPSDPSHPAHPESSLDNDL
EEFKPERWLLDSSPTTTTNPPTSNKNNSNGHANGHHHLDKAEADDLGVNTAADTAADLFHPAR
ARRLHPLQRRLPRLSGPPLRAGRGPGRPGRHLHPLQRRAGRRRFRHGRRGKRDG*
>CYP5078J1|124755|Acastr1
MTNFVHWLQNLPWKPLLWLLSLALLNLLYKRYWTSLRRVPGPFLASFSNLWKFNAAWHQDMPR
RNAAVHEKYGPVVRIGHNTVSVSDPSALSIIYSFQAWDKSAFYPIVEALYKGKPVPNIFNGKLECDSS
TDLDAWTRFTTRNTDYHQRLKRASAHAYSMTSLKDLETYLDKCIQLFLTRIAEVTELGTKPLDTSM
WLQYFAFDALGEVNFSQQLGFLKTGTDVASNIAAIDGLLQYLSLIGQMAWLHKFLLGNPLMHRLI

PQLESSNEIQNFALKMIKQRQEHPTDAHRDILARFLEVHDKDPSKFTFTEILGLTTTNLIAGSGTTSV
ALRAILYYLCRNPNAYAKLRREVLDAEAGGRISKPVSYAESLQLEYLSAVINETLRVHPSTGFILERIV
PRGGATISGVYLPEGTTVGVNTWVLHHNKDIFGNDADVYRPERWIDSSPEQLREMKRYMIAFGVG
PRACIGKNIAMMEMCKLIPELIRAYDWELAHPEREWKVLGHWFTKQTDLDMVFVKRGPKEA*
>CYP65FP1|124783|Acastr1
METVGDLYALDKQRPSDWFSISAATLATLFFVRYIGSVIYEAFLSPLSVYPGPFWCRISNLPAAWQL
WHGTHAYWINRLHKKYGEVVRITPTELSYTSAQAWKDIYGHAHHGKGTNGKEPRFYGPVKDLDN
KTPGILESDDANHTRMRKIFSHAFSHKAIQEQEPIFQKYTSSLIKKLKMAIAEDSERQFDILAWYNYT
TFDVMADLTFGESLNLLETNTYAPWISAIFNSLQAGVIFRSMRYWPPFYRILRFIFGKTLREKRRVQF
KYCADSVDRRLEKDPEHTHPDLWSLVLRQKEEERLTLAEMHTNSSAFMMAGTETTATLCAGLTYY
LLKHPEKMDKLVKEIRTSFNTEDEMTITRMQRLEYLQACLDETLRVYPPSVTGFQRRTPPEGNEICG
RFVPGETTVYVSNYACFRNENNFRDPDEFIPERFLATGGYTTDNRSALQPFSYGPRNCMGRHMAYV
EMRMIMVKMLWNFDYILCDKNDNWLDQKVYLVWKKEPLMVQLKPRKDVKELL*
>CYP65FM1|124788|Acastr1
MSTVDVPAKGILLGLGPSLTAILAIALFIYVSIIIYDVFFGSMSQFPGPFWHKISGLPLAYIQALGGEAE
ALPALHEKYGPIVQIAPKELSVVSGAQAWNDIYGFKKPGQPKPFKDPVLYMTNVIEAESINLASDA
NHSRQRRVLAHAFSDAALREQQPLLKSWAEKMKAKMEERVQVGGKAKVDMVKLYHYTTFDIMG
DLTFGEGLDMLEEGQYAPWVQTLIQGIKLDAWMRIAKHFTIGHFLIKTLLNTKIARAKHWEHFNY
AKERVDKRLARTPKRPDFWSRILAKGEGHGGMSMGEHYVTASLFMIAGTETTATALCGVTYLLTQ
HTEAMKRVKEEVRSNIFSFEDMTLEKLAGLKYTNACLQEAMRMYPPLPCSLLRRTPEGGTTVCGQ
WIPGDVSVGVHVFATHISPLNFKNPRAFHPERWLGDPEYADDHFGAMEAFSVGPRNCLGKNLAW
HEARLLLATLLLHFDVALAEESQGWIHQRLYTLWEKPPLMVNLTPAVKA*
>CYP617G20|124868|Acastr1
MNSPVKTFALTSTLGSIFLIRQVPRYAIANSYIGTASVFFLVQFLVFAIWQVILYPRFFSPLRHLPQPKF
ASFINGQMPVIAAEPTGAPMQRWVNEIPNEGFINGQMPVIAAEPTGAPMQRWVNEIPNEGLIRYT
HLFNADRLLITSPKALGEVLVQKNYDFVKPAMVRNGLGRLLGIGVLLAEGEEHKTQRKNLMPAFA
YRHIKDLYPIFWSKARELVQAIASSVQETAPVSEGEKSSSSLVTEVGEWLGCATLDTIGVAGMGQDF
NSIQNPNTELNLTYRKVFKPSRQAQILGFLGLFFPIWLIRSLPIKRNEDIMDAAKLIRKTCRQLIDDKR
EKLRNTEKEADIDIISVALESGGFSNDNLVDQMMTFVAAGHETTATASMWMIHLLCKHKDVQTRL
RNEIRNNLPTIDDPTTTVTADMLDRLPYLHAVCNEVLRIYPPVPLTLRIAAKDTSIVGQFVPKGTTIIL
APWAINMSKDLWGPDATDFNPDRWMGPGRANTGGAASNYSFMTFLHGPRSCIGMSFAKAEFAC
LMAGMVGKFEMELVSDKPVEIQAGVTAKPKGGVNVRLRVLEGW

>CYP527X1|125258|Acastr1
MALFSLPSSSRGLPCVETEEPGSRSVTGSVARIGPNELLTSSPELLIRMNAARSPYTKSDWYKGVRIQP
GRENVFSGRDEKHHNLRRSQMAAGYSGKENPSLEKSIDTQISNLIQLIRTKYISTNSSLRPLDLARKV
QYFTLDTITDIAFGQAFGDLVRDEDVYHYIQSTEETLEVLMFLSVFPKLQDLLSNEWIAKLCFPSDKD
STGIGRLIAIAKKLVAERFGPEAITYPDMLDSFIRHGLKEEELVTESLLQILAGFDTSATVIRATMLFLM
THPVTYRTLQNEIDAAINAGKVSSPVIETGQAQKLPYLQAVIREGMRIWPAVTGLLSKVTPPEGDTV
ELDGKMVFLPGNTKIGYCAWGVHRNKDVFGEDAHVFRPERWLVEQSEERLGRMQRTAELIWGYG
KYQCLGKNVAMIELNKLFRNFDFSLVDPTTPWQSENIGLWKQSQLWVRVTERSRTS*
>CYP671L1|125397|Acastr1
MADASMLSLEGMQNVLGANLPSTVTAALCLGALFHLSIRKVEVDYLIWHYLALSIGVYSALVYAYL
SLTNCSLLEASAKALLVGVSFNTGLTLSIGIYRLFFHRLRKFPGPFGAKLSRFYTVKLAAKSVQYNLEV
AKMHEKYGDFIRTGGAHPMSFEVPSFLLSQFFRQVHESSAFQVSKDHKKCSIHMTRDFEDHRRRRR
AWDRSFSIKGSKGSVKSLATYEPRIKSKVDLFLSQLGAPGRTSVNITDWAMFLSFDVMGEVGFSKDF
NNLATGKEHPAIKGIHDHMLILGILSNTWTPDQNPQDIVSWLMKAYKEKDMSAAPSQAALDEDA
RVVIIAGSETTSTTLATALYYLAKHPATYKTLQDLVDRALPGGERDWSYERVREISFLDGIINETMRL
KPPLMTGYYRVTPPHGIQVDEVHIPGDVNVFVPVQVIQRDPRYWPKPLEFIPERWSDQKGQLSIEN
APFIPFSLGVYSCPGKNLALMTVRSALARIAQQYDVAFAPGETGEDFDLKAQDTFTTTLAPLQLQFT
PRYRG*

>CYP682BE1|125569|Acastr1
MPLQYLTRLSVAIPLGTVFIYLAGLAIYRLYFSPIAKFPGPKLAALSRWYEFYYEVVLKGQFTFHIQEL
HKKYGMCIPFRAVNARRLIIAAGPIVRITPDELHVEDSDYWDELYSRTARYDKYEWMAGRFGNSTS
TFTTAKHDLHALRRGALNPFFSNKPSTIREKVELLCKNIAKCKSTGKEFRLEIAFSAFAGDVVTQYAL
GMCYNHLNSPGFTESFHEAFMAVSEFGHVALQFPWVHPFLNALPDAISEMMNPPLHMLLVLQRD
LKIKLAQIMKGELKTDKQHPSIFQEILQSDLPPQEQSVQRLGDEAQLILGAGLETTGWALSTAAFHII
NDPAIFKKLRDELKQAMPDPTAQLDWLQLERLPYLSGCIQEGIRLSYGVSARNPRISPSKPTKYKDW
VIPAGTPVSMTIVDVHHDEEIYPKSRSFIPERWLDNPRTANGSPLNRYFVAFGKGARSCLGINLAYA
ELYLALAAVFRRFSFELYDTDISDIELAHDCFLPSPKFDSKGVRVTVKSIED*
>CYP682P2|125690|Acastr1
MAWLQSWLSFLLRSFTFHNGPLAWVGLAIGYALVLAIYRLYFSPLSKFPGPRVASLTWGYQFYHDIV
RRGQYIWVVQSMHAKYGPIVRINPSELHVCDSSFYDTLYAAGGPQRKREKWAWDNVGAAGVEDS
KWAWDNVGAAGVEDSTLSTIDHDRHRMRRSAIAPFFSTQNVRKLQPVIQAKVNTLFRRLRDLKAT
GNPVNLSHAFSALTNGKIDVIMEYCFARSSSRLEAPDFDPTYKNASHRGVRMAQIAKHVRPLTWIA
KALLYLPESWAEKLGPAFRMFLVERRVSALIQSTMVPRFCPCFAHVTVFRELLDSKLPSQEKEPSRLA
ADAQVLVSAGSETTARALTFATYSLLTSPVVLQTLKLELESAIPGSDPAAKITLEQIQHLPYLSAVIKE
SLRLAYGVVGRLARIWPREEIQFGEWSIPAGTPVSMTSYDVHHDEEIFKDSFRFNPQRWIDNPGLDR
YLVSFGKGTRQCLGMNLAYAEMYLTLARLFRWFGCGGVRKSDDVGVLELFDTDETGVRKSDDVG
VLELFDTDETDVKMVAEVLVPFVKRGSKGVRAVILP*
>CYP5148B20|125970|Acastr1
MSLRRRAGRLKRVPPPAAPRDAKLADSNDDDDDSGDDDNVEVYMLAESQRFYSYRKECPPFVRT
MARRNGLREDRKAVVLADDDGCRPDESDCQRSELVSHVYAPKAGFWTLELGGVRMIVKAKAHG
NGAAYVIWWGPEEGFGEKAVAFSPRAPRGEVRGWSDGETEQPDHDSHHEPSDEENEFRSVDATQ
PSSMRRNSSRNAKDKGQAKIKRLIAGLTGTDRGLTTKRPSQQLRVLLEQHGQAEPVTNKAARANR
PSLPTPRRRLLPSPSTSDIPLAQVRMKRERSVSEGAARQPPKLPVQTSSHNPPHLPSKNDNGSVPFYL
HSCMTIETLFASALSVWRLRGKDEDVAALTVRFDWLKKAEAMIIMREVADSFQKMLETIAEAPVW
LKTDETRKSCDVRVGSYNSYVTLQDLVASGLVAPLVTLGVIATTGLVMLYNQLFGRVLVDKHGNKI
PDGPWGFPIVGSFPFLRRYPELALDHWAKKYGDLYSMWLGNQLFVIVSDPGIAKDLMVTNGAVFS
SRKELYIKSQTVFVGRGITATPYNDRWRRHRRIAVGWLNQRVVDSYTSILDREATVLVMALHNASK
GGTVPVNPQSHVGRCSLNNMTTITFGLRTDSIHHPLVGRALKLSREFMNCTGPMSNLVDFVPVLQL
FPNRMRIRGRKLHEALLETYGGLIKEIELEFKSGRDVDDCLVKSMLQARKEENLDDLDMVILASAF
MIGGVETTAAIMQWFSALIPAHPDIQKKAQDELDRVVGRDRLPTIEDEKDLPYCHAIVKEVERCHN
PFWLGTPHVATEDFTYKGNFIPKDTVVVLNTWTMHHDPKHYQDPDTFNPDRYINDHLTSAESAN
LANPHARDHWMFGAGRRICPGMIVSEREIWLTVSRMLWAFNMMELPDEPIDLKEYDGLSGRSPVP
FRIMVSPRHAGVERVWKGEAGVGDC*
>CYP5089B1|126315|Acastr1
MPSLFTAVQCAFLAVLARCIYRRYFHPLSRYPGPFLASFTNIWKFYAFFRGDHHLIEQNLHDKYGHV
IRTGPDSLAFSSLSAFEAIYGFNRSIEKGDFYDFGRDANTQAGSIFSARTDAAHREHRRKVVGPALLT
SKVAAYEPIISKNTSIFLSRLTDARSLSRDTSTVNVAPLIHRYTFDTLLEIVYGEPICPQPYTDTKAAHN
VLTGFRDLSKLAWGAALLPWFGWLMSTRPMVYLIRRPTYDSEGNLTSIAALAANTRDVTFAHPEK
ALQSTQPSCLKAYLQLPPTDPKHMPPAQIFRECFNLTFAGPGSTAAALTATLHELGSDPRGQKWQA
RIRADLRTTGSSSPTSSPVLVAVLKETMRLHAPFPTAFPREIRPRAESAIPHLPAPLPVGTIVSANSYVL
GRSKEIWGADAEEWRPERWLGTVAEKGEGKRLDDKFVVFSKGPRGCVGREIAMLVLAKAVAAVL
GRWEVSATGACKRRNFLEMQYEECWIKFGDLAG*
>CYP5317A2|126469|Acastr1
MDTLFNSTVTGASVEVTSNLVNGARDLSFVYASAVVALTALLLSGFEAGRKILNRILWFFDGILGGA
PHTVSLPGPPGLPIVGNLLELSNGHVPKIAEWTKKYGDVIRVSLGEREAVFINSHGALAKTVVQQGP
AYQSRPTFKLFHSDFASSGIWTVGTSPFSDRLVRTRKALSSQISPRLLPIYTPVIHPKLKKLFGDILTISE
GPAVDMAEKLHRFGTGQVSEQLMGIPLDDDMVGMLAENETNIFRQRTIGSPARDYIPILRGAGWL
RYVAGKMLGIKNWSFDEKEEMAREYRKRQQVYIDKMLDSLMERIKNGDETPSILGNILRQDLLKKE
EVLLASYTGIAAGVNLGYSLTWIVGYLANRLDLQKNGYEAIREVYNGEPPKPHEYDRVEYVKALHT

EGSRIYTPVRLGFPRQTLDGASYNGHKIPKDMLVIMNLMAGNRDPVAFDRPEEYLPERWLNGRKG
RTDIPGQGGEKLGVTHLTYGCGRRVCPGIDMANRGLYSTLVLLLHFFTWERQPLGEEEKKHVFPLF
RAERECSLEMDAIADTATPTEAQAIPWSAGIKFGRPVSSFIAVILKAFAPGLRLRRPELSCRSRHELTG
VVSDKLFQASLYTPKSISLSEFSFDLFRAGKIQHQNV*
>CYP665A2|126478|Acastr1
MELAGTMSLSVVGLCLPIVLVMAVFSKYFLASCRPRNFPPGPQTIPFVGNISQVPKVKAFLRFHEFRA
DYGSIIGLKLGSQSVVVLNSYKHVRALFDKRGAIYSSRPNSYIANEVVCPNEIHLLLLQYGNEWRKQ
RKIVQSLLNVNAVDNVLPIQNAEATQTMFQLLQDPQGYYDHIRRYTTAVILASVYGQRGARFDSPN
VQALYHAQDQFTAILEQGATPPVDAFPFLKMMPEFMAPWKTRAKAVREEQKSLYLALLRETRERM
EAGKGPACFLHLMLKDQEKNDMDDEHIAYLAGNLMEAGSDTTASTLLSFLLAMIKYPEEFKKAQR
EVDQVCDTLRSPTSDDISRLPFIRACMDETLRWRPVAAGGIPHMLTQDDTYHGYFLPKGTIFLANT
WAIHHDETEYDRPEEFIPDRFLNNKFGTRNPVDESTEDHRRVSYGFGAGRRVCPGQRLARNSLMV
NMAKIAWGFDLSPGLGPVNVDINTAYSDGFLIAPNKFPILFTPRSDKHKDIITKEYEAIKPFFIKYRN*
>CYP530A44|126571|Acastr1
MVHLGECFESYHISFSTWSLHAPTYHIQDLENKQMLYEMVETPAQFLEHIRRYSNSLTTSMVFGWR
TVNYDDPKLKQLFDGFGEFSTLIQTPTAALLDSFPFLRWLPDFVLPMQAQAKKLHKTEKKLYVDH
WLNCKASIQDGTAKPCFCVDLARQQKEHGFSDDQAAYVSGSLLEAGSDTTSSTLYAFVQAMVLFP
DVQKKAQQEIDRVVGPDRLPTMEDEPYVQYIRGCIKESLRWMPTAILGFPHAVIRDDEYLGYHIPK
GAGVLSNVYAIHMDPTRYPEPRRFNPDRYKDDFLNLADSAANPDASKRDQFTFGAGRRICQGMHI
AERSLFLGISRLLWGFDFAPVTGADGNLVVPDAERLTQGFVAMPEPFQAKITPRSEWKAELIRRVW
AEAKKDLNVETEQWEGIPDGMALSKF*
>CYP51F1|126818|Acastr1
MGFLATIAGPIPSPFSKSSTGVAIALGFTSLIVLTILLNVFKQLLFRYPNEPPVVFHWVPFIGNTIAYGID
PYKFFFRCREKYGDVFTFILLGKRVTVFLGTKGNEFILNGKLRDVNAEEIYTPLTTPVFGKDVVYDCP
NSKLMEQKKFVKYGLTSEALRSYVPLIAGEVYKIVDQSPTFKGSTGKFDVPSTMAETTLYTASRSLQG
KEVRDKFDSSFADLYHDLDMGFTPINFLLPWAPLPQNRKRDSAQRKLAQTYMDIIRARREGGSEKD
TEDMIWNLMSCVYKNGSSVPDMEIAHIMIALLMAGQHSSSSTSAWIVLRLATRPDIMEELYREQVD
VLGADLPPLTYDDLQRLPLHAHVVKETLRIHAPIHSILRKVKSPMLVEGTPYTIPTSHVLLAAPGVAS
RIPDYFPNPLTWEPHRWDAVADHKAEEEDEEKIDYGYGLVTKGTNSPYLPFGAGRHRCIGEQFAYV
QLGTILATLVRQFKFRNPPGVKGVVETDYSSLFSRPLGPAVVEWERRGKSG*
>CYP676A8|126930|Acastr1
MAPALGSLIFPSKLSNLNCTSTQVVSLSLLFIILFALKRRYLTPIRDIPGPILASFSDLWKLWHIPTGHIE
EATIALHKKHGTFVRIAHNEVSVSDPDAVAQGNFYAAFAVPDHNYVNQMSEMDPKEHVRKEKN
VAAGYSLSNIIKSEPYVDATIELLVRNLDGLAQAGEKVNLDRWFTYCAFDIIGEVAFSRSFGFLREAR
DVRGAIANSRNLFLYISLIGHAYWLHQVLMANPILGWLNLQPTSHIFDTCLAAVDSRKKNDKVRR
DMMERWLEVRRTYPDRMEDKEVLAAAMVTIGAGSESISTTLQAFFYYLLRNPPHLLRLRKDVDAA
HRRGELSRIVSYAETQKLPFLQACIKEAYRFHSAISFNLPRVAGEEGVTIAGRTFPKGIVISLNPWVIH
RDKGIFGDDAEVFNPERWLDPENARVMEKHLIHWGAGYNMCPGRHLAHLEISKIAATLLRDFDIA
LVDPTQEWGWRSWFLPLPSGWPCYLTRRDLGKISQ*
>CYP61A1|127065|Acastr1
MAADMANGTFASPVASALYVATLEPNTGGLLSQLYKGLNGWSVTITLLLLLVAYDQFKYVWNKG
SIAGPSWKIPFMGPFLQSVNPKMDEYKSKWASGDLSCVSVFHKFVVIAASRDMARKVLNSPAYVKP
CVVDVAQKLLRPTNWVFLDGKAHVEYRKGLNGLFTRQALELYLPGQEEVYSKYFDRFVEVSQEEN
RGKPVPFMPHFRELMCAVSCRTFVGHYMSDKAVKKIANDYYNITAALELVNFPIIIPFTKTWYGKK
AADMVLDEFAKCAAKSKVRMAAGGSVTCIMDGWIKSMQDSERYRRKVAEGVESEKPATLLRNFS
DFEISQTIFTFLFASQDATSSAATWLFQLMADRPEILAKVREENLAVRNGDRDREFSMDILEKMTYT
RAVVKETLRYRPPVLMVPYMVKKSFPITDSYTVPKGSMVVPTVYPALHDPEVYPDPETFDPDRWITG
DAEKAAKNWLVFGTGPHYCLGQTYAQLNLMAMIGKASLHLNWEHHVTPLSEDIKVFATIFPQDD
CPLVFSHRN*
>CYP620E39|127250|Acastr1

MAIALPTTLTVFFGIVALYLISRLLRGQGNGRPLPPGPKGIPILGNINDMPKPGTLECHHWLQHKDL
YGPISSVTVLGQTIVIINDPQIAFELMRDRSAIHSSRPSQIFSGEIVGWKNATAMSPYNDTWKIHRKNI
MKVTSTNVSVSVFDRVQEAESAHFLLNVLDSPSDLFDHIRKEAGSVILKITYGYTAEAHGRDPFTAE
AHGRDPFVGLAGKTMQTFAEATVPGKWIVDILPFLRYLPDGCPGTGFKDTGRRMASTLRQCVDQP
YEFVKQQMREKRHKTSFLSQAIENIGSNAEMEFIHKWSALSLLTGGADTTVSSLMTFFLAMNVFPEV
QKKAQEEIDRVIGSGRLPVSADRDNLPYIMAIMKETHRWHPVAPMGLPHTSTAEDVCQGYRVPKG
AMLMPNTWWFTHDPAVYPEPMTFRPERFLETPTHKPEPDPRQFIFGYGRRICPGRYVADNALFITT
AQTLAVFNIEKLVENGKVLEPEIKFEPGMVSHPIPYRTSIKPRSKAHEDLIKAAEQEYPWEESDAKEL
GNIKW*
>CYP527R3|127753|Acastr1
MAKTALSGRMNHELTTVIEKYGSLARIGPNDLLTSDPETIRKMNAARSQYLRSEWYDSMALDPDL
HNILSEKNMARHDSLRFRMAAGYAGKENPTLEQNIDDQILELVRVIERKYLSTDSVLRTLDFGRISQ
YFTLDVITNIAYGKAFGYLPKDEDLYNYIETVEAVGPFLNFMSVVPALQKFLSMRWIRWLIGPSVKD
KHGMGKLMAVAQQVVSERYGPDRKDRNDMLGSFVRHGLSQREAESEVLAQIMAGSDTTATALR
ATLLHIITNPRVYRLLTTEIHTALSQNLISSPIITDAEAKTLPYLQAVIREGLRIWPPVSAWLEKEVPAS
GDVIEGRFVPGGTKIAVAGWAMQRATTVYGADADVFRPERWLEAGRRRRGR*
>CYP53A61|127786|Acastr1
MFLSFLFTPYALLLLPIIFYLLPYIRNWSIRDIPAPFPATFTNLWLLYQCRRGRRYLAVDNAHKKYGPL
VRIQPDHVSVADPEAIPIIYGHGNGFLKAEYYDAFVSIERGLFNTRDRAEHTRKRKTISHTFSTKSIGQ
FEQYMHHNLEMLVKQWDGISSTAPKDNGGYAYIDSLHWFNYLAFDIIGDLAFALKPYAKYLPDRF
FRDGLEAVQNLAGIAVARVNDRLQPGVAEKNERVDLLARLMEGRDENGNKLGRAELTAEALTQL
IAGSDTTSNTSCALLFWCLKTPGVIPKLQKELDEAIPDKNTVPDFATVKDLPYLHNVINETLRIHSTS
SLGLPRLVPPGPGITVHKHHFPQNTVLSVPAYTIHHSTAIWGPDADVFRPERWDGVTEEQKNAFIPF
SYGPRACVGRNVAEMELALIVSTVFRRYEFDLRQDKLETREGFLRKPLACEVGMRRRS*

>CYP505A69|118843|Acastr1
MHSVAEEVVAERRRHPSDKKDLLNAMIKGRDPKTGEGLTDATIYNNMITFLIAGLLSFLFMYLMKT
PAAYQVAQQEVDKVIGKEPVTVEHMSTLPYITACLRETLRLQPTAPAFSTQPVASTTEWPVLLGNE
KYEIMPGQVIVALLPGVHRDPVVFGDDAESFRPERMLDEMFEKLPKNAWKPFGNGVRACIGRPFA
WQEALLTTALLLQNFNFHLEDPSYQLAIKQTLTIKPKDLFMHATLRGGIDPVQLEKAMFSRGSQEL
QVSSKDKKIEKMTISGGAKKPMSIFYGSNTGTCEALAQTLANAASGHGYSARVAALDSATDAMPR
SQPVVIITASYEGEPPDNAAHFIQWLGNLKGSEIEGVQYAVFGCGHHELLDARGGQRVAERGYAD
AAAGDMFNDFDQWEDQVLWPSIRKVFGDNDANAEEPSGLDVEVTGSVRISSLHQDVREAIVTDSK
ALTAEGEPPKRHIEIKLPTDMTYRAGDYLAILPLNQPPIIRRVLQRFELPWDAKITIKPGQNTVLPVG
EPLSVFGLLGGYVELSQPATLKNVIAISKSTSNEATKQEFQVLCGDSFKSEITEKRVSPLDLLERFRDIR
MPFGDFLVMLPPLRIRQYSISSSPLADPTSCTLTYSVLDQEALAGGKRFMGVTSNYLSMLESGERIHV
VVKPSHQAFHPPLDIANVPLLMVCAGTGLAPFRGFVQERAKQIEAGRQLAPALLFVGCRYHDRDR
LYPEKFDEWERLGAVDVRYAFSREPEKSNGCKHVQERLWNDRKDVVALFDGGARAYIV*
>CYP5087A3|118963|Acastr1
MHVKWAMTLLIHFLAILTVFTATLSLQKHIRYRAYRLLSITHGYQDPPKEGPYDIFGIFKVLRATRHL
LNKTALADTIALFERYGETYASKVLIQTVFFTCDPRNIRHVLITRFVDYDSSVVRVHLFRPITEHGIFA
VDGSDWKLARSLYRNQFANTRLIMDFDMHEEHFQSLLRSIPPTGKPFDLQPLFLNLTLDLTTAFALG
ESVDSLSPTQSDEKKHFVHALLYVKKIIARNGFLGPLHLLLSKRDFYRACSDVHRYVGCIIERTLDEK
QEEDEKGGQTKDSSRYNLLRGLMENSSNVVELRDGVITVLIAGIDSVASLLSTTFWLLARNERVYQK
LRTSVFDTVGQELPTYDQLKSLTYLRYVFNEAMRVYPPVPFNARIANKDTTLPYGGGADGTSSILVK
KGQRVVYSSWATHRSIKSFGDDAHDFRPERWVNMKAEALGYIPFNSGPRVCPGQQYALMEASYA
VVRILQTFPYIKSHDSRGFTEHIGLNLSNENGVVVELKRG*
>CYP5236B1|121044|Acastr1
MAIVINLQDGFVYVHSTTAWSQLVSVEKELAPRAWLSYINPKCSQIQMAIVEDLLGAFKPGIALAVL
LFLLIATLVAHPLLFSSLSHVPGPIWCKLSWYYIAYFDVRLQRNDKIAEWHTKYGPVICQKRMLISNF
YHRTTINKPVVEQPIRERMHQLFNQINQRLQADAHTRTINLYPIFNCFAFDNISHLLYGPRHCTYTIE

NDCHERQLLLRMKQAQLWGPLKFNFPLVVQTSILTKRFLPEGFSESLVAEHNLADWNWRTLMEAI
EDKDVANDFTLLARMLAVRDKEGKPLDLNYIGSELFDHLNAAQETVAVALAYVAYHLSDHHDW
QEMIRKELQALPVQEDGFPPFAAVETAPLLDAFIREVHRVNPGASGRQERYVPDGGKEYDGIMLPE
GVRASASTIALHHDETVFPSPEFFSPWRWLNLSPDQLQRMERSFIPFGYGARHCLGKALATMELKLL
IACLCLRYKMEVDRDTMTPKAMWQTGTMDSVPYGLRCDLILHPVKLP*
>CYP5192H1|121236|Acastr1
MTPTLSAMLVLLAGLLINSAHQLLPQTVQATTADERSLLRADKPLSRTSQCRLITHFSATYPSSPRSC
HIPVFAKIVRSLPPDVHPHCYPHYIRQAYGLGPIFYLDTWPLGDPMLIIAGDPEAAHEVTVTHSLPK
HWSLQAFLAPLGGWENLVSMEGHVWKAWRSIFNPGFAPANLMGLVPNIVEEVEVFARVLGEWA
DRGEVVILEEVATKVTVDVIGKVVLDTKLGDMCGIDIGGMQFNSQTSNNELVSALRSQVRWSWPA
DMYAPIRNLNPIRPFVFWWNTRTMDRYLDKELERRFELGSGGEMKKRKYVIDLALQTYINEQSPER
KGKGFDKTFKKMAIDQIKTFIFAGHDTSSSTICWVYHTLHQHPQALAQIRHEHDTIFRTDLSLAPQL
LKSTPHLLNKLPYTLAVIKEVMRLYPPASSVRSGEKGYSIRNTYPTDTFLVWPALYSLNHDPAFFPSP
NKFLPERFLPVPKPPSSTSTSTSTLTDFPPAAFRPFEHGPRNCIGQDLALLEIKIVLVLTLRAFDVRAVY
EEFYATEGSKERKKIDEVEGEKAYQILWATAKPKAGLPARV
>CYP6677E1|121603|Acastr1
MHKELGDIFMHVSPGDTEVYISNAEAIYEVCSRKRDFPKPLARLEFLNAFGKNVDTVEGHDWQRH
RRITAPAFNERKNNLVWLESLRQAVDMRDWWVQSGPVGSSNVAADTRTLSLHVLSCVGFGTSHD
FRKSGFSAPTHGHTMTLRESLDVVYSNIFMVILLPHRLLSLPFLPKKLFNVGQAIEEYKRYMVEMIEQ
EKQLLTQRAPGSNNLISLLVRGSEQVQTALMSDTKTSSDSAEGLTDSEVMGNVFIYTLAGHETTASA
LNVFIYTLAGHETTASALSYCSLLLAVYPEWQDWVAAEWQDWVAEELQHVLQGHERIETWDYET
LFPRLKRSLALMLETLRLFHPVTAISKYTADQAQQLTINGKPHIIPSKTVVQLNTTAVHTHPRYWGN
DSLLWRPSRWITSPKTGAAFETVLDAETVLEPTKGSYLPWSEGARICLGRKFAQVEFVAAMAVLLSG
HRVRIVPKEGESLEEARRRALGLMEERRLDVVVEKDDQESVSVAWDPVIMPGEGGHQ*
>CYP61B1|122715|Acastr1
MSITGLLLLDSVVAALLVKSYVLTYLPSHSLFLTASALFLLQVLSRLLWRCVLYPKLYSPLRHLPQPPG
GSFLMGHFWQLVKDTSGGPLRAWVDEVPNDGIILYPDLFNAERVAVVKPKALAEVMVTRSNDYV
KPPHIKKGLGEVFGTGLVFLEGEKHKVQRKRLMPAFTFRHVKDLYPVFWRKSTQLVEALEAYTSDT
GADASSVVVHVCDWASKATLEIIGAAGLGQDFNTIGDPHNELVKTYQTVCSPSKTAIVVGLLGFTA
PARILGSFLLRFTDDFLGAAGVVRRHCHSLVEAKKQYLRKADQHPSVDILSIAVQAGDISDDDLVN
HLMTFLLAGHETTASALSWAVYFLCKYPNVQTRLREEIATHLPHVRDAAWSPEAADIDNIPYLHA
VCSEVLRLTPPVPVVPRMAEVDTSIVGHFIPKGTVIILAPWAVNTSKELWGEDAAEFNPERWMGPG
NANTGGAESGYAFMTFLKGPRGCIGEAFARAELACLLAVWVSRFETELADKDCVVTVKSGISSKPA
PLDVRLKTLGACRSRC*
>CYP530A42|122732|Acastr1
MSVSMTLVIVGVVTLVLAKLSQIGRRPAGLPPGPPTLPLIGNLHQMPAKDGHLQFQKWAEEYGPIY
SLILGTKVMIVLSSDQAIKDLLDKRSGIYSSRPEMYIGQIVSGGLRVLLMEYGTTWRMIRKMVHNILN
IKAAKSYVPYQDLENKQMLCGFLDCPELFIDHIRRYTNSLTTQMVFGFRTISINDPKLKQLFEGFEKF
CEVTSASTAALLDLFPVLKRLPDFCLPLRSYAKQLHKKERELYIGHWMNVKKAIKEKTAKPCFCVD
LVKAQDVEGFSDDLAGYISGSLLEAGSDTTAATLVGFVQAMVVFPKVQKKAQEEIDRVVGPDRLPT
MEDEMSLQYIRGCVKESMRWMPTDILGVPHAVTRDDEYMGYKIPEGAGVMWNVWAIHMDPNR
HSNPRAFDPSRYAQDSQTASDAASNPDASKRDQFVFGAGRRVCQGMHIAERSLFLGISRMLWAFN
FEKVKDADGNEITPDINKLTQGLFVLPEPFPAKITPRSEKHAERIRKEWADCQQLLDGNKQWREVP
KGMAFSTYDPDLLVKH*
>CYP578Z1|123776|Acastr1
MLWNRFKGRESTAVHAAHVKYGPVVRLAPNEISINCVDGGTRTVYGGGFERHEWYIHLINNLVSII
PSKPHSIRKRMVSNIYSKSYLFSSLPLHALSQTIIYDRLLPVIQPVGENRTLDVLELFYAVNMDLMSGY
LFGLTNGTNFLQDEKTRKHWLSIYQRRKTHMFWQPEFPNLTAWLGRIGIRVVPREFDITTKEIETWC
LSMCDATEQFLATESITEWQDPKFEPVVYKQLKHGLANDRSDQPPSHLPPPDQRLTLASEMLDSLIA
GFETSGITLTYITHALSLRPHLQARLRAELLTLSPPLLYPREPHTPAQLPSSKAIDALPLLHAVIMETLR
VHPPVTIGQPRVTPPSCTTTLGGYTDIPPGTRVNAQAYSLHRNAEVFPEPEEWRPERWLEGKIENQG

ERDEKSRWFWAFGSGGRMCVGSNFAMHDHIANMNFSQVIYLIEAYTTVWTVRLGVKGILYICSGF
LPLDGELERGTSRSVGLQNTKSQLSDTTREDSPHP*
>CYP52AZ1|125462|Acastr1
MASSPILQAVFLFPLVYLLQRWFRGYLAERRFQRFARENGCQPPHLSVNKLPWGIDRIIEVFRFKGD
MLDDLIFARYKQEGAWTYTHTTLFGQHILHTVEPRNVQAILATRFKDFENGEIRHHQFGPLLGYGI
LTSDGPAWEHYRALLKPQFSREQISDLKVAERHVQHLFQALPVKDDGWTEGGDILELFYRFTLDVI
TEFLIGRSVNLQLAALGKAADDTQASAEAYFRDEFTVAQETLMWRIRMQSLYWLMDSRAFRRACS
MCRRFIDQYVAIALDPNRLAAEKNARATDGAKEKYVLLDKLAETTQDPIELRDQLLQLLTAGRDTT
ATSSGFIFWTLARHPEAWKKVREEVFRIFGAEGQGEEITFAKLKSARYVQYVINETLRLYPVAPLNN
RFAIRDSVLPVGGGPDGEGPVAVRKGTLVNFSDYILHRREDIWGDDVLEWKPERWIDRKHGWEYIP
FNGGPRICIGQQFALTEISFVIVRMAQRFEKIEPLDPGEKLVKGLRLTLVPRNGVKIRLFKGTKT*
>CYP6001C38|125721|Acastr1
MAYLTVPAFFASLFVVAFLYIGGTTKMMKRLSSTFKKGRSKEEEPVNGPHHPQKPGQDGAARSPGL
ERKEAENTHHAVKQEEVQSSFRQYAQLIHAAQRPLPTQSGDGSYLEEDVPSGMMQDLKSLGFQDV
KTLMAVMKTKATGALADDKTYLMERVIQLVCGLSERSRSRIDLTNSFIDELWNSLQHPPLSYMGDK
FTYRQADGSFNNPMIPSLGAANTPYARSVLPATLQPGALPDPGMIFDSVYAREEFTPHPNNVSSVLF
YWASLIIHDLFQTDHKDFNLSQTSSYLDLSPLYGDTQEDQDQVRTFKDGKLKPDCFSEHRLLGFPPG
CGVLLIMFNRFHNYVVDQLAVVNEGNRFAKPKEGLSEELARKAWTKYDNDLFQTGRLITCGLYM
NITLLDYLRTIVNLNRSNTTWTLDPRVDMGKVFGKDGTPRGIGNQVSAEFNLVYRWHSATSKRDEI
WTENAYFEIFGKEAGEVSMQELLSGLGKWEHELSSKDPLDRPFAHLKRGADQRYNDDDLVKIMTE
SIEDTADTNESPWKVHLVPTMSQKPFVLSLRAVEILGMQQARKWNCGSLNEFRKFFGLKPHKTFED
INSDPYVADQLRHLYEHPDYVEMYPGIVAEEAKVPMVPGVGIAPTFTISRAILSDAVCLVRGDRFYTI
DYHPKNLTNWGYNEVAYDLNIEQGCVFYKLFLRAFPNHFKQNSIYAHYPMTIPSENKKILTSLGRE
SHYSWDRPAPIPPRVNLTSYVGAKYILEHANEFHVTWGEGLGWLMGKGGLDFMLAGDSKFHGKQ
RQLMHQALYRDQWHQQIKDFYEYITLKLLKEKSCKIAGVNQVDITRDVGNLAHVHFAANVFSLPL
KTENSPHGIYSEQELYMILAVIFACIFFDLDPAKSFPLRMAAREVSQQLGKLVEANVKMVNMTGWI
AGIVDGMHQHHTPLTDYGVHMIRRLLESGLGPSEIAWSQVLPTAGAMVANQAQVVGAARDPDV
FPSPNEVRVDRPLESYIHYGEGPHACLGKDASRVSITAMMKVVGRLDNLRRAPGPQGQLKKIPRPG
GFYIYLREDWGGFWPFPKTMKIQWDGELPTLKKL*
>CYP511D1|126182|Acastr1
MTIMSKIQTSKGFDLLGYGLLLGILYYVGLVIWRLFFHPLSKFPGPRLAAATQWYEFYHDIILGGVTP
KRYSALHKRYGPILRTTPETLHIGDPEFYIGDPEFYKELYAWNSEYYKAPSLYEAIGVSNSLASMCDP
KKHKIHRSVVAPLFAKQSIDRLAPSVADKVEDAVEVVKHCHAQGKPVDIQLLYRCITVDIISKSLFG
DSHNLVGSYDGDKPLLDSIELFSTSLAMMKHFPILQKTALNLPSILSEKLLPGYASFRTVGHNPVNY
GPVLVLISFPIKDCAKWISSIAARRERGLLHSEDGTPTIFDLMLESDPKKGYEVPTLEELVDDAFVFLF
AGSDSTAYTLSVATFHILEHKDVLTRLQSELKAAPRDTHGTFVWKDLAHQPYLTAVIKESLRLASPV
AIALPRIVPPAGTYVGGEFIPGGTTVAMSIFTMHNNPDIFETPEKFIPERWLGEKGKQLDQWNVAFS
KGDRNCIGQYLSYLEMRICLATFFTRFDLELYETDRSSMEFLEKGSLKNASNVKVLAKPILLVPTTEV
LLTSRDRESGSLYSELTFHEDFLESHVLRLRNGTAAGSGSDGGNVRDNRGKALQFTTLNGRTIVVKD
AFVYSNKGFKNLNQAQILNDALYHSDALDSQQWLIYYISRPLIGSFETVNIIPAALPDHSNGAKGPS
RRNVSETEGGPPAPPRKKDIKSFNDLLNNFPMIARQMHPGLARLFKDFHRDFEKSLPAASLRSLKTT
ASGQEEMLPVIRSNSHDSLRHGMLNGHAKSPSISGEYNDDELDTMRRALESAVTAAIDLFQLVDQQ
QLSLLGATTDLTGPVVERLIERYITEQVHDSVLFPKVCRSTRSDDLELDTRIRQMENIDITQVGIVIEG
GRQGKEELMQRLSRGVEEFRKLGVAGSPQEMMDILLATQKTVTSAEVTPSGKKYPSEDSELSLTSEK
PSPIMTINADTLVSLLLIVVIRSQVRHLQARLSYMRHFIFIDDVEGGEVGYALSTFEAVLSYLAKDSGG
LRKASRRNKRLWQATRNGDISELRAVLEPEKGEVTGEDVILDPEHDGEAPVEEPNTIEAETPSAKSM
NGSVYSQYTPTVGQDLESTSEETSLEHVFPFQTQTPSPLAIQHVPRKDKRVSMDMRSLSGSSGYSVIS
GHTTIDSRSSGIEGDTSIEKLSKTQDSTGDSVLMMAVEARQPDALRYLLTLSNYYPVQAILGDSNSQG
TTLLSAAVQLAHTELIDIILDYVFQAEEDRIVIDYFAKQDLMGRTVAHYLFNAPELVSKIGRLLPWRK
RDKNGQTPLFALCRSYDHPHYSDMVNDALNVATQTQGDGQALHLDDHVDNKGNTLLHVVNEP
QLTLWILQRCDSDVNATNDKGFTPLMVASKYGRVDMVRALFGDPRVDLYAKELRGMTAVELAKD

DDVRNRIDDLVLFSNPPAADGRITTVVRSFFVEDATIRLILKSGALNANATFTVTTCRRSLSDFENLA
RWLAREHPASWLPSISSFRSPFQISSKPARSVLYDIQVRLDSFLKVMLSHSTFSTHETLWEFFLVPEVQ
PAMMAERSEKKAEIRVEKVKEEYEPVDDVKDVELFVGHATEMVRGVNHLTKSVIRRVNKIRNTSS
DLSDAQQLSYRALSTLTFLPESHVAAFSRYSTTLRHLESDPHRLFLSDMHSISSTIIAILYALARPQSLIT
LMSDTSKAIDRHTSSLRMSDTSKAIDRHTSSLRRSDRWPLGLLDDTRNKYHLEAAEKVEKSKEELRG
LGSELRYTQQTVASELAGWQDLHEKMGKRAIRMLARRMVVREKDRLEGMKRASIFVLVVLNNYK
PPSTSRSQKLPTTMPLVVPGINNKATSNDPKEEWMNKLAGKKLSDSTTDSTNFARRDLPKEHRVVQ
EGSMLTQDFNQDRMNIHVGKDGTVKNVDFK*
>CYP59X1|127667|Acastr1
MPKDAHPNYLPDMIRRAMPDLGPVYYLDTWPFGPQMLVVASPGSLYQITQEHSLPKYHALKTFLQ
PITGGGDIVTMEGQMWKTWRGIFNPGFSASHLMTLTPGIVEETVTFCDILQTHVQNQTLFRMKHM
TDNLTMDVIGRVVLDTQLDSQRRKNPLVDGLRMQIRWLTFGADPFERYNPLRPFVHWYNTRRMN
KYVYWELKNRMANYQSSESVQTIKRTKTVVDLALAAYLTESQGERRIQGMDSTFQKFAMSQIKLFL
FSGHDTISSSICYIFYILSINSLALSRIRAEHNEVFGSDLAKTASVVIQNPFLLNQLPYTLAVIKETLRMY
PAVSSTRAGEPGFDVMDDQGRRFPTEGFLVWANPQPLQRDPAYWLRPDEFIPERWLVPPGDPLHPI
KGAWRPFEYGPRNCIGQELAMLEMKIIMVMTVRRFQIKPAYEELDRHKPSGKVRTVYGERGYQIQR
AQPCDDLPCRVAKADR*

>CYP532A31|119882|Acastr1
MAALHSALQTLSPTPFSSVPHEKSELKAYLQDAFAKSQVILESVPPPSAEETSAARSRSNTTTSTASN
ASQISASSARTPPPSQEYAALQKEWGKPIKLAAKDNPLGIAVFKLPSKDSKGAWFARRSVHEGLGF
NRWKKSLEREFPESLKVQGGPVYRLSAQFPGPTTPRDFVTLLLTSSTALKDPSSSSTQPSSPLHHDFT
HEPRHFMVVSKPCIHSDCPPRIHSDCPPREGFIRGEYESIEFIREIPVQPKRSTSATDLLKGGKSRPTSST
INKDAILRNASQKSHTFPLHHDDASDHQLSEDGHAAPSSISAERETTSEGRRRGKTISFAESRGQTAK
GEQLAKGEQFDTHQGEDNEDETNPVEWIMITRSDPGGSVPRWMVERGTPAGIVADAGKFLDWAC
KTEHSQSEDDAHVDGLNQQHASHEEENLRDYQTNGHLAGLNGDADDVNVSNENPLIEPPQQTST
APVQSGMLQNFASAAYTGLETYGPKAVVDHLPGHPAQTEPSSPPDMGEPILRGQRDENDASSLAE
TSSVASFASADSHLGDEEGENKSISSKTTSSNNKDGLLIGQDKELAKLNDRKKKLDEKLAKTREKEL
KDRQELTSKEQAAIQKAEEKHAKEVAKQEEKYKKEVAKLEVKKSKEAAKLAEKRKRAEDKDEKA
RLLREKEEVKAELDVVKKERDILQTQVGDLQRENTALAARLGRDENGKGLLKEVRDEMLGGGRG
RRTRSNSLESGGGSDRKRRHLEGAKKSVGSIVAMSKPRVIYWFRTDLRLHDSPALKAALDLHPECLY
PIWCWDPHYVYRARVGPNRWQYLLDCQSDLSASLTKLNPKSKLFVIREAPTTLLPKLFRAWKISHL
VFEKDTDAYARQRDGQVMDLAREAGVEVIVRSGRTLYDSDELVKANGGKPTMSITQVQRAGSKV
GKIPRPIPAPLTIPDPGETPVDFDQEQPPQEPDINAIQRDGDEISYAKLAGPNGEFAVPTMEELGLKP
ATTSHRGGETVALNALDEIIANEKYTATFEKPKTAPTAFLPQATTLLSPHMHFGSLSCRLFYWRAQD
VVTNYAGKASQPPTSLTGQLLFRDMYFGAQASLGYKFGQAYGNSHCRFIPWHLPSQIDSQTGLITG
SHHTDTPLAETWFHRWKYGITGFPWIDGLMRQLRYEGWLHHLGRHAVACFLTRGGCYIDWERGA
EVFEEWLIDHEAASNIGNWQWLACAAFFSQFYRCYSPIAFPKKWDSDGKFVRHYVPELKDFPTKYI
YEPWKAPVVDQKRAGYLVRGDGSEVAEEGGVKTYPRPMFDFDERRTVCIEAIKNAYHVGLYGDHP
KVLDGTWRELFPDDGEGPTEGKNAIDAAMTGSKERIVKDVDPRKGDMGVDEDGSEAAERGVLLE
HWLGVLLIISMAYLASNRFNHGLQKYPGPFLASVTDWWRFIDVWGRRPDITQIKLHRRHGDVVRL
GPNALSFANPKALKQIYGLNKGFTKSGFYPVQQAMSKGERLPSIFSTTDEQFHANLRRCVNSAFSM
SSLVQYEPFVDRTTEVLLDQTQRHFASKNAVCDFAQWLQFYAFDVIGEMTYSKRHGFIERVEDVDG
MVSYLGKLFSYVAPIGQVPWLDLLMLKNPFLRLLDKFSIMSFTFPVVTFAKKHMNDRLAEMQQRK
GNGDGDVTDRRGDLLSMFLKAKADHPDFMTDARVLTLAVSMAFAGSETTAISLAAVFYYLLKNSR
CYQKLIEEIDGAVMDGTIEDRSTGIVTWAESQKLPYLDACVREAFRLHPAAGLTLERVVPSQGAEIC
GEHIAGGTIVGCNAWVIHRRPEVFGDDVDAYRPERWLEAEIDQLKEMNGTMFHFGMGARTCIGK
NISLLEIYKLVPSFLRRFEVHQVDPNKEWKLHNAWFVKQLNFNTTFTPRHMTAK*
>CYP52K8|121808|Acastr1
MLHVYLPLALLSYLIVFAVYNLILRHRQRQVNKAIAARHGCLPPPRLQNQRPLGLDRLEQIFRANTE
SRLMELFLFHFRQTGYTLEQVFLRTQAFGTVDPANLEAILSTNFGDWGMGARRSITFPMFGDGIFTQ

EGAPWKHSRELLRPQFVHKQYEDLEIFREPVETLIDILSTADGVVDLQPLFFRLTLDVTTAFLFGESV
QSLKAPESTGEQTFAEAFNTAQKYVAKRFRLLDLYWLIGGKEFRDACDAVHRFADQIIDRSLSLDVE
GQEKGKKYVFLSTLAKSTPDRNALRGQIINILTAGRDTTACLLSWAFFLLVRHPHVLSNLKAEIASSC
QNSADLSRTDLRNMKYLQNVLKETLRLYPSVPVNTRTALRTTVLPTGGGPDRKSPVLVPKGTAVAY
SVYTMHRRPDFYGMDAELFRPERWDEDMPLNHTPVNAKWGYLPFNGGPRICLGMDLALTEAAYT
ILRIIQRFPTIELPEGEVVDLMGVEKQTMTLVMSITEGCKIQLL*
>CYP6003E1|124731|Acastr1
MAAPGRINDRSTHQEANPSTSQSGTQQPPEAQMTSNAAPAEQGEQPAASRRKRTHRGGKKKRNR
RQSFAAPSDDTNTANPARANRDLLDVPSSSTARPPFYKLGQSGGRNLSSTSLDSEALLDHRDHRPM
RARRESRTTQNTFGGRPGSSRNDSNSTARPTYTNPFVSSESAHRRPRVYRAHRSSGGESEENDEVTD
DRTPLMSSTSRDRPRSGGPSGYGGLGAQGRVNRPDTQARRPSTGTSRSSQHRRQGPIWEQSHSDPQ
QDYDVNNPPSVPNSPQLGPEMGYDDVLVTGSFGPSRSIEDRRATAPALSDHIINIDHAPGADQTYG
NSSAPTPRAASGDQRRRTVALQAEEDVCFPIEGMSEMAEDDYMHQDQGSVRCGTRRRRSMRWPD
LSVLEEWSREEKEVRSEGIRTKKISEPVLVGGRLRPQKNGWHRTEEDAPYRFTYFNEEFQSTIHSQTIS
ELLQPGQDFKELFIPDPPELSDYSSEEEDDLETVDERQELSPTSGSKAGTRHSSIIGESKHHSREPSGDG
HDNAQSQQPKERPKRYGPRPTFWLDVLSPTDTEMRVISKTFGIHPLTAEDIMVQEAREKVELFRNY
YFVNYRTFEQDMNNEDYLEPVNMYVVVFREGVISFHFSMTPHPANVRRRIRQLKDYLILSSDWISYA
IIDDITDVFAPLIQSIEDEVDDIDAAILQLHNADNVDTSKEKSKAKDNERKSESGDNGPGESGRDML
RRVGEARKKVMGLYRLLGNKADVIKGFAKRCNQQWEVAPRSEIGLYLGDIQDHILTMTSNLSHYE
TLLSRAHSNYLAQINIRMNERQEQTADVLGKLTVLGTIVLPMNIITGMWGMNVEVPGQRVDNLFW
FWSITAGLLAFGIVYVYKYGGKNLHIGFISFPRASSIYEEASVEFSGSETGNLRNKEKRRESRDLGVKG
KLLSYLESQDGIGALLKKLGDNRGKLYRALLAGNAGDMRTNLRKLPVQLPLDDPVSKLASGFAVH
AYYSAMLHPPQTYLGNKFQYRSADGSDNNMLFPQLGKAGMPYAKSVPNVRAVHGSQPDPGDLF
DLLLARDETDDEDIRDSNLGISSMLLYHATVIIHDIFRTNMGDKNISDTSSYLDLSPLYGKDAEAQRT
IRSFRRGLLKPDSFAEERLLNQPVGVCIYLIMYNRFHNYVAQQLLEINENQKFFQPPSERPASWMPN
GDWQPPRHWAPVGIWKPAKWQELDSAKQWSSYRQNLTKAAQENEAAKQEMWMLWKTAAEEK
LDEDLFQTARLITCGLYINISIHDYLRVLTRVHLHNTSWTLDPRKEIPIAGIDPRGIQRGQGNQVSVEF
NVLYRFHSPLSRRDMKWSSTFLKQLLQGFVEPKDGSHAQDKTSLTQDQLDTFDIPIPVMRDALAKM
YKMLPTVDDKLIAPAMPIGLGHAYWPGADTREFGAKNTARLFRSIEILGILQARKWYVNVPFPIQN
LKAYSYYISIYNRELATLNEFRKFFGLPEHEKFTDINQDPDIARKLKNLYRHPDQVELYPGLVCEGN
GRCLDPGTKGPKSDTALWSAVFSDAVTLVRSDRFYTTDWNVGSLTGWGMREVSSVPEVMKGSVM
HRLFQRAFPGFFAYNSLHLWQPFHIPAMNYVLAKHQGKLSDLEDLSQMGVNSHNIEEVEKFIASTK
EGDTGLKALGSQTKPLKELEEKGILGYTRTEIKQIKEIIQNGDAAYEIGAPQLAFKPWKRLIKMPETR
FCAPTKATTICVTNYSTIVDEILAKRSIYKNPGFLDYQVIPEGALRGILTGNPTGKLKKDFEERVEGFF
TRLRSKVTPDKEKVFMDYFTQQAQDYLSSGKRDYQKLKSKVEGKEKEVQVWQIDIVSEYVHLRTTV
KSYIDWFTNMLAHSYAMPVVAQFIADFLGFWDWIDPKIPEGIENAKVVYEHLNNCQDYLTYDSDE
TTVASRRVAFNKSIIWLNDRAKYGVKQVIANGAKRGEHGPNDVASELRQFGTQMALELLEGGPDK
KTPATVDEVAAIRLSIALDAVHKSILMVSGPAANYRTYAKAIQFTEVLNYLLHKTDGSEYLWEYIQS
LASKTSNPGEAEKKLTEAEKKLTEAEKKLQRRNSGRCVLEAQRLAVHVPLVRQVPSDAKVDAVEH
PIDDNKPPTTRKFQRGDMLLLQVDKAQQNPEHFPNPTEFKFQRREDTDDSGEYIIYGRKKGAPFTA
KHLTIIAITALIKQAAKLKSLRRAHNTEGRLNKAKTPDGVQRYLTQEFDQLVPFPTTWQLRFDGFG
QGVWKGKDNSELQHGMDQVYQDGALSADGGSFTSS*
>CYP52AA10|124970|Acastr1
MPKSLAKVHKKISKKRGNTNSLNENSRDAQRLRRAGARDDKLAKLSAARAKVNQPHIQRVAFFQ
QATRELKAPVPVQVVQGLIQSYITQDDEELAKLKTERRPGRPSSTREDLLKQRIDTENREYDAGFWIP
DMEDPDNIARLENWMGDWTSLNTLTKRETINIIINMHPYILVSLGAGVAFLLYKVVSNILASRYYAA
EAQRLGCIPPPKQENNWPFGVDRVRRNLLRADTARVFPDFMAQRFREMGVNTFEFSMLGTQGYLT
ADPKNIQAILATQFNDFSLGPNRRNAFMPLLGNGIFTQDGKPWEHSRAMIRPSFVREQVSDLELEE
HHVQDMMRALIVKQDGWTAPVDLQVLFFRLTLDSATEFLFGESVGSQVANLPAEAQVNGAPKVT
QDEKVFAKVFANAFDRYLARRGRLLDKSWLDNTPGFRLACKQTHEFADHFVRLALHPELKEKEK
DMEKGGRERYIFSEALAAQTQDPIELRSQILNILLAGRDTTASLLGWTFYLLVRHPAIYDSLRATILA

DFGTYTHPRDITFQNLKSCRQLQHTLNESLRLYSVVPFNSRIATKDTTIPVGGGPDGKSPIFVRKGQP
VDYSVHVMHRRKDLWGPDADEFRPERWVGRKVGWEYLPFNGGPRICLGQQFALTEASYVTVRLI
QRFDRMENLDPEPSPKHNLTLTNCSASGVQVRLHEARD*
>CYP584E29|125593|Acastr1
MAMEMYLAFHPKLVFALVAALGIIACVSIRRCFIRRQFARGHGCQTVARSLNKDPFLGLDTIPGTLR
ALRQHKILEKSCESFRVYGNTFTLKELHRRAILTVEPENIKTILSLKFKDYGISHRLEPFKPLLGEGIFD
TDGDHWASSRALIRPSFTRDQVADLTSFEDLIQDLFSLLPRDGETVVDLQDLFFRYTIDSATEFLFGQS
VGTLKKTQSELGFAQAFHYAQKAIITRGTLGPLNMLYRDRKADECNRICREFAQHFVEEAFHAVD
VKKEDKEERQAETKRQKHIFSHELASRTSDKHRILDELMNVLLAGRDTTASLLSNLFFMLAKNPAI
WDKLRREVAGLQGRAPTYEELRSLTYVQCCMNESLRLHPVVPRNEREAVRDTILPLGGGKDGLSPV
LVPKGTLLCYNVYAMHRRTDFYGPDAEEFRPERWEDGKLQPRWGYLPFNGGPRICIGQRYALTEV
GYVIVRMAQEFRVLESRDTGPWEESLTLTLCSRNGTKVCLTPA*
>CYP584L7|126332|Acastr1
MAEYQFPPYVKTLVGGLVPPYVKTLVGGLVLVALYYLYHQLTVGAARRRIIRENGCKPPAKYPHVD
PIMGVDLFLTNLRALRENRFLPTMRQRFNTYGNTFQLNLMGTKVIATIEPENLKTILALKFKEFGLG
ERRKTALSPLLGYGIFTTDGGAWQHSREMLRPNFTKSQVGDLDTFERHISRLIQAIPRDGSTVDLSEL
FFRLTIDSATEFLFGESTNSLTPGTSMTSAARFAETFNYSQEAVGNRFRLGLLSHFIPNRKFSRDVKYV
HDFVDSYVKKALEYRRTHDLEKADVKADERYVFLHELAKQTDDPIQIRAELLNILLAGRDTTASLLS
NVWHTLARRPDIWAKLRAEVDTIGGQRPTFAQVKDMKYLRHMLNESLRLYPVVPGNSRTAVVDT
FIPLGGGPDGKSPVFVPKGTMVSYSVNAMHRLKRFYGEDADEFKPERWETLRPGWEYLPFNGGPRI
CLGQQFALTEASYATIRLMQEFKAIANRDPSPWAEALTLTCTSKNGARVSLSPA*
>CYP539A51|126516|Acastr1
MLDAIIHNISFGSLALIGTAALTAWYVACQIRDSRRIATLGGRAPKVAIYLPLGLDLTYRGVSAAIRH
KNLEFWEWLFSSFGTVGSPYTVEAQVGPQRFIFTAEPENIKAILATQFEDYGKGQPFHEDWRDFLGD
SIFTTDGEQWHDSRSLIRPQFIKDRVSDLAIFEKHVNVLLGLMGGKGQEVDVSALFFRYTLDAATDF
LLGTSVDSLHNSQVEFAEAFAEVQRVQNIIARAGPMNRFVPRRSFYANLKILNSFVNPYIEQALRLSP
EELEKKTKSDSGYTFLHALARFTRDRTVIRDQLVAVLLAGRDTTAATLSWLFYELSRHPAIVARLRA
EIHTHLGPDRRAPTYTDLKSMRYLQNTLNETLRLYPAVPFNVRMSLKETTLPRGAGPSGLSPIGVLA
NTAIGYSTLVMHRRRDLYPPPSAHFPDIMEFAPHRWESWTPKTWQYIPFNGGPRICIGQQFALAEM
GYTVVRILQRFETVQRYWGDGEQ*
>CYP5328F1|119780|Acastr1
MASYLLLSLILPLVSIIVWNSYKLAANYAVARKVGVPLIVLPISPENPTWVLISDFCIPMIKRIPFGSGT
FTRYCHRGWEFYDRNRTHLELGDVFMAVTPRKNVLYVCNAEALADVIERRNEFQRPLEVLLKTLD
MLTLFGPNISTVKGKDWQRHRKMIGSLFSEHINKVVWAESLHQVRDMLHYWTTQTPNAVRSTAK
DTKTFSLHVLTAAEFGKTFPFQSSIESAETGQMLSYRDSLSLVLDHAILIMIVGSRFLTAPFLPKRWAR
VGQATIDFQQYMTDMLEQEKRLIVQGAPGSSNFLTTLVRASQAVSEPAASSGGCKGAGTRRNSQG
GLTDAEIYGNIFVLNFAGHDTSARSLAYCLTLLAAHPEVQDWIAEEIRYVLSESDSSTWNYEESFPRL
KRCLAILLETLRLYFPVIAFIRSTGADARTLNIAGKPLLIPANTLVIPSIMAIHSHPRYWGHDALIWRP
SRWILETSIPGEEEGDAVSDREILYTPPKGAYLPWSDGPRGCPGKKFAHVETVGALAGLFRDHRVEP
VPEDGEDMEMARKRTMDVVNDSGMVLLLQMLKPERAGLRWNTSYLPHPCSPTEPPSFSQPPHPIP
HKMSTTPPAPQSHPTTSSTHNPPSLPTSPLPKRFKPTTSADTTTTTANNNSKTTMPDATSTPATTTIE
QSPPLLIKKLSAKAKTPTRGSQFAAGYDVYRPDGMAWDGMGREIGVGSRADENFLTGKIAQNTTIP
AHGKALVETDIAIAVPAGTCQLAPISSVDLQPLLAALVRPFYPLLHHGIPSHLPSHPLPSHPLPSHPV
PTYLPTCHRTRHYQTADRSGPEATDGRIAPRSGLASKNFIDTGAGVIDADYRGMLKVLLFNFSEVDF
EVKEGDRIAQLVLERIYTPDVVVVEELEESVRGAGGFGSTG*
>CYP6690A2|120689|Acastr1
MSNQFWENPKLMSLLDASPRVGVLSLPIGAALLAIFGILFYVYKKVLLPQPVPGIPYDEKSAQKLLG
DFPDMISEVSQTREVQNWLLKKVHQLKEPLCQVFPLPFSTPWVLLSDSVETRNLLTQNPAFGRSNF
DRTGLTHLDGFHSRHEMGDEWRRTRNWIADLVSPTFINKRIQPILYENAFRLLDFWNSKAYLANG
RAFDAALDLDHVALDAMISFAFDEEFRHAALDPQIEELAKLKPSDIPTGSEGEALFPQAKSRPFTDA
MYTTVEAIVYVTSSLWPAFAKRWVLLSPHFSHARNVRRKVIKAQVTKALGRVEATGEAKTAVELM

LMREKKAADKQGRKPDYYNQVLMDEIGGQFFAGFHTSSTSLSWIFIHLTRNPAVQAKLHDALHTA
FSIAYAEQLHDALHTAFSIAYAEHRVPTQAEISTIRVPYLDAVLDEVLRMQATLLSRLALKDTQVFG
HRIPKGTTVFMLCNGPGFHSPTFEAAKVMRRAAGEKDTEAGWDESRDMAAFDPERWLRKKENAT
DDDDVEYDQNAAPSMGFGMGLRGCWGKKVAYVELRILVAMVMWHFNLQEIPKAFADPKKTLSV
IHRADECFVRLKPREDASDAFAKALSQGASGNYEPAGTGTSSDWSTRSNSRHGQGKYSLAELFGHV
EYSGTNTLALVRKLGLSEDDSQSGNREIPFEAFGDIEKIIRRLPDREIFDCLVQYYVTEVHWMEQVVY
APWFLEQYQKWWSSGRPRLVVDVEFAVLFLRICAYASQFLPSPSYTIDHIRGMPLTNVRDACHDIA
GKLAAICKRLNPTGALIRVQHLLILGLQRGCEGRIRDFWEALSNAVRVAQRIGLHRGRTAWTYGM
HEFDKEIRCRVLCNLYTWDSVLSRHLDCPPFFTFNLTSDHLPLILASKTDDPDAPEDYSERVVQTRL
QKFWRSFPPRQVADHEPHTAEERYQSFCSDYLQHLTPAFALEPSEEWDERLPRLPLQRQMLHVAIF
ASLCYNFRPVLFCDPLQLRCLPAYKQLLLALQKCTLAAAALKLLHSVSKLHAMLGRAHTRLPTIILP
TFEAAVILVSLTIDASFPGLCPLGGEEAPMRPLDSDPLGAERAHLTREKCKQAVQEALARLEMLAEV
SNMAKVGASTLMRLMARVPTGEVVPQTWPTLESYDATALEEFLTVCSAETFTGSTLSFAHPELA*
>CYP51200A1|122305|Acastr1
MFLTVQLWLAGAAGIISHLGFFIRGEHHLEAPKTFRLYVILAGFIFLTNTYCILDVKTGAKSTCLVVSI
YGVALFCSMLVYRTLFHPLHGFPGPFMAKTTKLWNVIQALDSDNFRLMENLHQRYGDFVRTGPNE
ISIFRPEAVRALDGVGSKCSKAAWYDIMQPRVSVATTRDKELHSQRRKIWEKAFTTQALQEYEGRLI
YFSKQLQKRILGSEIIAVNVSSLFYLYSFDVMSDLAFGKPLNMLESDEYHFTVGLLQDGMRFLGPLSP
VPWLVRVGYSIPGFAQNFKGLLAWSAQKLKDRLEDEPTKRDIMSWILDASRQAGTMKQDRHWLR
GDCFAIIVAGTDTVASTLISIFYHLAADPKQMQKLRTELQGIGSITDMKALQSMGHLNGCINEALRL
HPAIPSGAFRVTPPEGLTIAGQYIPGEVVVSAPSIVESCYEQAQDFVPERWYSKPEMIKDKSGFSPFSM
GRHNCIGKNLALMQIRSVVASLVTQFDISFAPYEDGVAVWRDLKDQFNAHPGKLDLVFSARKREII
TIQAGQCGNSVGSQFWQQLCQEHGISQDGNLEDFATEGGDRKDVFFYQSDDTRYIPRAILLDLEPR
VINGIQTGAYKNIYNPENFYIGKQGIGAGNNWAAGYAAGETVQEDVFDMIDREADGSDSLEGFML
LHSIAGGTGSGLGSFILERMNDRFPKKLIQTYSVFPDTGDVVVNPYNSLLAMRRLTQNADSVVVLD
NGALSRIAADRLHVQEPSFLQTNQLAGPPSYTIISATDDAPGLHGHVSTVMSASTTTLRYPGYMHN
DLVGILASLIPTPRCHFLMTSYTPFTSDNIEQAKTVRKTTVLDVMRRLLQPKNRMVSTTPSKKSCYISI
LNIIQGEADPSEVTSPQSLRSPLPKFRGLLPNPQVHKSLLRIRERRLATFIPWGPASIQVALAQKSPYLP
NTHRVSGLMLANHTSVATLFRRIVQQYDRLRKRNAFLDGYKKEAPFADGLGEFDEARSVVMDLV
AEYESAEDENYLEGGGDAGESEGKGDEGGAER*

***Cladonia grayi* Cgr/DA2myc/ss v2.0**
>CYP51192A1|558|Clagr3
MSILTHLFRSWKSMTNAPASMPWAGTRNEILPNFRACMREFIAGLRTLRIGYDNYARKGQAFVVP
DPGLRPQIMVPKEHLRWMVKQPETVLSARLPQVGRFAIEYLVPGLDVNHDLFMMDVIRKDLTRNL
GRLQPSIYQDISESIDELMGIDTDNWRKICLWETMQKTVFKSTNRVFVGLPLCQDEGYLRSSAAFAN
WLEAGAIVVGQCMPSVLKPFFGYLTAIPIYIQKNKSFGNLLPVFKERMENIRRKRADPTFEFNEPKD
MITWMSTAVIGNPTTTHSKSEALAERILFFTLGAIHTTIMTAKNTFLDLLSSDPELKYYETLQEEALN
NLYREERPGRPSSSREVAGGWLAAPVVGIHNDERFYPEPEKYEPFRFAKAATETPSNRETDDTQTAK
AAQNRKLQGLSTASGSYLAFGYGRHSCNHWRSLPPTSPGRWFVAHQLILLLAYVALNYDIQPMEK
RPLNPLSHRKQLQLACGGARGREIRRRKPEICEWLLPLGRTLALGDL*

>CYP51164A2|3614|Clagr3
MLDTFNISFSFSKSAVGGLALGCLLAWYAGRLTYNVYFHPLSKFPGPKLAAASRWYEGYFDNLVGQ
GGQYIYEIDRPIVRIGPNELHVKEIEFRDTLYALGVKRSKVPYMTDMFGTPLALIGTEDHELHKSRRM
VLNPFFSKRSVTRLESVIQEKVRRLSDNMSACKGRGQDVQLHHALTATMVDITTEYSFAQCANALD
QPDFSPEWAQMMSGVSEIVPLARQFPGIFQVAQALPISITKVLNPLMAKFLKYKLLIRRQISEIIARYSS
SQGEFSASGTEKPAHAQSIFHEVLNSKAPDHEKTVDRMTQEASNIVAAASETTSSVLATTIFHVLDN
TQIYDQLKAELNEAMEDPKSLIEWRRLEQLPYLVSLFYFLLSHGTD*

>CYP5696B1|222|Clagr3
MSVLIFMLAGVAGIILLQPFIAYARDPHRLRRFTSASSIAAFTPLWLMYHNYFGKRYIAVDKAHEKL
GPIVRIAPNHISFAEPAAYKEIYGHSTPAIKDEWYDNLAAGNPSMATATDKTVHSQKRRNLSNVFS
PKEVVVMEPRINILTHKLLHALKKKSQGEMLSSADRYAVVNGAFDLRPWINMFSFDAMGSMCWSS
DFGFLEKGDDSCFARTAEGSVIPVHAMETFHVGVAFTTLLAHLPLQWYKLGRRVFKKTWSGQCAE
HFGSMTRFKVMERMSTPPAGRDLFSHFPVQATEKRPVPMQMPEIIVENQVMLSAGSDTTQSALTYT
MFLLAANPDKQRALREILEKTLPAEEVQGLVASYNCLKSIPYLKACLDESMRLYAPIGFGLPRRTLG
KGATVGGHHIPGGVTISAPVYTIQRNEKLFHDASEYIPERWLPEDPDTTEEERRNLKDYYLPFSLGGR
ACIGRNLAYMQMSLVVSAMVLAFDWELAKPGFEIVPEERFIFNSGPLPVKAKPRVEIV*
>CYP61A7|742|Clagr3
MDTKASSFASPSASVRDGDLLDVEYAVNVGNANLLTITFTILAVLIAYDQIRYWLNKGNIVGPAFKE
PFIGPFLQSVNPKFEEYYAKWKSGPLSCVSVFHKYVFLGMLVHSKGRALTNFDYRFVVIAATRDMA
RKVFNSPAYVKPCVVDVAHKLLGADNWVFLDGPAHIAFRKGLNGLFTRKALQSYLPGQEEVYRRY
LRKFLKVTEDAGGQAVPFMPEFREVMCAVSLRTFCGTYISDEAIKKIADDYYLITAALELVNFPIILPF
TKTWYGKKSSDNVLAEFSKASAKSKARMAVGGEPTCIMDAWIKNSFASKKWLEAEANGLCTENF
GEKPTVLSREFTDYEIAQTVFTFLFASQDATSSAATWLFQTMAQRPDVLDRVRKENYDIRKGDIHA
AVDLDQLESTTYTRAVIRELLRYRPPVLMVPYAVKKAFPITDTYTVPKGAMVVPTTYMALQDPEVY
PHPEIFDPERYYTGDAETKGAKNFLVFGTGPHVCIGQHYAQLNLALFLGKASLLLDWTHHATPQSE
EIKVFATIFPMVR*
>CYP51F18|758|Clagr3
MLYLITSPIKMGFYVLNIIAAVVSLMTIVVLTTVLQQVLFKNSNEPPLVFHWLPVIGSTVTYGIDPFKF
FFDCQKKYGDVYTFVLLGRKVTVYLGSKGNQFILNGKIKDVNAEEIYSVLTTPVFGKDVIYDVPNAK
FMEQKKVPDPILRQHSMPVFTPIQFIKFGLTSDALRAHVPLMEDEIRSFLKRSPHLKGEKGTVQIAKII
AQITIFTASRSLQGEEVRRQLDTTFADLYKALDDGFQPINFMLPWFPLPANRKRNIAQRKMTQIYSD
IINARRAKGGHKDSEDMIWNLMGSVYKDGTPVPDNEIAHMMIALLMAGQHSTSVTSSWIMLHLA
ANPEIMKELYEEQLRVLGPTSIPLTYENIQKLPLVTNVIRETLRLHPPIHSIMRKVKNPLRIEGTSWIVP
PSHVLLAAPATMGKSDEFFPNAAVWDPHRWEGMADPAEQEKEKMDYGYGLVSTGADSTYLPFG
AGRHRCIGEQFAYVQLTVIVIMMVREFRFKSVGGKKEVVATDYSSMFSRPMEPAFVEWERRVDAP*
>CYP5519A2|1415|Clagr3
MTHDALHGLMMDISPSIMVTASVGLLMILGILTLRSVSSDIILPWVPQLKGVPILGAMPIYLKHGMP
QLLSKLIAIGDNGISYAHVVNNVLVSVHDPAMVREVLAYPEHIASREGDPGRMSWSPFWTLRRLIG
NSLFTDVGPEISHHRNVFIREFNSTKSNSEKFDTIAKIATAHANALTGDVSTNEVEDIRYSADNFAIA
LWGDILYGNPNHYVGGRVLSLSETLITLAGNPWPSVWYSFLLFLKLVNPGEPTYSEAKLRAKVAKV
VENNIGKLEEYEQNNPDAPLKTIRNLSVMTGGGITGPLSKFAFEFTNLNLFGGHHSIGLNVTWSLIE
LDKHPRCLAKLMAEINAVDTTDFTNVNSKMPYLNAVIMEINRLHPTVHATLRVINREVKLASSKRP
VVLKPGMLIYLSYWHLHTSREFWGPDAGVFVPERFLGGYRKDQPFMSFGYGPRNCVGYKFATLAA
KQYLITLLKTYQVDVKDHDHEMKLGTLLETTKPVAVKVTRRS*
>CYP52BA1|1521|Clagr3
MAWLSFGFFLGAAVLLISLNLVSSLIARRRLNIEASRQGCEPPYALPSKDFFGLARLLGLLKATREER
GPQYLLDTINAEAGEDVHTFHVHIFPRAQTLMTRDPENVKAIFVTHASSFEINAHRSGIFRPLLGDGI
FTSRGEAWRHSRALLRPQFSREQLSDLTLEQRHVDTLLSALPTGPDGWTDTVDLQPHFFRMTLEAM
TEFLYGHSPSQLDADPSAPSTAVFGHHMDAGKTYINTRLAVGKYHWLIHPRAFTNHCNQVNAYV
DYFIQQKFQHRWWKQSPAPLDNDKPKKFVLLDELAKQTSNAHVLRAETLNVLSAGRDTTASLLSF
LFYFLARHPATFSRLRSTILSTFGSNPHDITFPQLKNCTYLHHCINEALRMTPILPIMERVSLEDTTLPR
GGGKDGQKPIFIPKGQSVLISIYALQQRKDIWGPDADVFRPERWENRRPGMEFIPFGGGPRKCIGQQ
FALTSASYTMVRLLQRFDKCENMEMPAGAGIRFQHGISIRSGTGAVVRLREARPTVVDENVGDGEQ
EKV*
>CYP511E1|2336|Clagr3
MSHLRSFTCAAILSLLTIIIACIRRVYFHPLTKFPGPRTASFTAWHGFYFDVLKGGIGVKRFSSLHQRY
GPIVRVAPDLLHVDDPDFFREVHGPHGTYVKSKFFYRTLGAPSTLLNLQDPHQHKEKVEKAVCIIE
QHQFEDEPLDIQTLYRFITADTLMESMFGYSQELLSHLRNRKGEPPLFACIDGFLSHIHWMTNFPLL

ARLAQNLPAAIARKILPGYVGYREDCARWIQKIADRRSNGVYTDSHGRTVMLDLLLEDNLNKGH
HRLSLEQLIDEALIFLTAGTDSTSYSLSAATFYVLHTPVVLQRLQEELCSVPRGERGRIEWRSVQNLPY
MTAIIKETLRLANPTTSVLPRVVPRSGAHVQGQFIPGGTVVLTITLSIHQNAKLFLSPESFIPERWLGE
KGRELEKWFVPFSKGSRQCIDLNLAYMELYLTFANFFGRFDMQLLETDERCMEWIDNGVAVNASH
VKVKTKSTVL*
>CYP6308D1|2782|Clagr3
MASEAGWLGIHQYINQSIFSMPNPDLKTAYFEASNHVCSILDGQKIFPPSAGVLPATGWLIATRPITA
LVWCLSSLWFVAITWKTVYNLFLSPLRNIPGPPLAKITSKWLTFNELAGNRSLVVAQAHEKYGPIIRL
APDELSFADRSCIKELYLRGSKFPKSPRYDGFASGTRASFDMTDIDQHRERRNLVRHVLAQSNIDEC
ETLIADQVRKALLWIPRVKGQSVEVMLWLRRLMLDTAGGLFLGRSFDALENYEPPSFLGDLDDFFA
ITALRWFAPWLLTILSWLPSTSVQHFLGAQRRGYQYGRKAFNDYIDQYGRHSGRIDLLTKMVGSED
YAPMTDAEIADELGSLLVGATDTTVVVATWLLWELAQRPEWQMRIRQELRDNKVNFIEGVPKYKS
IASLPVLNGFVMESMRMHPAQSIGLPRVARNDGESIGGIKVPAGTMVSIQSRVVQRDPEIYPSPNDF
LPERWIETSGGTKEMKDSFIIFSKGSRSCLGQYVAQMELKFFVSAFVNGWNLRLGKETTKDTMMQT
DYFLAFPKARECHIIFEKYLEPGGTAMKQTSGLITSYD*
>CYP532X1|3083|Clagr3
MLHDAYWLAFGLVLAIVTVRLFATRYLSSLRKFNGPSEFYPINSATTKGRRNNTIFSAPNKLYHDNL
RKAVGSAYSVTSVLRYELFVDPTIGLFLKTLNQRFANRIGAEGVIDFAAWFQYFAFDTIEELTYGVKD
GGLTESGRNVNGLLGYIQSFLGYGYVARQMPVVDKIFKQNPISVSLSAGGWFLGKDFPGIPFAIKRIQ
GKQERLKTISPEVADSATRESLLDGFLRAKQEHPETVTDREVSITLTAFFYYLLKNPECYSRLQREVDE
NFPAFGVKALENPIDYTKAKTLPYLHVCLQEAFRIHPASGFNNERVVDPEGATICGERIPGGTIVSCS
SRIIHRNKDIFGEDVDSHRPERWLGDGEKVKEMNRAMFQFAQGNFSCIGKNISIMEMIKVIPAIIRAF
HLSLGDPDMELKVFNGQNVRILNLNVRAERRWE*
>CYP51198A1|3212|Clagr3
MSFDADVHWTATAGRAFVSVVACWTLYSVAVYVYRLTLHPLAGYPGPKFAAASYLYEFWYDVVL
DARYTEEIAKLHERYGRHPDASDPKFIDVLYPVAGNRIRNKTKHQMSGFGGLSLRVDRLVYRRHDR
TCSPPHASFTAQQFFSKSKVLRADDIVHQRSRQMCDKLLAQCGRNWFDAGGAFNCFTTDAIVEYC
YGESPGFLDQEGWEPNCKDVFEALETRSHFTRHLPWVRRMLHWVPLSIMRTISSTYDAPLRYHVQ
MPGLLKRVIAVGGTGEAESRPVFAELLKSDDLPPQEKTPKRLAYDSNGAALAGPQSTATTLSNIVY
HLVKQPDIAKRLQEEIMANVPDPAELPSWPTLEKLPYLSAVILEGLRLIFDFSQERLSYEPSQERLARV
AVDEDLQYEDHGGMENSSLPTKTVYTIPRGTAVGMSACLVHLDESIFPEPNEFRPERWLDDAGQR
HSRLDRYLLSFSKGSRFCVGFQMAYCVLYVSVAELALRVLPRLEVRDRSRYGDEQVYNDHGEREPR
KTTKGYDMRVVDI*
>CYP51187A2|3214|Clagr3
MGLQSLGLTLLFTFLAYGVYIVFYRLYLHPLANFPGPRLAAVSTLWRAYHQVWRDGKLLHTTKGL
HKRYGPVIRISPNELDFCDPAAFFDIYNNTFNGIKDPDFYNHLRVESDTMLIVDGDPADHKARFRPV
ATLFSARQYETHEASIVAKVEQFSHLVSLSAKRGRVCSLAAGYRSLTIGIMYDFIFTDVPDHFKALRG
PNFDDPLTVASADSLNWTSWLIRNFPMFSAMVMKLSPAMVSRVTSSFEGATQIVEYEKNTKGHKN
PDCVAQRLLNEHIQDRPAVASATLDSVVRSEAIGFMLAGTVDPPNILSLSTFMVARDLALQARLYQ
ELKSSWPNMQSPMPSFKVLRSLPLLRGIVQEGIRLTHGVATGPSRLVGKGGARVADYDVPAKAVVA
APSYFVHMDPEVFPNPEKFEPDRWAVDDRSNSLVAFSRGRRMCPAEQ*
>CYP544C3|3666|Clagr3
MYAIALAVTLCIGLWFLQGQRSAIRRHGQRLPRPPGTLPLAGNGIWFLQPRHKLLDWFVQCERLV
GFSTYEISVPSLPPGIVINDPQNVEHVLKNNDVFIKGEFVRTRSWDLFGNGIINADGDLWKIQRKAG
LRFFSTPNLKTLIDVVLPPIVADTERLLDAAAKNATLVDLQSVLLELTTRLMGNMAYDMDMPASLP
FSKAFDFASGAIGERFQNPFYKLKEFLFGAPLRNSIHEVKTFGSCIVSAAVQKRKEHIDAGSVDPLQN
NLINSLLDNIEDHDIVADAAMNYLSAGRDTTAQSLTWTLYLLMRHPSVQYRIISELRNTLATASHSL
HLSFESVQLSVLPYTSAVFNEVLRLYPPVPIEIKECTAATTFPDGTWLPKGAVVMWANWALGRSKKI
WGDDADDFRPERWLVSGKGAGSTLKSVSSYEYPVFNAGPRLCVGKKMAELLATYVIADLIWKYEF
KEFFGKLSNKGERPIERKSRNSLTLPMEDGLLCYVRRRGEGSVAI*
>CYP541A11|3770|Clagr3

MTTPIPQPPGVPLVGNIFDVDPSNTWVSLQKLWEKYGEIFKITVLGQQIVFVGSVALCEELCDEKRFR
KYVGGPIVEIRNAVHASLFTAFETEAIWGVHHRVLAPCVTPEAVGGSFTEVRDCAYELTAKWRALS
GTPVSAISELNRLDIETTTLCFYGRKLNNITGPESPMIKWMNDSTSEAMKRPTRPKFLNSLFYQSKFE
KACKAMRKYAAECLEYRKANPTDRKDMLYTMMHAKDPETGQSLNDQQIIDEIVTMPIGSSTAPCV
ISSAIYYLLKNPDCISKAREEIDSVIGNSEFTHDHLESLPYCAGIVRETLRLSAAAPGFNIEPLPETKGPV
LLGGGKYEVPAKQTMIIVLHGVNRDPNIFEEPEAFRPERMVGEAFEKLPEGAKKWFGNGKRVCYG
KNHAWMWNMITLVALLKDVDFEMADPSYQLKQDGWFNLRPVGFNVKVKARAQ*
>CYP50334B1|4140|Clagr3
MPIELLAAPLSSSNLITISMLIFIVSFTIALLVAYVLWNPHACRACTIPMLQNKFSNPKACREILACHG
YASGDRNTIPAVESRAGPNQRLVKAFNIDNAFTTKNDEDRKSFTSKARAKLSAVKEADWKRIASH
AEALIHHGLRGKQCCLDSLVRSTSLKITLHTMFKLDPMEMKDETIGEITSSINDLWVESKKPGEPSAP
MKNKLRQALAGLFPGAEFNGEENPLNFILPAYETLWRVVLSGFIEVTFREGAHPGWKYELVRFIEEP
TIDKLKSTSYDTSVSMESITNEALRLFPSTKSVYREFRMDTKKTTDVVIADIEKCHRIPSIWGANAKTF
DPSRWKNLEAQTKEAKEAKEAFMAFGYGKFTCPAKPVYGPMIIAVLVAALAKHITAEQWTLELYR
AGSEAGYELRGSEAFIADRKSYEKIMIHRKETAWAGAGRYTVGSQ*
>CYP59U3|4315|Clagr3
MATKDYARKSQHTDAGYYASRICNRYLAYLYVKQPPHLCIYSESGLCTSTQSIEKRLRFIACNLRMP
SLGLTETGRKVMTPGNLTASLLLIGASLLAWLVTSFYPSRKHIAELRTRGLPVAPHWNWISGNLILV
AKMFPSMPRKAHKTLLFGDILRNFFAQEGCCYLDLWPFSYPLLMVVSPSLASQATQTCTAVACERP
PKLEWFFKPLAGGPNLFDLPEKDWKPWRTIFNKGFNADNLNSFVPGMVEETRKYRATLTKFAESG
ELFQLDSVTLRFTIDFIGRTVLNVSLGAQSHYNVLADSMLSQTSWQMPNREINPLEFLNLPRYVMQ
WWNGRKMDKYIGAELDKRFQDYQEKQIRPNSASVIDLVLKAYLAGSAGNPDDAKTNKLDPRFRA
FATAQIRLFLFVGHDSMSSTICYVVHLLSLNANTLKKIRTEHDTVFGTDVAALPATIKARPQVLGLLP
YTTAVIKETLRLFPPGSAIRQGSFGADLVSEDGTRYPTKDIMIWILHTAMHRDPQYWVDPDSFLPER
WLVDPGHELYPRKEAWRAFEWGPRNCIGQSLAMTELRITLAMIVREFDFKPGYEEWDRLHPRKGV
QTYRGERAYQIEEAAAHPADNYPCRKCMTQ*
>CYP51189A1|4783|Clagr3
MTRRNSPSGIFALKLFGTKYNTVWTPSLIDAAFNNHHASLNAIEPRWTLLRKVFGANGSGKDEYV
HLYTNDQVCLANAHLSTGSFHIAQTTLHAIQQHAPNLLSFAERPIDQNPWEQNANPVLHDSDIVEI
SLFELISRFVAHVSLPTLVGTEFLEIHPITVEDLWDLDGGFKWLKWGFSRLWGIPSLTRAHIARRRLL
AALQSFHKAADAVSNGDEAKEPWREIGDLTDLMKERRTVWKRFQTSLDVRAACDLNLLWAMST
KPACIMFWMLLHIHTDPTLLTQLRAEMAPFVLITRPPQILSIPSPPRLSIDLPALVTSCPLLKSCLYETL
RLYTTPSPTLFVAKDTTLHDPEGKVRSHQDYILDKETYVTAPLTLHHHNPAYFWQPHEFQPRRFLK
ANENDPLTEHPTHLNKNHTTNPPKHSTTPPPSTPSASAPPPVQPAPTQKQKS*

>CYP51F17|4971|Clagr3
MALAALLLEFAALILLSLSLYRLSQYLFKRKDEPSVVPHWLPFVGSAITYGQDPYAFFFRCREKYGDV
FSFLLLGKKMTVCLGTQGNDFILNGRLQDVNAEEIYANLTTPVFGSGVVYDCSNAVLMEQKKFLKF
GVTAEALQSYVPLFEAEFNDFVTHSEALQGIEGSFSVTRVMAELTIYMASRTLQGKEVRQKFNSNFA
SLYHDLDMGFTPINFMMPWLPLPVNKRRDLAQRTLARTYIQIIEARRKLEKGQAMENDMLWHLM
SSRYKNGNPIPDQEIAHMMIALLMAGQHSSSSIASWIMLRLASRPDIAEELYQEQNRLLGPCERGMR
RPLTQENISLLTLNSLVVKETLRLHAPIHSIMRAVKSPLSIRTNRADGLEERVYHIPTSHVLLSAPGVT
AQSAEHFENPSYWDPHRWERTAVKEGDEKVDYGYGLVTKGASSPYLPFGAGRHRCIGEQFAYLQL
GTITALMVRDFKLSLMDGETAIPGTNYTSLFTRPLDPAMIRWERR*
>CYP61A1|5505|Clagr3
MASLVNGSFASPLADANMRIVTEGGWLAKVYNGMSGLNVLISFLLVLVAYDQCKLQDANGCGVR
LLMWEIVMYVWNKGSIVGPAWKMPFIGPFLSSVNPKMDEYKAKWASGELSCVSVFHKYGWPCNG
FGWGCQLTAMARFVVIASSRDMARKVFNSPGFVKPCVVDVAYKLLRPTNWVFLDGRAHVDYRKG
LNGLFTRQALDTYLPGQEEVYDKYFEKFLEQSRKGDQEKGVPFMYMFRELMCAVSCRTFVGHYMT
EAAVKKIADDYYMITAALELVNFPVIIPFTKTWYGKKAADMVLNEFAKCAAKSKARMAAGGDITC
IMDAWVKHMLDSAKWNEKVAKGLPVDPSEKPNPLIRDFSDYEISQTVFTFLFASQDATSSACTWLY

QIMADRPEILDRVRAENLAARNGDKNARFSMDLLEGLTYTRAVVKELLRYRPPVIMVPYIVKKPFP
VSETYTAPKGAMVIPSCWPALHDPEAYPNPDTYDPERWYTGDAETKTKNWLVFGTGPHYCLGQT
YAQMNLIAMIGKASMMLDWKHIATPKSEDIEVFATIFPQDHCPLIFTKRPQC*
>CYP619C8|5770|Clagr3

MTKWSKKYGGIFSLKRYKNTTVVISDWKYIKYLLDKKSTLYSSRPKSLVVDLITHGDHILMMQYGPT
WRTMRKLIHQTFMESRCDKEHWKVQEAEANQMIYDFLTRPKDNMLHPKRFSNSIIMSLVFGIRTKS
IDDEYMKRLYHLMDKWSLALETGATPPIDSFPLLKLIPQRFMGNWRGRAVEVGDLMTSLYTEVLQR
VYARRNANIKKDSLMDRVLDQQEKNRFNEHQLAFFGGTLMEGGSDTSSSLILAIIQAMTQYPEVQK
RAHAEIDATIGNDRSPQWSDFSKLPYINMIIKEAHRWRPVLPLGVVHSVSEDDHVDGMHLPKDSTII
LNVWALHNDEDRWKSPEAFIPERYEDFPELSPVYASSDKRDHLGYGASRRICPGIHLAERNLFIATA
KLLWAFDFSQDASLVNDTSAETGSSQGFMHCVKDYGCRIDLRSEAKRETIERERAEAEEVFNRYD*
>CYP51201A1|6448|Clagr3
MAGFEVIVICLTLLILYYVSTLIAYGLWDLPGPALAKVSNLWRFIATWQGHLELVLRELHRKHGKLV
QIGPNVVSIADPDAIETIYGIKADLPKGDFNKPMHNYRNGKLIPTLLTATDQGIHRRLRTAVAHIFS
MSTLVQLEPGIDETILDLIQQLAHRSAEPHRSCDIHNWLRYFGFDAIGRISFSRPFGFIQQSHDIDYML
TNQDKNSKYLAVVGQMPWLDHLLRKNPLFPLLPSFLNPFSAAVPVIALAGQIFGQRMADEKTGKG
SDYLDMTSRILAIKRANPDQIPDQAVIGYNLTFLQAGSDTVSIALRTIIYYLSKNPPMQRKLQAEIDA
AKLTYPVTWKAAQHLIYLDAVIKEALRIHSPAGVPLERVVPPTGLNLPNGRYLKPGTIASMTPWTLS
VDEDVFGKDATKFNPDRWLKGPDESEDVFHERLRKMKRADLVWGHGPRSCIGKPIAQLEMYKLV
PTLFGLFDMRLANPEMEWRYGTYIITGQCDMDVVISWREGVDRSVLRR*
>CYP5080Z1|6778|Clagr3
MSTNSSITSLWGESSFPTSKLADIFHFRKTVNPAYTILQGPQTLSTLMRSPPDLSSTLELLRKIPHSFSFY
IVLLLIPLFLHSFYKAYATPLRHVPGPWIAKFTRFWLLGAINSREYQRINVELHRKYGPVVRISPNEYS
IDDPDAATIIYRSRDQLVKAPRYSSWGLPNTEANIFTSRSIEHNAARRRQIAPLYQLSALTNIEYQVDL
TSAILLSRISAFASQDRPIDLCQWIQYYAFDTIGTLTFGKPLGFLDQGEDIAEVISTIHTYSRYGGVMG
VFNELHPYVFRFLQFIAPKGEVGIAAIYAFAAKRIAEWNCRSENDKALSVEQNEGGEVLKTDYLTSL
LAKHRRTPENFSVDDAFYHIISNVVAGGETTGIALTAAMYLLVKAPRVLEKLRGELEGMKRGKDGR
VGVLEAQNCGYLSAVVKEVLRLFPPAGLNLPRVVPKGGLVLVGRWFPEGTIVGVNPWVAHANTS
VFGLDADDFRPERWLEADQAFANRMEQYFFTFGRGPRTCLGKSVTLMEMMKIIPELVLKFDFSFAY
PLREWSVRNDWFVKQEDFMVRVRERRTERP*

>CYP5127F1|6860|Clagr3
SGSTYLNISCTQYIIFQSLPLQSYLSAGHLQILLLTPIPLLSRLSSPSHQLRNRTKTLFTMLIDSSLLLRTSS
WTMIPTLLLIGVIFLLFLSLIFSLKEKPVPMPSVFQIVKKYIRAWAYLFNGPSIVQHGFDQSHGKPFEIR
SPDTRMVFVSSPKHIKELDSTSDTILSLNGAAKHVHNPSTPTYLRVLSPLLTLISRNQMLQPLYTMNG
FNWFDQRGVEGVGFVRTLRTLLTNNLPRLLPDLRVLTTTRWSQSLSDRATVSDKGTVYAPVYPIMM
NLVVLLNARSLFGEDLVKNKKFMASALSYVEETLFNAEIVKLLPKFLAPVVGGILSRCLNSHRTFFQS
LIPATQQRIEEQASKNLGYSGPKHADCIQWIIETAPKQSPWSAERVIYELMAIWFGSTIVYVIHDLCL
HPEYIEPIRRELEKSYEEFEHTGYGLPLLDSFIKESARLTPVESMSVRRAALQPFTLSDGTHIGVGEWA
CAPSGAINKSAEYYPSPDQFSGFRFIDAAHLPNAQEGLPHSIRPQQPNPSKLTDVDHSFLMWGTGR
MACPGRYYATAFMKVVVAQLLLQYDFTLMEPDAPRWISWRVARIPRPWTKLAFTPRK*
>CYP617AB1|7119|Clagr3
MDVPWQLITISSLAGSFGLVRLAPQYSPSRYFIATFVLVFLGEIIAAIIWQAILWPRFISPLRHLPEPPGA
SFLNGHFAIIAREPTGHPQRRWFKEVPNDGLIRYRALLNQERILPTTHEAYKEVLSTKSYEFIKPSMT
RNGLARLLGVGVLLAEGEEHKRQRRSLDPAFETRHIRDLHPTFWALSAKLVRALTTWIENERKGTI
TSLSDAPVVEISTWTSRATLDIIGIAGMDKNFDAIANPDSELYATYQKVFQPTSGQQLVGVLSFFLPL
WFVRSLPFAHNETMAGAATTIRKICRELISEKQEKLKLMQKEVQEPRFQGSTDKDILSVAMKSGQFT
TDNLVDQLMTFLAAGHETTATSLTWACYSLCQYPEIQSRLREDVRTHLPSIDDDSETITAALLSQCH
YLHAVCHEVLRFWAIVPITLREAAHDTTIQGQFVPKGTKIIICPWAVNTSVSMWGPDAEKFNPDRW

TAPGQANRGGAESAYANLTFLAGPRSCIGKQFALAEFATLLAALVGKFEFEFEDPEYPAKMKIKGGI
TTRPKEGVSMRMRPVEGW*
>CYP532A34|7337|Clagr3
MMIEEILSSLLRHWPTAILILTIAYLAKNRYHNGLQKYPGPVLASLTDWWRFFDVLGRRPDITHINL
HKKHGDVVRYGPNALSFANPQALKTIYGLNKGFVKSDFYPVQQAMSKGERLPSLFSTTDEQYHAE
LRRCVNSAFSMSSLVQYEPSVDITTAKFLDQTEALFTSQNKVCDFAEWLQYYAFDVIGEITYSKRHG
FIDSGKDVDGMVGYLGKLFSYVAPVGQLPLLDRLLLKNPILRFLDKHNIMSFTFPVVTFAKARMAE
RLSEIEHQKSKGNTAAANEPIAGNRRGDLLSMFLKAKEERPDFFHDGRVLTMAVSMAFAGSETTAI
SLAAVFYFLCKNPPAYAKLMEELDQAISNGTLSDRVTTTWAESQKLPYLDACIKEAFRLHPAPGLVI
ERVVPKQGVEICGEMIPGGTIVGCNAWVIHRRAEIFGKDVEAFRPERWLEASGEARKRMEGSMLQF
GMGARTCIGKNISLLEIYKLVPSFLRRFEVQLADPTKEWKLHNAWFVKQLDFNTTFKPRRSPATAK
YQ*
>CYP548BG1|7493|Clagr3
MGLLVVSPLGLTGAQLVLVCLILGSCYFLLHAVYNLYFSPLANVPGPFFAKVSGLPSYYHALRGDR
HIWIWQCHQIYGSKVRVNPSAVVFQSAQAFRDIHGFKSNVQRSMKYKFWQRNESAISTFTTTDIAL
HHKKRRILNLAFSEKSIRAAEVFIQKHIDRWDELLPNGDSKDWSQPKNMTEWSDYLVFDILCDLCF
GRSLNIKDPGNKQFKGIPKVIHSYMTFTYPFTRSPFLGLLLWLKPRGLDKLFEIISPSDIKAYFAFIDESI
AARQKDEELSERLSTDGKGGRQDMFHFLFQATDPETGKKGYSLQELFAEANLLVVAGSDTTAITLS
SFFFYIVRNPRAYKRLVKEIRSTFNSADEIAGGPKLSSCKYLRACVDETLRMSPPVPSELTRTVLAGGQ
MVDGELYPAGINVGTSEWSNGRNDEYGDPNVYRPERWIVDGEADVSAGEVARISSYLHPFSAGWG
NCVGQNLAILELLTTIARTLYRLDVRAEPGSTLGEGRPELGWGRRDRKQFQVDDAYVSIRDGPMIQ
FRKRNA*
>CYP6171A2|7645|Clagr3
MPTMIFASLAPVGAQILILSLSFVVCYSIYRAIYNRYFHPLRGFPGPFWGSITDFFKLWILSTKQAHTL
GLKYHAEYGPVVRAAPNLLAINDPLLLPQIYHRRVDKTDVYTPGVLGELAPPFQTIRHDEHAAKRK
RVASSVGQKFTLTNLKPLEGQIDARIMQWTSVINDRFAESGQKLDFAAWSQWFAYDTICQLSFGEP
LGFVKEGRDVEDLIGNFHKMAPFAAVVGALPWLARPFLENPITRRFAMPKPGDGTGTGKIMAFRD
KLLEQRLSDPKSHQHGDFLDNILASKNADGSSITVNEVKTECFVLMVAASDTTAAFFCGFVRFVIET
PGVYEKLVAEIDDFDKRGMLAHPVPLYEEIMKLPYFKACHLEVTRFYPSTPMIIPRYVSDGGMDLYG
MHAPAGTEIGANPYVVQRDKGVFGEDACDFRPERWLESVEKANMMEKYILTWGYGTRICLGKNI
ALMETYKLMAQ
>CYP5168J1|8086|Clagr3
MAAIFGASTGLASHLLFYIRGEHHMYAHVLFYVYITLAVGIFQYHLYGLRYEVSKASTSTAVIILSYL
AALFASIIIYRIHFHRLRSFPGPRLAKISKFWHVYQVRHSMNHRLLERMHSRYGSFVRTGPSELTVFV
PEALSALDGPKNSCTKSAWYDLSQPLKAINTTRSKAEHDWRRIIWDKGFTTHALQDYDLRVLKYG
TQLEGHLSTTSGQPVHVNDWFYWFSFDVMGDLIFAKSFDMLVNERWHHAILMMRDFMWLLGPFS
PVPWLALVGFGIPGIAQDWKNWVGWCKGIMAERIEAEVEKPDVSSWLIKASKEQDRLEKDRIWLE
GDAITMIVAGSDTVAITLTFAFYHLASCRRYQSRLRDELLSVPSFTDIEVLLKLPILQSIISETLRLHPPV
PTGGLRDTGPQGLTLEGQFIPPFTTVVVPRYNVARLERAFTRPEEFLPERWTSRPEMIKDRRAFAPFS
QGRYGCVGKNLALREISYLIALIVTKFDVTFAPGEDGIRVWKDMKDEFTAAPGRLMLVFNKRQKT*
>CYP53A63|8118|Clagr3
MLLDSLLTPYTLLLIPLIYYLLPYLRNHSIRDIPGPSLAAFSNLWLLYQCRRGRRYLAVDEAHKKYGP
LVRIQPDHVSIADPSAIPVIYSHTGGWLKSDYYDAFVSIQRGLFNTRDRAEHTRKRKTVSHTFSQKSI
GQFEQYMTFNLQELVKQWDRLSETAIASGQPWAEMDCLHWFNYLAFDIIGDLAFGAPFGMLPKG
KDIAEVKMTPDSPTTYAPAIEVLNRRGEVSGTLGCYPAIKPYAKYLPDSFFRDGVRAVQNLAGIAVA
RVNARLDSPDEEKGGRKDLLARLMEGRDENGNKLGREELTAEALTQLIAGSDTTSNTSCAWLYWT
LKTPGVMERLQRELDAAIPEGVEIPDYDMVKDLPYLRNVISETLRIHSTSSLGLPRVVPPGPGVTLLS
HHFPPGTILSVPAYTIHHSPSIWNPTSSPAFSPNVFNPDRWNEGTLTQLQKDSFIPFSTGPRACVGRN
VAEMELGLIVASVARRYEFVLREEGELETREGFLRKPVRCLVGMRRRRQSG*
>CYP5574D1|8153|Clagr3

MALGTAFLVGFVIAIVVLLLRQLVSEWDAPWMDRLPPYLREKILMIAYLGHGPKMIERQYRKFGQK
PFVLQTPGKQIFVVSSRQHIKELSQASATALSMQSVAKEILQPMHTMQGFDVKDQRGVNGYHFLLA
IRKEFTSQLPTTFSRLEKAVSDEFSKELSDCTVVEGKHQVPVFHLAHRLIARANLTACFGADLSSDPE
LVKAAMQFVMDCFVAGEIIQAGPKRFSLPLAYLVTRRYKACRVLADALVELVERRREVASRDRTA
QKPADGLQIMMDACPKDQDWSATQIAHEALAMFFFSVLPLTNTTAFTLVDLCSHAEYVTPLRQEL
TRGWSEFGHHVEGLPLLDSFIKESSRLNGTEWVTSRRKALTPFIFSDGLYVPRGSWIYIPTGSINRDAK
LFNDPLQFDGFRFAQEKPSRFTDVSRDWLIWGTGHVICPGRFYASLILKLVLAHTLRNYQCELRDPG
KPQTKMWRSGIRPRKDLTILVSAREKQDEDLGLKL*
>CYP573A25|8435|Clagr3
MDAVNFVFKILSAITLGRLCLTLLSIILLSFSYQVIYYRFFHPLSIFPGPFWASVTRLYSAYFNFVGRHYL
HEWELHQKYGPVVRLSPTELVVSDATFLPKIYNRYADKSQHYITGSFGKIESVFNMQKHKQHAHH
RKIVAGPYSFTNIKGMEPLIDARIQAWISKLSDEFAKTGQKFDFAPWAVYMTYDVISDIGFGGPFGFI
EKGEDVGGLIKGFRDGLPLFGVMAILYPFTSWVKSTWMGTKYLVAKPEDASGIGTLMRFRDRLLSE
RISENENDKDRERVDLLQTFLDARDGSGEPLDIAWIKAEILLVLIAGADTTGTAFQGLMCMVLNDP
AIYSKLMDEIDGATRAGHLSAMPQFDEVTQHCPYYVACIKETLRVWPSAPADFPRLVGKGGMDLF
GKFAPEGTEIACNSWVVQRDSNIYGEDAMEFCPERWLDKEEIVTEYTRYNMSFGYGSRICLGMNVA
LIELYKGPLQLLRTFRPQLLKTEKPAKFVVLGGVAHWEDLWLKIENRAGVV*
>CYP6259B2|8703|Clagr3
MAHLSGLTGALIVVYILSILCRRSSQHLRFYKAARQHGCKPARRYPNWDPFLGTDLFVRFRKADYR
GNRSQAFVELHKRYGRTIEMKAFGPAYILTTEPLNIQAIAATKFNDFGVGPRRGNVGAPFLERGVFT
EDGDFWKHSRALIRPTFSRNEIADLGNFERHVGRFLAQIPRDSRTFDLQPLAKRLFLDTSTEFLFGQS
VESLLPQTPFDAAQFMKAFDYSLLGLALRLMAGPLRFLFALDPTWKKAYTKVHDFVDKNVAIALD
RQRSLVETGKPANNGGHRRYILLHQMAMETQDPYDLRSQILNVFFPARDTAAIAFSNIMFYLARYP
QVWADLRKEVLQIGSQDLTFELLKTLETTRAVINETLRLRMPASRVVRTALRDTILPAGGGSDGRSP
LFVPEGQNVEMDLYSLQRDPDIWGPDAEQFRPERWTKGRPLWEANWQYEPFLGGLRMCPAQNQ
VITQLSYLLVRVAQNFQAVKNRDSVWEYEENIKMTIESRNGVKIALVPM*

>CYP6677A2|8830|Clagr3
MNWTIALASILVAFLGWTTVSIISNYIAARKVGLPLVISPVYPMNPFWIITYKLFPIILLLKNLPFGLGT
WARCTYIGWQFHEKHDLHDELGPMFLIVTPGGNEVVVADAQVAHTVLSQRKEFIKPPIMYDQLN
VFGPNLDTVEGEDWQRHRRLTAPSFNERTSSLVWDEAIRQTKEMAPSWTDQGRMGTSETVPDTAT
IALHVLTSVGFGHSYPFHGGVRDVPAGHAMTYKDALALCLGNIITFSIFSRKTLSRSWLPKKLRALG
VAAKEFQTYMEEMLSYQRAGVEERENESPNLMSALVRASEEAQKTEDESGSRRVGLSDDEIFGNIFI
YNLAGHESTANTIAASLVLLAAHPECQEWLAEEIHQVLGIAKATEGWKYEVVYPKLQRCLAVMYE
TLRLYGSIVFIPKSTSFHSQTLTLKDRTHVLPPDTFVIINVEGLHTDPKIWGPDALVWRPSRWLRPAQ
SEKTTNPRMTPESFIEPDPGAFIPWADGPRSCVGRKFSQVEFVAVIATLFSQYRVRPVLRPGESEADG
QRELQSMVDNSAISAITLQMREPKKRALRWEKRTLNG*
>CYP611C1|8987|Clagr3
MHLIPFLAVAIFLALLYAFSRFQESKHDPKEPPFLPQKIPFIGHIIGMIQKKTRYYVHLRQRYAAPIYSL
LMPQGSKIYVVTSTDLISAVQRQPRILSFPPIGVKFAMSLSGSSASANKIATNNINGDEGDWGLSMDF
NKAIYPALSPGPHLEAMNRIMIPHIAESLDKLRMGEEGGRRRRIVLGRWIRHELSRAITDSVYGPQN
PFKDRAVEDAFWDYENELFRIMMNLLPSLTARKGMRGRKLLGSAFLKYFQNGGLKDASVFAQNRF
DSWNNHELALEDIARFEVVTLIGTLTSTIRTVTWMLYHIYSTPSILAELREEIPAVMSTSTTPQGVPFRS
LDITRLRANCPILGSTFQEVLRCHALGTAVRQVMRDTLLENKYLLKKDSMVLMPSVVLHTDPNLW
GPDVGAFNHKRFLRQGPSTSKSGNYKTPSPSAFRGFGGGTTLCPGRHFATTTTMAVTIMFLMRYDV
VPVEGRWPKMTGEKTGVVSAVEQPDHEVEVDVKARKGFEEGEWSFRMDNSDTVFAMAVEDFE*
>CYP6406B1|8989|Clagr3
MVEATANWQHVPYMLYDELRYALGPENVTTKVRQWQASTPNPPILQLASQGHWGMMLGASLLP
TFLLTYVTTFLRSSIAIKRQKVGRQPPIMPYWIPFIGNLIPYIWDTSGFCTKTTKTYGYSMPIRVRIGPLK
LYLVSGADHYLALFSSKASRCMNTKAAVLLALENLFGTPSNVLPFYAADNSGVNSTPVPWSRVKPE

HRITYLQTRAAHKYLSGPGLAHMTEFYLNVLRQGFQRSQIGSEWIDHPDLYKFLQDTVFRAAVEA
MCGQHLLAQSPTFVDDFWEFVDSVPILIKGLPRWLSPRPYRVRGRLLGAIKRWHNLAHEKSDSSKI
GADDPEWDPYFGSKLIRARQEYSRNMPSLNAEALAAEDLGLLFATNTNAIPSLTWFIYEICRDNDLL
ARVEKDVEACRTRSSSTDEMSLDIDKLCSQPLLQSIYAETLRLRVALFVTRTPENESFSLGDWKLPPN
ETIVLSSRTGAMNPDVWNAGTPINPHPLDTFWADRFLIYPNDPLSGPSRKDLISFDENHGGSTTEER
PLETEPRFSLEGVAGGWIPYGGGQRMCPGRHFAKQEIIGTLALLLTHYEIELREPTKGAPECNMRFF
PFGGLPPTKEIPFRMRRRNI*
>CYP51F19|8991|Clagr3
MDVFIGERLGLSKFALAMSAITLLFTFFILMNGLKQALFKKRNEPPVVFHWLPFVGSALSYGQEPNK
FLGECKAKYGDIFTFILFGRKITAYLGTTGNNFILNGKQVDLNPEEVYNVITAPVWGSDVVYDAPH
AKYMEQKRFIKFGLTSTALRSYVPLIEDELYSYFKRTPAFQGSAGIVDIPHTMAEIIIFTASRSLQGNEV
RKKLDSSFAENYHDLDMGFTPINFLFPGLPLSRNRKRDIAQQRMANTYMEIIRARRENPYAEKSED
MIWNLMRCTYKNGHPLPDKEIAHLMIALLMAGQASSAVTSAWIMLHLAAEPSIAAELYLEQIRVL
GSVNKPLCLDDVQKLPLHADVVRETLRLHPPVNGVMRKVKRPMPIDGTDMYIPASNILLAAIGHIG
RSAEHYVHPERWDPHRWDTNPYMDDENKSVKVDYGYGLVSASATTPYLPFGAGRHRCIGEQFTY
LQMVTIVAIMVREFEFRNLEGQKGIVATDFSSMVARPMAPAKIRWQRRSKVATDFTDRIHTGPT*
>CYP6760B1|8996|Clagr3
MIVEIWQFLQHVGSDKNGNIDYLRLTIPTLALGWLSWRIWAFTVKPTFQPREPRELPYWIPSHAISFF
KDTEGTITRARLYFGDSREPFAITLAGGKLYIVTAPQDVTEIYKNTKTLTFDEYVRDVMKSIGVSDDGI
AKLYKPPGEIGDGRLHKALAHAGEDYYREQLLPGAQLDILWEHVLSDIASSIRWDNLPGSRFNRAE
NSKTLSLLEWTSDVLLKSVIRAFFGDRLLQMDPQLLQNFAAFDKESWKLTYKLPRGVAKDTYDAM
DKMVRSVEAYLQLPQNQRSEATWLIKKLEVEAGKANIDVHDLAAMITSLVWVILSNAYKLCFWIIA
YLLYDPSLYAVIKAEVTVAVRDGLPGLETKLEECPRLAAVYDEVLRLTASSASIRTVESDTPLRNVVL
SAGGKVLMPFRQLHFNKEVFGSNVDEFDAERFLRDKSLGKSSSFRPFGGGATYCPGRYVAKREVMV
FIALAMSRFDDIRLAEKEQSAFPKLDTRTPSLGIMMPMAGEDVQVVLCILENQDRKYFGYSDETYKL
TFPGQELYLMTSPGDISLIYRNTVELTFDTFVRQTLRALGASSHAVDKWIPLHLSKEGMAHTSSSSTP
HAGEFTHIGERLCQQQLLPGKQLDILQRIFIDGIHESLQWQSVTQKITIRSSPDIKTISLLGWCREVLL
QSATRAFFGDRLLKIDPDLFQAFFMFDASSWKLHYGYPRVFSKELYCARDAIIEALTAYFKLPRSERS
GAAVLVDSLEAEMRNLDIEDKDIAALIMPVYWVINANAYKQCFWMMAYILYDQRLLSTLRDEISP
AISENSPGLEYRLEQGCPRLRAVYHEVLRLTASSSTVRGIQCATEIGDKVLGAGANILIPYRQLHLNP
EIFGSNADQFDPERFLRNSNLSKNSSFRPFGGGRTYCPGRYLAEREILTFVALALHRFDIRITASKSHD
DEKARNVIPGFPRIDNKKFCLGIMEPIKGDDLILDITRRG*
>CYP6389B1|8997|Clagr3
MTIATQSDLQHLFGQPSALGGLFVVLWLIWRMKVILQKESHASITGLWSWITDVKYLFYYADYLQA
NYVSGVPYTLSTSSNTFVHISSSAQVEELGEVPEGTLSLVALAEDVSLEHSVFGFGMTSFPQMLQPKY
TMDGLEVLEDSVTGMSIQYRVLRTLLTANLPSLQYGLQQKISKSFEDEVSSNSPSPDCWSMLRTFPM
AKNVVRTATSFAFFGEELSNDPVFLDAALLYPESVFKAAEAVRYVPKFLAPTVVSWITENHRASRTL
VDKLTPVIEERTGCRQLRDLPSLKPNDCLQWIIDASPRKDPWPTSKLIQIALGSWWASIHQLSMTLT
NALYDLCEHSEYIDILRNELGQCTMEGRPTDLPLMDSFLKESARLHPADTVSMRRKALSPYTFKDGL
HIPAGSWVCAPSRALMRDSAHYTDPATFDGFRFVDKTTMHNPAGPIASISKYTDTEPKFLFWSHGR
RACPGRFYASHVLKLMLRHILLHYDVKFADKAEPRSFAWRATIVPRENTRLLVRRREAEDLQA*
>CYP548BF1|9839|Clagr3
MTVALYLGVLATLNPVAIIACIGTLVSVALVITYRLTLHPLAKYPGPLLGRLTDWYSVWYIWSGDRH
INFYQIHQKYGDVVRYGPNRISINTAAGLREIYGSKANTQKSSAFYNVFAHFFHSDSTLTIVDRAVH
GRKRRVMSQALSGNMVKAMEDHILKHVNIFCKCMIGQTGGSETKATSGWSPARNMTTWSSRLTF
DVMGDVAFGRTFEMLISETNRYILDILPDGVHGLNLVGHMPGLLKLQLDKILFRKLTDGTRQYEAF
SKAQSDARIKEGTSYKTRDIFASLLEFKDPETGEAFTLPELVSESSLLIVAGSDTLATAITSAFFYLLHYP
ETLTKLNAEVRSTFSNVDQICVGPQLTSCHYLRAILDESMRLSPGVGGVLPREVLPGGLHVEGHDFP
EGTVVATCHYSIHHNEAYYPDPFTFKPSRWITDSDNDTTPSSVELAKSAFCPFSIGPRGCIGKAMAY
AEMSILLARVIWLYDMRLKEGSTLGEGSPALGTGRTRCDEFQLYDKFVSMTDGPLVEFKVRKM*
>CYP65FG1|9921|Clagr3

MSASGGSAHTISLNGFTVAGITTMTLGWSQRILTVAVGAFCVKFFLTVIYNLYFHPLRNFPGPKLAA
ATPIPFAWHLLTGRMVAWHKAMHAKYGMVVRNHPDELSFIGASGWGDIYAARPALPKPITGTLQ
TPSGVPSLIFIPDPQTHGRQRKILNPAFSEHALREQEYILQKYGDLLISRLREQGDEVNIIEWYNYTTF
DVTGDLCFADSFRCLEAGDNHPWVAAVYRGVKFGQILTVFDHFPPLGAIVKWCIPPSIQEKAQEN
HIYTRKKIDQRIASKSDRPDFMKYILENNREGGMTREDINSTVSLLVLAGSETSATTLTSATYYSLNH
PRVFERLKKEIRDAFDHDPSSITVSAVSELPYLNAVITEALRLHPTNPVSIPRQVDRPVEICGIMVPPG
VRAGMSGLATFTSPMNFVEPMSYIPERWLKDADPRFAQDKKDAFAPFLVGPRNCIGKPLAWAEM
KLILAKVILNFDLEISSKNIGAWDDTRVWLLHEKKPFYVKIHSRTQRKK*
>CYP6171A1|10074|Clagr3
MGILSLVFSLFGILFAYASYRGIYNRYFHPLRHFPGPFWASVSDFFKLWVVHTKQIHTLGLEAHRKY
GPIIRVAPNLLTFNDPELLPTVYHRRVDKTDMYTVGALGDLAPPFQTMKHDEHAAKRKRIASSVSR
HIPTKERCTLPVPLIPQKFTLTNLKRLESQVDDGIQEWLGALKKRFADTGEKMDYARWSQYVQTGS
EHIKYLIFHSYFVHDMMTQLSFGEPIGHVKQGRDVDNLINNFHQMTPFAGFIAALPWVVKPLWES
RIGKRLFMPKPGDNTGTGKIMAYRDRLLEERLNHPKSHHKGDFLDNILASKNEDGSPISIEEVKVEG
FVLLVAASDTMASFFAGFMRYIVETPGVYEKMMTEIDDFDRKGLLSTPVPTFDEVREMPYFVACFRE
TLRLYPSTPFIIPRYVSEGGFTLHGKYVPAGAEIGANPYITNRDKNIFGEDADSFRPGRWLENAERER
EMERYFFSWGYGSRGCLGKNIAKLESYKTMVQFFRTFKPSFCDVERVWRQENLAIFVHWDMWMNI
ENRGVHPENQKL*
>CYP532W1|10380|Clagr3
MLPTNVRSSVPVEAWIISLVALAIARLLFYRFKKGFAKYNGPFLASFTDLWRVFHTYTHMNQPPML
DIHQKYGDIVRIGPNTISFGKPEAIKDIYGPGKTWNKSGFYPVQAAVVRGESAITLFSSADIPWHNAL
RRAINPYFTATAAVSYEPLIEKTINVFLAQLDSRFSGKKGPEGIIKLEDWLLYFSFDVIGELTYGSRHGF
LESGYDSQGFIAFLQGFAVYGSVMGPSPFWDRFLRHNPLLLWLERKGYYTGSTTSFGPFVEKRMKER
IMQQKNTKNLETKESEDLLDKFLKARETHPKAVSEKEVMSLSLTMVLAGAETISITLTALFYYLLKN
PSCYTKLQHELDTQLPPRDPSSLKCDVDYTQAQKLPYLHACIQESFRMHPASAVLLERVVPPTGADI
AGEHMAGGTVVGVSSWAVHHNKDVFGQDVDKFRPERWLEPSEAQVRLMERSMLHFGAGNHLC
LGKNISAIEMYKVVPSLMRTFKIELVDPEKDWKIITTIFTRQEDVDVRIERRR*
>CYP51F1|10426|Clagr3
MGVLAFVLEPLRVLSNRSTVLACLAGLFSLIAFAIILNVLNQLIFKNPNEPPLVFHWVPFFGSTVSYGI
DPYKFFFSCRQKYGDVFTFILLGKKTTVCLGVKGNDFILNGKHKDLNAEEIYSPLTTPVFGKDVVYD
CPNSKLMEQKKLVKFGLTSDALRSYVPLISQEVEDFIARSSVFRGSRGTFNVPGVMAEMTIYTASRSL
QGKEVRSKFDSSFADLYHDLDMGFSPINFMLPWAPLPHNRKRDYAQNKMAQTYMEIIKARREGGS
QRDADDMIWNLMSSVYKDGTPVPDNEVAHMMIALLMAGQHSSSSTVAWILLRLATRPEIMDELY
EEQKSVLGEDLPPLTYDSLQRLPLNAQVVRETLRIHAPIHSILRKVTTPMTIEGTPYTIPTSHVLLSAPG
VTSKSEAHFPEPDLWEPHRWDQGSPLHGTNQEVDDDEKVDYGYGLVSKGAGSPYLPFGAGRHRCI
GEAFAYVQLGTITAMMVRHFRLRNLEGKQGTVETDYSSLFSRPLSPAVVQWERRDRATKA*
>CYP5328L1|10580|Clagr3
MIHISAYAYGVAAVIGVLSHVLYFRRGEHHKETLPYLQLFAASIPISALALAHFLHFSYAQAILCATC
IVGSFVGGIWTSMITYRAFFHPLNKFPGPWSLKISKFSQLVSSWRLDAFRKSYRQHQKYGNFVRTGP
NEVSIASPSAATVILGPKSQCHRSPWYEYAIYQDLESLHSTRDYAVHEARRRVWDRGFSIKALRDYE
GRVTQYTYDLMSKFALFAGKPIDASAWFNYYSFDVMGDFAFGKSFNMLQTGSYHYAITWLDSSMT
LLGRFTPIAWAIPVGAIAPIIGATFRRFIRFCNAQLDERRVAGSKVPDITSWLLKAAPDVNDLEATK
WLHGDSRLIIVAGSDTVAIALTHLFYYLAINPAQVDKLRKALDPLMRGDEPFTVRNVQNAKHLNA
VIHETLRMHPPVPSGVFRTTPPQGITVDGTFIPGGVNLIVPFYTIGRSEECFAFPNEFIPERWYDQPDL
VKVKDAYAPFSLGPYGCIGKQLALMELRTVVSLLVSQFDIRLAEGEDGSKLLEESRDAFTLRMEALE
VVFEERGKKMMTKATMYGVIVLGIKPSTAAVTDDAKNWTFILKYDDECDDKTKDQSSNQYT*
>CYP6272B1|10772|Clagr3
MTSQPRLPVLPYGSPTTLSGFSETFSFHASPEAFITSRVLAFQASNASLADSRTPIRAKVLNRNIAVVSS
YDHVQQLLCNEANTSRLSAGKAYDDLMAPFFPPPNLLLADGPSHQPMKNPWVSRMASLPAEIQSL
VQETVLLHFRGIPSESTIDLYESMKALSWKLILSIFIPSSDERGQAQKDAAEIESLQEDLLRGQFSIFPVS
ISTPLWRSPKAKGLTARKKLQSLLKARVQKGKKGCPFATIDSAAEDDIANHLLLFTSSLAVKSLASFL

TAVMLNLYVYRDEASSQQATSLASRILNSEIPSSREELLKSTILETERLSPPVVGIMRRITQDITLSAQER
FPDILIPQGWDIWLYFVGAARDPAVFGKTADSFLPGRYYNTADGVEHPRPGFAFGAGSKSCLGETL
MREVVTTVAKTCLGMEGGSKGGVVLEASMEDVPRGVQGWLGWQKDVKPQEWARDMKQLPTQR
PIKGVMVKIVHDLSPT*
>CYP59AE1|10971|Clagr3
MPPHHPIFGHLLVLARVMSKLPKDAHPHYLADQLRQAYPEMGPIFYLDAWPFITLTLVVASPATLA
QITTEHVLPKFPAIKDFLYPLANGKDIVGMDGKEWKYWRSIFNPGFSASHLVTLVPEIVRETTVFCDL
LRDHAMQQDIFQMKELTDNLAMDVIGKIVLDSNLDCQHQSNPMVNALRRQMRWMAFGSEGNP
LQQYHPFRPFVHWYYTYQMNRYISPEVDMRFEMFRNAPRSEDKSSSIVRNKSVIDLALIAYLKQNL
NISSSSGIDPLFKDIAINQMKLFLFSGHDTTSSTVCYILYLLSIYPDVLSRVRAEHDDVFGPDPSQAAN
KISEDAYLLNKLPYTTATIKESMRLFPAASTIRRGEPSFTITDPRNGLHYPADPSTLIWLASHACHHD
PAFWPRANEFLPDRWLAKEGEELCPKPGAWRPFELGPRACIGKELSFLELKVLLSLVTRRFELTAAY
KELDAAEEERDIEVDLRKKVRMAAVKSVDGERAYQVGKGEPSAFLPCRVREVVTKF*
>CYP559L1|11089|Clagr3
MDDFLQRLSTPAVAIPSLLLVIFSVYNRLTHVSNIPAGVPWIGKDTTKVFAGTRASLAGFNNVRKWL
EDGYDKYGKNNLSYILPDSFCEPKVILPRSQLAWLTEQPDDILSASAFHHDSLEGDYAFTSPLILKDP
YHEHVIHKYLPRRVASLVPEMWEEVQVAIDQTWGMDTTEWKDVGVWQNMMRVIPSITNRMMV
GRQVCRNEDFLENMGKFAMDVISCSSIYLRFTPRFLKPLLGPLVSIPNRRHWRNTTKYTLPIINERLA
YFKRKQEDPSFHWDEPNDYISWHIKLAMAEGRHDELTPDMISRRLMPVNFAAIHTTALTITSAIFDL
LSSPNSPGWLKGIREEAEKVLREEGGQWTKNGLARCHRSDSAIRESMRVNNFMALNVVRKVTPAE
GIENKVGGWRAPQGALLGVDMYSVQHDPEIYPDPNTYDPFRFSRPRETSDTLNVHSKVNGTGNTG
LVATSDIFLPFSHGRHACPGRFFVALEMKLILAYMVMNYDIEPLKNRPPNTWFGLNAFPSMKSTIKV
RRKEGTTGD*
>CYP6003D1|11283|Clagr3
MAGLLLGNASGQDTSTLGHEDGKYTGSDMDYTYGDAKQKMQTYNDDIRILLRKFPSHAVSLVSDF
LKSGRLPGQTQEMMDIAGALPYNSPIRNKVQAMTVSTLFNSLEHPPKSYLGEKHQYRTADGSNHV
STCDQDFQALNTHCVSFRIDLNLQNFMLPEFGKAGMPYAKTVRSAKKMHGVKPDPGVLFDLLQA
RDEFHPNEAGISSMFLYHATILIHDLFNTNRQNSDISDTSSYLDLAPLYGSSQAEQDRVRLGKDGLL
KPDTFHEDRLLGQPPGVNVLLVMYSRFHNYVATMLKEINEDDRFTPRPNTRVEDAMKPPRVPEKV
VDNDIFQTARLIVGGLYISISLGDYLRAIQGVHEKETTWTFDPRMYIDKTPQGEAVPRGMGNQVSAE
FNLLYRFHPAISDHDAKWTERFMIEEARKRLGKSDDFNLEDLTPTELWTMLASFAREKAAIEPCKR
DLEGLKRGTDGKFSDEALAEVLKRSIDDTAGAFGARNVPRAMKIIEVAGIVQARKWEVASLNEFRT
FFGLEPHATFKDVNADPEIQIALQNLYDSPDMIEMYPGIFIEGVAQEKGKKSKGVGFPGTAGRGVLS
DAVTLVRSDRFYTTDYTTATLTNWGLCEAQSDYKTLGGSMFHRLLQRGLPDFFDFNSVYAMQPMY
TPAANKEIFQRLKVDDQYSLDPPKAPAVKTVIETHGAVCNALSNTKYFASIWSASVGTLVSKKASD
GLDTLTELDQLAESKQLFSQYLIDKAESTVKRSSFRLRKSSYQIDIVRDVAIPLNARFLAELFGLSVKTS
TNTSAFHTSSELYKLLIEVRNWVDYPNADPAARWYRRRKAQESAKVLTESTKSSLSKIIAKPWAFSS
WFWASSEVVENRTAKTTKDVGTIVAKSLHADGRNDEDAAQIAWMMAVEGAGSPVTAFAEVLDY
FLSDAGNEHWPKIQKLARENTSKADDKIADYFMEAHRLSSERICKRSVKSGKDSVKIGDHNLKPKQ
EVLLRLSAANRDPTAFQAPNTFKLNRKSSVPAGNNLQNSILRVYCTALIKVAAQMDGLRKAPGQM
GELKRVQLDEQGCKHTLGQAWGRHYMTADWAYMVQEPTSALRYVQRRGAGARGEEERVGGFF
VAIECTNPKKL*
>CYP6001C42|715|Clagr3
MLRRFSTKFGKSRKDGVNGVNETNGTTSSNGVQTNGSYTNGAETNGTSIEKPASTKRGSSFGFTSKK
PKTQASALNADHAASRAEVESAFAQFAQLIHASRQPLPDQQGHGVDPAQEEHSGLISDVKALGFK
DVQTLMDVMKNKAKGDLQDDHTYLMEKTIQLVAKLPSHSKTRVDLTNAFIDELWNSLQHPPMSY
LGSEFQYRSADGSGNNIMYPHLGAANTPYARSVPPNTLTTGALPDPALIFDSVMARKEFKPHPNKV
SSVFFYWASMIIHDLFQTDHRDFNNSQTSSYLDLSTLYGDTQEDQNLIRTFKDGKLKADCFSEERLL
GFPPGCGVMLIMLNRFHNYVVEQLALINENGRFTKPNEGLTHEMAEKSWAKYDNDLFQTGRLITC
GLYINITLYDYLRTIVNLNRTNTTWTLDPRAEMGRLFGKNGTPAGVGNQVSAEFNLAYRWHSCISD
RDDKWTQALYKRLFGKEAKDVSMQELLIGLSKWEKSLPRDPQQRPFADLKRGPDGKFNDDELVEI

LASSVEDCAGAFGPNNIPTCLKAITILGMQQARAWNLGSLNEFRKFFQLKPHESWEDICPSDPDAA
EQLKNLYEHPDYVEMYPGMVSESAKVPMVPGVGIAPTFIISRAVLSDAVALVRGDRFYTIDYNPRNL
TAWGYSEVQYDLGVQQGCVFYKLCMRAFPNHFKENSVYAHYPMTIPSENRKILRSLGREDQYTWE
RPARMPQRVEFTSYKAAKYILENAKEFNVPWTEPFGYLMPQGGLDFMLAGDTPFHAKQRKLMGE
AIYRDKWHQQIKSFYEYITQKLLVEKSCKIAGINQVDITRDVGNLANVHFAANVFSLPLKTEDHPRG
IYTEEEMYMVLAIIYASVFFNLDPSKSFPLRLAAKAVTQQLGKTVESNVKMVNATGLIAGIVDGMQ
HNHSPLQDYGVHMVRRLLESGMGVSEITWSQIVPTAGAMVANQAQVFTQMLDYYLSDEGKMHL
PTINILAKTEGPEADDKLLHYAMEGIRLNGTFGSLRQSTVSTTIDDNGRPVTVKPGDKVFTNFVSAS
REAEFFPEPNTVKIDRPLENYIHYGLGPHACLGGEASRVALTAMLRVVGRLDNLRRAPGAQGQLK
KIPQPGGLYVYMRADHGSYWPFPTTMKINWDGDLPPFKKM*
>CYP578AB2|1910|Clagr3
MFFLTLIAVIFYVYLIYKYLIYPSFLSPLSKIPNAHFSSSFSPIWILWKRYKEEENNTIHAAHSKHGNIVR
LGPNEVSIACVDEGIRTIYSGGFEKWDWYPNQFSNYGVPNMFSMVNSKPHSIRKRMISNVYSKSYIQ
SSPEVDQISRTMIFSRLLPLLEEASVKRQPLEVLELNFSSTMDFIIAFIFGLQNGSNFLQDIPSRQHWLS
VYQSRRPYRFWAGELPGVVTFFKKLGIHIVPAWVEDANRELEDWTLEKCTAAASSISLESKESSNPIH
PSSTATEPIVYTQLHTPLALSTPPPPTPNLLPFLIATELHDHLAAGHETSGITLTYLMHELSLHPTIQSA
LRTELRSLSPPLIYPSASSTDHDLPSPRDIDTLPLLHACLTETLRLHAAIPGPQPRITPSTPTSLAGSPPLP
AGVRVSAQAYTLHRNAAVFPEPEVWKPERWLNAGEEKEREMARWFWTFGSGGRMCIGRHFAVQ
EMKLIIAAIYTNFSTHVVDDEGIEQEDAYTAAPRGNKLMLRFERVE*
>CYP682BG1|2763|Clagr3
MLGVIQYSILTIFWLPIAAAALYGAGLAIYRLFLSPLAKFPGPKLAALTRKYESYYEAYQNYEYYWKI
KELHKQYGPIVRVNPHELHIDDKDFYYKLNSFQGAWNKDPYTAHQFANPGSIVGTIDHDIHRKRR
AAIMPFFSKQKIYALESVIQGMVDKLCYRIEEYGKTGQPVNLRNASKCFAADVVGEYCFAESGGLID
KPDFAIEEMNQQQQGLKAGLRARYLPSWWMPVVRGAPAWIRASIDPAAKHFEVWHRAVEGPVG
RMEKRKNDEFYEKAGHRTIFHELINSPHLPPEEKGTGRIIQEAGAMVGAGGESTSQVITAFVYCLLA
NPQVLSRLREELRSVIPNADSPAPTLRQLEALPYLTGCVKEALRLRTGKIARHQRVPRDRPLYFNEW
EIPAGTICSMTPIFLQIDPEVYPNPHAFMPERWLNLDEQQRQRLEHNLVPYSKGTRGCAGLTLANA
ELYMLIPALVTRFDLELFDSDAWDTEMAVDSHHHSPRPDSKGVKVFVKKSTF*
>CYP51203A1|3091|Clagr3
MESRLIVELLVFVGLVFFGSFLRRWLYSVESETHLPWVGIGGGRFKKLRASVRQISDSLETLSYGYTM
YGKYGQPFVMPDPSLQPQVMLPQEHISWLIRQSDGVMSQGQVRRERNALAYLHMDVDYKATMA
LIDKIINPCLQRKLNRVQGNVFDEIRASVDDTFGLDDNSWHELKLHESLQTIINRTGTRAFFGLPICR
NQDYLYSLKRFILAMGAGTLIVGQLPLWLVRPLAASLINIPLRYYKAKALKTLLPIFAERVQSFERHE
AGYTIEDEPEDFATQTIQLVTHAKDGTYNRSFGYLAEQHLLLSFAALSSTAAAATNLLLDILSSAPEV
DAYNILRSEAALTFTTEQAWGDPASLQKLTCTDSAIREGLRRNALQIRGLLREVMPKNGITLPDGTH
ISRGTWVGIPLQAVHMDERFYAKPEMFDPFRFARMRTDPALEGERFDATQTSDKFLAFSYGRHACP
GRWLVSQILKLLIAYITLHYDIQPLEKRPLNTVFGDTNIPNVSATIRVRRRKFA*
>CYP5105E2|4317|Clagr3
MPSWTNIVIGFITLQLAYTIYTVITRIFLSPLRHIPGPKLAALTSWYEFYFDAIQQGKFVWKIKELHSKY
GPIVRITPWEVHVNDVEFLDDIYAASFRRREKYAYQTRTLPVPLSVGGSLQYELHRKRREALNPFFSK
RSVVELGPMISQKADLLCKQFENHLSSQIPINLSEVYYAFSNDVVLQYSFGHDDDLLGNPVRAATLR
AHLSELLLGVKFNQHFPWIVNTLELLPMALAKHIMPPGVLDMKEFSTKISEEIEQVLHDHDDTKRG
GKRSIFYELRDNPTLPPSEKSSLRLEHEGTLLVMAGTESTAKSMAITHFHLISNPAIMAKVRAELRTV
REDASWTELEQLPYLSACIAEGNRLSFGVTARVCRIAPDEPLQYKSYTIPPGTPVSMVTLCAHTDEHI
FPDPWTFNPDRWLGPQGAARRKYQMAFNKGGRNCIGINLAHAELFLVIAAVARYDLELFETDISD
VEFQHDYHVAYPKLDSKGIRAVVKRKIAMD*
>CYP5042B6 |4615|Clagr3
MYSIESSYYPYLGFFLFLCSSCVALVQFLKPTDVKRSGGRKRWAMPPGPPGLPVLGNLRQMMQARR
GGALSFNEWLSSLIPYGEMVTLHMGSQTWIVLNSDRVVSELIAKRGKITNERPQMPIASGLVSNGKR
TVIRQEEEWREGRRVMHQLLSGSNLKVYAGMQELESVDMLRRYVREPDLWFSNNFRYATSVLYRV
VMGYPLNKTKAALDDYQRVTIEFVTTINSSYVDFFPSLSKLPHLLQPWRHYWAQMGSFHRRVLQQ

WWDPIRAAVSKGSAPPSFVRDVLLHPDTRYSGDDEEAMYLATSVMAAGGDNTRMTINTFIMAMIT
HPEAQVRARQEIDRVCTSGGSLRLPQMSDLLEMPYVAAMIKETLRWRPTVPLIPPHQVTENLEFDG
YFIPTGTVFLINSIALSSEFANAHMFQPERWIDSSEARTTNNFWGFGGGRRICVGWKVAEQALFIAF
ARLLYCFELSPRGPIDSERLNHRTLDEPFPVKATVRSAEYALLIEEEASKYEASFSKFRLTAK*
>CYP5134G1|4621|Clagr3
MDSNTSASHTTALLAGLLVTLGVLLYRAALPKPIPGIPCAQYSINRLFGDVPDALKYHAKTSETVAF
LASRCEELKSPIVQVFVRPFARPWVVLADSRESQDIMVRRTREFDRSGFHSDLLGAVVPNPFQMTSA
EDELTLMKEVVMRTNDQWRHNRRLVNDAMSPRFLSNIASQQVYNQALNLVELWRQKMRLSKGH
AFNLRADLQLCTADSIWATTFGDDIAACKVQSDYLRTLDSIKLLEDEEALAQFPAKPNPDAYAAW
KAISESGEIPMNSPLGRHHHWFAVTFYPYLRRAISLKNNIVKNKVQKAWGKAKIDNASESNAIDSIV
DLVVDREFALAKKEGRVSEHDSKFVYDELAGFMIAGVDPTATNMGWGLKYLAKHNDVQTRLRK
DLREAFPSALQEGKPPSANDINKARIPYLDAFIDEVLRHSGGQPTNVRVAISDTEILGYHIPKGTDIFL
LVNIPHKPLSSIPSPSYVLTMGPSYKSPALPIDESIRSPSSREFKKDSEKWAHEDLGSFNPDRWLIRNEQ
GEIDFDSRAAPMQAFGAGMRACYGRKLAYLEMRIIYALVIWNLEVLPPLGALFDFKAIDKLSHQPQ
NVYMRFDVPK*
>CYP682AW1 |4961|Clagr3
MDKMTMAEILAVLFLFYIFGLYIYRMFFDQLSHIPGPKLAAASLWYEFYYDVIKKGQYTFEIGRMHE
KYGPIVRISPYEIHINDPEFIDEVYPGSSPRSMKYQWSQKMFGIQSAFLVTESHELHRIRRNALAHYFS
KQSLRDLEPGVQSQVDKLVSRLQGLKGTGTAINLLDVYACLTGDIIGQYAFAKPYGFLDDPDFSPY
WHQIMVEVSQNGHILKQFGWMLPLMQSMPEWMVKKTNPLMMTLINFQKVQRFRNQVIEVKESI
ARGEKPTGQTTIFYDVLTNPNVRPEEKTTDHLQDEAQTVIGAGTVTTAHILSTLTYHLISNPSILQTL
QSELSPLMSSTANHPKWSQLEALPYLSAVIQEGLRIGYGVSHRLQRLFPDKSLQYKSYSIPPLTPVSMT
AVFVHDHPSLFPNPRTFNPDRFLKNPGLKKYIMSFSKGSRQCAGLNLAYAELYLALVAVFAPGRFE
WELYETDITDVELKHDFLNTSPRLDSKGIRAVVN*
>CYP51195A1 |5450|Clagr3
MNFFDNYDGLVSHGRAYFDDDAPFAISFPGRTYNVVVKPEDIADVYKNSTSLSWDYYLKELLLSFG
VSKSALGKDLRNPGTHQNGHTKQPNGIKFEHKLLVNWTEDLYRQQFPPGLRYQKLSDAMLRLVD
DALQWKKLPSRYTVNQERIPLMDFCGDILIQATTRSLLGDLIYEIEPKFTPMMIDFNEEAWKLLMFP
YPQIVAPRLHNAKKGIHKALCKYVQSSPDSQTQLAWVIDEILEAKEAAKLGDTDKASMIHTLLWIV
NINAYRLSFWILSYVLFDPNLLANIRDEIKPAFGSNTVDFSYILERCPLLMSVFNETLRLTYGSVSVRR
VMAPTMIGGKTLQSGTSLVIPIRQLHYDDEAFGGDADEFDPQRFLKNDLDHSPSFLPFGERFDAFPR
PFIAKREVLVFVALVLKRFEFEVAGNSSEEKAKPSPPQIDSICQTLGIMQPVKGTEVYISLKEAA*
>CYP6573B1|5774|Clagr3
MGIIAFTGYMPERCSDFVMIILALSVITAAALVLHRLLLSPLSSIPGPKFAAATRWYEFYYDCVLPGKF
FPRDREDAYIIWFAFFACTLVTSDIYSLFQGPIVRISPNEVHINDPDYFSQLHSFASKLDKCGWYYNFA
ASPTAGFSTASYDLHRIRRGALAKYFSMSNVARLEPLIQSCVTRLRSRLEEHRKEGTVVDLSSAYRCL
ASDVIMEYALPHSRRLLDSSDFAASYHRVVRNLGRIGLWNRQFPFIIPFFRAIPRWIIAKLDPGPALSL
ADFQIVSSNNFFFSNNVLTQENSTKSLRDNAISITSSGCRIESIEAKATVLRELYQSDLPVSEKTIQRFVE
EAQTLLVAGSETTGNTLTLTTFYLLADPERAGKLKQELAQVKKGQNSPLVYHDPHRLPYLTATITE
GLRISSSVAGRLPRINPHAAMNYSDFVIPAGVAVSMSIRDVNLNERIFPDADHFIPERWLGDSEKRQ
VLEKYMVAFGRGPRNCIGMNLALVEIYLVIGNMFRDYNMRLVETKEEDLKMTHDFFSPFGPINSKG
LRIKVG*
>CYP65FD1|5876|Clagr3
MHLIDNPGLVFLTTVIAVILYIAISALNNLYRHPLSRFPGPRLWAISRIPYVWVLIKGDLTQRTHELH
QRYGPVVRLAPNELSFIDGQAWYDIYDHHQGRPNFPKNPLWMAPGDDGIHSILSANDADHARYR
RLLSHAFSEKALRQQEQLLQSYIALLIQRLRTRASSAESAIIDMVQWLNFTTFDIIGDLSLGESFHCLD
ESRYHPWLSILFTQFRIGSLFIALRFFGLAGYAKRLLPKSLLEKRKDHINMANERIRRRRAQGASVDG
QRNDFMTFILRHNDEKGMSDPEIEATLRILVLAGSETTATALSGIIGNLLGNSKAMKELTAEIRSSFR
HTSEIWSERVSELPYLGAVIEEGLRLCTPVALGMPRVVPPGGAEVSGHWLPEGTFVSASGYASNRSR
LNFPNSPLNFVPSRWLSRTPSLDKPNPQTNNASPPSAFNPFSLGPRRCLGRNLAYLEMRLILAHLIW
AFDFESAESEESPWSWERQKSWILWEKTPLSVRIRCRE*

>CYP65FS1|6484|Clagr3
MPQMNGLPQDIVGRCLFLGCGAVALYSCYQLITAIYNVWFHPLASFPGPRTAAATHIPYVRASVAG
KLPHWMKKLHDQYESDVVRISPSELSFIGASAWHDIHGHHHDAQPFEKDPRVNGKSFNNVDNML
TAKHEDHERMRKVFCHAFSERAIREQEPTVQMYIQKLIRRLHEQINNSTQGKVDLVKWYSWMTFD
IIGDLTFQSSFKCLDNQANHRWIDMVVSSFETLVLMGICDRFTISRIFLPYLIPKRYKKMIEDHWNAT
EETLARRFESGTKRRDFMTPVLEDNVDGKGLSRHEILSNAWLFVNAGSETTAGILSATIYYLARDRRI
LSIITAEVRGKFSDECQMNAQSVAELPYLLACLSETNRILPAALIGQAVLVPPQGATICGYWVPGNT
GITNNQYACYRSKTNFKNPDDYVPERWLDDPGYASDQRKAFQPFSYGARNCIGKSLATMELRLAL
ARTIWNFDVGFCEESDLNWDDQKVYMSWKKTPLIIKLAASPQARS*
>CYP526AB2|7158|Clagr3
MAPWNPSMPLRSGLMTPTGVCLVVAFVAVGLYAIYQWLLPKPLPGITYNPEATKSLFGDAPDMIRE
ISVTGEFGLWMAKQVEKMRSPVCQIFVRPFSKPWVLVADFRESQDILMRRTEFDKPTFLSDGLQALG
DFHARYKTNSAFKARRQLKQDLMTPSFLKNFMGPFMHSKGLELVKLLETKMNLAKGRPFSVIADF
DYAALDVMLNYGFGGNMVDSALGPQLNLITQLDSSEIPDGDPDEPVTFPKAPISHFLEAVHDAPEV
IEKTIIAWTPRLSHWWWKQQSWYKKIFSQKSRCVPPQITKALENYRAGEVKSALEHILRREQVTAEK
QGREPQFDSQSMVDEIFADLIAGHHTTSISMSWVTKFLTGYPHVQFKLRSALYSALAGAVAEKRFPT
FDELRRARIPYLEAVIEEMLRLTPFSMSRETTCDTEILGRMVPKGCQVFMVNSGPGYLSPSIPVDDVQ
RSPTSKAAKVRGSWDETKDLRIFEPERWLVQKEDGDVEFDGAAGPQLGFGMGIRQCWGRKMGYL
GIRTIMALVVWHFELKKIPESLGGYGGFDGMTRQPHRVFVRLGRAVS*
>CYP52K11|8178|Clagr3
MLHPHYLLLAAAILAILYCRRLVHDYQARQAHESFAKQHGCLPPPRIQNQRPWGLDRLEQIFRAD
KESRLMEVFLFHFRQTGNTVEQVFLGTPAFATIEPANLEALLSTKFKDFNFGKRRDVSYPLFGDGIFT
QEGAAWKRSRDLLRAPLQHKHYENLASSSPVFLFGESVHSLTATKFANEQRFGEAFNKAQAVIAK
RFRLPDLYWMIGGSEFRRACSVVHDFVDQLIDRNVFRGHGKSNVLLDEVAESTADRVALRGQILSL
LVAGRDTTACLLTWTFFLLEKCSDPANLTRTRLREMGYLQNVLKETLRLYPSAPVNERKANKTTFL
PTGGGPYRTAPVLIPKGATVAYSVYALHRRPDLYGMDAELFRPERWDEDMPVLHDKTNAAWGYL
PFNGGPRSCLGQDFALTEAAYTVVRLLQQFPIIRLPMRHKVELTGVEKQTMTLVISSTEGCMVEMAP
*
>CYP6001A25|8730|Clagr3
MPSAEAALPNTPPEQHVTFRREVATTFESVSGVIDAVTHPLPTQTGDGTYIQETKPAGVVSDLTRMR
PRDFKTLLDLAKTALSGDPLNDQDFLMEKLIKLASELSVTSKESGNLSNQLVQKLYDSLQHPPAAT
MGKEHRYRAADGSFNNPMAPMIGAANTPYARSVRPQSLQPVDRPEPGDLFDSLMARGKDFKPHP
AKLSSMMFYLASIIIHDLFSTDHQSGNNATSSYLELSPLYGNNQQEQNHMRTFKDGKIKPDCFSNKR
VLGFPPGVGVLLIMFNRFHNYVVEQLVAINESGRFTRPKEGASKEKLMAYDNDLFQTARLINCGLYI
NCILKDYVRTILDLNRTGSDWDLDPRNAKLTGPTANGTPGNQVSAEFNLVYRWHACISERDTEWT
KKEYERLFPGKEACEVSLPELLQGLGRWEQGLNEDPVKRPFADLTRAEDGSLDETALVKILTEGIED
KAGAFGSRNVPDILRSVEILGIIQARSWNLASLNEFRSFFGLTKHETFESINSDPQVADTLRRLYDQPD
FVELYPGLVVEETKPQMVPGSGLCTNFTISRAVLSDAVALVRGDRFYTVDFTPKSLTNWGFNEVSSD
FTVDHGHVFYKLFLRAFPTHFQPNSVYAHYPLVIPEENLSILHELGQKDKYTWERPKYTPLPTLITTY
AACKSTLANQEGFKVTWGEAIKFLMHNSGKEYGADFMLAGDSAPNANSRGVMCPALYRQDWEG
AVKDFYEHITLKLLRDKSYKIAGTNQVDIVRDVGNLAQAHFAAEVFCLPLKTEQNPHGVYSEGQLY
SIMALVFTCIFYDADPAKSFPLRQAAQKLTQQLGEIMETLVIPELAHGIFGGVMERLHKQQSLLSLY
GTHMLQKLSESGLGARDLIWSQILPTAGGMVANQGQLFAQCLDFYLSDEAAPHLKEINRLAKLDT
QEADDLLLRYFMEGSRIRATVGLYRKAETDTKIEDGGRTVHVKAGEKVLCDLVTACKDPKEFPEPE
KVILDRPLDSYIHFGWGPHKCLGYGASKLALTTMLKTVGKLDNLRRAPGTQGQIKKVPVPGPGGLT
MYMTTDQSSIFPFPTTMKVRWDGDLPAVN*
>CYP59AC1|8988|Clagr3
MSLASACSVTGTMSLKTGTPSWRLVLQACLMLLAIGLARFFVKLYRIRCKFQQMQKEGLPMPPHH
PVFGHLQLVARIMSGLPKDVHGHVLPHQIGLLMPELGPIFYIDTWPFGPPILAVAAPDPAYQITQLH
SLPKFHALREYMRPMTGGNDLTTMEGKEWKTWRNIFNPGFSSGHLNTLVPEIMEDISTFCEILRDK
AKKPDIVLMDPLTSNLSLDVICRVALNTRLSAQRSTNDFVSSLRNQIKWLSFGNEANLFERYHPLRPI

MRWWNGRQMVRYVSRELDLRFNSNRKRSGLAKNGRSKTIIDLALDNYLAQQTHNGSSDAMDAT
FRDFATSQLRTFVFAGHDSTGSTICYAFHLLSTHSSVRRLLIAEHNRVLGPDIDQASSRISQNPHILGQ
LRYTLAVVKETLRMYPPASSTRSGVPSHSIQGLNGRQLPTDGFLVWSNSYAIHHAHEYWPEPHKFT
PERWLVEEGDSLYPPKGAYRPFEFGPRNCIGQELAVLEIRLVLTMTAREFEIKSVYNEWDSLHPTNG
PKTVNGDRAYQILSGAAHPSDGLPCRISLAVD*
>CYP6352A1|9014|Clagr3
MGWITSIVSASSLKLLPWLAFCAIVIGFFHSAYVALAGPLSDIPAIHWLARWSRYYNLYMKYFYGTR
TTHYEAHLGTDKNSSVHPIIRVGPSEVSIMSTEGVKTVWGAGFERSPWYAVFSNFGKPNMFSSASSR
DHSSRKRIFASSYSKTSVTGDRVQNIIKSRVAKIMNFLERQSSSNSTFHGSSGPIVVRNVFRALQADVF
TAFAFSEGEGTSFLNHLENGPNTVEDLGMGMMDLFHDEKRDEFFFWESERPFKYISKFLARNGPKS
HERAERWLMELVKPHEARLQSRNPIKSTDKSLLQFNGGVYEKLMFYKNPETGNPLDWTERASEIM
DHAVAGQDAVPAALEFIIRQISTHPHVQAKLRLELLTSLPLSAEDRSFAMIDTLSYLNAVIMEGLRLV
DTVSSYQTRVVPRGGCVVCGHYLPAGTIVAAQPYLINRQPDVFPNPETFDPSRWLLPREDYRNLAK
SMWTYSSGPRSCVGRELSLAIIKIVVADTYTRYKTTLLDVDRTERRPWEGADSMAEVQFENTLPCED
GEWIENRVRKPSLFPVEAHEQVQEPVRRSSIFQPSSDNRALCEDKMQQTVGSPPERQRPTHIQFNLN
SFTKSNETPNADISFQKPAHEGEQKDNNFNSQAIPSHQNLDAQAWLAQHYGPRKTSLVPSLLPLPL
GAQGQKGGEQREDISRRPSVMHMYEADEDIAALTKPTEKVSRPKRPGLGRMRTAPERVDGILQGN
*
>CYP5391D1|9060|Clagr3
MDQTYAPSLLLAICGIAAVFLLSNVVTHRKPYPNGPVPIPFLGNVGTLRKLSAKLDPQLLQLKKDW
GSLCMLWYGRSPVIIINSPRAARELLNERGAINSSRPEQNEFRKKLWPWRLVSTPMNADFRFLRRLY
HNLLSAEQALLFRKYQDFESTIMLQNLLQTPENFLRSAERYTMSVIFSAVYGVRLDRLDHPILVELN
GLLDATMKYFEPGTLLIDFLPWLQYLPTALQPWHRRLVPLVDRESALHAAFLETLRKAVEQGTAPD
CFCKQLIEVQQRESLSDTKAVHILAMLIGAGADTTGTILQGFFKIMALHQAAVREAQEELDRVVGP
NRMPTWDDEKSLPGIRSLIKEVHRYAPIGSLGIPHCSTAPLTYDTHHIPAKTILFPNLTALSKDPDRYP
DPEVFNPHRFHNDHTTAAASALSKDHMQRDHFHYGFGRRVCPGVHVAEASLFIVVSRVLWGFDI
GPKEGCPLDMDAKTAILINKPKPYEVEIVCRGEAYRKVIEESASRGVATGILDLDSVVAPGA*
>CYP567E10|9343|Clagr3
MPSIHLVPEHGLMIDFTVARLALTALLCSSIYWAYATWRKASTHPLSRFPGPFTASLSNFTHCRRFM
GGRQPYEILKLHEKYGPVVRTAPNELSFNTAQSWRDIYGPRKGHELFIKSEFYDGLNFATKVPSITTE
RNPAKHTEMRKYLGGAFSDRSLREQEYLVAEVVDQFIEMLDNSSGEEDGSGKELDLVNAFNLTTFD
IIGSLAFGEPFGGVAFGKEHFWVSIAVSSLRKGALADGFKRFPWVSAAVQTLFSGFIKKMLEDKRKH
EAYSMELVQRRIQRQTNRKDFMTRILLEREHQDISDAQIAAHARDFIIAGSETTATALSCASYYLLKD
PRVLQLLQQEIRSAFKAYSEIDGTSTVPLKYLNAVALEALRIYPPLPVPLPRVVPAGGDTVDGHFLPE
GTVVSTNPFAASMDSTNFHEPWEFIPERWLGQSKEDELNASQPFSMGTRGCLGRSLAWMELRTILA
KLHFSYDLALLGDGQDVDWQRESRMHLLWKKPKLMVKATRLRR*
>CYP5070D6|9652|Clagr3
MAIKMEILPLLILSGGLAVGAQKFASMDALRVFGAAFGVSIFVYLAYWSYIFPFYISTLRKVPTVPGFP
LWGQFFTIITTEVGTPNREWHKKHGPIVRYFFPFGAERLSIADDDALNHMCIKNPYNYPKPDRAKQ
WMVRVLGEGVLLAEGNPHKHQRKALSPGFSIQSIKALTPIFWRKALLLSKLWERDMLVAKTKSMTI
EVLEWLNRTTLDIIGEAGFGTDLDSLEHPETPIREAYRLVFAFDIQSRVLHGLAAFIPLTKYLPAKMN
RDILQSRNIIMSKATDIVRQKHQKTHAKDKDIIALIVKDNMRMEAAGEATLSFETMRDQVMTFLGA
GHDTTATGVAWTIHLLSTHPEVQERVRAEIKQHYPFMFDHASRNDVDRLSSLDADNLHYLDNVC
RESLRYIPPIPMTVRETITDDRLGGYDVPAKTTVYIHANAIHRLPSFWGDDADVFDPDRWDRLPEGH
TTNAFMTFLQGPRGCIGRKFAETEMKCLMICLLSMYRFERDMAVDDPESWKMWRLVLRPRDGVQ
CKVTMLEEEEKMAIGA*
>CYP6001J1|9791|Clagr3
MFKLFGALGSNGPDKGDESNYGDDSFTEAKVKQSGLLDDIKALGPNLGKDALTLLEKVTSKGKPY
DDRTFLMERLIALTASLPHNSKMRDKLTQKLVGTLWDSLQHPPLSYYGEQYQYRTADGSYNNILFP
DFGMAGMPYAKSVRSVKALHGAKPDPSLLFDLLMARPDGKFAQNTAGINSMLFYHATIIIHDIFRT
NRQDANISDTSSYLDLSPLYGRNQEIQNTVRTFKDGLLKPDTFAEERLLGQPPGVCVMLVMYSRFH

NYVAQNIAAINDDNRFNMPRQGDPDYNLKIQKRDNDIFQTARLIVGGLYVNISLHDYIRGITNIHH
SASSWTLDPRIEVAGTANTPAVERGVGNMVSAEFNLLYRFHSAISQRDSRWTDQFFKSIFGDKDSQE
IGLEEFYRGVLKYEASIPEEPSERVFGGLARNPSTGAFNDGDLVTILRESIEDPAGAFGARNIPKHLRT
VEILGIIQARKWEVASLNEFRKFFGLKAHDSFKSINPDPEIADLLRKLYDSPDMVELYPGLFIEDAKPR
MDPGSGFAAPYTVGRAVLSDAVTLVRADRFNTLDYTVATLTPWGMAEIKQDYQTLGGSMLYRLIH
RALPKWFKFNSVYVMQPMYLPSMNEKILKELGTMDQYCLDPPGPPPKTTLLNSHAAITALLDDHA
NFKVKYGLQLPDLVFPEYMLTGDSPKNRDNHKFVAQRMMNVPGGLELYAKSFYDTTRKILARESY
KLGNTFQVDITKDVARVVVVQTHSEFLNLGLSQNGKEEDTLSEEEMYRCMVNYLNYAIVDGDIAES
WNLRREAHEAFAKLKRTTECAVRKHGRIGGLVGNFFAPPPAPKGSLKEVGQKLTQDLLGAGYSVE
TTATTLLTTASGGISNVPSTFVSVLDWFLKPENSHHWAAVRKLAAKDTAASYETLKKYVLEAGRLS
GFLALVRICVPERGEAAQIRTDRGSMVTLRKGEVVVCNATAAFRDASAYPDPDQVKLDRPMELYQ
MWSIGPHRCVGRAIAITALTSMIKACAQLKNLRRAPGDQGKIKHVPGPLPGSRKYLSDDWSRYQPF
AGTWKVHFDDYSS*
>CYP5436D1|10383|Clagr3
MTRSEWALLMRSKDPGYLFTGRAYVQSQQRFLGCLSFGLQIKGETWQFTGKIHAQYGSIVRIAPDEI
STTSPGAWKDLYMTKPLMPKDPFSQTPPMNRAESLFTAAGETHHRIRKTFINAFSDRSYKDQSPIKE
SYASLLMTRLHRERGKDPGGKVNMAKFYGYATLDIIADLTFGESFYGFLLGAKFGSVQNSLSRYHPI
DKKTATDKISRRLEIGDIGPSRSEFITPIVRKLNDTQTKGITRKEVNTNGLAVVIAGCQLTTVALATYT
FLMLRYPETLKRLTKEVREGFESESQINTYSTMNLPFLAAVINETLRIHHPTPINLPRVVPPEGQMIDG
HWIPGGNIIGVNLQNIQNDPDNWVEPHVFHSERFLPKTDCRYEQRFDKDDKQSFQPFSVGGRNFM
GGK*
>CYP578Y1|10401|Clagr3
MASLTIIGLVLSLLLLFYKYIVFPAFLSPLSKIPNAHYTAPFASLWISSKRRASAGTRAIFSLHQKYGPV
LRLGPNELSVCTPAALKTIYIGGFEKDQWYQDAFMNYGSPNMVSMLEHKPHSVQKRMVSNLYSKS
YLQSSEDLQKASTHLMLDRFLPIMQSVADKGVEMDVLDFLQGVGMDFTSAYLFGLSNGTDFMHD
VQYRQHWLAEYSVFKDQLPHERAGGEVERWCLAMCEAAEEFLHSENAENDSLTTQPVVFGRLSQ
SLAASSTKLDSKSHLMTTASEMLDHLVAGHETSGITLSYFMHEISQRPALQDRLRSELLTLNPPIRPG
NNSIPTPRSIDSLPLLDSMLQETLRLYAAAPAIQPRRTPSSPGGTTIEGYKNIPGGVRVSANAYTLHRN
PEVFPEPLKWIPDRWMDTTKGNKDEMKRWFWAFGSGGRMCLGSNFAIQDMKLIIAAIYTNYTTEI
VDDEGIEQSDTYISRPIGDKLILRFKHI*
>CYP5436C1|10437|Clagr3
MFLDTSVSSATPTLSTILAWSGTIFLLYYLLSALYSIYLHPLSKFPGPVTSSFSRLPFIRAHFGGKLHDYT
YAIHAKYGPVARIAPDELTIISSAAWKDIYQLRPGLPKDPYSRTPPPNGAETLFTAEGDTHARIRRTF
ASAFSDKSLREQASTIETYASLLIWRLRRDIHQDSSNVVDIAKYYGYATLDIMGELALGDSFHSLDGN
NENSWVHLFFKGVTFGSIRTSLSRYYPLDMIFGSIFLRLTSKIRMKNMALGTSLIERRLKMGEMGIVR
NDLMTPVSGNIISHPQEGKQKGLTRAELDVNVLSMILAGSPLSHIAMSAVTWYSLRFPTAMEALTR
EIRTSFEKEEDITVVSTQDLTYLGAVINEALRIHHPTPSDPKPRLIPPEGLQVGDHWIPGGTSIALPFRA
AFLSPLNFTDPLSFHPERWLPESHPAYDPRFANDDKEAFKPFSTGTRNCMGTKIFLAEARVCLARIV
YAFDIQLAMPDEWDWPDQKAYLTHEPKSVWVKLVERL*
>CYP5095N1|11287|Clagr3
MENSKLSLVAPYLVLGSILYLIAVVIYRLYFSPLARFPGSKLTAATGWIETYYDVFKGGQFTFQLQKW
HDQYGPIIRINPDEIHVSDPDFYDVAYASSAPFDKTPKWLDRLGFPGAVQSTVQHELHRSRRMTLN
PHFSKKSVNNVSWFIQQRMDRLCDRLLREYKGTGEVVTLNDAWGAVTADIIVKHALGFDYNFVN
YPGFVAPFTRSLKELVLSVHVAGHFPWFLKLLQFSPDWLVGLLNPGMKPVFSLQNEIKKQIIRIKQL
DNQNDSTERHETVFTDILGSYLPPQEKTTTRLQQEAGSVVGAAIETSKATLSVTSFYILHNPEILRRLT
TELREASLGDPETPTWPELEQLPYLTAVIKEGLRLSSGVIQRLPRISKHKAIQYGTYSIPPGTPFSMSSYI
QHTSPVFNDCFAFNPDRWLTTVKVAPAHGREEKSLDRFFVPFSKGTRSCLGQHLAWAELYIGLATL
FGRVDLELFETGLEAVTTAREYFVPLPSAETKGVRVTVR*
>CYP5094D1|11364|Clagr3
MSLLVQCWTFLMSLLLYVTCLVIYRLWFHPLARFPGPKTAAATKWYEFYFDIVKRPGGTFMYEIQR
MHDVYGPIVRINPDEIHIHDPEWLDVLYTRAPKSVRDKYEPAARMTGMPESSQNNLLTSEASTDTL

WLVFGTVSHDLHRKRRAAISPLFSKGAIKASETMIYEQAELLCVSLEKQLDQNGIAEMRTNFTAWA
TDVISILAFPKPLHLLEDLQAAVDYHLSTKASMLLTPLQKQFPWLIETAWTLPQALVRVMSPDLARS
VVLYRDMLEQAKAVIHENSKATLGDCVGNPPSQVSMKTDSGSDAFQAIIACDSLPLHEKQEHRIAQ
EAFNLVVAAGATTAVILTTATYHLLIDQDNYLVRLKEELEAVMEDPYTRVSVQTLEQLPWLELLNT
TDQSAVIKESLRITGLASSRLPLVSPKENLRYGEWNIPAGTPVSMTPRDILLDPSYFPEPSKFLPERWLS
NNPDLARMERVFVPYGRGTRVCLGMKYVILLTVTPEHSSLADTPPISSLGHSELYIAMACLFRRFNF
ELHETVRERDVDGVRDCFGIEPSLESQGVRVRLAKTAVM*
>CYP584L8|2841|Clagr3
MATFQLPDLLHVLLASIFFYVIYRVYWELTTGASRRALIKEHGCEPIKRNPDYNYFPNNVIGYRTLLE
NIAGIKQHNFLERIRNRYLRFGNNTMMQRMIFTNMIQTIEPDNLKTMLAVNFKDFSLGDRRTSALT
PFLGHGIFSTDGAAWQHSREMLRPNFTRSQVGDLTIFETHAGQLINNLPRNGVTVDLQELFFQLTM
DSATEFLFGESTNCLGSTASSPNMKFPECWNRGQEAVAEGSRSGKLGRLLNNRAQFQKDTQFVHE
FADRFVRKGLELRMNMDVEKAGDEERYIFLHELVKQTSDPVQIRSELLNILLAGRDTTASLLSNLWF
ILARRPDIWQKLRTEVDALGGEPPTYQEIKDMKYLRAVLNESKASIHPFLPFPIKISKLQTPSALRLYP
VVPGNARTAIVDTILPLGGGPDGKSPIFIPKGQIVQYSVYAMHRRKDFYGEDAEEFKPERWETLRPG
WEYLPFNGGPRICLGQQFALTEASYTTIRLMQEFSTIEPRSSQPWQEWLTLTCAVHPGCFVGLTPTT*
>CYP584AQ1|3933|Clagr3
MLFSYPLTLLQTLSAAVSLCVVYYLYLEITVALPRRRLAASKGCLPPPCFPASDPIFGLDFFYENFKAL
KAHRALELFAEKYWRLGRNTAKLNVFGKTIFTTIEPENVKQVLSLSFKDWEIGEQRKKVMEPFLGE
GIFTTDGAEWQHSRDMLRPNFVRAQVGDCEMFERHVQHLIRAIPKNEETVDLQPLFSKLTLDIATD
FLLGKSTDSLLQEGADAKTKKFVKAIDHAMNGLGSQDEGLIFWIAVMMGIMVQNWTLRGEFKIV
HDYVDGLIEKALAEKQENLEVEKNQDSIGSGDKKRYIFLHSLLSQTTNKLKIRSELLNILLAGRETTS
ALLSNLWFVLAKNPHIFAKLQHEISTTLPNDGEPPTYEQLKEMKYLRAVLNESLRVHPIVPENARIA
QVDTILPIGGGEDGKSPIFVKKGQIATWSLYAMHRRRDFYGEDAEEFKPERWLDTDEKKGLRVGWE
YLPFHGGPRICIGQQFALTEASYITVRLMQEFSELESRDSEPWYAKPFLFPYPPMFAELVLLVGMRLT
LCFLCCGRREKFSLICSNFGGCKVGLKARV*
>CYP6839B1|5322|Clagr3
MEAANSHLASSFLGKSIFSEDGAYWEHSRNLIKPTFSRSEISDIDLLDTFVERMIHLVPRDGTAIDIQP
LRHKLFLDRSTYFLFGSSADSLLPDTPFDTPESINAFNQSLAGVGRCRRVSMLRFIYTFDKSWRVAYG
KVHKFVDSHVKRALDQTAKEADPAVYKPSEIDGNAARHHYILLREMAKEIRDPIELRYQIFQVFLP
ARNTTSIAVGNALFHLAKKPERLAGTASLVRFRHVIQEKIRLQGPSGPVLCTALQDSILPVGGGPDG
QSPIYVEKGVVVALNVWCLHHDRDIWGDDAHKFNPHRWIDKRPMWDFVPFLGCPRICPAQQQV
LIQAVYVLVRLVREFARIENRDPVAEYVEFTKMTTESRNGVKIALFPAESNLQ*
>CYP584AV1|9094|Clagr3
MRLNEISLLTILILILTYLIRTFLKLLSRSKIVAKYGCLPPPSLPQRDPIFGLDVLLQFFKALKENRRNMS
MNELFQLHGQTFQSVTWGSTKIYTIRPENLQAVFATDFSSWGVQPMRLFAFEPFVGRGIMCVDGAL
WEQSRALIKPTFTRAQIADLHLTAYAVHVNKLIDLLPKDGSTVDLQPLFSRLALDSSTEFLFGESVGS
LSPHSISTDAKSFLEAYDYGQMVVGKRLHLPKWNFLTRDKKFWDSCKIAHEFVDGYIAQGRQLCE
QSENGKVVPERYILAHEMIKQTRDHNDIRNQLLNVFLPAHEATGVALTNVFFNLARNPSCYAKLR
QEILDAGEQAAWTFERLKSLKYLQYVINETFRLNPAIGTNTRIALRDTILPTGGGLTGTRSIYVNKGD
IVTMSFYALHRRKDIFGDDENFFRPDRWKTLRPVPWSFVTFGGGPRVCPGQQLALTEVAYTIVKIVQ
SFPMIENRDPVKEFVEVYKITTTSRNGAKVGFPTP*
>CYP52AJ5|9656|Clagr3
MTFNMRLLSPFSFLLGLVVLLAYKVFSSTREKRRQHAEAARRGCSTCPVLPRKDLFGITQFRESIRAT
RAERGPRYVVEKLDSMGKDVHTIQVRVLDYDLNVTRDPENARACFSSQSSDFDIGVHRTQSWKPLL
GVGIFTSQGEAWKHSRALVRPQFTKEQISDLDLAERHTRDLTKKIRAGSDGWTETMDLQPFFYNFA
LDTVTEFLYGYSVHAQNPDARAALPTVRGMDEPNLLELGVNFDKGKRWIEERGAFYKWYWLISSK
DFDYRCREIHKLVDWFVYCRLQGGAKDTDPTLPDGRKKFILLNELAKETQDPLELRNESLSLLFAGR
DTAGALLGWVFYFLARHPHVYNKLRSIILDQFGPSPTSEISFQQLKACSYITHVINETFRVAAVIPLNE
RVAVRDTTLPRGGGPDGSRPIFIPKGRQVLIATYAMQHRADIWGDDVEEFRPERWEEGRKVGFEFIP
FGAGTRACLGQQFSHTETSYVIIRFCQRFDRIENMEPPGPIRLCHAIENRSGSGVQVRLHEAEKS*

>CYP539A53|10665|Clagr3
MLDLLTFHPILTLLTLLLLPTLYFTLTIRLESLRISRLGALAPHVPSPLPFGISIFLRSITASRHNTDYEFW
SYLFSHSPHPHSQTVETHLARQRFIFTADPENIKAVLATQFADYGKGEPFHEEWKWFLGDSIFTTDG
ELWHASRQLLRPQFVKTRVADLEIFEHHISKLMALIPPGGGEVDISALFYRFTLDSATDYLLGKSVDS
LRNPDTKFATAFAEVQSVQNEMSRAGPFQKILPKGKFWRGLRVINEFVEPFIDSALALNPADLKEKT
GQSFLHALAATGTRERKVIRDQVVAVLLAGRDTTAGALSFTFQELAGNRRILDKLRREILETVGERK
VPTYEDLKNMTYLQRVMNETLRLYPSVPFNVRMSLHDTTLPIGGGPDGMQPIGILKDTPIAYSTLYM
QRREDLVPSPSADFPPVLTYCPERWENWTPKPWTYIPFNGGPRICIGQQFALTEMSYTIVRILQRFDRI
EKYWQEETQGLRSEIVLSPTNGVKVGFWEAKSG*
>CYP52K9|10690|Clagr3
MHILNRADEAFGESHGCRPAPRLRNGWPWGIDRLLQIFEADRNSRLMELFLFHFEDVGNTLEQKFL
GTPAFGTIDPKNLEAMFSSKFEGSCNDNQRRISYSYQADFDYGLCRQIFYPLLGDGIFTQDGRAWKH
SREMLRPQFARQQYQDLDIFREHVDDLIASMSKSKQYVDLQPLFFRFTLDTTSVFLFGESAYSLRAN
QSAEDNKFAREFDIAQDYVVQRYRYLDLYWLIGGRRFQDACASVHTFIENIIDRRQASRDKNPDSQ
GRYVLIDAIAEDSRDRKALRDQLVNILLAGRDTTACLLSWTFYLLPRHPQVMARLREEIRSATGNNE
DLKREDIKKMTYLANVLKETLRLYPSVPVNTRTVHRTTVLPTGGGPHRSSPVLVRKGDNVAYCVYA
MHRRKDLFGEDASEFRPNRWEEDLPLYHDEINAKWGYLPFNGGPRVCLGQDFGLTETSYAVVRIL
QTFPVIRAGQFNRPQSQEWLGYSFHHKQGVPKLAKERQKMTLVMSARDGCPVELE*
>CYP534N1|6993|Clagr3
MPSTIALTLLALTALWALKIYIQFAQNLAAAKASGIKYTCVPFFVVSRIYQLACIVIVPLVRSLPKSWT
EPWFDMTLIDWEWARRYESFKRYGSDTFLLVTPERNVLYTADADVISQMTTRRNDFPKALEVYEVL
KIYGNNVVTSEGQLWRHHRKITSPPFSEKNNHLVFTETLGQCQDMVDSWMDGDKESSKTVHTVA
DDAMRLSLHVISRGGFGVRLEWPKKAKAAENGHVKGEEGNTSTASVPEGHTLTYADALGSLLHN
LLPVLIVPKFLLRNLPFQWAKTAYVSYDEWGNYQKEMFREKKASVLAGTEGDNLDLMIAMIKGAG
ITAESASQGEASRQTLSDEQILGNAFVFILAGHETTANSIHFCLALLAMNVAAQRHLQSDLDQIFQG
RPPSQWDYDQDVPKLFGSMAGAVMNEELRLIPPVIGIPKSTPKGSPQPLVVDGKKCTVPENTYAVIV
ASAAHRNPNQWPTGPPSDPEHPTHPLSNPDNDLEEFKPERWLLDPNSKSPATHMPDTDTDTEAST
IGFNTSADTSSLLYRPPKGAYIPFSEGYRSCLGRRFAQVEILAVLALIFTQYSVELAVDAYATDDEVAK
MSEGEKKVVWEKAKGEVERQMREDMRSVITLQLRKGSIGLRFVRRGRERFAF*
>CYP6456B3|3122|Clagr3
MEISLNISVLLVLAIASATTWYALDYFYVPKQLPNEPPLIGHHIPYTGHIIGLLRHGTRYYEIISANCN
LPIYTLKMLNMKVYVVTSPALVNAVNRNSKKIAFNPFIAQLGKRMTGHDEATGQIVQHNLNGED
GPGYVINVHDGTVASLAPGKDLEQMTQAMLKQVSPYLDGLAIDGEVNLFEWTKKMVTMCSTRAV
YGPDNPFNHDETLINSFWEYDRNLNLLIADIAPSILAPKGYRARNKLALAFQKYFENFDPSASQTSA
MIRARYTNATRHGITPLNQARLEVGTLIGILANTIPTIFYLLVHIYHDPDLLSDIRKELANQDLLGTPE
EIARNPKLLSMPELCPLLHSTFQELLRVHALGTSARYILDDVILDDKFLLRKGMIVQMPMAVMHND
PTAWGDDVKDFQPRRFMKQKNVYKKNLTVYRPFGGGASLCPGRHFVTLETMALAAVMVARWD
MRPVEGGWRVPGQKQESLATNVFPPERDIKVKVSLREKNSLNN*

Dibaeis baeomyces
>CYP6001J3 |12221|Dibbae1
MAEVAQNYETLGGSMLYKLIHRAFPGWFKFNSIYVMQPMYTPKMNQAIQKELGHIDDYVLDAPA
PPPKVTVLATNAAIAPILKDQANFKVKYGRQVPDLVFADYMLSGDSTGCRANKDFTSLRLNKCPG
AHDVYFKGFEDWTRKILTREAYKLGKFYQVDMSKDVARPVVVNTHAEFMNLPICKRGETTGGMT
EEEIYTYCTNYVNYANIDKDVAESWNLRRLARKHLKALTTATEELVKKTAHSTGVIGHFFGVAAAP
EGSLRAIGQKLTKDLLDSGLGVEKTAVVCSATAAGGVANLPTMDLKFVEALNWFLKDENKEHWA
TVQKLATENTPEAMDKLKHYVLEAARLSYVISLLRICVPADGAGQISVKDDKVYYDGNDMVVHA
GEVIACALTAALHDPAVFPDPMTVKLDRPTDVYQLWGIGPHTCVGREIVINGLTAMIKVVATLKN
VRRAPGSQAGRFSQYLGILVVAVI*

>CYP5076P1|200|Dibbae1
MRSVLQEYFAEEIDTRVNLDSLSVNAISTQLQQLRSDGEREQAWCLVYLETDFEDREARWATIKAN
LRIAAAKVGITLRTRIVENETAQTRERKNNRFPRKRRPVYDYSEDSEDGFPRRKRKRLLAPAPTPRTP
GTPADLIDKKCYLYTPSSGKSIKRSAIPTIRAIGKEQSKTSNKVPQKNSLLQTALSNLKRCQKSARRPI
PDGYNVWKREMPLKKAVAFRFFDLDSSGLNTAGGFLAGRFTDMENKECIDLQHTEDPVPPADFFS
DEFVESAFQHLTRVKKPTPFISLRENLLSVMHHGLISKTKACVAFVDLRYISLHQPFHFPRLFPGFKIS
QRLKELGKVQDNFYSAYGEWLVWDRIETEAVVACFSVDRFRRYMERYPRIKIVLRLEELEKCTGAG
DYKKYKRMLKKTALQLDLSLDRNSGTSIGHFLAFTGLSKINLKDVALKIGRNWKLIGSGEVKRKGPF
LEGVEDGFEEYFTDLGKNNEIDIDEFALTRKKIKATLGSKGKLYDTAGSHHCPMVGVEINQPVVDPS
FQMDLLGTDPGLIACARVWADRVAQADQMDRYGDVGCETVQEKFNTEPAPAELHLISRPDSRSPE
ISETDHDLQDHNDVTTLLSDLNLNNEHQLSNRSRNLTSLPSHSAQPSPNVQISLDRPRSTYKPGPNL
QFCLRGSTHQKTSNTQRYKNSLFKPFSPTSPGGSTGKHTNFSKYLYRPTQNSSKGANEIEVRREVIITS
KYFFPSKTSDQRTTASSHQLRKQQIISDSSGMAVNSMARGGLSMSQPTRRKLFEGEIAARAKRGNG
ADDDDLSAPELVVRSHSNIPRRIPPRIKNNSVRRAAVSKPSVSRPAESKVNLSVIKRFKRSTQDSKAK
NGAFSVVQLAANLGARVSRPDASKFAQSNHWRSAAAAEPGVEVFATRPPTSNNHNLRENEVSAV
VTNAMDWLSDYNGDDVLPSIETDVNNVEAWREKVFREKFEDTTSQTIADLASRRSSSATTGIIIKAE
RSLDAPMETHSRPPNRIIQRITLRESKVETEYVQSRSDEPQASTNQARVQVWDSQDAQRVPDSQIVI
QPSPGLALDFALPRPNAEEGEASEDEDADIENRDPGLPKLNTDKQEEIFTFTRREMDGFAAAAAGI
ASHLFYFNHGEHHMYAVRYIQSFFAVFAIAMLYGVVYCHQDAGEVLSYMLPFTACYLGGLYTSLL
CYRIFFSPSNSFPGPFGAKISKFWFSTQLTRHDAFKKIHELHQKYGDFVRIGPSELSIVHPKAVQAIYG
FGSKCRKTAWYDLLLPIISLETIRDRTIHDARRRIWAPAFKEKAIREYKDRLIPHQERLIKQIHANSGM
AINISKLFMLYGFDVMADLAFEESFGMLQSNEYHWAVKLLQEGLKPLGFILPEWFFRIMSVIPGLAD
DWFKFIGYTKERLYERMKVEKAATDIIDTTAGALTHIIYELISNPTCLETLRIELGPYLAPSVKLPPYED
LSKLEQLDSVIYETLRLHPTLPTYLPRITPPEGLQIGDTFVPGDTIVTCPPYVMGRSKSPHPGLDRSFDS
YETQHLLTTCLDAKDESAYVHAESFIPSRWTSQPSLVKERSAFAPFSTGPNSCIGRPLAMLNLRTTLL
RLLQTFDFGFAPGEDGRGVREETREHFTVEVGRCEVVFLERGKGMESEREN*
>CYP5076N1|280|Dibbae1
MGATEGIEKYQQHAQLFAVVAGVCLHLGYFYHGEHHMYGLRYLQASTFFFITSTSALTSGYELPLA
SSLSFTTSFFGCLFLGLYGSLLCYRLVWHRERRFPGPTAAIISSLWYSTQVTNVDAHKKTLELYNKYG
PFVRVGSSDLMISHPLGVPAIHGARASAGRRPGMTRAGLGSQSIQVEIINFTVNGEGRIVVYNKALID
PLGEHGGRPVNAAKWFNYYSYDVMGDLEFSKDFGMLKSGEQHFAVELLDDALSIQGLKLPTWIFH
LLTLQSDSRTIIVAGSDTTVASLSHVFYLLAKHPGHLVKLREELLPLVNSDGIFEHQKIHRAHHLNA
VISKALRLYPVPPTAIVRKTPPEGIVVDGPYIPGNMNIWTPQYVIARSEAAYERPFEFVPERWYSMPS
MVKESAGYAPFLTGPYGCIGRPLALMQIRLVIADAISRFDIAFPPGQDGSDFIDNTKDRFTWGLADL
NICFRARVGRHT*
>CYP53A64|460|Dibbae1
MFFSFLFNPIFLILVLPIIFYAFPYLKRWYLKDIPGPPLAALSNLWLMYQCRLGRRYKAVDDAHTKYG
TFVRIQPNHVSVADSEAIPIVYGHGNGFLKAEYYDAFVSIQRGLFNTRDRAEHTRKRKTVSHTFSNK
SIGQFEQYMQYNLQELVKQWDRISLDAVKSGKAWAEIDCLHWFNYLAFDIIGDLRELICQAFGSPF
GMLSKGADIAEVRKTPDSPVTYAPAIQVLNRRGEVSSTLGCFPQIKPYAKYLPDRFFRDGLEAVENL
AGIAVARVNARLEQSGESDRVDLLARLMEGKDENGNKLGRGELTAEALTQLIAGSDTTSNTSCAFL
YWATKTPGVIPKLQKELDAVIPEGVAIPDYDTVKDLPYLQQVINETLRIHSTSSLGLPRLVPEGEPVK
VLDKSFPPGTTLSVPAYTIHHSKKIWGPDADEFRPDRFSPENLTDLQKQSFIPFSTGPRACVGRNVAE
MELALIVATVINGWDWELRQKGELETREGFLRKPLSCLVGMKKRRRA*
>CYP51188A1|634|Dibbae1
MLVDVFWNLYTLVLNSRWWVIGALVAAAVFQDAFNASFMWWHRPRKIRIEGPSLWFGKWWSSL
KFITSAPELIMEGYRQSNEAPFAIPAREGYLVFVTSEEHIREIENSPIDQLSFHQAMEDRLKITYTFNGF
VMGPIDPHDAVPIRVLKTLLRKDLPQLRPKIQDAIEETFDFWWKHIATYHFSEELSLRVNCKIIFGED
LANNQDFFRAAHKYHRQALVAMLTSHYLPRWTDKYFLPSMMALGGAMKKLDGHVTRAVKQRL
QDMRQGKENIPRDGITWVIEASTTKEQQAVERIVQQVFAIFFASAHQLPMLLMFATYRLCGHPEYV
QPLLAEAEAMLKLPTADHYKSVPLMESFLREAARHEPLDSLSVQRKVLRDFTFTDGSVVPAGNVIC

VPQQAVMRDERHYDRPDEFLPFRFVNTANGSADGEDENGRGEDEVAIRKFTDLQPNFFLWGAAK
KPCPGRWYASAVMQQFFVHLLTRYNFRLADPDAKLTMTYTTIVFPRPGLEILLQER*

>CYP6196B2|637|Dibbae1
MPVALWNQSVAIASTFSTDYDVSFNYTSLIAIGLAALYCLWRLWRFSIRPFFHPDEPRELPYWIPCIG
HAISFFGSSHDLIERGLYALRNPPAARDYTGRTHEPFALRVGPETLYIITAPKDVAQVYRRADVLVW
DDHLNQIFLNFGFNPQSLKLAWTKPVKGDPTYLENNPVNPNQLPLIHLIENIYTKQLLPGVHMDT
MSEVFITSLQTALCWPKMELFALETNIGSSTVSLKTLCQYTLLEAGIYSFFGRTISQLDPKVIPTMLAFS
KNAWMLFYGLPSMFSSAVATPKRALIETFAKFASLPESQRSDQSWSVQQILIAQEIVGIDLNSRACM
LIMILWAANSNVNNTAFWVLAYILYDERLRHAVKQEVNAAWESGELDIKSLCVNATVLNSTFHEC
LRLNAGAMMGRRVSAPTRIGNKILKPGACILIPSRQLHSNEDVWGANHKDFDVKRFAGNEGRLK
HSSYRPFGGGVSYCPGRKIAKEQVFGLVAILIHRFDIAMSKGKRLEFPRIDASTPALGVLSPARDMDV
LVDLSHIGKHM*
>CYP5328E1|639|Dibbae1
MLGVNSAAVLVPLALLLGCASYLLYGLLTNYNAARKTGLPLVVLPVDCGNPLWLIIDRKVAQLVR
RLPFGSGTFTRFNWRGWEIWDRYRAHQELGDGIIFVTPGKNYLQLCDAEAVSDIFQRRGDFPRPPE
ATEMLNIFGPNLGTTDGQQWQRHRRITASCFNEHVNERVWSESIHQAIGMVRYWSSKPSINSVAA
DTRTVSLHVMSGAVFGKSYPFRGAGEKRPSSEEDSTSYGEALRVILDRCIPLVVLGRKNLSKSWLPKS
FKELYQATLVFQEHMTKAYEDEKRATSRGEKQVNNLMTALVRASQADANKKDTYTGSRQEGLTE
EEIYGNIFVFNFAGHDATANSLALGVSLLATRPDVQDWIADEINTVLAGVDSEESSYEAIFPQLKRCL
AVTFETVRLYTAVAIAKSTGSSPQPLKIGKSTVIIPKNTTVIPNYSALHTHPRYWGKDSLEFEPSRWITS
DRQPNEDVSGPNAALHAEKFKEPPKSNSPFVGWSGGARNCPGRKFSQVEFVGVLVGLFRDFRVKP
LLQAGEDDAMARARLLEQIRKDTGMRLLLQMLHPEKAVLQWSHR*
>CYP51F1|728|Dibbae1
MKLISILVEPLLEAYHKSPVLTVVATAFSCFVLIVLVNVLRQLLVKNPNEPPLVFHWFPFIGNTITYGI
DPYKFFFDCRAKVCDPYQKVVRENTERVYQYGDVFTFILLGKPHTVCLGTKGNNFILNGKLKDLN
AEEVYTSLTTPVFGEGVVYDCPNAKLMEQKKFVKYGLTSEAFQSYVPLIVDEVLNLMKVHPQFKGS
KGEVNVPAFMAELTLYTASRSLQGKEVREQFDSTFADLYHDLDMGFSPINFMLPWAPLPHNKKRD
FAQKKMAQTYMEIIKARKEGGMKKDSEDMIWNLMSCVYKDGTPVPDIEVAHMMIALLMAGQHS
SSATSAWILLRLATRPEILEELYQEQVSVLGADLPPLTYENIQLLTLNSQVVRETLRIHAPIHSIMRKV
KTPMPVEGTPYTIPTSHILLSAPGVTSKTPEHFPNPAEWEPHRWDALDAAKLLERDEEEVEKIDYGY
GLVSSKGAASPYLPFGAGRHRCIGEQFAYLQLGTITATIVRHIKLRTKSGRKDDIVGTDYSSLFSRPLN
PAVVLWEKRDPTAE*
>CYP59U2|845|Dibbae1
MANLTESRRDGTPFGGMWSFSLAFNAAATILLVSSVGYFFTKLYEARMRVRDKQKKGLPVAPGHN
FLFGHLLYLKEMLDRLPRGAHFQYAFGDIARQHFTEQGALYIDLWPLSGLFLATVSPQVAVQAAQT
NLTLATERPYLIKRFFKPIAGGPNLFDLPEEEWRPWRAVFSKGFSADHILSLVPGMVKETYTYCETLR
RLAQKKELFYLDPTTLRFTMDMIGRTILNTSLGAQRGKNDLADGMLSQIRWHQANAETNPFEYLN
VARWYMHWWNGRKMDRYIGNQLDKRYAEYRADSENTRSKAVIDLVLQAYIPEDAKSKPEKLDPE
FRAFATRQIRLFVFAGHDSTSSTICYILHLLSTNPSTLALVRAEHDKVFGTDLREVMSTLKKQPHLTN
SLPYTTAVIKEALRLFTPAGCSRQGKPNVCITDDQGNLCPTEHAMVWLIHVEMHRSPKYWVRPDE
FLPERWLVEPGHELYPMQYAWRSFEHGPRNCIAQGLVMTELRVVLTAVVREFDFQPAYEEWDREH
PSKGGQMYRGERAYQIEEGAAHPVDRYPCRVSIRKRGDT*
>CYP65FJ1|892|Dibbae1
MVQVDANEQDHARFRRLLSNAFSEKALRDQEPILQTYVNLLISKLHEQIDGPKKGVVDIAEWVNF
MTFDIIGSLTFGRSFDCLETGELHPWVALLPGAARTMTYLLALKHAPDLVFKALVILIMPFLSTRREH
QEFTNEKIKIRLSDNTERRDFITPILKANDEKGMTYPELESSINLLVTAGSETLATFFSGAVYHLSRNP
QVMKRLREEAQTTFHDTRDVTMAEVHSLPYLNAVIEETLRIYPPAALSLTRIVPPGGAIICSKKIPAG
TGVGVTSWAATHSPKNFTAPEEFAPDRWLDDPRFAGDDRQASQPFSMGARNCVGKNLAYAEMR
LVLTRLALDFDVELQPESKKWNLQKLFTFWEKPALMVKVIGMREL*
>CYP5039E1|895|Dibbae1

MTTWYEAYYDLWLGGQYVWKIGELHKQYGRFFPKKADRVLFRLLTSVNKGPMIRINPHEIHCNDP
EFIDDIYAGPSRKTDKYRFTGRKTLTKQSMVATIPHEIHRKRRGAMANFFSKSSVRAVEPIIQNSLRK
LLWRMERASKTGEKMPLMYVFKAATSDIITKYAFGKSTNFMDMDNYNMPFFKAIEVTFLMSPALM
HFPWLGPLMEALPQPITRMLMPGLADMYRMREGWMAQIEEIKNSKDKDAGVGTIFDGVLKSKLP
EEEKATARLGHEAQLTVLAGQDTTGKHMHHYGTPDEAETSIATTLSSAVYELLANPDKLKKLRDE
LIATFPDPDQPLNFSEVEHLPYLGAVIQECLRCHPGVITRMARVSPEVPVIYDRNGKHYTLPAGTPM
SMTSTHVHFHSDFFDNPREFCPERWIENPRLDKYLIAFSRGTRNCLGINLAYQELYTLLAGVFRKYD
VFDGTGTQKMPTLELYDTLRERDVDMNFDYIVPFPAKGSKSVQVLVRHWVAEEAKCEEGR*
>CYP5039D4|898|Dibbae1
MDPIKQTFAERGFSAVYLVAVLLGSWTVYLVGLGLYRGPKLAALTTLYQAYYDIWLHGKYFKQIDQ
LHQEYGPVVRINPHELHINDPSFIDELYTSGAKRRDKYKWAGRSVLLPDSLVATEPHGLHRMRRAA
LNPYFSKATIRKLDPVIRNGLAAILRRLHDCAKSPSIFHASLAYKAATCDMITEFSFGVSTGYIEKDDY
EDSYFKAVDDHLNMAWMMTYIPWLGPVMNSLPPAFMGVIYPGLKHLWSMHSRWVAQIEKIRAL
DKRDSEGTVFHGLLNSDLPASEKSNGRLRQEAQLLVLAGQDTTAYSLSALTYQLLANPEILRKLKEE
LQRALPDPDTPMTSAQLEQLPYLTAIVQEGIRLHPGALVRQTRIAPDQTMIYRDADKKKEWIIPPGT
PVSMDARGCNLNPKAFADPHKFMPERWIDNPRLDRYMLSFSKGTRICLGMNLAYSELYMILAGIFR
RYELYDGTDKQTAPTLALHDTMHERDVDVIHDCLVPFPAKGSKGIQVKVRAGTEL*
>CYP65DY2|1002|Dibbae1
MYAKYDSSWVVANQLLRLMSTSILANLTIVIILALALRILYNIYFHPLSSFKGPVYLVASDVPLALLQL
RGTSHHTLAKAHEKYGNVVRVAPKTLSFIEPSAWNDIYGYRKGRAVLPKDPLFYNEMLLDKETITL
ASDDDAVPIRRAINPAFSHKALLEMEPMLLSHIDRLMTQLAKTNQEQGSVDIRQWFTFSMFDINSD
FAFGEDLGCVKRGEYHEWVQFVVEYFYAATLIHQCHKFWPLNRVLASLIPPSVRDKKERHSEASLQ
RVRRRMKTPTDRPDFMSHFLRHADKEQLSKSVIEAQATVIILAGSETSAVALTAAAYHILSNPDVHK
KLSDEIRSTFATSAEITLQTVLSKLPYLDAVIKETLRIHTPLANGFTRVVPSDSSGTVISGNWVPPNTVV
TINHYCANMSVRNFRNPHTFIPDRWLNDPTYDGDKKDVVQPFSVGPRDCPGKRFALNNIILTLAH
LIWNFDLELGDGTQNWTVDQRIFNGWVQPALPVLLEKRA*
>CYP61A1|1425|Dibbae1
MATIMNGSFASPLADANYSPPSTFNGGLVATLTSGINGWSMALTGLLVLVAYDQVAYIWNKGTIA
GPTFKTPFIGPFLESVYPKMERYKEKWASGDLSCVSVFHKFVVIASSRDMARKVFNSPMFVKPCVVD
AAHKLLRPTNWVFLDGKEHVDYRKGLNGLFTRQALEIYLPGQEEVYNKYFTRFLEISQKEAAGKPT
PFMPHFRELMCAVSCRTFVGHYMSEAAVKKIAHDYYNITAALELVNFPIILPFTKSWYGKKAADM
VLDEFAKCAAKSKVRMAAGGNVTCIMDGWIKTMLDSEAYREKIEKGIQVDPSTKPNPLLRLFSDFE
ISMTVFTFLFASQDATSSACTWLYQLMADRPEVLNKVREENLAVRNGDRNVPFSMDLLEKMTYTR
AVVRETLRYRPPVIMVPYIVKKSFPVTETYTAPKGSMIVPSVYPALHDPEAYPNPDYFDPDRWITGTA
EQHPKNWLVFGTGPHYCLGQTYAQLNLMAMIGKASMFLDWQHHVTPKSEDIKVFATIFPQDDCL
LAFTERSA*
>CYP676C1|1698|Dibbae1
MALEQILQAATGVQVTGLLGLAFCLSVVVYYRYLCPISSIPGPFLSSFGPLWQLWQVFNGHFEEAVT
KLHEKHGHFVRIGYNEVSISHPDALHDVLGAPLRKGDLYLPFAIPNISYNNLMSERDYKKHSSMKA
NVSAGFSLSNIIKSEPYIDRAIELFEERLSQQNGPVPLNLWISFLTWDILGESIFSKRFGFLQEGRDIGGS
VANTYMLALYVTLAFSLPWLHALLLGNPILRWLDFQPSMHTFDTCMAAVEARKINPEVRDDMME
QWLNTWRKHPDRMQEKEVLCAALSSMGAGGDTISSALQAFVYLLLKNPTHLWTLRKEIDDAHAR
GELSKIPTQAEAQKLPFLQACIKETLRVCPSVPWNLPRVVPKEGLTIAGRHFTSGTLVSVNPYVIHHS
LSIFGPDAHTFNPYRWLIADPNASKSLERYLIPFGMGYNACPGKNLAMLEFNKIAATLVRDFEILEA
EPQTSWRFRTVFIIVPGKWKCFLRRRAVSMRTGGEQVQKREEVGTKEVGPAQVHADDVEEKMKVE
KKEETDEAKATKNIETGENLEQRKEEKSEENAGEVDDADTQAPETPPAPAPCENEAANPDPPNASI
PT*
>CYP5168L1|1969|Dibbae1
MFFWRTYAESTATWFGLVRVLLSELSGALVCCVYTDLGRKGPNEVTIFDPSILPLIYEDSENKFTKDV
WLSTIIGVELGAMDLLQRVEALLALKVDSSDAEIKPALENYSVQIQKYADQLDQYIEKHASQSINAT
QCFYWFSFDVMGRFAFSRSFDLLQNEQWDETIESLHSGMSLMGPLSPVPWLVRVIFSFSFLPDVRKW

NRMNRWIAERLDERLERTVKEPDISHYLIEWSKNHNSLAADMHLLNGDAFTLIVAGSETVASTLVY
IFYHLALHPHHLSLLRAELSTIPNVRDPKALQSLPHLNGVINETLRLWPPVPTGGLRQVPPEGIMIDG
HFLAGGTSICAPRWSIGRLPSAFPQPHAFIPERWSSQPHLITDRRAYAPFSLGKMNCIGQKLAFVELR
VVTALLVSRWDVRFRDGDAARKCIEGTKDCFSIVPGPLDLVFERRKDI*
>CYP548A29|2252|Dibbae1
MLNYNNTKSVAESLASTLAIVLAVGSIYVFGVIFYRLYLHPLAKYPGPLLGRITDWYSVWQAWNGS
RHLDFYRLHQKYGDFVRYGPNSVSVNTDTALQKIYGFKSNVKKADFYSAFPVNPGAYSTHSAIDKN
LHARKRRVLSQAFSDNAMRGLQPHILHILRKFCNVIADLPVTTTSYENKPKTWSSPKNMGQWTNY
MSYDVLGDICYGKSFETLERDDNRFAVDLLARSSKFHYLNAQMPRLKKLGLDRLLFPKIRADRERF
MAYSKSRLSERMKLGNDTGRRDFFHYLLNAKDPETGQGFAVPELWGESNVLLIAEDALNVRLGSD
TTSTALSACFFHLLHSQHALARLTKEVRTTFSQLEDIVTGVQLSSCHYLRACIDEAMRLSPPVASVLP
REVLSGGIDVDGEHFLEGTVIGVPAYALHHKRQYYPDPFAYRPERWLVGDFSNENVAAFTAEDVA
LAQSAFCPFSIGPRGCIGKGVAYLELTIALARVIWLYDMRLTEGEDVGRDSDGGYTLIDCFIAHKDG
PVLEFKLRV*
>CYP682AU1|2447|Dibbae1
MAVPFIHDISISTAFGVLGAFLLSYAVFGAIYRLYFHPLAKFPGPKLAAVTLWYEFWFDCILYGQYTF
EIGRMHEKYGPVVRISPNELHVNDPEFIDTLYAGPGSRRDKSEFYCNAGAMPGSTVFTVGHDLHRM
RRSHMSMFFSKASVRRLSPIIRGTVDKLCAALETHRNSGKPVVISSAYSGFAIDIITEYCFARSYNLLD
DQTFERSYHSAFSKARSGMHWIRHFPWMFNVMKYLPRSVAVRINEGMIALRELQVELRAQVAEIIH
EKEKGIKPSDKSHATIFHGLLDSSMPDEEKQLSRLGIDGFSLVRAGTETMGSSNGVIHEGLRLAYGV
ATRLQRIAPDESMVCGDWVIPPGTSTGMTAVLVHQNPDIFPDPMEFKPERWVENPRLDRYLLSFSK
GSRICVGINLAMEELYIATATIFRRMEMELYETDLTDIEVKYDNFTPKAKLDSKGVRVLIK*
>CYP617AC1|2631|Dibbae1
MGLNAVAYTAVAFVTALLCLAAGVEYLLGVKNDPREPQYISPRIPIIGHLIGIFRDKNLYYVKLRSRF
PDLPIYTLNVLGTKMYIVNSPDLAVAVQRNSKTLSFSAYAVKLAARVCGLSSQATEILADNVGGEK
GPGGFSATFNGVVHSKLALGPALEQLFQVTLPALAASVDELATRSAAALPPEPMALTEWLSHTITIA
ASDAVYGPGNPLRDRATEQAFWDLDAGITSLAISPLPELLAPKAVAGRRVVVDAITKYLKSGDQAA
ASDYLQARYTTCFQFGISLEDVARMDIGNLSSLNSNTVPAAFWMVVYIFSTPTLLDSLRRELEKVLVT
TLDNSDGTTKRTLDITTVKEKCPMFVSTFQEVLRLRALGATIRLVLQDTILQDRHLLKKDGVVMIPA
PALHADAAIWGPNATNLDPYRFVKRNLSLPQAETAAQNLKAKIPNGAFRVFGGGTSLCPGRHFA
VTEVLCLVALLILRFELKPEPTVEGGANNWRLPAPTTRNMAAAIPFPEHDVSIYVSTRKGWEKGSW
QVQASDLRGKFDVANVTG*
>CYP5241B1|2999|Dibbae1
MSKEHLPQLPRGDFSVLSTLSQSFSFHASPESFISARIRSAGAQTLINQLDDEEAGHSKPSIVRARILGR
HVAIISSHRFCEAVLQLSGGDGGNPITIRAAKPRESLSPDSFVAKAAYQELMADFFPPPNILLMDSDV
HRAKRQAWNKQLESFPTDIAPLAVDIAKDHFQSWTEGRAIDLYESMKDLSWRLLLGIFLQLNPGG
KDYHLIESLQETLLRGQFSLFPVAVRAPLWRSPRYKAVEARKKLEGLLKEKISLQADYCPFLKQGRIE
KNEVASHAVLFTSSIAVKALSSLLTASLLNLFLLPSGVPPAEDIRKQDLETGQILLQSVLLETERLSPPV
VGVMRRAQQDVIFTNGGDEPDTLIPAGWDVWLYFVGAGRDNAVYPLADKFLPERFIAKGETNHG
FAFGSGFKCCLGSPIIRKLVHVVASVLIKSELKLEGSVEAEGVRGWLGWEVNTSAEAFARDLKQLPC
QRPKNPILIRIEHKSK*

>CYP5128L1|3127|Dibbae1
MAFLSPISYNESVHPSWLLLLLFPLTFLSLRTFYRVYFHPLRSVPGPFLAKSTSLWLAYHGYVGDDCIT
IRRLHSQYGPVVRIGPRDVDIADGEALWPIYMDKGGFPKPDYYKTFDIGPHASIFSSILPGQRDEPVK
AVNPIFSMSNIRSAGNVISECAEKFVNRLLEASRSGQSINLIDHTRAFALDAVSSYVFQSNYGSLEEKS
DGFSAAECVDMFISDSRWYYLPDLLTRLIQRYMPYLLPDTKATQSEHLVDQYLWGIISRGKDGKCD
GSYQSRMLERNITETEVFSQCKDALFAGTDSTGNTLAQLIWYLVRNPEKYARLKVEIKANRDGQTD
IMNLPYLNGVVKEALRLTMTISIRLPRLVPESGWSYHGYHFSPGTVVGVSAYQLHLTEAVFPQPFDF
LPERWLEATPEMNRDYLPFGKGARSCIARNLAMAELCVVTERLVSKDLLNGAKSDQEIIEKYEWFF
SRIKGDKIDISWPKKVQ*

>CYP50230B1|3162|Dibbae1
MSEPTYTVFTLLNCTKTNLDYIPRIYGTKDEFLKTDAYAAFAPRVNGKAIPWWISILDREQFGVMKK
PVVGFYTPAAVIKYEPLVDDMLKKFVDRLKGEINGETSKNLDIAPWVRYFAGDVIANITFGDGLGFL
DAGKDIDNFLSHLDENNDEIALYFTMPWSAQIFKYNRIAEYFAKDNRFYLNWSLKQLQKRFGSISE
KQGTPDTKQTKDLLDHFLTAAQIPGSSPMNYNMDNIMNWTTSAIIPGSETVAIVLRTLIYYLLKNRS
KYDKLYEELRSAELSYPVTWKESQQLPYLDACVKEALRIHPIVGQGLERAVPESGLVMPDSYVLPKD
TRVCINPWTVSQSSIYGENLEQFIPERWLQRTHETDLEYKDRIVKTRRADFAFGGGRHQCPGRNLAL
LEIYKCISTLILSFDMGLVDPAEEWHLIDRFVVRQENIRCWLKTRV*
>CYP684M2|3389|Dibbae1
MDRRTHEIHPALAKEGPPFATTIIMLRSVFVGPVVRIAPNEVDVSDCEAAREIHRARGGFIKSSFYST
AGRVQSMFSTADPSFHSNRRRLLGPCFTEASLSTLEPVVLERVRLAIRKLGEESKNAGSTDILKWWT
LLAMDIIGELCFGESFQNLEAGKKNQYATDIANAGSILPLRTAFPTLIWIAAYLPLPFFKNIAQARMR
IFSYNKERVARYIQVVENNPDNTKKTLFTSLLKGGSKDLTELDLTVEAQSYITAGTDTTAVTLTYLV
WAVCRDRKIQDTLVRELQSVPENLTHQDVRDLPYLNCVVEEALRRYGAAPGSLPRVVPPEGATLA
GQHLPGGVVVSTQAYSVHRDPEAFPNPEKFDPSRWENPSKKAKDAFMAFGTGPRGCIGIHLARME
LRLAAALFFRTYPHARISTKDGMSDRDMEENISFLLMPQGHRCLIEV*
>CYP65FN1|3450|Dibbae1
MVAVTPELLLAPKQSISLGYVSLGLGILIFVYLFSGIIYNIYFHPLAKYPGSKLYTATFLPYLVDGWTG
NLKVRNQQLHDRYGPVVRFKPNGLSYITEQAWKDIYGTRHGKKQLAKDEDFYARLHQTPDIIVSN
DADHSRMRRLISHAFSEHALREQEHLILHYFDRLIAKLREQIDGPTQGKINAVRWYNYTTFDVIGDL
AFGESFGGLEKGGFHFWIDVFMDSFKFHQFLKAMRVYPWIKVLVPIIAKFYPNLPHAREKHAEYTK
ATIGKRLNSTAPHPDRKDFVDYILRYNDEKGMSIPEIERTSEVLILAGSETTATLLSGATFQLLKNPRT
MEKLTGIIRSTFKNEDEITFQSTAQVQYLHVVLEESLRMYPPVPEMLPRKTPPGGEMIAGQFVPGNT
AVGVHQYSAYHSPTNFHLPNSFIPERWLPAGPDNPDAHRFAADNRNVLQPFSVGPRNCIGKNLAY
AEMRCILARVLWNFDLELCPGSQNWADQKIYTLWEKGELWVRLSERKRE*
>CYP59Z1|3598|Dibbae1
MSTVPLNIHTQYIPLAVRVKNLDLPPVFYLDTWPLGMQILFLSTPETCYQVTQERSLPKDEVLATFM
KPLLGGNDLVTMEGNEWKTWRNIFNPGFSTGHMMTLMPNIVQEVSIFCEILREHAVKGDLFQLEE
VATRLTVDIIGKITLDTDFNSQRGHSEYLTAFRTLLNWLSSGDEINPFRRWNPIRPWVQRYYSRILDR
YLDGELEERYAGYQKLGEDVKTKRNKSIINLALEAYRAEHGQSKSTKGLDPTFKKFARTQIKTFIFA
GHDTTSSATAYMYYLLSQNPDCLAKLRAEHDAVFGPDIGTVGAQVSANSHLLNKLPYTIGVIRETL
RLYPPASTIRAGAKDIYVKYDSKLWPTEGMMIWCVSLPLHRDPNYWPEPMKFMPERWTVPEGDPL
YPVKGAWRPFEFGPRNCIGQELANLELRLILAMTAREFDVVPGFEEWDRLHSKTGVNHFHGERAY
QILIGSAKPVDGMPCRFKIRENK*
>CYP6003A4 |4288|Dibbae1
MAAADDLRTLGIRGTLAATAATAAYLLYSNRNEISMDSASKKRKYQAGEEYEDPKVLSTTLFQDLK
AIGFAEGKSQKEIHALVETIKNTGKPLDDKKLDTENLISLLTKLPTNSASRKALTNKLLDALWDTLQ
HPPLSYLGGDTKFSTPAADPVVKSGELGGAPKLDPAVIEYVDPDGVTIRQSVPDAPSGIFQYRTPDGS
YNNILNPNLGRAGTPYAKSVRSGKKLHGVRPDPGLLFDLLMKRDDQKFKENPAGLSSMLFYHATII
IHDIFRTNRLDSNISDTSSYLDLAPLYGSSLQDQLEVRTMREGKLKPDTFHEKRLLGQPPGVNVMLV
MYSRFHNYVADILLKINEGGRFTLPDTKTKEERSKALAKQDNDLFNTARLVVGGLYINISLHDYLRG
ITNTHSSDSTWTLDPRVEIGKQFLDDGTPRGVGNQVSVEFNLLYRFHSAISKRDELWLDDFFDKMYP
DRKGSLEELTIQELAVGLAKFSASVSKDPSQREFGGLKRGPDGKFKDADLVKILQESIEDPAGSFGAR
MVPKALRMVEVLGILQARKWQCASLNEFREFFGLKRHEKMTDINPDKEISKILEDLYTHPDMVELY
PGLFIEDVKPMMATGCGICPPYSVGRAVLSDAVTLVRSDRFNTIDFSVANLTNWGMAEIDYDVNTL
GGSMLYKLIQRALPGWFPYNSLHVMQPMFTKNANRKIAQKIGTYKWYTEADPAPPRPVTVLIKKS
EVAKVLKDQKKFRVPWLPAVTDMRPDGRKVDWYMLSGDGPANAENRKMMSEVMSSVKNLQKA
VSTFISKSGSQILKDATFEMRPGFCQIDVVRDIAIPLCAGLVGDLFYMDLCNSENPQGTLGPSDLYKH
LLNIRVWGANNNDPGLAWNRRRWASEGVEIICKTGEELVRSVQAESRTRGLIELVTSVFYPKYRHR
SAAIKEGSLRSCGHRMVQEMLARGETPERIVDNGWLNAFGSVGVPVTAFSEILQFFLLPENEKLWG
QVQDLASKNNEKSDALLRKYVLEAQRMTSHQRNLRIVAETTVIGDKTLQAGQAVVLMIGEAGRFE

DPSDKEAVKDPKVFKYDRPDSDISHFSFGPHACFGREIALSYVTGLIKIIAGLKNLRPAPGNMGLLK
WIQVGTERCYLSDNWSYLTFDPTTWKLHFDGTGKGVYHAPANASPQGSLPKAESSLIQVENVLRR
QKREVMVEVEKTEGKCHAQV*
>CYP65FR2|4578|Dibbae1
MFWLRNVADSIWLLVGAICIVYVAQRVITAIYALYFHPLSRFPGPRFAAISYVPYAFHLANGTFTNW
VKEVHERYESEIVRLSPHDLSFISPAAWKDICGGAGHRSFEKDVAVYGKSPNGIDTLLTAPKPDHSR
MRRVLDHAFSVKALRQQDPTVQNSVEKLIRCLYEQIDGRARGKVNLSDWYSWMSFDLIGQSATSL
LLANRREWIHTGDLAFGESFNCMENTANHPWVQMVFGNLQGIVFLNACSRFPIFASLLPYLIPKKI
KNMMGDHWGSTQAILAKRIAKSTTRPDFITPILENNSSKGLTPGEVEANASLFIMAGSDSIATSLTGL
AWYLIKERDIMDKLRNEVDRAFHKDTEINIQSVDGLPYLCAVVQELFRIYPVALAGQAHIVPPEGA
NICGFHIPGNTGVSMNQYAVYRSKVNFHNPDIFDPNRWLNDPKYAGDRKDAFHPFSFGPRNCIGK
NLGLGATRLVLAKMIWHFDMDLSEETDMNWLDQKSFIVWQKKPLFVKLNPRRSSRPPDIIK*
>CYP6120A2|4640|Dibbae1
MNHEWHKKYGPIVRIAPNHLSFSDHSVQKIIYGFGTRTIPSFEKDPRFFTPEVDHSKNIINECDKEEH
ARMRRMLSFAFSTSNLLDNEDIVIRRSNEFLEMINGIKTENGKTGMNIVQKFNYVTFNIMGEMSFGD
SWERRLKEQAGWYLFSILRISLMYAFTEHRYHWADVIVDSTYWNDVMRAVVCIPGLFSLMEWFPPT
RSRETLYRHAEYATEHTEARLKLYTSRKDFVYHILSSKGPSATNKEIASHFNVIMMAGAVTTATFLS
GVLYYLCHNLQAMQRLQDELRGTFPDIESIKSKELMQCTYLNAVVEEGLRIYPPAGAAHLSRIVPKG
GVEMAGRFIPGGTRVSVHPWSILRNPNVFHMPEKFIPERWIASDPPGQMGDKLDRSLPFSYGPRGC
LGRNLAYLEMRMILAKMFWKYDIVWFNGDEIDWERDTKGYTLWEKPELRCIVQERSMPGAQ*
>CYP6187C1|4764|Dibbae1
MDALPDRNRLVLFSSALISFTLVYSIGLAIYRLFVHPLAKFPGPKLAAVTRYYEAYYDVWERGKYLW
KIKDMHARYGRALSFTFSSFSMQYTVSAIVQTGPIVRISPYELHIDDPDFHEKLFRYDGRWEKYDWYI
RAIGAPYSVQLAVDHDLHKMRRAALNPFFSKQKINSLEATLQAQIEKFCKRVEDFAYSGKILPLSYA
YGAFTMDVVTEYAMEKSYGNLNREDFGADLVGSIKGIGPMWHFGKHFPWLLRHLLATAPSWLLE
RLVPKSSQWTAFQENCLTQLRKIISASTTASEAIASKSHRTVFHELLSSDVLPPHEKSESSLRDNVQIVI
GAGVETTAHTLKVITYHLYTNPTLLQNLRAELRTVQHPGSSVKLAQLEKLRYLTAVILEGLRLSYGV
TARFIRIAPDHTITYSPSSYSPSSKTWSIPPGTPVGMSTLLTHHNESIFPDSHTFKPERWLEDAERRRLD
RYLVSFSKGTRNCLGMNLAWAELYLCLAAVVARFEFEMAEGTGLEDVEVSSDQFVPAMRGAGRL
DVFVKRVGV*
>CYP59AD1|4974|Dibbae1
MYLQISSVSEHPVRNAFFVLVAIPIVAFCVELYKKRRRSYGWKKRGLPMPPWNPLFGNIPTLIKVMS
KIPRDAHGHYAPYLIRKAFPDLNQTFYLDAWPLGPQMLVVGSVETAYQVTQENSQPKHPSLREYM
LPLTGGNDLVSQEGQTWKRWRNIFNAGFALSHLITLAPGMVEEVEIFCEKLGEHADRGEVFQMEE
ETTGVTSDVIGKVAIDTRFNSQRSKHPFVMALRNQASWLVFSNEIHRFHPFRPFTLWWNRRVMDKF
LYGELERRIATYEERNAQTGSTTRSRTIIDLALDTYLLDESTKKSFKKMDTTFKDVAMNQLKVFLFAG
HDTTSSTLSYVYYLLSKNPSCLARIRTEYDFVFGPDSSEIASLIASNPQLLNRLPYTTAVIKETLRLFPV
AATPRYSPDPNHTIWDSSGHVFPTEGCIILVMNQCIQRDSKYWPSPNEFLPQRWLASPGEPLYPEHK
GAWRPFEYGPRNCIGQEMALLEIKITLAMTLRRFDVVDAFEEWDRRKGRTGVRTVDGERAYQVLV
GTAHMVDGFPARVVRR*
>CYP682AY1|4997|Dibbae1
MSTTTSLSSLLAMLSPTLLLTILPLIYVLAVVTYRLYLSPLAKFPGPKLAAATFGVEFYWDVILRGRYE
WVIRDMHRKYGPIVRITPDELHIDDPDFFDEVYPGTAKQVEKSKAIAGVFGKSLSIIATVNHDLHRK
RRAQLNPFFSKQTLMKYIGSVEAFLEKLSDRLEASRISQEPLNLRNTFSALTLDVISLYSFGEEYNCLE
MPDFGESLHKGIASAGELALLLKQYPQFFELANMMPYWLVRRLDPNTMSLIEWRDDLANQVRKV
VERYSKGESPKHASPTIFNTLLTQDVPPLEKSMDRLVGEGIALIGAGSVTSAQTMATCAFHLLSNPAI
LQHLRHELDTAFPDPAARIELIKCEQLPYLTAILKETLRIAGGITHRAPRVAPDRDLNFRGWIIPRGN
VVSMSWHATHMDANVFPAPLEFQPERWLPKDYSEEKAGKTTSSPLDKYLQPFGHGTRNCLGLNL
AWAELYLGIAMVFRRFELELYGTTRADVDIAHDWIAGAPRLESKGVRVVITGRRG*
>CYP532A33|5125|Dibbae1

MIDSIISLLLEHWLFAVAVFVVAYFARNCFYNGLQKYPGPFLAAFTDWWRFWDVLKRRPDITQLRL
HRELGDIIRIGPNTLSVADPKALKIIYGLNKGFVKSDFYPVQQAMSKGKRLPSLFSTTDEQYHANLRR
SVNNAFSMSALVQYEPMVDDTTTKFLDQTEAIFASKNEICDFALWLQYYAFDVIGDITYSKRHGFID
KNEDIDGMVSFLGSIFGYVAPVGQIPFLDLLLLKNPILRALDYYGIMSSPFPVVKFAKARMAERLSSD
PEKTPSVARGDLLSMFLKAKEDRPDFFTDDRVLVMAVSMAFAGSETTAISLAAVFYYLLKNPAYFQ
KLVAEIDEAVRNGDIEDRESCIVTWTESQKLPYLDACIKEALRMHPAAGLLLERIVPRSGITICNNQFI
PGGTIIGANAWVIHRRPEIWGADCDIFRPERWLEVGVEKRK*
>CYP6102C1|5261|Dibbae1
MEEAALAYDEHWGLDTDNWRNVGIYDTMQDIIAQISCSIFIGPPAYRLLKSRIGRSKNFLRHAIGFA
RAVGTSALLLKPFPDFLKPIFGPMVTFPNRWHYWRSGKYAIPIIKERLARFSERQKQPKTKSTEPDDFI
DAHIRFASESPNPELEMTPEMIWLRIIGVIFTSLHTTVFTATNFILDLASSPPERGYIEVLRQEAEQALA
EEGGKWTKAGVAKLVHMDSAIRESMRLNGIVTRGPERIVAAPNGVTTPNGLHLPKGAHVGIASYG
LRLEGYSNAADYDAFRFSRMHEQASSAPDASGYKNTDMVHTSDTFLQFGHGRWACGGRFFAAAE
LKLMFAYLVLHYNIKPLETRPPGWWTGDTLLPPLKATVSIQRRASSTV*
>CYP682AX1|5292|Dibbae1
MFQEVPYYRLAAILLGALALYTVYGLVYRLYLSPVAKVPGPRLAAITFFYEIYYDWIKDGRYSWKIAE
LHEKYGPIVRINPEEIHINDPDFYDEIYHGPSQGKTDKWERSVRIFESPNQTVATVGHDLHRARRSSY
AFFFSKQSVRAMESSINACIDKLCSRFEQSRRKGEPVNIKLAYSALTSDIITAFCFGESYNLLDNPSYAE
PLCDLIDHSISENIVIFQYFPWVFLLLNALPKAM
VKAISPGFALLIQYQGKWKAQVREVRERNVDLSEKGELVEKDASKTTVIEAIYNSKLPASERSDSRM
MYEAQTMVIAGTATTAAALMVSTYFILSSPSILSNLLSELASVSSNPSDVPSLAQLELLPYLNAIVYEG
LRMSYGVSHRLQRVSPDRTITYGKYALPPGTPIGMTSIHIHDNPTIFEDPRAFRPERWLPLETTGLHL
QKYLVPFSKGSRSCLGMNLANAELRLGLAQVFLRFGKSMRVVDTVRERDIDISRDGFNPLTAKESK
GVFIMLDGKE*
>CYP6103B1|5457|Dibbae1
MSRKDAHTTFNAISKELHHRKRKLLAKAFSSSSLPGYEPALVLQFGNLVRKLGGEATDAEPVTRKV
FNVAHEFHCLMLDVMGNLCFGAAFGFLENESNGVITSIQQRAKRILMTGHEPLFKRYKLDVLFFPH
LFLANTALIKYAQTYALPRIQRHRNIDEKAALPIQKTSGDFLEHLISARDDETSTTYSDVELLSESLML
MLAGSETTATALTSTITHLLSSPSSLSTLRAELSSTFPTYESLSPAATESCTYLRACISEGMRLCPSVPSII
PRIVGPGGLKLLNGTHFIPEGIHVGVPNFTLFRNEKYFSNPHAFIPERWIAGSSEPPFTEESVKRATSA
FCPFSVGPRHCVGKTLAMKELSYVIAGFVYLFDFEMEGEAVGGDVGLLGKGRHLLGMQGRVLLEQ
RDVFTSVEEGVMVRVKVKVRKEMGTYM*

>CYP660J1|5618|Dibbae1
MNSLTFLAAAVAAVIVYNVYNYFFRSRPDPREPPMIPSSIPYIGHTINLMLSGSKYFASLRCEKYPTLP
VYALQFGTQRMYVVNSPSLLHSLERSSKTIQFTPFVRIAAERLSNIRGRSLEQFDGSEKGEGLSVAEIK
FTKHAILHSTEDLNLQMLAPLGDLLGEPLQDQGKPRKLFHWIKHAVTLASTKAAYGPLNPFDDPK
VEAAFSEFESDAALLMLNIAPSITARRGCRARDIISNAFLKYYLANGHEQGSVLAKVRHDGPLEFGL
PEGDIARLEVSFLGALLSNTVPTAFWMLVNALSKPELVAELRIELTDAIRTSKEEDKQTRHTIQVSHL
KQNCPLLMSIYQEVLRLQSVLPNTRTVIADTLLNDQFLLRKGAIVQIPTSYIHRDTSTWGPTAREMD
LRRFMPKGSNDGKGGMKEEEFVKPAQTVMRTFGGAPHICPGRHFATTEILCTVALMVLRYDIEPVG
GKWVIPGWIESMFSSVAPPKEDVEVVIREREGWHGIWDFEMGDPKIRFQLALA*
>CYP613C7|5651|Dibbae1
MTPEVVLTDSEQLKAVFRDSDKHVKAVNNNSGYLMSQILGQCVGLISHQSWRDVRAIVEKPFLRTS
TSNHFSLIWNRTQAFLDKLCASRILPKGLVHPAEDLKLLPFWIVAEIIYGELTPAMEASLIELVPLRED
LFKHVIAGGLSRFSWFKHLPTTGNFLLREFQKKWSIFNEQAYKHAVSLQNDAPIIEMFQAVKDKKIT
LQQLYQTIDEMLYANLDVTLGGISWNLVFLAANNDVQARLRAEASQWHIQVLADSSAASQYYLD
SSSYLAACIYESSRLRPLAAFSVPQAIPTARTISGYTFPAGTNFIVDAYALNVRNPFWGPDRTAYRPER
FLERNATEMRYHFWRFGFGPRQCMGKYVADLILRVLLAQLVQGYELALLDGMEKDWARNGEMW
ISHPSMDLRCKKRENPKSEE*

>CYP51181A1|5661|Dibbae1
MTRLLSGRPSRYVSELNGLKIAALLPHWIPFVGHGLWFALFPEKLKHVRDVTRDGMFSLSMAGYTH
SIIVTPSLAKSLLQQKLNVINNHSLVWYLIDNFHGIAPEVHQIDPSIIFKEIHSVHHILMKESYLEKVM
SQVKAAIETHVADLVSFDEKHFRHRMWQDGGDGKLVKDDPYSMEISLFPLIRNFVSLLTTPILMGT
DFMRNFPYTVEDLWEFDAGMLFFLLKMPSWIPSKKNRNAHAARARILSSMIEFFTAMEHVLDGYD
PGPRWQNLKDVSELMWERCKTWKRNGVPPEHYAGGEIALLWAFNINSPNVVFWLLIRICSNPNIL
GRVRNEIAPFASPTHLNLNTKDSFYACNLNIQGIDKHCHFFTACEYECFRMDIGTSSIRTAQADFVIK
ESAADTFDGALPQSYLIRKGEVIHVNHDLLQKDARYFPDPGRFIPERFLAEEEIQGSNGNDEGTGEK
NKVLVAKRGIIRPFGAGPNMCVGQKFAEREILLFVAGLLIAWEFSPVGGEWILPGHKRTAGAYMPD
RDFRVKVKRRRE*
>CYP504A48|5678|Dibbae1
MALSYQTIGFATVAVLYFLIKYFNRTDVPKIKNLPEVPGVPIFGSLLQLGQDHARVAGEWVKKYGP
VFQVRMGNKRIIFANTFDSVKYLWINNQSALISRPTLHTFHKVVSTSQGFTIGTSPWDESCKRRRKA
AATALNRPAVQSYMPIIDLEVNESIKELLADSKNGEIDVDPNPYFSRYALNTSLTLNYGVRIDGTVD
NELLNEIFHVERIISNLRSTSNNWQDYIPLMRLWPSRNQQAQEYRARRDKYLKTLLDMLKDKMAK
GEDKPCITGNILKDPEAKLNEDEIKSICLTMVSAGLDTVPGNLIMGIGYLSSPHGQEIQARAYSEIMK
VYPEGDAWDKCLGEEKVPYITALVKEILRFWTVIPMCLPRTSIKDIQWEGATIPAGTSFYMNAWAA
DYDETHFKSPFKFDPERYLTSTEGTPHYAYGAGSRMCAGAHLANRELYTAFIRIISAFEIVEAQNPGD
RPILDALECSANKTSLTTEPKPFKVGFRVRDQGKLTEWLHAH*
>CYP5090F1|5962|Dibbae1
MLLFKPGEYLLTDNVLTSAQENVWLIVTGLFFTSWGLWRLWKFTILPRVYPDDPKELPYWIPGHGIS
FFNNSNHLLDYAKDYFRNAREPFAITAAGSKLYILTNSKDVGIAYKNTSTLSFERFVQAMMRTSGSS
EDVVSKMYQVPDPEKAKFPNPNNKPLAKLARELHIFQLFPGEPLNSLCSAFVEYFKQTLTLKTMSRY
RYTKSFSDQDVVIPLYTWVSDVFVSAGQEAYFGRVLGQVEPELTWAFLEFDDLTWQVLYQYPKFLA
NTMIRAKSKVIAGLERYFTLPPDKRTGASWFTPAMEIEMRNLGFDTHEVAVMMMTIYWGINTNTR
RACFWMLAYMIFHPELLQTIREETKPAFVGENFQTPDIKYLEEKCPRLNGVWDETVRLSAYSSSVRY
VTEDTIIGGKILRKGNRVMIPNRQLHFDENVFGDRVREFNSTRFIENDALIRSGSWRPFGGGATMCP
GRFIAKHSTIAFVAMMLQRFDVEPAVKQSFPRLEEGNPVLGIMSTKDGDDLSIRLKIRVQKA*

>CYP6415B1|5993|Dibbae1
MSTSYIANSVNSSGLPSWQWQLSSYVSQQQADLPMMGSRLLALVFASVLLLVYVSSPLNFWMGLYL
KKGGQTPLTLPYALPVFGHVLWYSWDTQSFALMAKRNFQEFPVKLRLIVMNPVLITKAEDVVHVF
AQSAAFTNKPYRNFVSSTFGLPRKFSEFYIADDSGWRHKAHPQSCVKPENRVDYLMHNFITRFFSGT
SLIAFTGRFTINMTNRLENTGFGDEWAEGMDLFKFLKTQIFHAFVEAVFGTHIFELNPDLCDDFWA
FDSKVPDLAKGIPKWLSPNMYGVRDRCLQSILKWHLHLQKHGQEETTTINKKYDPVFGSELMQQR
HQAFSNMSSDIVCAQAKASEDLALMWATNANAAHSAFWVIYQILRDPQVLARFLQCIKSAELAP
NPASAPGILKQYRVDALCSSPYLSSVFAETLRIHVANMLLRDPLWDDFYVREWRVPRRTVISIMSYN
MQHDEKAYNTGTPQDNHPINEFWAERFLVLAPEQSTGALSHAVQTPEENADSTNEAWVESAPAH
MQPSDEGAPKTVFSTVGLKGNWIPFGGGAHICPGRNVAKQEILLTAAMLLGNFEIELTGPPPVAD
WRFFGAGALGVIGKQECRIRRRRH*
>CYP6828B1|5997|Dibbae1
MRFGTKYHLRISKRFPLPIYTLSVGLRRVYVVNQVDLAAEIQRKSKIFSFDPLIVVAGQNLAGTSEAG
MDIITRSLRDRDSKRGFLHDMYHRLHSMLGPGPALDEMTLTMSGNLSLCLDAIKPEGEVLPLYGWI
KHMIAMVSTDAMYGPMNPFRLNPKIEKAFWDYESKLPVFLLNAWPRIIASRAYHARELFTNAFAD
YFENDGWATASGLIRASYQTNSKHGISNKDQGGFELTTCIGLIVNTAPTIFWLVLTIFSHQSLLEELR
KELTAIATPMNGSDLNGMELSLFRLKNDCPLLHSTLREVLRQRTHSQSPRVVMEDTELGGRYYLRK
GSIVQIPSSILHSDVTIWGPDASSLNTRRFLKTEPRSKKDGAAFRAFGGGVTLCPGRHFATIEIVIIMAL
LILQFDLSPESARSGWVLPGEDGSKITTSVLHPMEETRVFISRRKNCENVRWKVVLA*
>CYP6264A2|6712|Dibbae1

MEGLKQKIIASPPSMIGGIEEKLLSWPVLAAIPFLWIFLNGIYRLYFHPLAKFPGPKLAALTLWYETYY
DVWQRGKYVYKIEEMHKKYGKIVRINPHEVHIDDPDFYHKFYQSSRKLKKYPWYYKVSGVDEVSF
GTEDHEVHRQRIGVYKDLFSIRSIVLFDPVIKTNIQKLCDKLDQHVELRTPINLSHAYRVLTSDTITTYI
GLGPAPLLDDEDLGKSYRRYARIVTESSVLVRHFPPLAYFRFLPHRLVANLSSDFGILKDHLDRLRTQ
VELACTQYEKQPDTPHSTMIKGIIGNERHSNKTIQEITEEALILEGAGTDSTGQALEAATVYILSTPGV
AGRLREELEKAIPEPDQIPTFPKLREIPYLTAVINENLRVCSPASSRFPRFNEHAVTQYKEWEIPASIPIS
MNIWNTHYNPSIFPNPAEFCPERWLQPSSKDLEKYLVPFGSGSRMCIGMNLSLAEQYLVLATVFRRY
KLNLYETSKRDVQMTSSCLITLCPPESKGVEVTLEKL*
>CYP5130A2|6713|Dibbae1
MHEKYGDVVRVAPDEVSFIRSEAWSDIYGHKGAKSFIRDPKWYANLTEGQDDIIVSNEFDHSRFRK
AFSAPFSDKALKENEPVIKANIDLLIERLYGQMKTNDGVADMMKWYNWTTFDVIGDLVYGEPFGC
LQNSEFHPWLAIVLQNIRLSSYVALMERYPLFKKLIMNLIPRSLLEKRNMHIGIIREKCARRNESKPN
RKDAISQISESDASLSQRELEANLALITMAGSETSATALSAATYYLTRNKEAAQKLRQEVRSAFTKES
GISWDVVKDLPYLTAVIKEALRLYPPTPVGLPRRVISSGEMVCGYYIPKNTVVYITQYSAYRSPTNFH
LPNAFHPERWLPVHGPKIPEDPNPFAADNLPVVQPFIIGPYSCIGKSLAYMEIGLVLARMVWNFEW
ELVADGKGCGFEEEKVYALWQKSGLVVRLRGEGGGVRV*
>CYP51202A1|6905|Dibbae1
MTRVYKSGTKFDKDADWYSNPFFNGSHITIPDLKTAKQRKDMFQPYFSKAAILRVEPLLKESTRSFL
DALKRAASEERVVDLTFGFRCLTADIIMDYCYQKPFGALKAVSFEFPLIVALDKYSQAGQWDKYFL
HVFRVIAAILNSIPPNLAKLILPPMSSIQWMQKVKECGTQIHALKQRAKSSAHSSYPTIFDTMLHPSS
SKNQYTPSDADSTAEAVLMLAAGMDTSATTLVLGTWYVISDKRVYQKLRDELCRAMPKKDKIYDL
AVLENLPYLRGVVKESLRLSYGAPGRLPRVVPAVGARFCGWDVPPGTIVSQSSYVYHAHERVFKDA
AKFKPERWLGDDYAELDKHMVSFSRGSRGCLGIKKFSLTFSSLAYAELYLTFAHLFRNFELSPCRTTA
ADMGWGDYFITVTRGHLKVTLREAEQQERADLSKVT*
>CYP65FR1|6956|Dibbae1
MILQTEDLEAYVLSLGGGLLVLSLAGFLAWLSTVIYSIYFHPLSRFPGPRSAAASYLPYSFHLVEGTFT
TWVKELHDRYKCDVIRIGPNHLSFISPSAWKDILGGSGSRSFEKDVAVYGRSPNGVESLLQAPRADH
SRMRRVLDHAFSMKAVRQQDPIILQTVNTLIGGLKDQVSGDAVGKVDLVRWYSWMSFDLIGDLAF
GESFHCLEDKGNHPWVQMIFGNLKGFVFMNACSRFPAFASILPWLIPPSVKRMIDDHFKQTQDKLS
RRIAKGASRSDFLTPILENNSAKALTKDEIDSNASLFIIAGSDSVGTSVAGATWYLLENPKVMDRLRR
EIDETFSKEEDITTQSVDGLPYLCAVVQELFRIYPVALAGQASVVPHEGAVISGHWIPGKTGVSMNQ
FAVYRSKTNFQDPDVFDPERWLDEPEYGWDRKDAFHPFSIGPRNCIGRNLGLAATRLVLAKMVW
HFDMKLSGETDPNWLHQKSYIVWQKKPLLVELAPRASAKAKKEDNGIALKKEGGKAGLTDAEVM
PAPAVTNTPVIEGKREDGIPEATTETVLGSALENRRSEVKNGKAL*
>CYP51200B1|7063|Dibbae1
MSPNLLVCGTAAIAGVVSHLTYFIHGEHHNQSPLYFKLLLAGIALIFASEFLLGVGTIMGSVANTACI
VSAYLVPLYTSMLIYRIFFHPLRKFPGPFMYKVTKLWHVLKLIKRNNCHVLEDLHQQYGEFVRTGP
NELTIFNTDGMYALHNTASNFPKAPWYDILLPEIAMSTARDKAVHDQRRRIWDHGFSIKGRVTGY
AQILESQIGKRAGQGVNISEWFNWYACDVMGDLAFGKPFDLLATGQNQADVELLKKGTPVVGLF
TPTPWLFILLAKMPGPNREWKELQKWCYAQLERRLKSKVDVPDIMSWLIEASEAQNRLESDMHLL
RGDSALIILAGSDTAGAGLSSLFYHIISEPKYLVALREELRTIPSLTDVKALEGLPLLNGIVHETLRMHP
PTPSGVLRITPKGGATIGTTFVPGDVTVAVPFMSLGRSEKCFVQANEFIPERWYSKPELIKDKLGYVP
FSIGQYSCVGKNLALMEMRSVTALLVAKYDIKFAPSQLAKQTSFELGLHDHFTSTAAGLDLIFTPLE
KQPTNVK*
>CYP682BA1|7152|Dibbae1
MGFLETITSIWFGPSIVLLYTIYGIIYRLYLSPVAKFPGRKLAALTFWYEFYYDVVKRGEYVWEIQKMH
EEYGPIVRINPYELHVNDPSFMPTLYPNGAKNVEKWSWSAGMFGSTGMAFSTVNHDLHRLRSSAF
RNFFSKQSIREYEPTISQLISNLCQGLERHQRNGEVVNLVDAYSALTHDVITEYCFADCSNWLLQPD
FCKEGIDNVTKPAESTHLIKQFPLMLPTVDLLPNWLVGLAHPLALQLRRNRASYEVQVDNILRGID
KPLTGSHPTIFHSLRDDPNLPPAEKTKRRLAMEAQSLIGAGSVSTAHVLALTTYQILSNPSVLSRLLSE
LEAAIPDPNVIAPIVELENLPFLTAVIYEGLRLGPGISHRLQRVHPDKSLTYGKYTIPPGTPIGMTSVFT

HHNASIFPDPETFNPDRWLPLDDNKDIFKYIFSFGKGTRQCVGQNLAYAEFYCTLATIFRKYGRNIT
LYDTEWHRDVEVRKDFFVTSPSLESRGVRVLIGGAKRGE*
>CYP544C4|7403|Dibbae1
MIGIVVSLLLLVALWFLQGQRSEIRRDGKRLPRPPGTLPLAGNGIWFLQPRHKLLDWFAEVQPKVG
FGTFEISVPSLPPAIVVNDPRSVEHVLKNNELFIKGDFFRSRSWDLFGNGIINADGALWKIQRKAGLR
FFSNANLKSFIDEVLPPIIEDTKKFLDKAAKEESLIDLQDVLLELTTRLMGKVAYDVSQRTGFNLMDI
DGSMPFSRSFDFASGQITARFMNPFWKAKEVFFGAKLKKAVAEVKTFGRKIVAAAVAKRGEKPIAG
QGVFKSEDDPLKNNLINALLDHIEDHQMVADAAMNFLSAGRDTTAQSLTWTFYSLMRHPSVLPQI
RQELVTIFSPEISSLPLSYDAVQSTSLPYTMSVFNETIRLYPAVPFEIKENTSPTTFPDGTYIPAGSVVVW
VPWAMARSTQIWGPDAPSYRPERWILPDGTLLNKTAYEFPVFNAGPRTCLGKKMAECLAVYVIAA
LVWEYDFVEVRDPKLGGCGWGAGRERKSQDSLTLPMEGGLPCWVKRTR*
>CYP6798A2|7580|Dibbae1
MNSSLLLDDLGFYHIEPLQDTQLIPPVKSSFERWLPVLFLFGMIAAWEVKRMILVFRNRCPENVTIA
GVDSIIGKWLTSIRYCWVSQSVHNKAYAEGSGKPYAIPTRSRYQVCVSSQKLVEELSNASIHQISLRD
ALWELSQRAFPEQTIDGLKVDGLDQNGSLSQKVFRHHARLHLPSLQPLYEKRLEEAFVREVDNQK
KKDGWIQVSAGRAIHRITAKINNILLVGDELASDPVFFENVFLFPLTGWILMKATGVTQNVRKYIVA
LAKQRLNNKSAKGEEPKDALQWIIDSQKKGTAEDVAQQTLAYIFGSAYQMPMLISFAMYNLCKHP
EYLQPLREEIERSGGVILNHQNDEMPLMDSFLKETARMNPVTIFGMPRKVMKPFTFSDGTHVPANN
WICVPQQRMMKDPANYPDPDTFNGFRFVKEKGEDAVTVSESRLSHPSWRFPFWGSVKQACPARFY
VTDMTKMIISQFIMNYDFKLADENVPDSFAWGVLRVPHPFLAFLLKKRETY*
>CYP6466A2|7584|Dibbae1
MPGGKVYVINSPDLAIAVERIPTKISFWHVEAVFTGTLAGLSPFAVKTLKENVNSDNGEPSYLREGM
VSVHTTMKPGEGLTAATRAAADLLVPSIGKLAQRSATQIELGQWVTDELMASVTGSIFGPKNPYQD
PDVAKGFWDFQDNAGGLMSLPYPSVTCAKSYAAREKVATAFKKYYDARGYDSASPWVQYSTKVS
DKYGISDTDKARIDVSNSHAILANTTPTAFWTIYHIFSDASVVDEVRAAVIPFLTTKLKDGSVTYDIDI
SQIRDVPILKSVLSEAMRHYASGTGTRIVVEDTMLDNRYLLKKDSFVFLPNRSYHFNSSAWGSTVEG
FDARRFMKSKTPRGSFRAFGGGANLCPGRFFAMSEILAMAAMLALRYDIKPVAGTWVHPGVDDS
NMTLIVHPPKEKVLVKVVPREGWNCGEWSFTVK*
>CYP6615B1|7746|Dibbae1
MSVVFNGVLLAGSLIFIRICYLLLLHPLARYPGPFWAKLTYFWSAVTRVLRVRSFLKGEQHNVDLQL
HQRYGKFVRDGPDSLLISDIEAFKAVYGFTGAVEKGDFYRIMSNGKPHDPNVFAARTEVLHREAKR
KLVSTAHIAQYDESITKNATAFRQKIHEAVKNRGAGRSINIAPYIERYVFDAVLEVVFGSNMGFIEA
NHDKYGLLAQKKVKRWPFTITISSVPWLARFMSQPRIGAYFRKPRLDKDGQPIGLTAAGFFARSAVS
AATSGTPTPSPSILKCLSEVPPSDNRRLSKEMIFSETFNAIFAGTGSTTGALTAVVYELARHPELQSELY
KSLVSSRGSDPSDSSSFPSYSALKEISVLQALIRESLRLHPPQSGPFERVIPPSSTSPTTIPNIPTPLPPGTRL
WSSQYVILRSREIFGDDADEFRVERWLPGEGGPQKREAVKKMEDIWNVFGRGSRTCLGRDVTWSVI
EKGIAALLLNYEMSGKEEGLKVKNAFEMQYEELSVVFMPRV*
>CYP5527B1|8142|Dibbae1
MIIDIYGLKPNKRQLAKDRATYDDNEGNIIRLLTPLVFSLYTADFEANDADHARMRKLLSHAFSDA
ALREQESLMSSYFDLLVDRLKTEIDGPSQGIVDLVQWYAFTAFDIIGDLTFGESFGALERGEYHPWIG
NIYKGLKFLRFTIIANHYPLIRQLLSLLSKLPALANVRNAHYGFSTARAMQRLNRKTDRKDIITYVLR
HNGERGMTRDEIVSNSSILIMAGSDTSAKCLSGATFHLLKNPEILRRAQEEVRSAFSNGRDINLVSVG
QLRYLPAVLNESLRVYPPAPGTFYRRTGPQGDVIDGKYVAANMAVGVHQWSANRSRKNFADPESF
IPERWLESPPEKYQNDKREAMQAFSYGPRNCLGQNLAYAEMRSILARILFHFDLELCRESENWDDQ
DVFFLWNKPPLIVKLTLRS*
>CYP548AJ6|8150|Dibbae1
MLFLPNLVFTLWRGFVLLIFVCVYRLYFHPLAKYPGPWLAKITDLYGVYHTARGQYHIVRDNAHK
QWGALIRLGPGKLVINRATALHVTYEKDIYQSKDVQKSSGYKLLAPEKDGYSTLTVIDKSIHKHKRK
ILDYAFSQQSMRSFEPALLSLVDIFVACLTKSCTSHGGDNAWSSPINMTDATNYLAYDTMGKFGFG
RSFEMQLEEHNRFLIDCVKAVTFRAGIYTIYPKLASLKLEKLLSKRVASMYKKYLELMSDLVRSRLTI
GFKTDEHDLFSFLITAKDGETGQICTETELWRESRMLLVAGTETSSTALTALFFYLSRYPISYTKLATEI

RQTFTHGSEIQSGPKLSSCRYLRACIDEALRMSPPLSLVPYREVSSESGTAGGGIHIEGEYIPPGIEFGVS
IYSLHHNAEYFPAPHTFSPERWIESSSNPSAAIERARYAFSPFSIGSRACAGRNLAYMEIGLAIAKTM
WYADFRPAAGPLGVVGEGDPDQPDGWNRVKEFQIREHLTSQHDGPWLEFRRREEVWGEDGGPS
QKGSKDAVRSI*
>CYP65FQ1|8543|Dibbae1
MEFRPPDQKSLLLAIVVLPILGLSYLLSKVIYNLYFHPLRHYPGPKLLAATRLPYVRWMYKGTLAHN
FRALHEIYGSVVRTAPNELSYTDPAAWKTIYGFHQPAEGFLKNPMFNPPATNGYHSILTAEGKDHA
RMKRVLTHAFSDKALREQQDILQHFTDLLIRRIHECLDGNPGPVNLFEWFNWTTFDLIGDLSFGEPF
NCLQEAKFSEWVALVYYAFKTITFINISKCMAPLDKIVKLLIPRSLVTKRNKIFNMNSSKVDRRLVSN
TPRHDFMSYILKHNDKYGMSDGEIYANATLMVLAGSESTATVISGMCFFLMKNPEVMRKAVEEIR
GTFKSESEIDFETVRQLKYLAAMVSEALRMYPPFPEGLPRMAPRDGAEICGRFVPGGTLVQISQWA
MHRGSYNFVDSDSFVPERWLGDPRYDGDYKEASQPFSVGPRSCIGRNLAYLEMRLIAVRMLWNFD
MELCPESYEWDKQESWVQWDKKPLMVKLTPVARYEELNTTPQTPSTVVA*
>CYP5238F1|8618|Dibbae1
MEGQLVQYLLGFVAISGLVFIIYKYLLLLTRLPNAPGATFWESQPWVGRKKQMFSQLRASFRSVKHS
QSMVDEGYAKFSKSNRHFVLPSMGSTSVIIPPLQLHTILNKPENEIEAMDSQNETLQASYTIQDKEIY
VNSIHFNVVNKQLTRDIPSFIESIAEELALGFQRYWGTSTEWKTVGAWDTCQKIVARTANRVFLGTT
LCRNEVFLDHSRKYALSVFGGGIVINTTPSILKPILGFLIGLEANRHLAVCKKILIPVVEERLKNTIQLS
NDPDFKWTPPYDALQWIIEECEKTDDPKQFNPALIAHRLMMLNLVSVHTTSNTLINVILDLYSSPPS
SGFIDGLYEECTRVLADSDGVWTKHAVSQLFRVDSTIRESMRFSAFGIVALPRRVNSLNGLELDDKSI
LPKGVRLTFPMQAIHQDEDFYPNALNFDAFRFSRPYETKRADLNRKFESGDTITDTFLAFGNGRHA
CPGRFFAMHEMKLMLAHVVLNYEVEFMPTRPPMEHLMELKYPAIATQIRVRRKA*
>CYP6677B1|8715|Dibbae1
MVVADADAVNTMLANKKDFIKGGAMYDPLNVFGPNAVSANGREWQRHRKITAPAFNERVSLSV
WTEASRQAEDMVASWIQKGATGTTATVHDTALLALHVLAYAGFGIQYSFFEGTHSPREGHNMSY
RDALSLVLGNMILIALFSKKLLQSLWLPGKLREIGAATAEFQEYMEEMLINERRVISRGERASGNLVS
NLIRASEETSEGLTDEEIYGNIFVYNLAGHESTANTIAFSIVLLAAYPKWQDWIGEEINAVMREEPGY
QTAFPKLKRCLAVMLETLRLYAPTFFIPRYTPVQAPIQTLPINGTLRAVLGNTSVTINIQALHTDPKT
WGSDVLLWRPDRWIVSDPLNPCLDTETFYEPPKGTFVPWSEGPRDCPGRKFAQVEFVAVMATLFSK
RRVRPAARTGGDAELKEERNAKWRTGRKTGVGGSYLVGRKVP*
>CYP677J3|9120|Dibbae1
MFFGLSLIQITVLAVFIYLVSWIIYCRCFHPLRSIPGPFLASISRAWIVFKTAHGDMERTQRTLHKKHG
YLVRIAPNEVACSDPEAIKTIYGTKIIFTKVLIRRSSSKQSSLQQTDYYDAWAPPHTGYVGHFPSRDEK
EHSERRRIVNNIYSMSSVLECEKAIDSCTQVFCETMRDFAKQKSLMNESLSPKMKTGQRAYLSSRYA
FDVLGELFYGKMFGFMSERTDVGNYIKAIDSLLPAFSIGGTVPSYLTKLFLASTILFSPSVRGALGAIK
HLESASKSAVQRRKKEVEENKDNKRDMIRKMLEINADRGEKINFTYQDIHVESHSSLFAGADTTAI
AINSILYHLMHNPAAYERLTAEVDAAVTDGTLSMPAAYAEAIKLPYLKACINEGMRLHPSVGLTM
PRIIPAGGASISGFHFPEGYRVGVNGAVVHYDKDVFGSDADNFNPNRWIEGNAIRMDKTMIQFGA
GPRTCIGKNISLSEIYKLVPQIIRDFHIHLVDPSKEWKTQNYWFNKQTGIYIYIEERTHRPNK*
>CYP680F1|9312|Dibbae1
MAGFSASLLLYLPAAILIYLTTIIVYRRFLHPLSKIPGPSWAAITYLYAFRYNVIGPGSRFYLQIEKLHK
QYGPVVRIGPNEIHLSDPENYEKIYYVGSKFWKDPIFYGGLGLSTSAFGTPSNELHRMRRAALQPLFT
RKQVLELEDVVQSKVGLLVQRMDQDLDESRPVDLHHGFRAISVDVVTDYAFDNCYDLLKSPDLGR
WFCSMIRDLAPRIWILQQFPFVLPLSKSIPPALARKMSTNLATFLVVKDNCENEIRTIKKRVEASEKM
PAKKTIFHQLLSPDATEGHVVPSVDDLIDEAFTFIGAATDTTGGALAVAAYHSVSNPDIYQSLVAEL
KEAFPDPKAKLDFQTLERLPYLASKSLPLKSFPCVDRETDCGNQRRPSTRLSFGVPGRLPRVTPEGGA
TFNGYFVAAGTVVGMSSWMMHRDPSIFPSPETFNPDRWTNPATTGQLEKYLVAFGKGGRQCLGM
ALAYSELYITLGTIFRRFENLKACEMSTQDWEFDDFFGLYIPESATKFHVVNS*
>CYP541B12|9390|Dibbae1
MPKPIPKPPGVPLLGNIFDIDPSNTWVSLKKLADKYGPIFKINALGTQIVFVGSASILEEICDQTRFRK
CVTGPVVEIRQAVHDGLFTAYDSEPMWGIAHRIMAPLLTPAAVQTTFVEMRDTVAELVAKWTNN

KADGGAKQRVNVLNDLQRTDLQTNMLCMFGQKVSYLEGPEPAVIKGMHDSTMEAMKRPTRPKL
VNWLLYQGVFDRNIKIMRDFAAEVLAYRKAHPTPARNDLLNALINSKDPETGKSFTEQQIIDEIVTP
LIGSSTSPCLMSFAIYYLIQNPKEVTKAREEIDAVIGPAGSQLELSHLSELKYCEAILRESLRLSAAAPG
FNIEPLPSAKGTVQLAGGEYEIPPNQVMIAILSAVNRDPTVFDDPLVFKPERMLGEAYDKLPSSVKK
GFGNGKRECIGKNYAWQWSFVVLVMILRSVDLEMADPGYQLREQGAFNMETQGFFALAGPRK*
>CYP682BC1|9712|Dibbae1
MPFLHFPAFQKLIHNLRYEFYYDVFPYSGQYTFKLREFHEKYGPIVRINPYELHINDATYYNTIYAAG
GSGEKRDKWQWYSKIFGAPGSMFETPGHDLHRARREPLGRYFSLASVRRLQPVIQERVNKLIGRFRE
SINSGEVFKIHAAFAAFTNDVVMEYSFGCSDRRVDAPDFSPWFMEAVVAAQKSTPLMKHMIWIFH
VIQMLPDSFGKRFMPKLMQLVDLQRSIRAQVVKAKAQEKPTHYNSGHPNIFHELLQSDLPEEEKTV
DRITDEAFILTGAGTGTTAWCLGVATFHLLSNPQILTRLKKDLKAAIPDTNVDPPLPVLEQIPYLVAI
VQEALRLSYGVSSRIERISPDKALTYTEPSGKTWSIPAGTPLAMTSVLIHHNEAIFPNNRSFVPERWIE
DPRLDRYLVSFSKGSRACLGINLALSEIYICLAALFRRFGSEEVRDDGDEGYLELFETTLEDVEISEDLF
LPFPSRSSQGIRIKIRR*
>CYP5089D1|9723|Dibbae1
MGGNSHIVEQDLHKKYGHVVRIAPNTLTFSDLPSFEAIYSFNKFLEKGDFWNFGREGPNEAGNIFT
ARTNACHKDRKRKVFGLALTNAKVATYKPTISKNVSNLILRLNELRAASEDKSTIDIAETIHRYTVD
NQVDIVYGQSVCPVAFTDWPPARGVIGSFESSSRLIWGILLLPILGKIMRLSAVTSALRRPTHDASGN
ETGIGSLVSAAQKVVRSEPEKITEVGEPCILRYFLDVKESDSKYQEPDQVLRESVNLMFAGQGSQAA
ALTATLYYLAIPIGHQWQRWILADQNSEADMPLAASSILMAVMKETVRLHAPIPTGFPRTIMPGAE
TAIPGLPAPLPTGTDVQTNPYVLSRSKEIWGEDAEEWKPQRWLVETDAERKELDEKFVTFGKGPRA
CIGKEMAWIVIAKAVTAILQQWKISAEERTMKGANYLEMHYHTCKISFVER*
>CYP6456B1|9728|Dibbae1
MLSGKVYVVTSPDLVAAVNRNSKALAFNPFIAAVGKRITGHDEVTSQIVQHNLNGENGPGYVIEIH
DGTVAALVPGKTLEDMMEPMFWGVSSHLDELERSSEVGLFTWMRKMVTMCSTRAIYGPESPFEKN
PKYSDLFWDFDRDLNLLIVDIFPSIVAPKGNRARSILAEAFEKYFADYVPGVTQSSAMIHSRHAVNT
KWGLTMLEQGRLEVGVLLGILANTIPSSFYLLVHVYADQELLQQIRNELETHCVLKSPSETKRSISLM
NVREKCHLLHAASQEMLRIHAQGANSRYVREDTMLDNRYLLKKGMVVQLPMAVMHSDASIWGS
DVNDFQPRRFLRQNDASTKKSGASSVEEGNKSKQSTAYRPFGGGSSMCPGRHFVTLEVMVLTAYV
VLQFDMVPTSGPWHIPAQKQESMATNVFPPEKDIRVRATRRKGYEDVKWDFVIR*
>CYP59V1|9977|Dibbae1
MALHIFNVSYGRLGYTVAVVSICIVARFLVNLYKVRGRIWRLKKQGLPMPPYHPIFGHLLEVGDIYS
SIPPDAHPLYLPDQLRRKYPNMGPVFYVDMWPFAQPFLFTDSTATASQLMQEHPQMKADDIRRFM
YPLTKLNDLVSSEGQLWKDWRNALSPAFSANNLISLLPQILEAVSTYVDVLGENAQSQDTFSLENA
AINMTMEVIGTVTMGFRFDSQKSNRDFASALREQILWLTVKLNPFDAYNPIRPLFIAYYNSIMDSFLS
LQIDKRFAALQRLNDVDGAEDKKNTVLGLAFDKYKKTRKLRNPTNVLTSEFKHFVMGQLKGLILA
GHGTTANTMCFIYYLLSKHPKALQRVREEHDNVLGHDVSQTASIILEKPHILNQLPYTLAIIKETLRL
YPADSSPRSGSKDFLLRENGRLFPTEGCSVWTITHAIHRDPLYWPDRDAFIPERWLVAADDPLHPVK
GAWRPFEFGPRSCIGQELAMLELRLSLVMAIRKFDFEPRFEEWEQKNGVKGPRMVNGEIGYQTLDG
TNRPRNGFPCKVSWRY*
>CYP682BF1|9997|Dibbae1
MAFLEAFSVGRVTLLLIISWLSWSICLAIYRVYFSPLARFPGPKLSAATYWYEFYFDCIQHGRFSWEIE
RMHDVYGPIVRINPDEIHIRDADWHDEIYTGSTRPRNKCYFFVGRIGRGSIFGSITDEQHRLRRQAIN
PFFSKRSIVNIEPQIQEKVDKLCRRLVEHAGNGEPLDLGACYTALTLDVITHYAYGQPLGLLEMPDF
GSNWREMIQMTLECMPFVRHFPWLMDVLEKIPYKLARRFNAAVALMLHFQHQIRTQIVTTLQRGT
HSTPEHKSHKTIFEELRDSDLPASEKSIDRLKEEGFVLTAAAADATSMTLTVLSYHLLSNPSILARVRA
ELHSAQPDPTIPIPWQTLEHLPLFRACITEGLRISALSTSRLIRSAPHEALRYKDWVIPQNTFVSMTSH
FIHMSPEYYTAPFTFDPDRWLNADRQATRPKLDRYFVPFGRGSRSCIGQNLALAEVYLGVASVVRR
VSLRLFETTREDVRVVRDAFVGYPVESSKGVRVLVVGGVVGHGDGDVEMEKQVLNH*
>CYP6178B1|10011|Dibbae1

MHHSQALFSRGHSHFPGREPYALTIAGQKIVVIQTTEEVSTLWKQTVIFTFEPFVQNIMKAFGFDHTS
IEAMFHEDPGQLLPISVRHSALLSISNPKRKCYFKMQSEWLKHQLHPGENGNLKWLQDKYAHFLS
KSISWDELSSEFVISANAKEKLISLKRFTRQTLGDCAMKSFFGTKLFENSPSFLSSYQQFEDDSWKVFF
NYPRFMAKDLHRIKDKALDDLVTYFALPTEQRPGLAWIFQTLDSELKGLGINPRDRAGIVMMITW
AINHNAHKISFWIFAHILYDPALLTDIRLETQKAFNKDGSLDLELLLTKCTHLDAVWYEVLRIYNNA
AVARKATEDTIVSGKVIHAGETVFGPFRQFHLDAAIFGPGVSRFDPNRFLASKSLNHTKGYHPFGG
GNTYCPGRFFARSEIYILVATALDRLDLEVALGQTLPEVDLKVPSSSAMPAVKDVIVKIRPRGRH*
>CYP62W1|10033|Dibbae1
MLTVALTTGRTVRIAPCEVSVSDLHSLKEINKIGGGFVKSEWYSSFASEYEGVNGMFSTTDAKKHSE
RRRHFARAFAPQSIQEWEPFVQSKVQLTVSSIENLGQRGEVDILSWWSFMTSDVVTKLSFGESTGSLE
SGQKSAFQRDLDITLIWRGLQSEMPWLTFILYKIPIPSLQYLIRSQDRLETYASEIIQQSKRLNNLKGTF
FGKVFEEYGTEKSLTDLEITLEAKAFIIAGTETTAITLTYFIWVMSKYPIIRQRLQKELDSLSPGFTNQD
VQNLPYPQMVLMEIFRLYTTVPSSLPRIVPKAGRQLGGYFIPEQTTISAQAFTIHRDPTIFLDPLTFNPE
RWTFPTQDMKDAMQHFGGGSRACIGKHLALMELNLGIAHFFRECPTVEMAPSTTDKSMDFVDSFL
IKPKSGKCAVVIP*

>CYP5070D5|10089|Dibbae1
MVFEPALIPMVLAAGLGSVVAAKTTSLDALTVFLYSLSGVLAFYLTYYSYIYPFYVSPLRHVPTVPGF
PLWGQFFTIITEEIGVSQRRWHKQHGPIVRYFFPFGSERLSIADDDALKHMTIRNPYNYPKPDRARQ
WMVRVLGEGVLLAEGDTHIHQRKALTPGFSISVIRTLMPVFWQKALLLTNLWQREMMIENVNTKS
FEVLEWLNRTTLDIIGQAGFGADLNSLEHPETPIREAYRLVFAFDIQSRILHGLAAFIPLTKYLPAKM
NRDILKSRNIIISKATEIVKSKHTKANSKDKDIIALIVRDNMKMEAGDGDKLSFETMRDQVMTFLGA
GHDTTATGVAWTLHLLSKHQDVQEKLRAEIKQYMPFLWDPKQRNDREVYGKVDPDHLPYLDNV
CRESLRYIPPIPMTVRQSVADDKLGGYFIPKGTTIYIHANAIHRLPEFWGPTANEFDPDRWDHLPKD
WSPNAYMTFLQGPRGCIGKKFAETEMKLLVVSLLSLFRFERDESVTDPELLKMWRLVLRPRDGILCK
VTALEENVVG*
>CYP51180A1|10243|Dibbae1
MKLNSYIAILVENVVDHSSNGDKGNRERYTDIPVRIKLFFLDIYIITGPEHITSIFRQSQTVFPLGSLVRR
RTLHNIFGTPSNAGKIYDEDNSGSLSKPLPGTNVKPHNRVNYIHHHSLTKWLTGPGLKELAERFSE
NLIEQLRTTKELTTEWTELPDLLKFMQHELFYATSRAFLGDELFTLNPRLAEDVWEFDKGVPYLAK
GIPRWAKPSVYRARDKAVQRFVSWHKWVRNYVEREGMPEDPQTIRKVFGNDFMKTRFEAFWKM
DYMSSVVEGPASEDFMVTWGANANSVPAAFWFIFHILHSPSLLSTVREQIKPCVSISSDSKLDIDITAL
CANPLLQSAYMEIMRLYISLTLIRDSEESDLCLNEWIIPKKSLILIPGQSVHRSQKLWEGIGKAGTEESS
PYPSVDTFCAERFLKYPEKGGEPFFSAENLTGYWIPYSGGSNICLGRYYAKQEIIGAMAIMLTLFDIEI
LGDENIGEPDAERIFGFGVMKPKRSVPVRMRRRQV*
>CYP609P1|10265|Dibbae1
MTVFEIPDGIGGAFKIEWTQIMAVSVLFCVAGLSYLAGRQPQISPKAPPLINEGLPIFGAVRFWTERW
DFFRWAQMQNPSGYFSFFIGNHPVVGLSGDEGRKVFFESKQFGLTEGYSVLFGMFPSTKVENKEGQ
DTFHTTEFALHFKDRITRMVRKDRVEKTDTKARLDELVDNPTAIVDPFEAIYKLIYLLTMRTVGCNE
LCRNRHKLDLSLGLYETVARCATIWPIVLPWFPSPNLIKRTIAGARLYFMLNKIVSRRMKSGICEDDP
LQDMIDRGDEKSHIIGTIMGALFAAQQNSGINAAYVLVYLSCNPDWAAKARNEALTVARKYCPDE
SLSMADRLAMVPVEAWESELPTIELCLRDSIRMHSLGACFRKNISGKDIDLGTGIIPNEYFASYHISNV
HLNPEIYRDPEQWDPSRYLPDRAEDKKRPLAWLGWGAGRHPCLGMKFAKLEQNIVAAFFLTMFD
FHLSDSKGNPVDSPPKLDKNAYSSWKPETPVFIKYTPRI*
>CYP51166B1|10267|Dibbae1
MHYEAVPLHSAQAIMNRLHSGVQFSNFLEQMAASPPRLTFYNAFLATLIFLTLKFAFTIIYRLYLSPL
AEFPGPKIAAVSRLYEFWYQGIKRTEFPDVIKEMHRVYGPIVRISPFELSINDSEFNAGLFLHDRKLDK
DPWYYGLGFTESLFTLLNKDKHRQRQSHLAPSFSGAQFKNAEPIIEREVAKLLFVFEQNVKDSEGLN
LSVAFRKMGNEILRSFLMGERGDSTAPVDYMKDADVAYHPVFKTMTYVRHFPILYKIHALIPNWFY
DRWLPLAKYQRDADDHVRYLVQEHDKNVSKSEDDGLLYNFVELDPSYRKNNLQAAVEEFTALQ
WGGREVLGHGLTNIAFHLVSNPECMARIYSELCNSNINISTASYAQLQTIPYLNAVCKEGLRTQLGG

GFRIPRTCSTPITYKSYTIPPNTPISMCPKFFHDDEAIFPDHLAFKPERWLQGSEGEREALDKYWKPFG
NGSRSCIGMNLALEVVYRAVANIFLRYELSFHEGIDADFCRKDGMLKVFPHKNSRGLVVDVQER*
>CYP682AT1|10284|Dibbae1
MSYGWLAFYAGLWLGWQLFVIVHRVFWSPLAKFPGPKLAAATVWYEFYYDVWSRGRFWLQIQDL
HRKYVRISPYELHIDDPEFYDTVYNNTGKWDKYLPFAKQFHCPGSGFASIAHDKHRIRRRELNRFFS
RQAIDQQQPGLQKAVEKLCKRMQEWKGTGRPFPLKAAYSCFALDVVTSYFMPVSESFLDKPDFSPE
TFQMTVAANEFANLARHFTWAFSMLEVLPRRLCELISPAIAYQFQDFQITKTPEIFKAHTKAEAIVS
AISSIRDHETGRTSHNKTSTLEESTEFKTQRTIIEQLLKSTNLPPEELSAQRLASEAQIVLGAGTDTTSYT
LSVISFHLLNHPEMRARLKAELSVAMANSDRFLTWQELEKLPYLVMSPYCNTIVSKYVTDKLQTAVI
NEGFR*

>CYP5168K1|10516|Dibbae1
MPGLCHPILCLSDDLGLLTLHATCPCDQTIGTLRQLHPARMALQHLLHSNWVDDGPSAVGQDQA
GTKYPIHLPKRGHVAPPLRSTYTKFEILATQTMQDEATVFLEGVSTMQCSQQLAMVVVVGVASHLL
WFIHGEHHMRTPAIFKVYATVTGVVFAVYAVYGPLLEMFGAPKRHMIAHLIYFFSLYLAYFLSLFTSI
IVYRIFFHRLRRFPGPPLAKVSKLWHVFKVLHKDNYVLLEEFRKKYGDYVRTGPNELTVFDPDILQA
IHGYGTKCTKTDFYDIFLPSDSVAITRNKDAHRWRRHVWQAAFTNTALNNYEGRVQVHVQNLDR
KIEATRGKPFNITQYLEWFGFDVMGDLMFGKSFDLLTSGKQHYAVGMLQKGLDILGPLRPVPWLFI
LLGSIPSLKADYNRLIAWTSASVRDRIDRKVAVPDLMSWLLSSFGKSEDPDQEVTYKLQYKDEEMH
WLEGDALTAINAGSGTTSTTLVHIFSHLALHPDIVARLRKELMSIPPDQRSSNRGLTTLPLLNAVITES
LRLDPPVPSGMYRLTPSTGLTLPNGTYLPGKTTIVAPPYNLQHLESCFPRPDEFIPERWISGQGGRPD
LCLNKSAYAPFNLGSHTCLGKSLAWMELRAVTARLVMRYDLSLAPAMGGKGVGKMRDCFTAVP
QGLEVIFTRREGEKEEN*
>CYP6187B1|11009|Dibbae1
MLHSVFDVRGVLFWTGLLIVICSYFVTLAFYRLYLSPIAKFPGPKLAAVTYLYEIYYDVVKRGKYTFKI
KDLHARYGPIIRISPTELHINDPDYYDTLYNREGKWDKNPFYTNSMGNGAAGFTTVDHNLHRLRR
AAINSFFSKQKVTQLQPVIQRLTDKLCRKLEKIKEAEEVVPIECAFDAFTMDVITEYSLDTSFGYLDNP
GWSSDFRELERAFGEMGYLQKLFPPLLDIMNSLPNWLLLAMNPRVGLLQDFFRNCYAIAEKMLKE
TDAKKYEEKEHPTIFYEIIHSTDLPAAEKTPKRLESEAASILGAGAVTTAWTLTVAMYHLTVNPQKL
ERLRSEIRSIMPDPQNEPAKLQQLEQLPYLTCVIMESLRLSNGVSHRLARIAPDRSIYFRDWEIPRGTP
VGMTSTLIHQTPDIFPQPFEFVPERWLDPEDRKRLERYLVPFSRGTRQCAGINLAYAELYIMLSSLCT
RLNLELYETTREDVDIYSDMLIAEPKRYVNGVRFKVKEIIVNPY*
>CYP511A4|11068|Dibbae1
MAIVSFLQLEWNITRLVLVALGASLLYACTIVLYRLFLHPLRKIPGPWLAAATGWYEFYQDIILNGH
YVKEYPKLHDRYGPIVRVSPGRVHVNDPEFFHEVYHSGTKYFKDPDFFQTAGGIKYSLPMLIDPDYH
KQRRNMVKSLFSTKAIDQLSPIVLGVVKNALGRAIASNENGSPLDIQRLYTGITVDTIMRVLFDKQL
NLVESDEEEPPFLATLRTFSENFFLTKHFPILTTLAVNIPDSWAHKLVPGYAQFRKQVAVWIQEIEEK
HKKGIYSTADGRKTILDLLLKPDLSEGYQPLSKDILVDEAYSFCFAGTHTTSFSLSLGTYYLLRHPQKL
RKLVEELKIVKRNPEGLLEYRDVCNLPYLTAVIRETLRLSSPVPGITPRKVPAGGVICCGHYLPEGTTV
SISIRTVSDNPDIFPGPEKFIPERWLNSEAKEHWLVIFSKGPRACLGLNVAYLEIYLCLANFFSRLDMS
LYQTDENTTRWVDYGNAMLQKNVKVTVDKVKY*
>CYP5180F1|11116|Dibbae1
MLTKRKIFGFKHTLKNNVIELDPSDQTNATLRYRLFARSIRINGVAQISRLQKHLQLRLDRTCREYLD
AQISGEEWTTVKLAPIMRDAATGMMGTYFFGKTMCMQCQDFTLYGQTNSLLAEEPDFAYAMSRF
YVDVVKCMGALQVTPSFMQGSVYSYITKKGHALNILFNRLVLAISSKDGWEDDEELKQGAKITFLS
NMIEASKDSQYWTHELLVQAIIGIWFAASHQPWINLHFVFLELCTRPEYIDLIRDELSSQENLDYETI
DRLPILDSLMKEAVRLNPLDKTVAVRRKAVKPYTFKSTGQHVPVGQIACIPAWDLMHDESKYPDP
NVFDGMRFVKSSPDAPPSEVQGTRFTDASKDFPIWGLGSKVCPGRWHSALVMKLVLIHLVKNYDF
RLKDANKRYKWFWETFQMPVEDTEVEIRKRTRT*
>CYP51181B1|11146|Dibbae1

MVWYLLDNFFGGTEYTRNVDEAYIFKDIHAVLNILMKEAYLDKAMPIVSRGIRKNVAYFAPLDVD
PAKQTSWGRSSEPQIPRDAPSTVEVTLFPLIRNFTAYIASPALMGTAFMKNHPSCISDLWTMDAALP
FFITKIPSWLPIPTLKRAHAARARLHAAMIDFFTALERSLDGKEPEMRWKDLSDVSEPMWNRCKAW
RKTGLTAEQYSPPEISILWAMNVNANNVVFWLILRICSAPEILKRVREEIDPYITLKVEESTGTLSCDL
DASGITKSCHYLKAAMYESLRMDTATSTIKSVLNDFTIKESEADKFSETPQSYVFKKGEYLHVDHAL
HQTNPRYFPEPHVFNPERFLVEEVDEAEKVTMIANMGSIRPFGGIIFLDPHMGTY*

>CYP6187D1|11170|Dibbae1
MTDHYRHKMWRSVLNPFFSKQKIATLEPLIHTQLEKLSNRIDSFVESGKVLTIGIAYAAFTMDVITDY
AMEKSYGNLDFEDFNQGMANATKKFGKIWHLGKHASWFVPLFSKIPASIIKRLDPDSAQWKAFQE
SCLEESRKIVTADESSEVNRKRHKTILSEILKSEALPSSEKTVYHLRDQVQVLVSAGTDTVAHTLRVLT
FHICNNPDILERIREEVRQLELEDFTSLRLRDLEQLPYLTSVIYEGLRLSEGATLRLTRIAPDRDLRYRD
WVIPAGSPVGMTMHSILQNKEIFPNPSKFDPQRWMDPTDRRRLEKYAVMFSRGTRNCLGLNLAW
AELYLSVATIFSRYDFKLFETTAEDVEVGSDQFVPGTKSGNNVRVKATKADASARKIKGTSFYAF*
>CYP6174B1|11179|Dibbae1
MELFTIVLVLLVVWLVRVLSSPTLHKGIEKVGRSWKSALLLDNPVSDLVEKGYRKVIKIYQKPFLVRY
WAKDYVILPLKYLPDVKLADKRHLSFTENIPDGLNPQLPEASDLCLSEAAYAFEADIGDCAEKKQF
NAIEVLTSVTIRVVACVIVGEELARDEEFLKAAGTYFQGNFLTGVILLKLPLAIFRNVLGGSIAYFQRQ
KLRKLISMVSPIIEKRIQQKAQGVEKSERIDGIEWTLKLSEEYPFASNESGPVWGISKELIQNLWAAGA
SPAAIITQMLFHILADPSYLEPMRKEVEDAVAKHGWSERIINDLPLQDSFIREVNRFYPIFTLNCTRTI
MHKPFEFNDGFVLPAGTRIAFPAESSQLDPDHCDNPDQFDGFRFAKLTMAESKAEDNKVNTWAA
SHVGPANLTFGFGNHVCPGRFFAVRVMKIIFSKLIYEYDISWDRTEPGQPARFKIEGLSVPNHLQSIY
LKKRVPSS*
>CYP5125F1|11336|Dibbae1
MNGLEVQNEMKSNGNVHTRVFRVILTSHLEKLQPLLRDTISRAFSHEIDSAIRVEENWRKLHGFSM
AKRLITNVNSLVFFGDKLSQEPEFLLAAEQYLDDLFITAEIIRLVSAFLGPLVAPILMHQHKASKTMV
RFLTPVVEQRIRQSGNRGVAEQKHIDCIQFFVDATSRKKEWNATKIIQVLLGIWFASVHQPAMCLV
YALDDLCDHSEHIDLLREEFKHSAQNGGGNLPLLDSFLKESARLHPSDAISVRRLVLRPYTFSDGTH
VSAGDVACIPLQAILIDEKHYPNSMAFDAFRFLDQTSKANSTPFTNASAQFPLWGLGRHAW*
>CYP5180E1|11342|Dibbae1
MDSFSGQAMALVLGLILVSGLCLEIYEAIRLKGFLTAKLKAFRQLGRRCLDVLTDFLFNFEKTLENID
PNEPTSLRTRLYGRTLQILGPAQFEALRPFMQSKLETVFRERLGGAATGKGWISVKMAPLLRQAAIE
TMAITIFGVAMPNEPSFRAALFQYPRDMVRTMAGQQMTPKFLAPLVRLVATKNGQALDVLISRLM
RVTKSKGQDWDEAVEIKLLSVLQMLMDSISESPYWTPYTLAQAVVGIWFAAAHQPWVHIHFILLEL
CERPECVELLRNELAATTSLDYNTIDKLPSNLDSIRRKALRPFNFSGGGPHVPEGSVACISQWDILHD
PANYLNPFLFDPTRFIHTNVAGQSQTASDRVTEISENFPMWGYGSRACPGRFHAVFVIKLVLVHTLR
NYDFRLEDEKAPRWWHWDTFKMPYNNTKMEFRKREQHRQ*
>CYP5077B6|11408|Dibbae1
MYFAIPQTQSTTNFHGHLSLLFLALLPLFLFLASVIQCYFRLRHIPGPFPAKFTNIPRLLWVKSNHAH
DTHIALHRKYGPLVRFGPNMVSVGDPAEIGKIYGFGKPWSKSDFYRALLFRSRGKALPGIFATQDEN
LHRILKKPVSAVYSMSNLVSFEPYVDNTMRVFCSQLKTRFAENSREHSKICDLGEWLQMFAFDVIGE
ITFSKRLGFLDKGEDVDGIMSQIWNTFRKTSLQFLRGSRKSPGVVFATSRIQERKDLQRGTEKNDWK
VNDRDFLSRFMEIEAKDRTVPPEALLVWTTSNITAGSDTTAILLRTLFYNLLTHPQTLHRLRDELDN
AARTGNLDELASWKQTRDLAYLDACIKEAGRLHPPFGLPLERVVPPEGAEICGQFLKGGTIVGISG
WVAHRNEESFGQDSDLWRPERWLCDKESRKRMEASLLTFGAGHRSCLGKNISYLEICKVVPTLLRH
FDMDLVGPEEDGYWDVQNRWFVQQFGLKVRLNSRVEAH*
>CYP6431B1|11430|Dibbae1
MSILVWTSLFLCGVGSLLGVLRSIARRAKHIPELRSTSGWFGVWEDGMRYVKDSPGVLSEGYEKSTIT
FQPFKDLTHRLQVSQTSHTISPIVQNDKFHFETISSALTPSLEHKVGDILDEIKHAFEDEIGTSTEWKSF
TAAMKAHRIATRTANRVLVGAPLCRNEEYLQMSIRYTIDVFGGADKLRKYPEFLRGLMSKLVTRVA

EQQKIARKHLIPYIEERLAKQSKADEGKGSDSKPSDSLQWVMDAAPNDLERDPQRLLLRLLHINVA
AVHTTSVTFLNCLFDLALHPEIHEELRQEIKENVASFGWTKKTLLNLKKLDSFMLESQRLAPIASSQ
MTRYAVKDFIFPDGTLIPAGNWVLAPMYPMSLDDDLFPEAKKFDAFRFSRLREQPGEGNRHQFVST
SASHINFGHGKRACPGRFFASNEIKLLLAYTLSSYNIKYTEDQAPPVPFWYDRSRRPSQSAKISFRFRN
EAL*
>CYP617G19|11501|Dibbae1
MARGGQIIALSVLGSAVLLVNIKPEYSVKASYLWTFLTFYSIQFFARSTWSWYIYPNFLSPFRHLPEPK
DGDPLMGQFRTIMKEPTGVPIARWVNSIPNEGLIRYRMLFNQERLLVTSPKAIGEVLVTKNYDFEKP
PELKRQRKHLMPAFSFRHIKDLYPTFWTKSVEMVKAIGQVAQTGAANTGQEKGEPTPIIEVADWAS
RVTLDIIGVAGMGQDFGALQDPNTELNKTYRAIFSPNAQARILGILGFFIPFFIVRMIPVKRNADVVT
ASQSIRKVCRELIQSKQRKLNSKEPVNPDILTVALQSGGFTEDNLVDQLMTFLAAGHETTATGVTW
AVLHLCQNPDVQTKLRAEIRANLPSVDSSTEVTSEMFDKLPYLHAVCNEVLRVNCPVTLTRRAAVK
DTSILDQFVPKGTDIIICPLATNTSTALWGPDAAKFTPDRWMAPGMANSGGAESNYSNLTFLHGPR
SCIGQGFSKAEFACILAALVGRFEMELEDPYKGVEVGGGITAKPKGGLRVKVKALEGW*
>CYP65FK1|11701|Dibbae1
MSVASFTSLAWAHPLIIASLLLLVVRLLAVVGDIEACDVLIATTIYRLYFHPLSRFPGPTLWALSYIPYS
LAIRRGDLVYYTEKFHSKYGEIVRLKPDEVSFINPQAWQDIYGIKLGGKRNFRKDPMRYLPAVNGTY
SIIGCPTETTHARYRRLLSHAFSDRALRAQEPLLQFYVSLLMQKLQKLSDANEPIIILDWLNYTTFDIIS
DLAFGSSFNCLQTGTYHPWVRMLFSHFKALALISSVMYFPWLFKALMYTLPKAVKQRRIDHFNFAR
EKVHERMEAGSETGRPDFMSYVLRYGEGISTSDGKDSSGRERERERERERPEKGMTLPEIEATLAIIIVAG
AETTATSLTGIISYLIKPQYASTVLKKLVEEVRGMFEREEDITAEEVGKLTYLNAVIEEGLRLCPPVPTG
LPRVVPEGGDYVCGEWLPENTAVSYASWAATHSPTHFHDPEIFAPERWLASSSTSSSHSSPSLSPSPY
ANDTLPASQPFSVGPRNCIGKNLAYLEMRLILARLLWGFELEGVESGMGVGKGEGGAEWRDQKV
YTLWEKRPFGVRLRRRRG*
>CYP5570C1|11773|Dibbae1
MAASKYRGLGNPFAATAITPLLVLAAYQWLAQLQIDVQTRKIGLQLIACTLASTILSVATYRLTLHP
LAKYPGPWWAGVTDWYNVYRCLTGDRHIDFYRIHQKYVSLVQVFSTEKALGQFVRFGPNRISINT
NTALRDIYAVNANNQKSHYYDSYKHFFKVPMSMTTIDKKAHSFKRRIIARALTNRAPESEELMIRNI
RTFCRMLVDNPANDDWNSAKDMTTLVAYVTSDIMSDATFSRNWNMLNSKENRYIVELLATGVLG
VNMSGYMPAILALKLDKVFFPHIVRQVYKFEELSRAQSDWRAQQPRSANHRDLFAALLDARDPET
GLGFTHEENISEAGLLIIAGSDTMATGITSTIFYLLHYPLTYERVVAEVRCAFGDVEDIKAGSALNNC
TYLKACLDEAMRLTPGVGGLLPRETLSGGIIIDGEFFPQGVDLGVPHYAIHHNAEYYPEPFRFKPER
WLIEGSTRKEDVTLAQSAYCPFSLGRASCTGKQIAYQEMTTILARIMWLYDMRLQDASMCEGRRDL
GWGRHIREEFQVYDNFTSSHKGPMVEFKPATR*
>CYP5328K1|842|Dibbae1
MLLELLVVLPLLWAVYSCVALVRNLQQARTIGVPIRICPVSPTNSLWFIFEPLVLHVLDHLPFELGSF
GRYSRRGWHFYDKARSHIELGDVWVLVTPRETWLHVADPEAINEIFARRIDFVRPTELYNMRQVGL
TDWPRHRKIIAAPFNENTNTLVWTESISQAREMLVSWVKCGRTGTLGLAQDTRTLSLNVLAATGFR
RSYKFYRATEATTPSKIEPRSYQEALSIILENTLLIMVTPPKLLFLPFVPKSWAVIGQAISEFKNYITEML
NHEMSLLKDGKSGTGTLMTSLVRASGIDEKDIDCGGNPKGLTVSEVLGNIFFINFAGHDTTANSLSY
ALLLLSAHPEVQTWVSEEIEKLTDDECVTYEKAFPRLKRCQAVLLETIRLFPPLPALPKWTDNKPQSL
RVSGKTLHIPSETMVVSSLVAVHTHPKFWSPDPLVWRPSRWISSSATAKPNALSGQIACEQLYSPPK
GVYFPWSEGLQNCPGRKYAQVEFSAVLMCLFKSHRVEVVPEKGENVEAARSRVYHVVEDSEQVLL
LRMRKADSVRLTWREVQ*
>CYP505A70|1661|Dibbae1
MGTPIPQPPELPLLGNAKDIDPQNILVSQMRLADLYGPIYKLRIGGRERVTVGSYEFLNQICDEKHFH
KELSGALMQVRNGIGDGLFTAHNFEENWALAHRILMPAFGPLPIRDMFDEMHDIASQLLSKWARY
GPEYRISVTDDFTRLTLDSIALCAMDTRFNSFYRENLHPFVDAMVFFLKESGNRTTRPEFLSNLLYRA
DTQKYWDSIKLMRDVADHVIQDRRAHPSDKKDLVAAFIHGKDPKTGKSLSDENIANNMITFLIAG
HETTSGLLSFMFVQLLKNPVAYKAVQREVDEVIGQGPVTYEHMSKIPYITACLRETLRLYPTAPAFSV
VSNDKDRPTFLGKDRYEVPPNATITALLPRIHRDPAVWGEDAEDFRPERMLDENFNKLPPNCWKP

FGNGVRACIGRPFAWQEALLVTCMLFQNFDFIPEDPNYQLTYSQSLTIKPANFYMKAALRSGIDAIS
LERGLYGAPAKKDLAAKDKKIEDMATSGKPKKPMSIFFGSNTGTCQSLASTLANAAAGHGYDATV
LAMDMAAESVPKDHPVVLITASYEGQPPDNAARFVTWLEGLKGNELTGVKYAVYGCGHRDWQT
TFQRIPALTDKLFDERGAKRLAPRGYSDAADNDIFNDFDKWADKTFWPAIVSEYGGGAENDAEIT
GLDIEISTHIRSSDLRQDVSEAIVEDQVATIAKTIPEDETRNKLLAISKSEDCINMLKRASPLDLLETYS
TAALPFSSFLPMLPPMRIRQYSISSSPLVDPETCTLTYSVLDTEKDGRRYLGIASNYLSELQKGDLARV
AVRPSHQAFHLPLDIANVPVLMICAGSGLAPFRGFVQERAKQIEAGRTVAPALLFVGCRHPERDRL
YAAEFSRWESLGAVKLYYAFSKDTSSGKGCKYVADRLWAEREEARALFDAGAKIFVCGSGAVGDS
VREMILKIFLEGMKARGQEKTEEEARKWFMGIRNERFASDVFT*
>CYP6001C40|1702|Dibbae1
MLRRFSSTFNKKKKEEGQVNGTSKVNGTSGMENGASGKQLAPPAHTEHKDEHAVKREDVATSLE
NFAQLIHAARRPLPTQTGDGSYLDHPVPSGLMQDLKNLGFKDVKTLMQVMRTKTAGGYQDDKTY
LMERVIQLVSGLPDLSKTRVDLTNSFIDELWNSLLHPPLSYLGDKFKFRQPDGSYNNIMYPHLGAAN
APYARSVFPGTLQLGSLPDPALIFDSIYARKKFRRHPNNVSSAFFYHASIIIHDLFRTSHKDFNISETSS
YLDLSPLYGSNQEEQNLVRTFKDGKMKPDCFSEKRLLGFPPGTGVWLIMFNRFHNYVVEQLAAINE
GGRFNKPHEGLTVEQNQKLWEHYDEELFQTGRLITCGLYMNVTLMDYLRTIVNLNRSNTTWTLDP
RVEMGKVFGHDGTPRGVGNQVSAEFNLAYRWHSCISEADEKWTEGLYYELFGKGPNDVSFPELLQ
GLGKWEASIPDDPMARQFAHIKRGPDGKFNDDDLVKILNDAIEQPAGAFGANNVPVALRAVEILG
MQQARKWNVGSLNEFRKFFGLKAHETFEEINPDPEVAAQLRHLYEHPDFVEMYPGIVAESAKTPM
VPGVGIAPTFTISRAILSDAVALIDYHPQNLTNWGYSEVQYDLHINQGCVLYKLFLRAFPNHYKSDSI
YAHYPMTIPSENRKIMKTLGREDDYSYDRPQFIPPRVNLTSYVGAKYILERPQEFNVTWGDATGAL
MGKGGLDFMLSGDTAFHTQQRKTMAKALYRDNWHKEVKEFYEYITTRLLHDNACKIAGHNQVD
ITRDVGNIAHIHFAANVFSLPLKSADHPHGIYSEHEMYTVFAIIFVAIFFDFDPAKSFPLRMGSKVITQ
QLGKLIEANVKMVNSTGWVAGIVDPLMKNNNPLTDYGVHMVRRLLESGLGVHEITWSQVLPTAV
AMVANQAQVFTQLLDYYLSEEGIKHIPDIYRLAHEDTPEADEKILHYAMEGIRLNGTFGSYRRSTITT
TIDDGGKPVSVKPGDNVFCSFVGANRDPTIFPEPDAVRIDRPMDSYIHYGVGPHSCLGGDASKVAL
TAMLKTVGKLKNLRRAPGPQGVMKKVPRPGGFYVYMTENQGSYFPFPTTMKIHWDGELPPLKAR
HETAKVTTSGSGPAPAAPKKK*
>CYP6352B1|2110|Dibbae1
MSTITLPLMHLPGLRALSMENCIILGAAFAVLYLINSYFVNPLSRIPSIHWSAPLSRAWYTCAIYSKPR
THIHLKAHRNGGAFQPLVRIGPNEVSVMSTAAIKTVYESGNFNRTSWYEAFMKWGQHIMFTFQSA
QDHQNRRRLYMKNYNRTAIFGTHCQHIIKSRSAKLVNFLLSQTSDPTKSTTRPFLARSLCRALQLDV
VTAFTFSDADGTRFLDRLDASLYNTVDEVGMDVIDLFYDERNGSYLFWEIEAPYKYFINFFSKISRES
KAAHARAEKWTSGLISRFEARYQNCKDGAEQQRLVETSIYGKMLAFKSANGLGQIPWEQRAAEIL
DHIGAGQENLPAVLDFIIRQICIYPDVQDAIREELKTLPGGIEAYNPVDLENLPYLNAVVLEGLRLVQ
VVESYQPRFVPKGGCTIEGYYIPEGTVISSQPFLIHRQEEIFPEPETFLPGRWLVEDKEKKRVLLKSMW
AFSSGPRGCIGKDLAMATMKTAIGSIYSHFSTQLMSEDRPRRSWESHYAMSEVTFTRLY*
>CYP6232A2|3412|Dibbae1
MYSIASLVLCTIAGLLLFRYLQIAVFSQLSAVPNAHFLAPYTRYWLKYLKVTGQEHHVRDAAHKRL
GPVIRLGPNELSVNCIENGVSTVYGGGSGGWDKGAWYGVFENYGHSFMFAMAEPKPHLERRRML
ANVYTKTYIQSSPELTDTLNTMLSERLLPQMRSWAETGKEVDVHRENKAFIMDCGTAYIFGLGNGT
NFIQKPEEKALLRDFELGISGLFWFAEAPSFVKFMGCFGIKLIRDEVIKSFQVMEDLCLKMSEKAKLA
MQHNSAKVRSAPPTVYAQLRKKLEEAKNSPDAINTMTAAELLDHTQAGPDPAGITLTYVMAELSQ
NPAARHRLREELRTLQRKDAIWSSPRALDDLPYLDAVVMETMRLHPGAFGPFPRLVPPGGTTLGRF
SNIPQGTTVSTSAYSLHRNPSVFPEPERWRPERWLDSGAEARKEMQKWFWVFGSGSRMCIGNHLA
VRMMKSFIAAVYSEFDISLVDGKIPSQTDAVIAYPEGNMCLMRFHKIQPVLV*

>CYP619F3|7832|Dibbae1
MFSLLIFECVGALVLGVIYAVLSLSKGPPTLPLLGNLHQVPKTGAHLKYSLSQPVLQFTEWAKEYGG
IFSLKFGPANAVVITDRGLVKSLIDKKGAKYNQRPQSYVAYDLITKGDHVLVMQMGEPWRVTRKT

LHQLFMESKCENEHISLQNAEAIQMLRDICNTPEDLMLHPKRYSNSIIVSLLYGFRTPSVGSKHMKE
LYEIMEMFSSIMEVGATPPVDILPILKYMPERFWRNWQTRAKKVGGAMGALYEKMLQRVLERREK
FGSKGSMLDTVLDQQDKLQLSPNALRFFCGASMEGGSDTSASMILVFITAMLKYPDIQKKAQGEID
AVVNEDRTPLWSDFDKLPYVNMIVKECMRWRPVTPLSLPHSTAEDDFVEGMLIPKGTTVIMNVWG
IHMDPTRFPSPDTFNPENYRGKTLLAAEYAASNDPENRDHYGYGAGRRICSGIHLAERGMWQAM
AKLLWAFDFSELPSKPVDVSKETGYTEGFLHCPLDFECDVQVRSAKRKETIMREFAVAEKEVFSKYE
DA*
>CYP6524B1|8624|Dibbae1
MYLIGFFALFTMWYFSSLPKNYFLARKTGLPIIVCPVNPVNIFWLVVSVVLEPTFARYLPSFIYDQLKV
GIYGWEFRFRYTVNAKLGPAFVLVTPARNESNGSEWQRHRKIVAPVFNERIMNSVWDESSAQARD
MLEAVFKRPAGSKASLSGEKESDDMVEGVRTIALNVFGFIVYGDRQSFAHTSTKAEAPPGYQLTFM
ESILSILNNHFISIFVPAHILTLSWMPRSIQKMGIATAEFPKHTKDFIAKERNSPSAQNTLLSVMVKTA
DNERHQSAKPGRLASYLSEDEITGNLFNFTLAGFDTTSNTMAYALIILAIQPKWQDWITQEIDQVAR
LHPDASYEDTFPLLTRCLALMYETLRLYTPALHIPRCTATTQTSPVYIPAKTTVHLALACMHVSPAL
YGPDPLAFRPTRWIAAPSPGSDGETLISPPRGTFMPFSSGPRSCPGSKMAQVEFVGVMREIFANWRV
EAAQKPGETGVMARERLGKVVENSQPKITLQVKNPRDVLLKWIRR*
>CYP578AB1|9170|Dibbae1
MLPVTVLLSAAAIVSICFYKYFLYPVFLSPLSQIPNAHFTASFSSLWILRVRSKSTENHTLLSCHQKYGP
VIRVSPNEISINCVDNGIRTVYAGGFEKTDWYPKSFGNYDIPNMFSTAESKPHSIRKRMISNIYSKSYL
QTSEDMQSISQTILSQRLLPSLDSFAQSQIPIDVLEWNFAIFMDFVTAYLFGLGNGSNFLLDIEYRRH
WLSIYNDRNENNFGPQELWKSILYLFGLKIVPDKVAAANKEIEEFTLKMCQAADATITTDHKTDTS
TLKTSPVVYQQLFQGLERADLDPWSKYPKDLTVASEMLDHLAAGHETSAITLTYLMHELSKRPELQ
DALRAELLTLTPPITYPLSRTGDLMQIPNPRSTDSLPLLQAILLETLRRHAAIPGPQPRITPYNQPTPIK
LGQYSNIPGGVRVSALAYTLHRNPAVFPNPEEWKPERWLHASKEQRTEMDRWFWAFGSGGRMCI
GKNLALQQLNLVIAAIYTNFRTKIIDDSGIEQEDTYTARPTGNKLVLKFERAQ*
>CYP578AC1|9807|Dibbae1
MQHNDVWTHRFDAWDENVLTDGDDGPWSTFAIGLGTPAQYVRVLISTTCPQIWAVDWQGCAPT
DTLDCGNSRGGLFYSKESSTWQDKGLYQLDEELNLGYSGAGDFGLDTVALGYPGSGAVVLDSQIIG
AIALEEFYIATWGIAPRPTKLTTIDPINSYQSLKQVLGSLTIGAYDTSRVAPNNLSFGSAEDVSRDRIVR
IQSIVASISSMNGTRASSTSLLPNAAFEKLFDIQWGPVPDLNLVNNTLHSSLVAQNACIAFQLGTDTV
TQHTIDITLPYASFDLQATATYPGISNATACYARYLIADYERSNFAINQCVFGEGAQQDIRPILSVNV
TNTTARPIPLGPSNPGHLSVGSVAAIAICSVLAIAMIASGLGYRIWQKRKVQGIHWPGEETGNDKSP
RIELEDKSGLREDLEGSNQYRNEPENTEAPLDELGMSIAVVLASVSLFSFLIYKYIVFPAFLSPLSKIPN
AHFTSPISPAWIWWRRRTQQENVSIFGAHQEKGPIVRLGPNELSVNSVDGLRQVYTGGYAKTDFYI
DEFENFRTPNLVSMLEHRPHSVQKRMLTNVYSKSYLQGSLDLEIICRVMIYERLLPVLHSNAESGTSL
NVLELMLGTGMDFMSAYLFGLENGTDFIRDAVGRKQHLEAYRIKGYRLPGADQASKELEAHCLSM
CQAAEQTMAGIFQTTTKPVVYSKMFSELSKSDLSYASSSRRDIIAASEMLDHLLAGRETVGVTLSYL
MLELSRRPDLISKLRSELLTLSPQITYSSTKGNHSDINGITLPSHQTIDALPLLNAVVYETLRLHGIAAG
PQPRITPPNATIEGYSNIPAGVRISSSAYSLHRNPGVFPDPEVFKPERWMPGETTPKQSEAMRRWFW
AFSNGGRMCIGNNFALLELKLVIAAVYSNYLTSIVDASGMEHEDGYLAAPVGDKLVLNFHCA*
>CYP6002C1|10274|Dibbae1
MPLPSVVETAGPLVKDIEEKLKRYIKDLRSQARHLPEDVGILNSALEATLENGLDDDKNYLTERIIRL
ASDLPDGSPSADVLTSAFIRTLWDNLQHPPLSYLGDTFKYRSADGSGNNVMYPNLGKADSVYARSV
TPRHSQLGLRPDPTTIFDTLFARKGPAKEHPNKISSMLFYLATIIIHDLFRTCDKDRTKHANSAYLDL
GPLYGHNQAQQDAVRTFKDGTLKPDTFSEPRILGQPPGVSALLVCFNRFHNYVVGQLAEVNEHGK
FSLPAGMTPEHEGYKAAQSKRDNDLFQTARLITCGLYINIILGDYVTTILNLNRSPTTWRLDPRKNF
NDIFDVTGTPQGIGNQVSIEFNLIYRWHSATSRKDEKWAQGFFEQLFPGKDPRTLSLAEFRNGFQL
WAHSLDKDPGKWTFNGLKRTKGGKFSDADILKLLVEGTKDIAGAFGAQNVPTVLKLVEVMGIEQ
AREWQVASLNEVRKFFGMTPHKAFEDISSIPEIAATLETLYGHPDNVELYPGVTVEDAKDPLAPGSG
LCPGYTISRAILSDAVALVRSDRFYTVDYSPANLTSWGFNEVSADPKIAQGGVMFKLLMRALPGFYR
SNSVYAMYPFTIPSENQKILRDLGVESDYDFTQPCFVGPPLPVVTYSGAKEVLKDQLNFKVPWGPHI

SYMTHHDYMLGGDKSANAEQRRFVQEAMFTKAEDFLEIRKFYEAVTTKLIREHSKTLGGYWQLDA
VKHVGNPSHADFAARLFGFSDTFEDKELCQILATLFTWVFLDLDPVKTYELRLAALEATTKLTAAV
ETICDKVNRDGLDSDQDTDLEGRDSNLADYGTRLVKRLFAGGKTPDQVVSMIVPTAAAAYGPPSQ
GIAQVLDLFLTPPYSKHWPEIQRLANNDSKDSFEKLIKYGLEAFRLSTPAYGVVRACTAESPVAIHD
GERTVTARAGDLIYVDFATSSRDRSVFPNPSEIDLNRPDDSYIHFGWGPHACLGRPIAEVALAAQLK
VFAKLKNLRRARGLQGVMKSGTVPGTDVRVFMTEDWSSWSALPTTLKVEHDGFEGEGKEIDA
>CYP59AE2|10353|Dibbae1
METTSVKAVASPLRVISGVLVACVVVFLYKLYKERSRALRLQRLGYSMPPLNPILGHLPVLAQMMS
KLPKDAHPHYLPDQLRREYPDLGPMYYIDAWPFATFTLVLASPSTLAQVTTEHNSPKFPAIRDFLYP
LTNGRDIVSMDGQEWKTWRSIFNPGFSASHLMTMVPEMVKETVIFCRILEEHAEKQDAFAMKNLT
DDLAMDIIGKVVLLQDRIENSLLIPSSDSDLNVQQSVNPIVSALRQQVRWMAFGSEGNPLQQYHPL
RPFVHIYNTWKINKYISPEVDARFTCRKDQRRGKENKPGKSVIDLALTAYLKQNSTDTGDTSSQSM
DPTFKELAINQVKLFLFSGHDTTSSSICYTLYLLSINPGVLSRVRAEHDEVFGMDPKQAATRITEDPYL
INKLPYTTAVIKESLRLYPAASSTRSGEPGYTITDSHGHKYPTEDCLIWMISHAIQRDPAYWPRADDF
IPERWLTEPGDPMYPVKGAWRPFEHGPRACIGIELSMIELKLVVALVARRFDIEAVYGEVDAIQQRG
KGESRTVVGERAYQLGMTGQPSEFLPCRVKISA*
>CYP59Y1|11092|Dibbae1
MPPWSPIFGHLPAIAPILKTLPKKAQQAYYFNELVKDFEDSDTLFYLDLWPFSTPLCLVSSPSIAIQIAS
QQHDLIKPPTIEYFFRPLAGGDNLFTMNGPEWKRSHALFVPGFQANYLLSQMSHIVEEALVYVTVL
EEHAVKKDMFSLDEVDQSMLLIRAYNPLTSAMRNQVSWLCLEDELNPLVRWNPLRPLMFWWNG
YKMDRYISKELDKRFAERRRSERDSTESSRSIIDLALESYIVENPSMDTAKGLNDSFKSWACAQIRLFL
FAGHDSTASTICYIYYLLFTHTDALERIRVEHDEVFGSDITKLPTLLNDRPHLINQLPYTIAVIKEAMR
LFPPASGIRGGRPDVYLDVNGKRYPTEGTSVWIVHSVLHRHPKYWKDPDSFIPERWLVGPEHPLYP
VKGAWRPFEFGPRNCIGQTLVMLDVSVTLVMTLRRFDIHNAYAEWDSLHPGNAQEIKSVRGERAY
QISLGGAHPADGFPCRVSLREP*
>CYP6677C1|11371|Dibbae1
MIVRNIGLPIVFSPIYKMNVPWLVACRYTRIQYHLQLLPFGLGRWSRYTFLGWVFDDKYALHKELG
PAFVVVSPGGNDVFVADAEATHDVTKRWKDFVKPKEIYKVLEPFGRNLDTVEGDDWSRHRRLTA
PSFNEKSSALNWAETLTQAQQMSTIWLQKGSEGTRHTVEDTATLALNVLQAAGYGLYFPFHEDSP
EIPPGHSMTYRDAMATILSLYTLYMLIPISSMSSPWMPQFVRTLGHATKEFMMHNEEMLRNERASIS
KREQGTANLLSALVRASDEAQKTTVGGRSQGLSEEEIFGNLFIYGLAGHETTANVLSYTMCMMAIY
PEWQEWVAEEIAFVTKDNPDPKNWKYEEMHLKLKRCLAVMFETLRNYGSVVFIPKETNENPQDL
RIAGTTYMIPPKTSVNLNIHAVHVSPKHYSPDPLTWRPSRWIAGHAVAPTAHKDISVPGNESLIDPP
AGTFTPWTDGPRICPGKKFAQVEFTAAMAVIFQRMRVEPVVRPGETLEAKKKEVLAMCEDSQLQP
NTLQMREPGKVALRWREVGR*
>CYP534M1|11478|Dibbae1
MWSYIGLGVLLIVGLWTSATYRSFAKNLAAAKSSNIPYVVVPVYSFAPLWLAINPFLVPYLQKLPAR
WIDPWLLFVTGDWAWHNKYDVFRKMGSDNFMTVSPGGNMLWTADAPAITEIVARRNDFPKPVY
IYKLIDIFGKNVISSEGQIWRQHRKITSPPFTEKNNHMVWSESLHQTEAMLKSWTGPPGTPPRTIATL
AADTMRLSLHVISRAGFGVRLPWPGVENEQAVEVPGMTEGHTMTYVEALTELVEHLFWIILMPH
WLLRRIPFAGPARAWQCFSEWSRYMQEMYESKKLDIQEGREKEEGMDLLGAVMKGANVVQPKAI
GNGAAKSAANSSALEKGQKPAPTTRALTDQEIFGNAFVFILAGHETTANSLHFSIVLLAMYPSAQR
ALQRDLDRIFSFKPVSEWDYERDVPELFAGMVGAVMNEELRMIPPVIGIPKSTFANQPQEILLDGKL
CTVPGGTVVNLSAVAVQRNPKYWPSSDPNVDPVTDLNHFRPERWITESSPAYSCIQKPASHTKSMN
GHAANGAANGHAIKPNSVPIPKIGIATETRASTPSDDLHLNTAPDTSLALYKPQRGSYIPFSEGFRA
CLGRRFAQVEVLAVLAVIFSQYTVELAVDEWASDEVVDGWERGGEERRAVWAKARETARGRMDE
GMSSLITLKMRAGEVGVRFVKRGTGRERFVFEDESW*
>CYP6259B1|1963|Dibbae1
MAYYALELLGALAGLYYLAKTLSLRYRFSKAARQHGCKPAPRYSHWDPFFGLDLFIRLRKAHVAG
QSSQTHAKIHEQYGKTLEMKTMTGTQVRTAHPENAQAICASMFDEWEVTPLRGRIAAPFIESGIFT
HDGSIWKRARALVRPIFNKSEIADLGAFDIHVGRFLRLIPKDLSTFDALALSKRLFLDTSTEFLFGQSIE

SLLPQTPFDTAEFMRAFDASLQGVALRILAGPLRFLFLLDPTWKRNYTRVHDFVDKRVAIALERQRS
MSTISKSSEKASSEKYILLNEMAMVTQDPLDLRAQILNVFFPARDTSAITFSNILFELARHPDAWEYL
RSEVLSIDPSQKLTFDFLKTLKSTKAIINETLRLHLATSRVSRAAARDTILPVGGGLDGRSPLFIPKGRQ
VSIDLHTLHRDPSIWGEDAEEFKPQRWGPGRPLWEAKWQYEPFLGGPRMCPAQNQVLTQLSYLLV
RIAQEFRSIENRDDVYEYVETIRLTNESKRGVQIALVPA*
>CYP584AV2|4449|Dibbae1
MVYKATSEVLYALLGLAAFYLLARRIAIWLGRRKMIRELGCQMPPSLPSMDPLLGLDSVLQTIRSVK
ENKGNMTLKQQLDTYGLTFRTNLYGRTKFFSADPQNLQAILSTDFESWGIQPMRLFEFEPLAGKGI
MTTDGSLWANSRALIKNTFSRTQIADLSSFEGHVQRLIKLIPRDGSTINLQPLFSRLALDSSTEFLFGKS
VESLSPSCSLDAQAFLAAYNYAQAGIGKRMKLPKWNILTYDPKFWESCKIARSFVEGYVDEAIRQC
KSARGGEKLKLVLAYELARVTSDRDVIRNELLNVFLPGHDATAVALTNVFFMLARHPEVWTKLRE
EVVGLDISELTFENLKGLKYLRWVINETMRLHPVVGESRRIALRDTVLPTGGGPNGTSPILAKKGDI
MGTSFYALHRLKDVYGEDADRFDPERWATLRPRHWAYLPFSGGPRVCIGQQLALTETAYATVRIV
QTFGAIENRDPVNEFVELYKITTESKNGAKVALIPA*
>CYP52AA11|5277|Dibbae1
MQQWVLALVWAFVSFVIYTYVSSILAERRYLAEMDRRGCKMPVMQTAVLPGSIDRMYESLKADRN
KSFPNVVQKRFQDVGDKTFTWKILGHRGFFTADPKLVQAILATQFNDFYLGPTRRGNFMPLLGNGI
FTADGKMWEHSRAMLRPQFARDQISDLRLEEKHVQNLMRALPVDGTGWTKTTDMQVLFFRYKID
SACEFLFGESVDSQILNLPENASKGSSGSSATDESVFATSFDKSQAYTASRARLLDFYWLYSPKDYKE
SNKRVHQFVDYFVQKALKGDFHKEKELEEGHDKDRYVFLEQLVSETRDPIELRSQLLNILLAARDT
TASLLGYLFWILARNPEAFKKMRSTILENFGSYENPRQITFVTLKNCQYLQHCINETLRLYPVVPANS
RRAAKDTVLPLGGGPDGKSPIFIKKDAQVDYSVFVMHRRKDLWGEDAEEFKPERWVGRKPGWEY
LPFNGGPRICLGQQLALTEASYVTTRLLQRFDAIENCGPLDKEPKHALTLTSCPADGVVVRLHEAK
A*
>CYP539A54|7787|Dibbae1
MYAGANYVEWELYDGLFRKYGRGQWTMEGTVAGRRTVFTAHPENLKAVLATQFSDYGKGERFH
QEWEPFLGDSIFTTDGDQWHASRHLIRPQFIKERVSDLQILETHVHVLLKTIELEGSGKAGGVPGRM
DVDMNDLFLRYTLDSATDFLLGTSVDSLENPEQSFAKAFGEVQRVQNMIARAGPLNVLIPRRTYYA
SLIDVNAFVEPYIEQALHLSQAELESKTKSEYTFTHALATFTRDRKMLRDQIVAVLLAGRDTTASALS
WTFHELARRPEVVKKLRKEIIDTVGLTRPPTYQDLKSMRYLQNTVSETLRLYPPVPFNVRFALKDTT
LPFGGGPTGLSPIGIPAGTIIVYSTLVMQRRPDVYPAEWSTEGSNNPLRPLNWNPDRWLNWQPKPW
TYIPFNGGPRICVGQQFALTEIAYTLVRMLQEIVRIEDLGGLGAEERDEKNLKCEVVMQPVRGVKVG
LWRS*
>CYP584AA2|7868|Dibbae1
MAIFLSQVFLPAVGVLGFLFLVSTVRTWYTRRQIIKKNGCLPPKRLPQKDNIFGSDVVRENLKAARE
FGFLEKLRSRHEKYGPTFTTNTFFRTTLYTCDPKILQTVLATQFQDFGMGPLRRSSARPLLGKGIFTQ
DNKGWEHQRGLIRPAFLRAQITDFNIYESHVDQLISLIPTDGTPVDLQQLFFRMVLDSNTEYLFGESV
GLLSPNASQFAQDFHNALDYAQKGTIFRLRLGKIMWIHHDKKFREACATVHAFGDKFVKKALDIR
HRRINFGDEKTETGRKQYVFLDELAKDCDDPVMLSDQIVNMLLAARDTTAGLLSFIFFMLARRPDV
WEKLRLEVLEGWEEPMNYEALQRMTYLRYVIHETLRLFPPIATNSRMANKDVAIPYGGGPDGKSPL
FMKKNYVVTYSTFVMHRRKELFGEDADKFKPERWEKLRPGWEFLPFNGGPRICPGQRFAITEAGYT
TARLVKHFAHITNHDPTEFREQLTLSLTLNNGVKVAMGTA*
>CYP52AJ6|8878|Dibbae1
MRPIFAALLASLSTLIIYNYFKNKLIKRRRNAEATRRGCEPPPLLRSMGFDPTGLRRLVDNLRAVKED
RGPQYIVKSINEVGEHVHTIRVHVLDYEVLVTRDPENVKAMFATQAQDFDIGSFRTQNWKPLLGV
GIFTLQGEAWRHSRSLVRPQFSNEQISNLDLEERHVQALLGKLEPRENGWTNIVDLGPLFYNFTLDT
ATEFLYGHSVHSQNPTGHTSLPKIEGVENPNQAGFGPHIDGAKEWIYTKGILGKWNWLVYSKEFT
NHCKEVHKYVDWFVQLRLRSEAKHNAYTEAAENKQKFVLLNELAKTTQDPLALRNETLQLLNA
GRDTTGTLLGWIFYFLARHPHVYAKLRAIVLAEFGTDSDRIEFAKLRQCQYLHHCINEAFRMVAVV
PLNERVASHDTTLPQGGGIDGSKPIFLTKGTRVLISTYAMQHRPDIWGDDVEEFKPERWESRKSGW

EFIPFGGGPRKCLGQQFSITETSYVVVRFLQRYDKLENMEDEPIRLHHTVTNRSGTGVQVRLHQASG
A*
>CYP584AP1|9706|Dibbae1
MGKVIIWTNDPINLQAIHAVNFLDYGVQPLRRDATLPFLGEGVFTMDGPFWQHSRDIIRPTFTRNN
VANLSLFEAHFQRFLGLIARDGSTVDLMPLLCRLFLDTSTEFLFGESIGSLLPDTPFETEAFLNAFHYG
QNGMGKRLQLGKLMFLYLDPGWYRSIKIAHAFADKYVKKALDFRSAWLKDKEKEKDSFEKSSGER
YVLLQELAKETDNPVELRSQIIHVFLAGHDSTAITVGNAIFYLCRHQETWHKLRAEVLAVGNSKLTF
ELLKTMQYLQYIIKETLRLHPVAPTDTRYAYRDTILPTGGGPSGTSPILLYQGQLVGASYHALHRRSE
VWGPDAEAFRPERWETVRPGWNYLPFGGGPRICPGQNLALTETAYVLARMAREWSGCECRDETW
EWVENLNVSASSFNGVKVSLTPA*
>CYP52AJ4|9775|Dibbae1
MVTYTYGLLGLVVAVVLYNYAKSALEKRRRRLEAARRGCGPVPVLKNKDPFGLSTFISSLKATKDD
RGPQYVVETMDSIGKDVHTVQVRLLDYSLIVTRDPENVKAMFATQCGEFDIGASRQKSWEPLLGV
GIFTSTGERWKHSRALVRPQFAREQISDLDLEERHVQDMLNVLTKGKTDPSGWTNNVDLQPLFYN
FTLDTATEFLYGHSTHTQNPARRAQLPSLNGMDTPDRAKLSGHLDAGKYWIETRGAFWKWYWLI
SSKDFDYRCKEIHKFVDWFVQVRLRGQKEKLQPPMQSGSRKKFILLDELAEHTQNPRELRNETLSVL
TAGRDTTGALLGWVFYFLARHPRVFDKLRSIILQEFGNNHQTGEISFVKLRSCTYLQYCINEAFRVA
AVIPLNERVCNRDTTLPRGGGPDGSQPVFITKGTQVLIATYAMQHRADIWGADVEEFKPERWEGR
RVGWEFVPFGAGPRICLGQQFSLTETSYVIVRFLQRFDKIENMEPPGRIRLHHCIENRSGTGVQVRLH
EAAAC*
>CYP50112B1|10860|Dibbae1
MSRLLQVGLWAVCALCFLYAYDRLRRNLAYRRAALAKGCLEPRLYPHRDPVFGLDLFIAQMKNY
KHHRLLEQSCRWVEQCGDTYQTNSLGVASIKTIEPENVQTIFAHNFEDYGLGPLRTDIAGDFMGRG
IFTTDGKFWEHSRALVRPIFTKAQFADLNTLKVHVDRFISLIPRDGSTIDLLPLLHRLALDTSTEFLVG
TSVDSLLPSSSPELKHFKDAFDYAQRGIEMRAILGKLKVFYRNQKWNDAIDIVHAFFDKHVDRAITQ
VTAEKNALQEGQQAQRHVLLYDMAKQTQDRLDLRFQTLNVFIPAHESTGIAISNVLFHLSRNQVV
WKKLRTEILGLGSKPLNFENLKSLNYLRYVLNESHRLTPVIPMNTRICLQDSILPRGGGPDGKSPMLV
QKGSLVTANLWAMQRNKAIWGPDAEVFRPERWESIRPGWNYVPFLGGPRICPAQQMVLTESAYV
VVRLMQEFRAIESRDAEPWTESLKVTVANRNGVKVSLTPA*

***Graphis scripta* CBS 132367**
>CYP51167A1|9745|Grascr1
MTFMHDLFENIAQEIGDHVVEAVYDASIFEIFTLTCIIIAIGWHFRPTFYPYDEYPRIHRPVLSLPWSKS
VGESLRDGYEDVLKKGGDGFTMRWWSKDHLVYLKAAEPDQAVFLESISEKKALNLDSSSPDLFTHS
KRMIETVKRGLGATALIVPNVVEEVDTILEEVLNGRSTSHAFKASSLISSMSQKICCRFLISSELSNNPT
FLAKNARFTSSLFLNALAIKALPLGPFRDFLSWPLTFFHRRNLDSALSLLQPVVAHRLNNNFLDKNK
PITTSNGSPDPKANAPKTQLPPFSNNTKLYSDSIDCIISLPTASSLGSNSRQISLQLLHNLWLGSVPVA
HTITQMLYSLLSQPEYLDPLRTEISEMTQKYGWTEHALADLRLMDSFIREVGRMYPLTSPTCPRLTPS
STSEMWRFSDGLVVPPGTRVMWPGAEILRDEENFADDAATFE*
>CYP5238E1|11492|Grascr1
MEVNTLFSMFPLHFILAAGIVGPALATLLLLSPYPNGEHMFWKQQQWVGLKKQILARARATLRSIS
QTQALISQGYSKAPETLQFSKANLPFAIYSMASGPVIVVPPAQLKAVMNQHESLIDVHNPQNETLQ
AYYTVRDPDVYINDFHFNVVRNQLTRNLARFSEEIAEELALSFERYWGSSSDWTDVFAWQSLFTIVA
RSANRVYVGKELCRNEEFLENSRLYAVSVFGAATVINLCPEFSRPVLARLLTLFGRRPLAICKRICKPI
IEYRLEMALRAKSETAFSWDAPDDGLQWIIQECVKTEDPKQLDPNKITHRLLMLNLVSIHTTSFTLT
NVILDLYSSTPFEKYVEGIREECDCVFAEANGIWTRAAVSRLLRVDSAIKESMRLSGFGILGLPRRPRP
SMPMLSIHTDESLYPKATTFDAFRFSRPREAYLAEQKTDGRGSGSSSIIILSDGERVTVPENLGHGAIA
PVSNGHEEADQSASARRILSLKQQSVVSLDDGFLTFGHSRHACPGRFFAAHEMKLMIAHIVRHYDI
QRIPERPQSKTHEMKLMIAHIVRHYDIQRIPERPQSKTLVEVKLPDGWLKLKIRKKSGSCYGED*
>CYP65FJ2|442|Grascr1

MPSLGEAEGLVDANERDHARFRRLLANAFSEKALREQEPILQTYIELLIKKLHDQIDGPVGGVVDL
AEWVNFMTFDIIGDLTFGQSFGCLESGKLHPFVALLPGAARTMTYLLALKHAPQPIKLRLAADNDR
RDFITPILKANDEKGMTYPELESSINLLVTAGSETLATLFSGASFYLSQNPQVMQKLREEIRDAFESPA
DVTMGAVQSLPYLNAVIAESLRIYPPAALSLGRITPHGGAVISGHQIPAGTAVGITSWAATHSPQNF
TDPEEYAPERWLDDPRFNKDNRDASQPFSLGARNCIGKNLAYAEMRLVLARLAMDFDFELLPESR
NWIMQKLFTFWEKPPLMIKLLRPTTTCTYLGVIQKEGHPPESCWVFELLLYFLFPCISTCYYTVFPFD
AFASFSFFKMETNPSPHVSLEKMADSVGEKSSNSVSESVTNSTPDEAPVLGQEIQQYPSALVRWVIVI
ALLLGEFLIALDLTIVATAIPRITDEFESIEDVGWYGSAFFLTFAAYQSAWGKVYKYFEMRYAFFFSVF
IFGVGTLICALAPRSVALIVGRAITGVGAAGIVVPRKVPMYLALVGMDFSIASVAGPLLGGVLTEKLS
WRWCFWISLPVAGASLFVVLVFFRAPDSMKPTAATPREKFLQMDFQGTLLIMASLLCYMLALQYG
GTSQSWNSGTVVALLVLWIVLAIAFGAEQWYQQERAQMVPRIMTDRTIIGLSAFIVLINGTIFLMIYY
LPIYFQAIGGSSPAESGVQCVPLILAMSIALLISSAVVTRIGYFHPLMLIGGVCITVGSGLIYTLDMDSTS
GQYIGYQILTGFGNGICSQIPLIASIAFSKVQDIPLTTAMVLFKVYQLISGALSVSLAQTLFTNILIQKLP
IYAPTVDPARVIAAGASELRSAFSAEELTSTDTPSEKHTCWK*
>CYP5039E2|444|Grascr1
MTTWYEAYYDLWLGGQYVWKIGELHKEYGPVIRINPHEIHCNDPEFIDNIYAGSSRKTDKYRFTGR
RTQTKQSMVATIAHDTHRKRRGAMANFFSKQNVRNVEPAIQNSLNKLLWRMDRASRTGEAMPLS
YVFKAATSDIITKYAFGKSTNFMDQDNYNMPFFQAIEVLFTTSPALMHFPWLGPLLEALPVRVMKY
LMPGVADMYKMRQGWMDQIDDIKNSPDMSLGKGTIFEGVLASKLPNEEKETARLGHEAQLTVLA
GQDTTAVYQLLANPPCLAKLKAELETAFPDPHQPITFSECERLPYLGGVIAECLRVHPGVITRMARV
SPEVPVVYERHGKRYVFPAGTPMSMSSMHIHFHEDYFPDPHVWRPERWVDNPRLDKYLIAFSRGT
RNCLGINLAYQELYTLIGNVFRKYDLYDGTRTQKVPTMELYNTVRERDVDMNFDYIVPFPAKGSTG
PQIKTHAAAV*
>CYP5039D3|447|Grascr1
MESLKQLLSTDKTPTALVLGAGLAAWGLYFLALGIYRVYFSPLAKFPGPKLAAFTTLYQAYYDIVLG
GHYFRKIDRLHVQYGPIIRINPHELHVNDPTFIDELYAGNAKKRDKYKWAGRSVLLPDSLVATQPH
DLHRKRRAALNPFFSKANIRKLDPVVQHGVTSVLKRLEDCAKHAVVFPASLAYKAATCDIITEFCF
GVSTEYIERDDYEHTYFKAVDHHLKMSWMMTYIPGVGPFLDALPPILMGVIYPGLKQLWMMHSR
WTSQLDQIRASSKFDDGRTIFHGLLNSDLPPSEKSNFRLRQEAQLLVLAGQDTTAYTLSSLTYQLLA
NPDILRKLKAELAESIPDPNVPMNSADLEQLPYLTAVVEEGIRLHPGALVRQTRVAPEQALFYKDPS
TDQPWLIPPGSPVSMDARNCNLNPKFFDEPLKFKPERWIENPRLDRYNLSFSKGTRICLGLNLAYSE
LYMIIAGIFRRYDLYDGSGKQTTPTLALHDTYWERDAAVVSDCLVPFPQKGSKGIQVKVRAANEL*
>CYP65FC1|453|Grascr1
TQAFSTSASAADLIRQAIFYCVGKAIYNVYFHPLRHYPGPKLRAVTRLPYMYIQVKGTVLSSQKHLH
DKYGTVVRTAPNELSYIGASVWKTVYGHQLFPRNNMFYGAAPNGVRSMNSADKEQHARLRKAF
APGFSEKAIQAQRDIIERYINLLIEKLEEEAPKSSQGKPIDIVSYLNWTTFDIIGDLTFGEPFGCLETNQY
HFWVKNLFLGFKAISFLSVAQHLIPFKSLLYLFLPKSLMKKTIENFDLTVEKVDRRLSLDVDRPDFMS
NVRTLDGKDKLTKPEIYSTSSFIVVAGSETTASLLSAAIYYLHMNPEVKQKAYQELRQTYTSDKEINL
LSVAKLRYCSAVLDEALRLYPPVPEGLPRVVPTGGREICNRWVPEGTFVQVASYVACHSKVNFEAA
EEFIPERWLGDVRFASDQKEASQPFSIGPRNCLGQALAMAEMRLVLAKFLWKFDYEVCSESLDWLD
QRTYLLWEKHPLAIVLKAR
>CYP540A15|661|Grascr1
MTIKNFYLIGQDKAAFKHEIHIDQYHDFEALQLAVAGSYNIIDPNSIRFEDSKSSALQDLDEVLDCDE
DIGITINGQPIRDPAGPEGLPVIGSYYEVFPDHLGNHALGKNNYLTNDPAIAGIALQESAFFTKLITT
DHPLAGIKENRALFLGDTDTEAWRQAHKFLPPAMTPKAVRHYTPLMQACVKDSFKIFDTLDEQNE
SWNAYQYMLKLASQTVFKFALGYDAHHFESPDSHIDGLAVLIAQSLALNKKVTSRGPWYSKLGAIP
YTAANELKNTKAKLLAVLDQIIDNAPKGDTEDDLELNSAALGASCVVDYLKRATDDRGNKFPRDL
VIPNMLVIAGAGFTTTSALMSWLLYSLCVYEGYQDRLLQEIVDAGVTENTKWTPELSDGLTFLDKFI
KETQRVHNPAFQPGRTTKVDCIVPGGYKLPAGAVVVCAIHAIHKNPKIWSDPDHFNPDRWGTEET
KNRPKNSYIPFATGPRSCIGFNFALGEVKVLLPELVYRYEFSKEGEEAVTYDKAYELAKSF*

>CYP5077C5|684|Grascr1
MVILQRILDALYADRGAQAVATILVLLAYPAAVAIYNLFFHPLARFHGPKLWAASRLPFVYSLSTGK
LIQRQRRFHEKYGEIVRIAPDEISFASERAWDEIYAFRRGHKRALRDKTFATAPNEDVDPLITTTDPK
FHIRVRGLLSNSFTEDSLRSHLPDWQRILRGLINITDWINFFTMDVIGDLAFGEAFGCLAKARYYPT
WESFLQRLAPRSLTEDALEHQRYANQQINKRLDTKTNRPDFLTPFMKNNVNFENMSRAEILSTFNF
VIVGGAETSATVLTGLFSHLSRYIKIQQRLCSEIRSALKREEDISIDACKDLVYLDAVLNEGLRMFNPI
PSGLPRVVPEGGDTYCGAYLPGGTRIGVRTFAVNRSSEYFHDPDRFVPERWLPITKRPKTYHNEQLT
ASRPFSVGFHSCLGRPLAWVELRLVICRILWKFDIADDAHCRVDFDDSPVIMMVQKGPVNIRVQVR
EDHRKQ*
>CYP532V1|810|Grascr1
MLQILADVLNHARTVFSLQLTLLALPICLIILHHFQSPLYRFPGPFTASFTNLYRLIDVWRGSPHITLLS
LHRRYGDIIRLGTHVLSFSNPDALGEIYGLKANFPKSQFYYPQQILDPKGNHVGSLFSVIDSKEHARM
RKVVSGCFSLSALMRFESVIDSMTALFLEKTDEHFAQTGAECDLAQWLQFYAFDVICKITWGLEMG
TMRKNEDVQGVFKSVERRFAYMAVVGQIPSLDLLLLKNPLLRLLSKLNLIHANAPIVRFAQNRLSE
RLVRLKTESKSPAQPQPQFQSQFQSQPPNSLPKSEDPSQPNDLMSLFLHSRDLDPTFFDTNRVLAMC
MTMANAGSDTTAISIAAIFYYLLKSPACYRKLVAEIEGAVANGTLATHEIGTGSRLGLGLGPGLVSW
SESQKLPYLDACIKEAFRLHPAVGLPLERVVPEGGATILGEWIPGGTVVGMSAWVVHMSRRVFGED
VERYRPERWIEVETEEERKRMERGLFHFGMGSRSCVGKWISLLEVYKVVPAVVRRLEVSAVPWGKG
EVRGGGYGRFGGGAVGVSEWMC*
>CYP5519B1|814|Grascr1
MPSVFFRELLAVSGTSISLYAIAVALVVSLLLTIEAYCSRESLPGIPRLSRVPVLGALPVYMLHDIPRTL
HKLMTIGVDGISYAQIFNTVIVCIQNPMMAKEVLALPDEISSRLGNSGCISWSPFATLQRLIGNTLFSY
VGPTLSHRRSLYIMEFMHKKPLYEKFDTMIDIATIHMDTVAGKESTAEFKDLQRSADDFAISLWGEL
LYGNPNMHLNGVALTLSDKILEISTSPWSSLWHAFQRLLGTVTADEPTSSEAKVRADMANLVDEN
MAVLEAHEHHHPDTPPKLIRKISLKSGGEFSGPLSEFAYEFTHLNIFGGHHSIGSNITWSLIELNKYPK
CLAKLMREIDSVHPNDFAAISSKMPYLDAIILEINRLYSITVLLTMLDTLTPYRYPTVHSTVRVIDQEV
KLTASGAQSVVLKPGMLVYLSLLHINTSPKYWGDDAGTFVPERFLNKGDQDGRPFMSFGYGPRSC
VGYKFAILVVKVYLITLLKSYTVEVTDLYPEVETGALLKTKKSICARICRRV*
>CYP5094D1|1054|Grascr1
MPFNFGELLLLSTLACLIYVPCVILYRLTLHPLSNYPGPKLAAATSLYELLYDAILGRGGNLFYQKVD
EMHDLYGPIVRVAPNELHIKDSSFYTTLHANSPARRDKHHSTANATGAVQTTFATQEHDAHKRRR
IAISPVFTKGSINRSYNMIMQQLAILSENLRQLHSKGDVVNLHQILLAFTSDTVSHFIIGSSTELQTHPE
KARQWESALQSLFEMIPLTKTFPTLIDVLLKLPKSILLALSPKLAAVIQWREGFFAAAAAEQKFNYLD
SKPDSGSATEKTLFQAIQTSNLPPSDKTPERMGIEAESVATAGAETTVTALTHAIYFLLAHPSNLERL
RKELLPLYFESKKQEPSLASLQELPYLPRALSSAAVELYTNAQRQQTAVIKETLRISPIITVRLPLLAHT
ELQYGKWRIPAMTPVSTSILDVHNDPAIFPSPTEFQPDRWITADGKKSSEPDKFLVPFGRGHRMCLG
YELAYAEIYLGLSMIVRKFDLELFETEWEQDVKVKRDCFVGLPGKDSRGVRVKVTGLRE*
>CYP682BB1|2037|Grascr1
MDLTHGRLRDGSYTWEIEKMHKEFGPIIRINPLELHVKTVDLEFMSQLYPSVAKNVEKFPWFAELFG
NTEMAFTTIGHNLHRLRRSAFQNFFSKASVRRLEPLLRDLVDALCEQLLKRADTGQPVNLTYAFSA
LTQDVITEYCYSDRRNVLGMDDFAPLYYDMVQKPAILTAINILSFIPFRTLFPPGFSVVRARKWPYISI
GKNTCAETFALSWPNTCAETFALSWPNTPRPTPKTLPKKPHLPMHPLQPNPQMRLFPTSHPNPPH
PRLPRLPQKSLTPQSSTPSFPPPTYPHPSALLSSPDLPPPEKSLQRLAFEAQSLIGAGTLTSTHTLSTTFY
HLLSNPPILTRLNTELSTHMPNPSSPLPPLSTLEKLPYLSAVVHEGLRISYGILHRLQRVHPDNELHFE
KWTIPRGTAVGMSPYFVHTDPSLFPEPREFRPERWLGEEGEGRKKWIFNFGRGTRMCAGRELAMGE
IFLGIAAVGKRFAGEKEGGRGGARMRLFDTERERDVDVVRDYFITFPSRESKGVRVVIESSDERRV*

>CYP51157A1|2236|Grascr1
MFLTYSFLLPNTGHKTLDVLLFIAIAAFSVLFVTYGITSIRFRLEKTRYGADRKGREPPTLPYWIPYIGH
MLQMSDLHALYDYAASKSPDKSPVTLRIGSTTTYLIFGPDNVTALFKNSKHLERDNLTKRAFLNSN
QNASTNPQSSEHIVHQPSSPSSSPSTTSPPQSQPQTPPIDDPTAFRIGRKLHDLQHLHLSTPTPLPTNP

NKSHSHQIHPNLPSPTRRPHILSLTPSLSKIYWAYDEAFLLGLPELVYPIGNSLQDKLRDAVEKWISH
GMEAYDWDELEKEKENEVWEENFGCRLFRDSVREMKERGVATGDMAGALLSLVWATASNSIVLV
GWMVGWLVEDQELLKQVRRELEEGGVRRALISSRGLYADKRARISDDNEDEVFGKDLKTAHDGK
DGTFDLDISTLSFLPFLNSLYTESLRFHSSVTVTRRLTAPIEIAGYTLHKGNFVMTPSYLAHMDPNVW
GSRNNDAKSISKFSSASISTSTFTLASTPASTPTPTLTPTPSKISPTSTSTSIKSADLSTTLPSPSPRRFFAQR
FLPSPSHPTSALNPSTFLPYGGGSAICPGRFLSQQQILSAVATMVLMFEMEFVSYAEKIEEGDGGGTG
TSGEDMERRKWRTRNKRPEPDQRYAGGGVMPPDGEWVVGFKWRRGLGRD*
>CYP65FE1|2250|Grascr1
MSSEIQTHYLTAPGQTTDNIVTTSDSKFHSRARSLLSNSFTADAVHAMHPLIEGHCNLLISRIGELVL
QKPDQGALINMTDWMIFFTIDVIGDLALGKSFGCLENGDYHDWVRQLFQYVKMITVAAAPRYYST
WIVDFVFQRLIPQSVLDGQNAHKRYADEKINRRLDSKLNSNRSDFMTPFMKHNANYQTMSREEILS
TFAFLITGGSETTATALTGILNHLVRKPEVLARLTKEIRTYFVREKEIVLDVLVGENLPYLEATINEGL
RMCNPIPTGLPRQVPDGGDTYAGVFLPGGVRLRVQSYTVCRSAKYFHQPDAFHPERWLSKANGRP
QEFDNDRLSASKPFSVGYSACLGRAMALEEMRLVISRLLCRFDISEDPEERVEFDDWPMPSFVEKGP
MRIRVREKVSP*
>CYP548A31|2252|Grascr1
MTRKGINAKSRIIKTMKRYVQSAEESHDDASELMKSLIKECRRGGLNDQAIAGCLALNLWAPSSPQ
KYTANANEYRFIHWLAAYILHDPSLLATIRAETAPAFSTQPPNHKHIYESCPRFTAVYNETLRCTFG
GVSVRTVVSPLTLQNKTLRPGCTSMIAVRELRELHSNPDIFGANVDRFDAQRFLGNDLDKSPYFRPF
ARGSTYCLGRLLAKRVSMVFAATMLERFEDEIVGRDGGELGEKQLGAETMRRRMLKLPAVDWVTP
SVGIMKPAKGEEVYVRLSEREAS*
>CYP596W1|2395|Grascr1
MSLIAENDSAPSISQIVVACGAVLALYYVYRYFTVEQWHPDFPIVQLGEDGTGDDMADWLQNAKA
VIEKGMRMTDGPFQVTSAFGPMVLLPTRFAHELRNDPRVSLTKAISKAFFSHYPGFEAFVPADPNG
RVVRDVVQGKLTQSLGLITNDLVDEADLALSDRFPASKEWRTVEVKEDLLQVVARLSARVFLGFPF
CRNPSWLELSKSHTISTFIAASKLRRIAPYLRPFAARFWVPECKQIRQQVSQARTLLKAEIGLRRARA
EKALAAGQTTSKRNDSVGWMVEAAHKRGIKTDYAAGQLNMSIGAIHTTAEAMTRSLLDCCQHPE
VVEPLRQEMLGALKEHGWNRNMLSKLRLMDSFLKESQRSHPGQLVNMHRMVTEDITLSNGTILPK
GTHMDVLPGFDNPQVFREPQKFDAERFLKMRNEPNQDLSWQYVSLHASHMGFGYGEHACPGRF
FATNEVKIALFFLLLRYDWELENDEPALMQPEDGVSIRPDLKLRYKSRTPEVDLISLIDAKELEEMRS
N*
>CYP5042B7|2438|Grascr1
MNTALQIGIGVLILLLFCIRETLKPADQKWNFFTGRKSYKLPPGPKGIPLYGNLKQFLYNRHPQRLTP
YLTSLRTHGELTTLRLGSQTWILLNSKRVAAHFISKSGRLTNERPHMPIASGLVSNNKRTVIRQTASW
SEGRRVMHHLLSGSVLKTYGNWMEFESLSLLNSYLRQPERWYAHHFRYSTAVLYRLVMGEKLDKS
KEELDKYQQVTMEFVRSIGLSVVDFFPELDMWVPKWMQPWRRFWSEMGRRHRGVFVDWWTPIYE
MVKKGEGKAGFVRDTLLHPEVKYKGDEEEAMYLATSVIAAGGDNTRMSLNTFVMSMVSNPEILK
RGRAEIDRLCGADEGKLRLPGADDMEQLPYVSAIIKEVLRWRPTVPLVPPHQLTEDIEFEGYVFPKG
VSFVLNTISISQEYENWDDFNPERWMDGNEDNVTHDLWAFGGGRRICVGYRVAQQALFVAVARL
IFCFNITARGEIDNKNLNHVSLNEPFPVQVTVRSPAHETLILSGFDKAAKEESSQKMR*
>CYP573D1|2821|Grascr1
MLSKADPIALPDIYHRQVNKSPFYANGSFGDTDSVFDTQDWRKHADLRKMLAGPYSFSSIKNESVE
TGKAFDFANWATYLVFDVISEVAFGKALGFTEAGSDVHNLIKSWHDGLWAFSVLGRLYPMTQWIK
KTPLKRFIVANPAQPFGIGMLMRMRDEIIEQRVRDLEEGKLQNQEKVDLLQQYAFSFPSPYPWPCFL
PQPLKHQPKTPTNLPSPPSSILNSRSSTGASLPLAQIKAEVLLIIIAGADTTATILSALLNRLSTSPSLYTR
LQTELTSAHSLHLLSSPIPTYNEITTHLPLYCATIKETLRLSPAAPLVGSRMVSPEGLTLPGGWKVPGG
KGIEVACNPWIVNRDKGVWGEDAERFRPERWLEEKEKVKTYNRYLMTFGYGTRVCLGRELAMME
FVKSGVTFLGGFEVRWEREGKGEGDGAGKYKTRGGIAWFEGVNMRIGERGGRKRE*
>CYP677J2|3079|Grascr1
MEHTQRELHRKHGIGYLPVLHHLQAIADEVGHLVRIAPNEVACSDPEAIKTIYSTKNVFTKSDYYD
AWVPPTKRHVSHFAVRDEKLHAERRRIVNNVYSMSSVLESEGSIDSCTEMFCENMRDFAKRKSAID

LSLWTNMYAFDVVGELFFGSKFGFMQERTDIGGYMKSIDSLLPAFTIGGTVPSYLTKLCLVFTILFSSSI
RGALGAVKNIKTVSKVAVEKRRREVDKNTDDKHDMLRKMLQINAERGEKLDFTISDINVESQGAIF
AGADTTAIAMNSILYHLMRTPRAYKKLTRIVDDAVADGTLSMPAKYAEAIKIPYLRACINEGMRLH
PSVGLVMPRIVPAGGATISGFQFPEGYRVGVNPAVVQYDEDIFGEDADQFNPDRWIEGDIVSMEKA
MIQFGAGTRTCIGKNISLSEIYKLILQLVRDFRFRLVEPKRALRTHNYWFNKQTGIWVLVDERFQSSD
SHIEQESAS*
>CYP51168A1|3326|Grascr1
MSLTSAWAFRPEICALAVRPEICALAVCSSVLYIGALVLYRLFWHPLAAFPGPKIAAVSDLYGIYHDL
YRSGQLPKQMPKLHDIYGTAFYKDWSFYNSRAFEHSTLNILDTPSARTRKDAYIRLFSKESISKLEPRI
NAHIDRFINVLYEAGTKRQPVDLSQGFKCLTADIIFDFMFQEDFGGLSAKNFHHPLIAPTDDIVIFAE
WGFYFRAIFHPVEILVDYIPDSILRVLFPPALGIKTLINLCRETIQNLKRKPLTDPKMTQTVFGALLNP
TSSKTSGITDEAVLTSESTFMLLAGVDTTANTLVTAVYAMLRNPEMHQKLMNELTAAFPGYSSTDS
LTFERLQSLPYLEACIKEALRLSYGAPGKLPRVVPSTGHKAAGLYIPPGSIVAHSSYTYQTDETVFPSP
TEFKPERWLTGNATELDRFLIPFSKGNRSCVGIHLARTELFITLAKLMLSVNMKVWKTTPRDMEWM
YYSVTVHRGHLKVIVEISHDEIAVYTDNE*
>CYP5078J2|3380|Grascr1
MAKANFSIPYMGSVPFSHLSSSSGGANFMLDIPYVPTSVLARVAICLPVLFVAYGIYLRYFLGVSHIPG
PFIATFSNYWKIQAAWKEEMPQQNIELHKKYGPLVRVGPNMISVDDPAALGSIYGFKPIYQKSAFYP
IVEAIYKGRFLANLFTTTDEKYHARLKRASVNAYSMTTLNELEPHVQDVIQLFLRRIDEVTDNGQKP
FDIGNWVQYFAFDALGEVNFSSQLGFLETGTDVGNSIATIDGLLRYLSIIGQAPWIHKFLLGNRFIHK
LLPFLETSNEVQNFAIKMINKRVSGEQSDSRRDMLARLLEASEKDDARLTREEIIALTTTNLIAGSDST
AIGLRAILYFLCKHPAVYSKLQREVDDAFAAGSLSQPVRYADGAKLEYLNAVVTEALRAHAATGF
VLERHVPEGGITIAGTHIPAGTIVGINSWVMHANKKVYGEDAEQFNPERWLNASEAQLREMRRCN
MSFGAGPRICIGRNISMMEIIKLIPELMRKYDFRLVNPDKEWEILGHWFVKQEGIDMYFNKRNLGEK
TVRASA*
>CYP6980B1|3505|Grascr1
MGAKVYILTSSQDISAAYRNITTLSFDDAVNETMRSFGFSEDGLEKLWRRDYAGVYPNPSKKHFAGI
SHDIYKSQLHPGDKLNHLTHRLLSVMRKNLLWENLPSAAILQERASSKNMSLMALCYGGLLDAA
NTAIFGSRLNEISRDVPKHFRVFDGKVWQLLFQLPAPFAREMISAKMEIVKSFEQYVESPLETKADR
AWFIEALETEMAALNMSVKDQATMFFMLYWLFNVNAYKTCFWLLSYILTTPGLLSSIRAETAPAIA
ADGGVNVTRLVNACPYLTASYDETLRLALDVASARNVMEDTMLNGRLYRAGSKIMMPYRQLHFE
EAVFGSNPEAFDPSRFLKNKDLNRNPSFKPFGGGSTMCSGRFLAKYEVLSFVALSINRLELKVADGE
GEAQQRFPRYNRSKPGLGIMEPVDGDDLIVEVRRRRT*
>CYP53A65|3583|Grascr1
MALHDILFSPWTLLLLPILFYLLPYLRNRTLIPFPAPPLAAFSNLWLLLQSRRGQRFLSVDRAHKKYG
TFVRIQPDHISIANPDAIPIVYGHGTGFLKAHYYDAFVSIQRGLFNTRDRAEHTRKRKIVSHTFSARSI
GQFEQYIHSNLSDLLKQWNTLSDTATKAGKAYADIDSLHWFNYLAFDIIGDLAFGEPFGMVNVGA
DIAEVRETPTSPPRYAPAVQVLNRRGEVSNTLGSLPGLKPYAKYLPDPFFSQGLQAVSDLAGIAIARV
KARLEQERSGIKSERRDLLARLMEGKDERGEPMGQAELTAEALTQLIAGSDTTSNTSCAFFFWATKT
PGVMENLRKEVDAVLPEGGIPSFELVKDLPYLHRAIDEALRIHSTSSLGLPRVVPPGEGVEVCGKWF
PQGTVLSVPSYTIHHDRGIWGEDVMEFVPDRWLEGRLTERQKGAFIPFSYGPRACVGRKSILMSADE
FEKKILTAVVGNVAEMELALIVSTVVLNFDLELRQDTLETREGFLRKPLGCAMGLRKRVK*
>CYP654D1|3989|Grascr1
MTAVRDEIMSVFKVATNLSSTPDALWAAESDLSLYLTLKHIFGYLALVATVSWVLWQPLRTDPKDY
LIPGVYVVGGSKKHEVKATSARFRAESPELLLDGYRRTKGQEPFYVLSARGPRLIIPTRYMEELKSAP
MDQVDFTGIVHEMLEYKYTGLVDRSRMIPSTLSRMIPSTLNSHLTPRLPNIMPEVQDEINLAIDHEFP
QNDDCCVPLGTEVDVMPGITRMFAKATSRMVGGKAMSQNEEWIPVAINYTLWSFNAAQKVKSTP
WPIRRMITPWFEKVRKKIPWCFATAVKVGVPILEQREQTGEIANDFLQFLTETAKGVQTIGGILYDL
CQYPELIKVLREEYEGIVDRDGNIPKGGYAKLVKMDSVMKESQRHNPIAILTFERIINADRTLKDGFT
IPAGSYIGVASYNIAMDPEIYPEPEKFDGLRFERLRNDPAWTNKASFVSSNAQSLAFGYGRHACPGR
QFMEQEIKSFLVKFLTNYDIKFKDGQTRVKSTPLEVQLVLRHTPILLKRRNL*

>CYP59X2|4262|Grascr1
MLIGSSDETVQQFTQEHSLPKYPALKSFLQPIADGQDIVTMEGATWKKWRAIFSPSFSMSHLATLAT
AMVEETLKFCQTLDLRAQNKQAFQMKPLTDFLTLDIIGRVVLDVQLNSQVGSNPLTDGLRSQVPW
LIFGGDANPLAKLNPPRPLVLWYNAWRMDRYIAWILDRHLADSRTETSRKTIIDLAATAYLSANKD
NDISTGIDTTFKSFASSQVKLFLFSGHDTTTSSVCYLFYVLANNPSVRNRMRAEQDKIFGPDIKSATSV
IQSQPALLNDLPYTTAVVKETLRLYPAVSSTRAGEPNFNITDQHGRLYPTDGFLIWANHHIIHRDPA
YWPEPDEFLAERWLVTPSDDPLYPVKGVWRPFERGPRNCIGQELVMMEMKIILVMTARRYNIEAA
YEEIDGGALGSKWNTVHGERGYQIQRSQPSGDLPCRAIIETR*
>CYP684N1|4399|Grascr1
MELQSNPSQHLLLLYAVLGLGLGFLFVRLAIRAYAGPAASVPGPWYTRWTNLPLKYYSLRARRTQ
WVHSLHQKYGPVVRLKPSEVSISDVRAVREIHRISTPFLKTDYYMRLTGKGVLPGLFTFSDPKEHSAR
RRLLAGPLSESSLKNVEPTVNKNIDKAIEGIRMEMKENGTADIFKWFTFMATDVIGELSFAESFRML
DQGKPNQYSHDLHAVALATALNSELGILTHFLGALGLSPFRQAYEATGRFRSYSTESFKRYEQHVA
LNPTNPKPTLFTKFIQKVEGGEESLPRTALIREGQGYIVAGSDTTAFTTTYLVYAVTRNREVMEKLVA
ELEGLPEGFGWEDVRHLPYLNKCIDEALRLYGAAPSGLPRWVPKGGAHLAGYTFPEKFVVSTQGYT
LHRDPGIWPEPERFNPDRWDAPTQQMQQAFMPFGGGTRICIGLHLARDEMRMAVARFFRMFPHA
RVTSSDDDMEFRAYFLATLKRKRCMVAAS*
>CYP5589C2|4661|Grascr1
MTSYAADSSNNHHFLPCLVALFLTCFFALQTRSTGIYTKFQRKRRFTIFNPFTVKPAHEVVATGYNE
VIKRYGAPFTMQWWAKDWIILPPKYLPGIRDADPSHLNFLNTISDCFYLHKTVGRLYAGNRMAHV
VKRGLSPKLPLLTPKLLEEMDYAFAIELGDPGHGKSFVAQRLLSRIAHRVASRIMVGHELCRNEMFI
NASLEFVMSLFGTALVICKLPLGPFREVLAGPITLWHASKLNKVEKLVVPVVRDRITDWQAGYGQS
EQLDCIQWTLDLMPTEGATDTKVISQELIHNLWAGTSAPGGLVMDMLFQVLLEPHYLVPLRAEAA
DAVHMFGWSEHTFNHLTLQDSFIREISRLYPTGSITVTRTVYDRPFVFSDGLTLPVATRLAFPAAPIL
HDESTFPNAKQFDGFRFARQHTKDNDRKVGEKQNSSSSMSTTNLAFGYGTHVCPGRFFAVRMVK
MIFTKLILDYDITWEGHVTQRPPPVCIEGQFIPNLKQKIIIKKRV*
>CYP613U1|4665|Grascr1
MDRKVINANTLSLQNWSFVFSAFASACGLLVILPKLRKHVLSRINRCLSFLINQYLLFKYPICHNETG
RSIPTCPYVFPNGQGNVAKFLEGLSNAGEWSRQYGSVYRIWSGTKPEIVLSRPEHVKAVFYDSDRHT
KAKDNNSGYLMHQILGQCLGLISGKSWANLKEVVQTPFTYSSTTNHVQLVQRMVAEYIQELFDSK
TADELHPANDLKMLPFWITAQIIYGDLTPAMKTELQQLATARESLFRHVIRGGISRFAISRFLPTQTN
RDLSSFLRRWQAFNDKAAAAAETATKTTTTTSPTSTSKPPPPPPITQMYRSLHNGTSTRPQLLQTLDE
ILFANLDLAPFTLESSRLHPIAAFSVPQSAPTSRLLDGFTIPAGTDFIVDATTLNTNKEFWGEDAGEFR
PQRFLEGGGEVKEGKEGKREMRYNYWRFGFGPRVCLGRYLADLILRAVVAELVGGYEIEVPREWQ
EQGGKGKEKRVDGVWISQADVRLKWWKRGEGGEGG*
>CYP65FV1|5047|Grascr1
MMSWNGIQAGMRKALYYVYFHPLAEFPGPLLAAASAPVTIATFQGRSHQWAAQQHRRYGANCF
HTHSQTKRYGSRNPFYNSYAHLLVKQLREESKDKAEDVDMVEWLTYATFDIISDLSLGQSFGDLER
KQAHPWVNTFKSGIKQRVLFANARAIGIPFFDSTIARWVSPIIQRKRHEQFEYARAVVGTRLQDKSR
RTDFVSNFIRENVMGSVSREEIDSIFIIVTLAGCESPATALSGALLHLLSNPTSYKKLQDEVRGLPTDK
DLTITNISQLPYLRAVLDESMRIYPPVPAAMDRVVTKGGRTISGYAVPEGTWVGIPQHATYHSERHF
TKPNEFLPERFLEKHARIFEGDNRAIVQPFSTGPRNCIGQNLARAEVRLLLAKLVWHFDMRLADEA
EDWSGKQKVYGLWDKIPLIVKLEAR*
>CYP619N2|5107|Grascr1
MEALILILTALLVLLVVQWPYPWLAYRDKLLPPGPSTLPFLGNLHQIPRTGLHLKYTRWARSYGSML
SLKIGQGTMIVLSSALDAGRLLDKRSFHYSDRPSSHVIGDLVFGGHHPMFMNANERWKLRRKLYH
QMLQEPRCNREHIALVEAESSQLICDICLLPDDLMLHPGRYSNSIIMSLVFGARTPRHDTPHYLKLR
KIMTELSAIGEIGATPPVDLLPALKYLPEQLWGNWRTRVRDLRETIFDLYRPLVDSVIERRKMGRRV
NSFLDGVLDQQDDMQLSRDEIDIMCGNLLEGGTDTMATLILTLCQAMALNPLILEEAHTHIDSVVD
DSRLPTWADYERLPYIAMIVKELLRWRPPAPTGFPHTLDKDDEIDGMKLPKGSIVVLNIWGIHNDA

DRYPKPEVFDPRRFQNQTALASVYANSSDYQRRDHFGYGIGRRICPGVHLAERALWLATARILWAF
NIQPKSDANGKQVPINVAPATGYRDGFLNQCMPFEVEIEARSAKHREVMLAEVTRSEAEILTVYA*
>CYP5105E4|5167|Grascr1
MAFEALLEICFVSILVYSASLVLYRLILSPLAKIPGPKLAGLTSWYEIYYDIVQPGQYVWKIKEMHAKR
NLRWRFSKSEIHIADPAYLDEIYAGASRNREKYEFQLRTLPVPMSMGAARTHDLHRKRREALNPFFS
KKSVVDLQPMVQTKVGKLAEVFEKHRKSNTVVNLSSVYYAVVMQYSFGQGDNLLDDEVKAATLR
KNITDLLLGVKVSQHFPWIFTFLNALPFFIAKHIMPPGALDMLAFSEKIRRDINRVLEAKTSVEKDRK
RSIFYELRDNSSLPPSEKSPKRLEDDATLLVMAAFTHAAKGTESVAVTIATAHFYLLYLPDYMAKVR
AELRTVPVNASWAELEKLPYLTGVLLESLRISLRIDMSVTGRLARLAPEETLQYDTYKIPPNTPVSSTT
LCIHTDENTFPDPWTFKPERWFGREGTERKRYLMSFNKGARQCIGINLAYAEMYLALAAAVRFEM
ELYQTDESDVRFKHDFHAAFPKLDSKGIRAFVR*
>CYP51165A1|5300|Grascr1
MFRQIDVKWIAQLLVAFGASLYCAQLPFIYRFILRCSLDFQPFTLFLLSCTCAVLSLLLQVISLLFEHPG
PKRLPGPKGLPWIGTALQISLNRIDEWHQKYGPIFETVILGQRYVTIGSESIAVELLKNRGSIYSDRHY
NAVLRGQPHLPMLRMGESWRRQRKLFYQFTQACTINAPTTGSIYAKVVTREVEKLVRNLVGHPED
WQKIIDGYVSSISLALTYGKEIRSYNDHNVRRIVEHYRNFENASRPDYYLVNLFPILRHLPAWLAPFK
NSGQEYLQAERAYLFIALAQVQHKLDAHIGTNDRGQKKPEIDSASSNWCLAQENWQNEERWDA
QGLTLEEAAYTLGSFATAALASSPAVLKTFFLAMLHHPEWQQRLQDEVDRVYRDAAIHRKESTEQ
LWTKKETFPVARAIVKEVLRWRPTLPTGFPHILTQDDVYDGYTIKKGTMVLWSQWHMAHDPSLYA
DPHTFNPARHLPCSTSLPLSLANDSSFGFGRRACPGWQQAEDMLLAAVISIARSLELNRERDEKGEP
VGKLPWVEYEGGSTTRPRWFKVEIRRRGREDKET*
>CYP59U4|5662|Grascr1
MEALAGSIQFNKGFSSETLSSLIPGMVEATMTYCDVLSELSQNAKLFQLDIVTLRFTLDFIGQTVMNA
SLGQEEGNVLADSMLSQISWLAPNKEINPLEMINVPRFLVLWWNGRKMDRYIGQELDKRFLEYRQ
GGSSARGSVIDIVLRAYMNENTEGNTESTISEENIDPLFRTFAITQIRSFLFLGHDSMSSTICYAIYLLSK
NTAALESIRSEHSRIFGANPDPSVVADMIRASPYLLEGLQYTTAVIKETLRLFPPGSGIRQGFAGADLI
GEDGTHYPTENTLIWILHTAMHNEETYWVNASSFMPERWLVDPGLELYPRKGAWRPFEWGPRACI
GQNLAMVELKIALAMVVRAFDFNIAYDEWDAMHGRQGGEKLTYRGERAYQVEIGASHPADLMP
CRVSMAKIK*
>CYP617Z1|5774|Grascr1
MAPPANVVILAPAVAYLLFCAYQRETVAPSNSLTLDVFVVLLYAAVSLGLWTVVIRPRFFSPLRHLP
GPKGGSFWHGQLWRVLKEPAGTPQREWMATIPNKGLIRYLSLFNQEKVLLTVPSTLSEVLVSNTYD
FSKPAHIREDMYQRKHLQPAFKHRQIKDLYPIFMTHACRMVEKVDTEISAKARNSEVDEEGYTLVP
LRAKIHGTLMDTIGKAGADAQLGALDDRDAEINRLYGKVLSGGAVRYLLTVLSTLLPASFCYSVPL
KQNKALNDAGKEFRVLARGVVSRRKTRKIKDPDNDPNDILGVIIDGQAFTDEGLEDQVMNMIAA
GLDVTSVAVSWGLLTLCQHPEIATRLREEIRQHLPSPDKADGMKAITSDVVDGLPYLNAFCNEVNR
FFPGVPTTTRVAIRDTVISGQVIPKGTTLVISPAAINKDERFWGPDASHFNPERFMKPGQANSGGAN
NHFANLSFLQGSHSCIGEKFARSELACLMAVVVGRFEMKLGPSGKLPKARPGVNPIPEGELELRLRA
VPGW*
>CYP6481B1|6125|Grascr1
MYPSTKQIRRAHFGNTGEPFIIETAGQKMYILTSADDAAAMYKNTADFSLDEIIKIVHRGIANVSPRG
FELLWPKRDDGAVKTIATNKDGFTYISTRLLHKQLFFGAPLENITERFLNNIESSTQWDAFFPKSVLA
QDPKNDERVVSIHQWTLEVLVNAVTRSFFGDALPNLLPDISTIFDQWDMNSYMTNYQYPAFLSQA
AIVPREKIIRALTQYFDLPHEERNDAIPFIRELEEEQHRAGLSNEDAARLIMIVHWGTIQQEITAAITP
KQSPTPASTSSTTNLNRSTTPVSISHTHLTNSCPHLNSLFDETIRITSTTTHIRCAHNPVHIHNKLIPAS
ARVIVPVRQLMLDPSGFGPDAAELDPERFLRTKNLERSPYYRPFGGGEYLCSGRHLGRRAVLGFVAI
VLWRYEVGVVKEGESVMGVRGKKFPRLDLGKPSLGISMQVEGDDMVVVVRPRVGMGEGEEGREE
RGGEGRGGGEGGGGEGVSDGVGDISEHVRGSI*
>CYP5603B1|6346|Grascr1
MPFLDRKPYANLFSLQDSYHHAALKRSISPLYTKTQVQNYESRIDSCTMLFAAKLGDICGEGSKQLD
MAFWLHAYAFDSLGEINLSKKLGFLDTGTDVGGMMAAGEKILHMTGLVNSLPDTWIPEHELTTNS

MVNRLGCKSCSASCEICEGEKGIPFWRSCFDTVAYLDQFAASELSQRVEKPQDAHDLLSGFLELRES
EPTRISIAEIIGALYINLPDTESGTGRAEGNPNEKKVQRYSRLLLNQSSRQAGHDVLAVTLRTILYYLA
RSPATRDKLRLEIDDAAARLTYSEPIPYAVIARLPYLEAVIHEALRAHPNTGLILERVVSRGGATIDGY
DIPGDTIVGVNAWVLHRDREIFGEDVDVFRAERWLEADKKMLQGMKQNLFSFGAGPRMCIGRNI
AMMQTGKLLAEFYRRFDARLAHPEREWTVNGSWVCSQSDMDMIVSLRKGSATT*
>CYP531M1|6385|Grascr1
MTLPNDVLVNGQRVSNLFSTRDEDWHNSSLKPIRSLYSMTRALDVEVHVDKMLEDFMSIVEERFVK
NGKELDAAEYIPFFAWDSMCNMTFGKTYGLLETGQDLNNVISTSTRGLEYFAPVSQMPSLDLLLAK
NPVKSVGPPPFIWATIESYKAVAERQLEPTQIAFSRDSDFLDKFLVAKKEHPEIVTDDMVVNYLLSN
VLAGSDSTAIYACAALYYILRTPGVLEKVRKDLDPQKVKVPVSWKTAKQLPYFDAVMKEAARIHP
GIGLMLERLVPEGGLTLPDGRFIPEGTIVGMNPWVVNRSEKTFGKDTDKFIPERWLQQPNESHEAFE
ARKIMMKAGDFTFGGGSRICLGKSLAILQAYKLIATLFTKYEVSLARPDEEWQIRNCWFVYQYNIPV
KLTPRTVV*
>CYP65FL1|6555|Grascr1
MKERLYFNYLFPLLPGAVIVGAAVFVMVCLAKWIRNVFFHPLSKFPGPCLAGATSIPLLFHRSRGRL
PHWLAKLHAQYGPTVRYFPDGLSYIDHNAWNDIYGPRRKGGGLPKNPAIALPMFGGTDSMIFMD
DENHNRIRRLLSHAMSERALREQHEVILGYVNVLIDRVKATAQGLDAGEIDLVRWYNFASFDIIAD
LSFGEPMGMLQLGEWTPWIKAINDSMPFATLVQVASRCLPFLGPVLNLSLARMREALVLNLSLAR
MREALKTHIAYSRTQVNKRLARKTDRTDFWKYILREQRSQGTAIGVSAAENHALGMTLMVAGSET
TATALSGLTDCLLNNPDKHEKLVAEIRNTFNSPAEMNEESLARLPYLGACIEETLRLHPPAPVGLER
WVPAGGHRICGEFVPAGTHLLIGFTHWVAYRSPALWHRPEDYCPERWLPESKITGPFTNDVRQVL
QPFAYGPRNCIGKNLAYNEMRLIAAQMLWHFDLELSGKSRNWADQKIYGLAVKGPLIVKPKLAK
GVEKTL*
>CYP51196B1|7072|Grascr1
MQLFPVLTALAVITVSSYFFRLGNSYRQARKAGLPIFITFVHPHDIWWIILRIPVTQLLKKILPHSLYLR
FELTTYGFEMRTRTIPYEKLGQSYMLVGPGAKELWVGDPDMARAAAANKDIQQSKMAHSTHSLDI
CLETILTDVGFTELFGPNILTAQGETWSRQRKIIAPILNESISESVWIEGAYQATDMLDSLTESSTNGHS
KQLVKQDSAESIEGLRRIAINVLGAVGYGRPRSWKSKDEVPPGHKLSYMESRSLFSIISHLALAVFIPP
SILCLPIFRPDIQKVGIAKREYPEHNKEMVAHERKTADITKNNLMSVIVRLSDQEKNAMSKSKLYLSE
DELSGNLFIFTLAAFDTTSNTMAYAFTQLALHPELQDWIIEEIDQVLPNTKEIPPYEEAFPRLKRVLAL
MFETLRLYTPVPHLARETKVPLTLPSSSGTRHSIPADTNIYISPALTHVCPLYWGSDSTTYRPSRWIVTS
DDGTTSLFEPPKGTFAPWSLAPRTCPGQKMSQVEFVTVFANLFRRARVEPVLMGEETLEDARARLM
KVVEDSQPLITLQMNRPRDFIVKWKRRE*

>CYP526B4|7091|Grascr1
MSSVVFSEVPGRLVASLDTTIIAIALTATLAILLYGLYQWLLPKPVPRIAYNPGAIRSLLGDAPDMIKE
VSVTGEFRVWCARQVIKMDSPICQIFIKPFSQPWILLADFRESKDILMRRKEFDKSSFLSDGMACMG
AFHGIYKTGDKFKSNRQLIQDLMTSTFLNSHVGTAIYQKGLEVMKLFELKMKLANGRPFSAKKDFE
YASLDVMCDFAFGSNWKQTATGPQVDLLKKIKPSEVKAGDSDEPIEFPLVPLAEFLESVYEAPELVE
RTINAVMPKLQSLSCKRGGGPSSPGHVETGLEHMLMREQARAEKLGRDPDFDSAMFGDIIGGHHT
TSGAMMWMAKYLTDLPHVQDRLRSVLHEALSGARDENRLFTFEEIRRVKLPYLDAVIEEMLRINA
VTVTREALCDTTILGCPIKKGTQVFFVSNGPGFLSPSLPVDDSKRSENSRVAKLNGTWDETKDLTIFD
PERWLVRKRDGSGLSEHDVDFDGAAGPQLVFGLGPRACWGRRLAHMEIRTIISMLVWNFELLRTP
PVVSSHAGLEGIARVPQKCYLRLRKV*
>CYP6577A2|7424|Grascr1
MAETMFNNSSHWDPVWTLSLSPFLGVAFLLLLEKWFLSPPSTLPWIGQKAGFFPRIAACLREKNEGI
SDLKAGYLKFGSKGKFFIKPDMVFKPGIMVPREHIRWILDQPPDVLSVHEARYDKFALDIVNPGHD
EAVDGVFLDVIHRKLTRNLLKLQGDLAEEVSRNMDAVLGTDTENWVESNVWSTVENTVSSAVMR
ILLGRSVCREASLHEDIRRYTRAFGYSSIFVGRILPRFLKPFLGSILSGLTLHRQRNLLRKWFNPMVEER
FTNLLKKNQDPSYDYTPPEDLVTWAADALLCTGNAERCPPSGLSRRLAIMIPAALPSIKSTATNAIF
DILSAPPEMNLQDHLYNEVASAFDTTPEKGWADQSLLPRLVFLNSATRETMRVNPIAVVVAERKIL

PKEGVTLPSGQHLPQGTWLGVPTVGVQSDENNYTNAARYEPFRFCNRKPGEEADSKDASGPVIST
GMVNVTDTYLPFGAGKFACPGRWLAAHVIKLTIAYLLYNYEIKPYAVRPLNSAIFGTNLPDRQATM
MVRRRKH*
>CYP51160A1|7425|Grascr1
MNSVKLVTEVLHSDAKSYELHMNIYVYILSVVVIFLRAPSLVKSVRPSLSLSKKQRDRKNEPSKFSPV
PLWMDSTLGMVERPRCFFDFLLSRSSSGEASFLQPGSLVRDSLAHTDEVVEVFQRSKKLTTREYSAQ
VLQRSNQALNNRSASQTRFSSQPKSSEISRADQVWEDFQEVSDTNLVAKDSVALIAQHFVGGLVAQ
FDQFPRGKWSLIGVPKLFQQHMLEASIRTLFGPSLTKDSPLFSECLWKYHKAITACLTPSSDFCHKTF
WETRKECLEAVKAHLRKFWSEFDMQEVDATHGATLGSRLLREREQALTKANLSFDERAQLELELL
VSIESKTIPLATYMITEILQRPDLHGRICTELKKAAKTASKRGTLAFDHAKLEQLPLLLSVYQECLRFH
SSHHEAWQLEQEIKLKNSALKAGTVFLCPSYLAKQYDGTWDSAEYQSDVFWPERFLEEKPYHFGM
HFPYGGGSAISSDHHYAKQEILSAVAVILAKFDFQVVSFASEPLEETKEEMERKICAEARDERGLAVR
LRRR*
>CYP51203B1|7426|Grascr1
MFNSIVAYSLVLLGLSICYILYLEYFPHRLPNSLPSVGFRKEILSRSRASLRQILGTSRTLAEGYEQYGAE
NCSYVVPDTTFQPQVMLPQVHVKWLSTQPESVLSSQAVRMVRNGVNYLPTNLDPKSSLLFIDKIIG
QSLSQSLDLIQPDMFDEIRHTVDIKLGTDTDSWHDIDLSDAMSNIIDRTANRILFGLSLCRNEAYLRIL
RCFIICMGASTLLIGQLPPWILRPVAGVLLSIPTFVTKKMSIAYVQPLVKERMQEVKPDKKDSGHSKT
SHDFVTQSVKSVKKFKISVTGDVTTFLAEQFLILAFAGMATTGAAATNIFLDIFSANPRINLWENLRL
EAASIFKSEDNWTSPALMKDMIKTDSTIRESLRKNTLQSRGLLKQVMPKSGITLPDGTHVPQGTWL
GVPVQAMQRDQRIYPNPHEYDPFRFAQLRAAGEVEGKDDPSTSKNLDAAQPSDVYVSFSYGRNSC
PGRWFATRLLKLMIAYISIHYDIKPLAHRPRNFSFGDASIPSFTTRVMVRRRQ*
>CYP59AB1|7427|Grascr1
MFFYRELLQAIPSIMSKFNVNIVRMVDMSELCLVAKAVTIAITIIFLYRLYRARSYMRRLQREGMPMP
PHHWLFGHILVVARVFIALPPHAHLLLLADQVRRAYPHLDTAFYLDLWPFGPPSLWAVSPELASQF
TQENSLPKYEGVRRFLKPLTGKKDLVTMEGHDWKHWRAKFNPGFSANNITSMIPVMVEEVRTFQE
IIRKHAITGDSFLLEKATLAMSMDIIGRVVLDHELNSQKSYNPLTSGLIDQLAWCDAGMHSNPLEYI
NIRRYETANLRPKSKYPADLIMSAYLKETSSDGRPTKMDPVFSEFMRAQVKLFLEAGHDTTAASIVF
TLYMLQKHPERLIRLREELDTVFGPDITATCDFLKKNPHLLHHCSFLLAVTKETLRLFPPTATARFGV
PDFYLVNTDGKRLPTENCLVVANHYGIHHNPRYWPRVEEFLPERWLVDETHPLYPVKNGWRPFER
GARNCLGQELAMTEIKLVVAIIVREFNVHDAYTENDAKKGQTKKNLRVNGERAYQINRGGGHPSE
NFPCRVSLVAGKEAN*
>CYP51166A1|7476|Grascr1
MNDTPVYVESGQPHTYNIHPYTENAWPYISSIYPYFDSLPGLTITWTTVLGCFFAYTVVHICGLLLRR
LVFSPLASIPGPKAAAASRLYELWFQGIHHLKFPDEIKRLHSKYGPIVRISPHEVSINDSRFNIEFFNSG
QLDKDPKYYAFGFDDAVFVLSDNKKWAERQTVMANHFRGKVWQEFFPQLRRELDSLCAKLGKV
AENKTDFNISRGFRKTSNDIMRTFLLGDEYSGINANSRDFGPDAEPVFHPLFRMVAFTRVFKWMFT
LHEYMPDIVLETLLPMAKYKREIDRMVRSLVGNYQPEEEKLIQDKALFYRMIDSDPSYLERSGVAAV
EEFLELLWGGREVLGHASSNIVFHLVKNPQVMEKLHTELLKVPFDLHSAPYSQLKTIPYLWAVCKE
GLRVQLGGNFRIPRVTRKDIYYGDHLLPAGTSVSMCPKFFHDDAEIYLDPDQFIPERWIEADAAKLE
EMERFWNPFGNGARTCGGRPMAYEVVFRIIARIFVQYRLDWSSNCDADSCKNERILEAFPPTSSTG
MRVTAHEWDD*
>CYP6466B1|7621|Grascr1
MPAGKIYIVNSLELIAQVQRQPKVLSFWYLEASFGAKLAAVSKSASRKLLHNVHRDEGEDSLFDDSI
KMLSKALRPRDNLDEMNQVMARTIIESLARFESHLDPEGKCNVDLWDWVKHEMTVATTDATYGP
LNPYRDPTIESGFWHLSENVSLLILGIFPSLLARKGYHGRETVIQAFTKYETAGGPSQASMMTKEKYQ
ISKSHGISPLDRARFETESGMAILSNTIPAAMWAFFHVFSRPEALSLVRERALTLTTLTQRKNQNGNQ
ITVRIIPLLKLREDPILSSIIHETLRLQAYGAPSRMVMRDTMLNGYLLKQGSVLMMPNEAIHTDASISE
KPLEFDARLFSPPSEGKEDGEGEKEVREELPKGAFRNFGGGANWCPGRFFAINEILFMLVLVALKYE
IGPVAGEWCMPRLDVSEYVFYCDAATGEVPCFCKDEEGVGERDVGV*
>CYP6846B1|7702|Grascr1

MYNSLVSEAFEFWLYDVPISYLNVFLVAGLSLLFWRLYRFTILPILHPKDPKELPYWIPFLGHAIGFFV
NSYRVLDQGRIYFGNTREPYSLTVAGQKILIVTSPEDVQSLYKHTESFSWLTFVQDLYRWVGLSQASI
DKLWSPPTKEQKMNNPRRKLPPNQMIEEYEHHQLLPGKNHDDIASKFAVELDKLVSWRTLRSRSC
YKLESTPDFVKISLIDWTSEVFILTITQLYWGKCIFETDPNLLQTFRQWEETNWKYVFQLPRVLSKDTY
AARDGIVNAFTKYFQSTKESRADISYFVEISESELRDIALEDSDIARVHMLQMWAVNGNVHKVTFWI
FAYLLHYPELLSVVSEEVKAGMLNGKPNVTASPTFPTCWITVLASRRFTSNVVKETVVGGKIFRKRN
NVMVPYRQLHLNTEFWGPDAAEFKAERFLHDKNLAKSSNYRPFGGGQHLCPGRFLAKKVVFSFIA
LALSRFEISLDTKDELGKPRFPRADERKPALGTLAPVAGDEVILKIRPKRTL*
>CYP561Q1|7800|Grascr1
MALPPLCSTVEWGLFAIFIYRIAIIIHRLFLSPLCKIPGPKLYALSNLPVFYRKNIQGVWARKVYFLHER
YGPVVRIGPDHVSVDGGIGWKDVYFAKPEFPKHLDGLDEYYKLSIISAPTTEHRRLRRLYAPAFTDK
AIRAQEPILQKFVSLLLQRLGEQEGKGVDINTWFNYTSFDIMGELTFGEPFHCLDRSELHGWIALIFE
NLKAVVSLRAFRSYALPRELLPFLMTKEQLAKKEGHERYTQEKVLQRISEGRDARQDLMMHFMKH
MEKGELTEDEVRVNAGVFIVAGSETTAVTLSTLVYHICKDNEIYNGLTREIRGTFAKEDDITISSTMN
LEYFNMCLNEALRIHAPAPDLASRTSPGAFVGGHWLPDGTEITLPTYSTFRSPLHFHNPTVFVPTRF
APSPSSPYFDQAYEHDNLSVFEPFGAGARDCLGKHLAWAQMRLIAARLLWRYDVQVDWEASEG
WLERQRGLGGE*
>CYP61A1|8069|Grascr1
MASVAGGSFASPSADAQTAFFTSNELLVSRFITGLSAWNVALTILLGLIAYDQFSYIWQKGSIVGPSM
KMPFIGPFLESVFPDFRKYKAKWDSGALSCVSVFHKFVVIASTRDMARKVFNSPNFVKPCVVDVAH
KLLRPNNWVFLDGKAHVDYRKGLNGLFTRQALEMYLPMQEEVYDDYFERFIKISQEQNNGKPVP
WMPVFRELMCALALRTFVGYYMSESAVKKIADDYYNVTAALELVNFPIIIPFTKTWYGKKASDMIL
EEFSKCAAMSKMHESEEYRKKIAKGIEVDPAEKPAQILREFSDFEIAMTLFTFLFASQDATSAATTWL
FQIMADNPQMLDRIRAEQLAVRDGDRNVRFSLDRIEKMTYTRAVVKETLRYRPPVILVPYLAKKDF
PITDTYTAPKGSMVIPSVYPATHDPEAYPNPDTFDPERWISGDAEKQVKNWLVFGTGPHYCLGQTY
AQNNLIAMIGKASLMLDWEHMKTARSEDIKVFATIFPMDDCYLTFKGREE*

>CYP570V1 |8207|Grascr1
PLSRIGPSTLLTSSAPELRRIGGARSSWKRSYWWYRSMRLDPTKECVLSLRDDRAHHALRGKLLPGY
TGRDLYGLEGIIDREVEKLVALVEGKYAGERMGSGGGIESLRAKEGDEGRKGDSKAKISGDEEEGFE
KIEGRNGRSGLKKMDLGRVVQYFTLDVISSIAFVSGIWIRMHGHLDKDADIFSYIAATDNIAATDKA
LPVMLLSGLLPPVLMFLQSPIMRPLMPKPEDGFGLGKVIGIAREAVATRFGPDGKKMEEKEMEKKD
MLGSFLAHGLSRDEAEAEAVVQVIAGSDTTATAIRATMLHVVTNPMVYRRLQREIDGAVEEGGVS
RPVVADGEARKLPYLQAVVREGLRIFPPVTGMMPKEDDTVCGRYVPAGTNVCWTAVGIMRNKEV
FGEDADVFCPERWLGVSPEKQREMEQWTFHCFSKGLRWECLGKDIAFIELNKVFFEVGQESEAMIR
LLKNGGLIKFW
>CYP5076L1|8251|Grascr1
MLVQYFDHTVLHAMGLVTILASSFFSGLYASLLIYRIYLHPLNKFPGPIGARASTLYLSTKLGKLDLY
KKLVKLHGEHGDFVRVGSSDLSITHPKAPALIYGSASKCTKTCWYDLEHPKQSLQQRRDRASHDQR
RRVWSPAFSDKALRGYEQRIQPYVDKLALQIGAHNGQKVNVSKWFNYFSFDVMGELAYGSSFGML
EKDEEHWAIKVLGEGLKPIGLMLPMWVFRLIQAIPGVCYQCGFSGSFKLSLAQWGTGGGINAFVK
NNAQSDKRSSLMNPIVPDIMSTLLEPLKGKELTYDDRMTLEGDTNLVIVAGRSKPQKLAKTLSDTTS
TSLTFTFLELARHPEHVDKLRKELEAVISDPSGDIPHQNIINLDHLNGIIYESLRLYPPVPSAVQRLTPP
EGLDIGGTFVPGNMTVYCPPYVMARTTTFIPERWYEKPELIQEKNCWFPFGMGPYGCIGKPLALME
MRKAIAKIVLNFDVEFAPDVSDRGKSFEEGMQDYFVVGLAELNLCFRRRGV*
>CYP531Q1|8268|Grascr1
MLPLIVLQAVVAYTLYSLWRHFNSPLRKFPGPFVAKFTNLWRVVKVAKGQSLDAQKVLHQQHGT
AVQLGPNMISLDDPSLIKTVYSVKGTFVKSDRYSIVDQKVGNQMVASIFSTRNNEFHASQVKPIQKH
YALKTIMDMEPLIDRATETLVRALDTKFVDNKLPCELNKWLLYYAWDVVGLVTFGEPIGFLERGAD
IQDIQKTTDLSVDYFGAQRQEAVDKEKTGPKDQLDLFLKETVGDSKANDFQILNWLMGNIQAGSD

TTAATLRAILYYVLKNPQVDRKLYGELEAAKLETPVQYRETRSLPYLGAVVQEACRMCPGVGLPLE
RVVPPEGLQLPDGRLIPGGTVVGMNPWIIHGNKEVYGQDADIFRPERWLRNEDEAETEGEFDARRL
KMKEADLTFGAGNRVCLGKNISLVEIFKIVPTLLLRYKFRFVDPNKEWQVHNTWFVRQSGMEVLIE
KRL*
>CYP59U5|8342|Grascr1
MVNISPSIALQALQTGMISLERPSLLPRFFKPIAGGRNLFDLGGTEWRPWRAVFIKGFNTEHIFSLVP
GMVKETQSYCKILNRRAAEGEISSLDATTLRFTMDLIGKTILNTSLNAQQGYNTLADCMLNQIRWH
QANAQTNPFSYLNVMRWYVHWSNGRKMDRYIGSLLDIRYSKIQAEMAQGHESTTKSVIDLILRAY
ATTSSSQNSPHLDHEFRTFAIRQIRLFIFVGHDSTSSTICYILHLLSNNPTSLRSLRTELASTFSGNSPDEI
GAQILAEQHLLNQLPYTTAVIKESLRLFPPASASREGQPNVSITNDAGQSCPTDGAFVLTLHIAMQR
SPKYWPRPDEFLPERFLVQQRPELYPPRNAWRPFEKGPRNFMAEGLVMVDLKVVLAFVVSQFDFRE
AYEEFDRVSVKVKGKRTHRGERAYQVEEGAAHPVDGYPCRVFVRDDA*
>CYP5093K1|8366|Grascr1
MESDGSDSELDWEKEIDRFTASVVFAIAYGRRIDSLNAGVIRERLKFMHFAASLNVPGKYLVETFPV
LKYVPRWMAGWKREMQDMGRMEARSNMGLVEGVRRDLEKKKESEKGVEKEEEGEEGEGKGKE
DGDGERSSVFGVSNCMTARLLSLRSSDPAGLGLSDRDFAYIPGSLFGAGSDTTASTLCSGILALLTHP
HILALAHAELDAVVGSSRMPTFEDEGDLPYIRSLVKEVLRWRPVAVLGGTPHSSTENDVYQGWFIP
KGTTVLGNSWAINLNEEYYARAEEFEPGRWMGGELKGREVPGYLLGDGKEGEGDVVVGKTETKE
EGDGEGSTATYRLSREYLQSFVPKKQHPSPSGHSSFGWGRRICPGQGLAENSLFIALARICWGFDILP
AEGLRREDYDTGMDAYTDGFNIRPKRFRVRILIRDEGRRKVLVEEEKAAWKVLGGMEVFGE*
>CYP5586C1|8467|Grascr1
MVNALVGSLLALAALVIYLKLSKWRQNHRTALRIGLPVFYSPIYLSDIWWLILQPMLVPILERLPQA
WTSPWLSLAQPFKVWSAGHRPFQEAGADTFVLATPNGNIIWTCDNDIIHQLFTLHPKVDQPVELL
KFFDLWGPTIGSVDGDTWKAYRRAVTAGLGTTVNKIVWDETQYQTDALLSHWIETEGSVIHSTRQ
WTVRLALHVLSSGFFNHRLEWDRRTTKSAPSGHMMAFDEALPALLEHLGLLYVTPQAVLGKLPGK
MFKEAHMSFTEVTSYFHEFYAQAASNIQEIAAKRYKSILEAIAISAADEELTGKVPLPKESVVGNIFFT
LLAGHETAGSTMGFILVMLAIYPEVQKWIQKQLDEQLNGRPKTEWSADEDYIRLQKGYIGAVQKEI
LYIYHPASLILRKVVEAITVVDDAGKSYRIPEGTLALLNLAGAARNPNVWKRPEVAQERIAALSDSP
ALYFNPERWLETDDRPDDFKGEVLQWQAFGAGGRTCPGRAFAQIEMTSMMATIFKDYSLELVVTD
EVLRDAKGNAALAWERTRDRAIKIMYDDIEMNISIGLKKEIPIRIVKRIG*
>CYP609Q1|8469|Grascr1
MPLNMLVCDHPTGLSIALVSLAVFGSIFLYLFRPPRFHPKAPKLTSEAWPIIGSMQFFTERWSFFERQ
VAHSHSGNFSFYAGDKPVIGISGDQARKLFFESKTMSLSDGYAALLGGTPRVNRKNNPMNSESVDR
EEGFTHYFNSRIAAVVKGPVLAKALPQLIADIRGGLDKLTAKPDRITNPFDSVYRIVYQLTLRIAACD
ELASDQALLDKTLRLFETIGETATPLSIMYNWMPVPAKFRRLWAGVQLYRVIKSVVDARRKEGRSE
DDALQFLIDHGDDIFKITTVVVGALFAGQVNSGVNASWILLYLANNKLWTQRVRDELDTVANRH
CPDKSPPIRERLAYVPYEAWESEFPVIDLCLKDCIRLQVSAACFRKNISGRDIPIDKAGTEIIPPNAYVA
YHAREIHQDPTVYQNPLEWDPARYLPDRAEDKKKEYAWLGWGVARHPCIGIKFAKLEITIIVAFFL
AYFEELQIVDEKGQPRGLPVVDMNSHQVRKPDEEVFLRYWPRED*
>CYP605B4|8603|Grascr1
MAAEMPRWAAALALLAVAVLVTHFFTSKERVAIPRDLPTELQYRVICPPYTRMCLISKASFEKGDK
RPYLAKNSRHSYVVFPPKFFDELKRLPEAKASAKAFFHRANYGSWTFIGQETHTLLKTIIADLTRSLP
ARVFNRQQDCRTAFDAIVGYAPDWKEMGLLITTFEIVAHVNACAFVGRDLGNNKKWIRAVMQSP
LVIHVAVVLMNSCPALLRPWLAPLAFAPTLKNQWDMKRLLTPMLEEDKRTFSNSTDKAQLLRPKQ
EGKIPLTAMLLSRYKPEEASIKQLITDYILVSFDSTPSTASAMFYMICELAAHPEAVEVLRKELEEVMV
DGKLPQTHLQELKRMDSFLRESFRLHPVSLFSLQRVTGKPVQLSAGPTIPPGLIMGVDAAAINRSPEL
WEKPDEFDMNRFYNLRQKPGNENRYHLLMTGPDSPGWGDGNQACPGRFFATNTLKLAIAHLIM
NYDIKLKEGHYPPKVTPLANGTWAPDVGVTAYFRTRT*
>CYP5589C1|8610|Grascr1
MYLTSMRQALLNSLNFSQRPFTVIRVAIIIIFTVAFLHAHGEEVYRRFQRVKRSSTPLALWYSKPVNEI
VADGYDQILKTTGRPFVMRWWAKDWIILPPKYLPDLRDAKPNHLNFFETISNAFYLYKSVGDLYSS

DRMATVVKKGLSPKLPLLTPYMLKDVEYAFRTEIGDLEESTSFTAQQLFSRIAHKAVARAMIGNELA
HDQNFMRICLDFIMSIFTTALVICDLPLGPFRDLFAVPLSFWHQIKLNRAEKILLPVVKSRILEWDNL
QGKTGHLDCIQWTLDLSEDNDKEKDPSRITHELLHNLWAATSAPGGLVLDLIFQLLLEPEYLEPLRE
EVATTTNKYGWSEQTFSSLKLQDSFIREINRLYPTGSIIGSPFRFSDGLTLPVGTRFAFPVGAILHDEAN
FSKPLQFSGFRFAQMRIEEASEGEGASRWSASALSTTNLAFGYGTHACPGRFFAIRIVKMIFSKLILEY
DIEWAGHVDKKPLPMWIEGQSIPNLTQKITLRKRLYKS*
>CYP51163A1|8622|Grascr1
MSTLHNENTLFEIPTSFSLQGYRIDSNVVICVLLTVGLGWYFLRSNYKSNGVRLPTYILLEGYAIAQLL
QSSSWVHNLVELIRHGSSHLYGTSSKHRVVSSPSLVSSVLRRPHHMLNNDPAQWALQACVFGSREG
MRVHLESISEALFAAVSSNLLQETKARGIIHRTNDLLRSLVPNLVASPNPWLSNVDLKSLRQGHAEA
NLYELIRDFTTFVSVRMMAGADFQDLNPDFGSDLFILDKAFMLLALGLPSWTPLPIMREAVAARGR
MLKQLASFSDRLEAVATGKADSNLQSRTSDVNDIFWQRSKVYTSLGLTMDDRASLELALFWAMNG
NTSPFVFWAVAFIYADQELLKKVREELDPLIKVSGRIDDLDVEEINPLTKVSEMKEGRKIDDLDMEGI
ARRCPVFKAAYMETFRCAHEPTSLRYVQQDFNIVDPTLGKDAKPVAFRKGTFVTFPHTVQQFDRGI
YPEPEEFRVERFLKESEGVIKVDYGNLKPWGEGSGICKGRLFAEREVLVAVALIVRCWDMQAVDGA
WKMPKKIPGTGVMKPSSSLRVRISPRT*
>CYP603C2|8636|Grascr1
MARIGRAMETEIQAVGNPIQTWPELVVQWKLRSRRSGIRYEAGSTMWTVPNSPLTIIAWGTLAALV
VLRALSPHPPVTKRALRHIPELRFDDGDESAERYRKDSRSLLYKGYEKFYTNTERFKYLRHGVAFQM
RNPIAELGPQLFLPMKYLDEVKNARVSLFSFPAFSEKVRHPSCSGSLLRRIDETVDVSSELQQCTAAD
RRLSTRRILAEAMHGEVIAALDETLSDKQGPITVNAYRFITGIVARVTALALVGPELCRNQEWLELSI
GTTHAIMGASNVIRHAYSPRWRWLARWRDEAPRQMIAMRKRAIKLLSPLYKDRAEADKSGGDLS
TTYPDCLAYLLRMRDADTSLDGIANQQLFLTIASMHTTSATATSILFDLLAHPEYEPDILADIEMGLA
EKGDGPWTLQNVAAMRKLDSFMKESQRIHPLGFITAQRLSLKPHTFKDGLHLPRNTIFQFPADAV
HHDPSVYPSPYNFDPWRFYKLRETVDPNRFHFATASDTSLNFGAGSHACPGRFFSGLVIKFVLVGLL
RDWDVRFEEGSGRERPRDVCHDLQMVPNAEARVMIERKKGV*
>CYP6251B1|8729|Grascr1
MDIYLACLGGILAHLLYFRHGEHHLQAPYVFWTALALPFLVSAILFQFGISTFVSALKHVCLLEATFS
ASLMLSTGIYRVFLHPTKHFPGPFLASLTKWYHYFLGGRSFQQHLFLDRLHHRYGDFVRTGPNEITIF
TPEGIVPIHGAESLCSRTAWYDNPLPIFSVATIRTKVSHEPRRRVWDQGFSMKALRDHETVILKHASI
MKDVIAAQDENHVRDFQEWLQYYGFDVMGEVQFNYSFNMLEHDKNRWVVDLIKDGNAILGSM
TSLPWAIRVALTVPYLTTDLKLFIRWCIDRLSQRMQSDPSNADTIGWLLQHASENEGIKADWFGLV
GDMMGMVLGGTEPVVNGMVFTLYHLAESPERTAKLRSEIKTLTSYSDNTQLAALPYLNALINETYR
LHPGVPSGGLRTTPPAGLMIGGTFIPGGTTVLTPQYSIQKREDCFERAEEFIPERWTSRPEMVRDKRA
FMPWGLGPFTCVGKNLGLMEIRTITVVLVDNFEISLADSEAKNRLLNGTLDYFAAVPGPLHLRMKK
REPEAQIAQPKSYEK*
>CYP5104H1|8775|Grascr1
MRIEGNARDRIEATGQVDGAVSGFGAPFSDIPAPSEAASVTRLWYTYIHWQGKQHEVLPRLHKKY
GDIVRVAPNEVHLCTSEALRAVYGPGSPCVKSDWFRVFQGQRQFDLVSERNRQKHAEERRLVSQIY
SGTNIRYLEPYIDITLSQLLKRFDEKLNRPFDISDWIQYWSFDTIGMLTFSQPFGFLSAGSDWNSALHQ
IRSAGDSAAWVGQNPWFYLLCERLLFPLLWGYNPFALANRNGVIREFATTQSQLRQKRAAASSSQ
QKKQQQQDIMSSLLTVHEQNPQSFPSSAVNSMVATNIFGGAETTTSSTRAVLYYVLRNPHVAHKLQ
KETDIICVAHAWPPGQPVSLNLAEKMPYLHAVVYEAMRLFPANGLPLARVTPPEGLYVNDRYFPP
NVQITVYSWSLHRSEKVFGGDAGEFRPERWLDDDEEGDSDDEGGGDDGDKGEGGGEENCMGIRG
RGEGRRTALKMTSRRGRSEKPTTVAAATVAKMTTIETATNIPANPMMNLNQNQTQTQPQPQNPN
QKLESHISTATSSPSAWARGFVSDGI*
>CYP5105F1|8788|Grascr1
MTRVFFLTYTELDLAFLIPLIIHSICLAFYRLYVSPLASIPGPRLAALTYWYECFYDVFLPGKYAFKLKE
LHGQYGPIIRLNPRGISISVPTFIDTIYAPGAGSRRNKDIEKTKSNKDIEKTKTLGTDTSLGGAASHDL
HRLRQGALAPFSSQKAVCNLTDLLEQRFKEVDSTLMWYASSAADGEREDEERRVVNLSDMYFAVA
IELVFQFLASPCLIHCFHAHIKISIVKQYCFGYEDGSLANLDKAKQSRENLAQMLRGVKFTTHFGWV

QRIMNYLPISLRKRFEPKGVQDMRDLRKSIHNDVAEVLKTNDTVVKATKAHSILYGLRDSEALPPH
KKTAQRLEDEATLLVMAGTESTAKSLTIAHLYLLSQPDILRKLRSELLSYNYTRLPLSKLQDLPYLSSI
MTEAHRLSFGLTGRNPRCAPDETLIYPPLPPQQTESLSSHENHDTTKSYVLPPLTPISAPTLLIHTDPAI
FPNPWRFQPERWLGEEGRKRRKYMLSFIQGPRKCAGMNLANAEMVAMLRTAAEWEMELAEGTN
EDDVKLNSQGVKARVLRRWRGGKR*
>CYP55D1|9052|Grascr1
MAAGQQPELPTFPFARKQGYEPPPENAELRQKCPIAKAKLFDGKETWLVMKHKDICEALSSEKLSA
DRNYLGYPEIHAGGAKAKEARPTFVNLDNPAHDRQRAMLADAFKPEAIHGLRALMQATVDRCL
DDFIKRGAGGKTVDLMEYFASPVPTQIILEILGVPEKDIEAVSNDSEVRHSTSRNAAETSNRNLNDY
VAKLVSERIEHPQDNLISKLVKEQYSKGKLDQTDVTTLAFLILTAGNAALLNSIGLHPQQLDDFKKN
PSLAPQVVNEVLRYHTVSSLNSRRAVKEDLVIGDQEIKKGESIICAMQSGDRDEEISPDPDNFDIYRK
RDPSSLLGFGYGLHRCLAETFSRQEIEIALTSLFKRVPNLKLAVPESELKWSPLEQNVGILELPVTLAQ
SKSGSAAQSNLDGAGNGNSDFYGIGIRIGIYLQWISSLLANIFWPDAIDANLDTNTIFLLALFVATVV
ATATGTSSISTPEIVVLLQLCFGFLFSVLTIWGIRTGASAGDPVRFPLIGSTIRLALATLISVYGVWFWF
YGLQQYEPEPDPCGTVLFIFSPVSVSGGLGLFYKIQSVVVLATYAILFVKEYLLIICFLCYTAIWTSVVA
LISLIFSPHFEEHSWEVLRPEFDRLAKKRKRNGDSWKKVFLRGIRTEIKTLWLVLVHWLQLSACIFWS
QANGEKSAGDRRPQLRPWIAPFVNPWIFVEKIAKALDSRVNPASSRDRILFERMSDFADTIIHVFNV
VAVIWTIVAVEFTLAWNNITDIYTIDSTGQLIPFIIGIVSFLRVAHGLSIQGSIKELSNYYMKMFDNTSP
EDLEMVKTTDFRRLDLSSVDIFLLPNRKRVIFKKQRPPRRRSVQDFREMRMHYNFEADGTVSDRGSI
SRRTSFTGDSALESFRGESGVKVRLYKDFRRVGPSREESVDEDHFDETSFQHEGTVLDRHSEARSDLG
QLPLTPRIAGELKSAQLDEEARGADDYESANRSMTESESRSSDSLLTRSRHSWFGRLSRSDSLFALAT
TIFFTFFVAFLPFMLLLRICRKISKHFPAVEERAQEATLQLTEMLFAHYTPAWYREIDFESQIEHLKLT
PEEARKLRMKKALQTWAKRQDKKLEHARAVRDPPFQFVNAAPMILTGAVKLLLTGAVKLLLLGL
LYLIKGRLTRNKESETHGDDDNVDDPPHQTEGEIKHSEPPPPPPSPKTHGDVAGNTHPSKSPPSAEE
RNSNGSPDVIRKNNPQTDGNNVPPSPQIFIERPLPSIRSRNSIESHISSSPSSLTDPISAAVHRDLLTLLD
SLFDYLHSTNFLSILRKRSLPLCTLHIATYVLLLCEDRKLHEAERWMTGIRDSVLHLKEVRVKKKGG
MMSRSKQHEETMQVWKKIMGYENWKECFELTVERMEDGMRERVERGLGAGSEGGSLRRSLRRRR
SSSSSNSSSEKRRRRKSNDDSSGKKEKWERDEEAEEERRQQQRQHASLRLRRFSWPP*
>CYP503N2|9076|Grascr1
MIEDGVHDLLSSILILVGLTAAIVYASQVLLSPERVHAPLIGSRSFFEPRWLLGLRFTKQGRRFLREGY
SKYKDSIFKVQCNTIEIVLLPRKHVDELRSLSEQKVSSPQALYNKSNGPYTGLGIILESHIHFDAVQRH
LTSNLAGFLPPIKDELDYARRKELPPCDHEWVPLAVHATIARLITRLSSRTFAGEELARNDEWIQIST
TYPMNSFATTLALRMVPKAIHPLVAMAIPHYWRVQNNISSAKRIVGQIIDERRETGVKRRRSKDEPI
DLLQWMMDEAAGPHAQTEKLAHRLLFVSKASVMTTSLLISHCLFDLCAHPQYLEPIREEISAALRE
DGGLQHTTLGKMRKLDSALKESQRLNPAFLMTFDRIVKEPLALSDGTRLPVGTHLAMPTDPMLRD
ADFLPSGADADSFDPFRFSRAREDSSKPTNAQRFQLASTEATSLPFGHGKHACPGRFFASSEAKLILT
HLLLLYDFKYPHGKSRPDNWLFSENMAPDPSARLLIRKSKTVERDLEKLLLKR*
>CYP65FT1|9093|Grascr1
MSAFQSNTSLSSGITSTYIAAGGLLLLGFLAKVIHNVYLHPLAKYPGPKLWAAFHIPHYWSMWTGN
ISFNIKDAHDKYGKIVRIRPDALSFNTSQAWKDIYGIQTGKQQLQKDPDFYLQQSAGSSIFICNDSDH
TRMRRNLAHAFSNTALKEQEDLMTGYFKLFMKKLWEEVNGPQNGKVDLVKWYNCTTFDVIGDL
AFGQPFGALEAGEYHFWIRNIFKALKRSQFDRITNAYPVIQWYTNIRDRLVPQANEARIQHAQYSA
DAATKRMALETDRKDFMSYILRHKDEKGMTLPEIKQTSKVLVLAGSETTATLLSGCTYFLLKNPEKL
KKVSDEVRAAFASDEEINMTTTAMQLPYLQAVLEESFRLYPPVPSTLPRRTGPQGNSICGQNMPPN
VSVGVNQWAANHCESNFRDAESFVPERWLNDERYKTDDLGVVQPFSIGPRGCIGKNLAYAEMRSI
LARVLLNFDLSLCNESKDWTNQKTFFMWDKPQLFVKLSGRTGRDLAMANA*
>CYP50330B1|9184|Grascr1
MWITLAPTVVAVMVKLLTKRAVLGAWQWDDILISFALSQYVAHILYLMAIYTSKLVGLTFFLTLAG
PHSRKTLLKALYAFTAFWAIATIFVISFQCRIPYPWLQDSDHCINKLAFWTVNTIVDILTQIGVGLFPA
LLLRHLQAAKRRKLTSMLSFTPNLLCIPVSIVRLVYIYGTPVTSDYTWNVFALELVTSLHAILSVLTAA
IPFVKQFIDSLVSLPEVIADGTQVYTSNSPNRTSNSPNRTFKNSLKGSSILSYGKGLFGHSRSQPTPTGT

VGNFTEISSVPEDHEMQRYETREGSQEQMDLKFAKKMDTMLAFLKRESVISKPNKQSGNIPAFLQSP
DGKLQQALQALTETTLATRATKAGSSSLRHLWHLNNPFVSTSPKLQHAYRHALIKHFNTSNALTW
TATTSAVAAVVHSYQYPSRNPTTASEPQRIRYLIRDLSRDATLAAVLQDLFGIRGVPRATLDYIGTEI
HRLTIAKKVVDKASSASAAAATAASLDLTSMQLSSDILIAHLRTLFHTTGASTSSPLSSHLLHSVSGPI
TPDCQSSSSSVSSEDFNPLNLIIPAFEAPWRAVFYTLLAALQPGPRYPSHVHALRNCAAEMLGAPLA
PMPAAAHAVVLESLRLYPPVRRVRREERVDVEALQRDERYWGPRARVFEPERFLVDKEEGERQREI
DHSRAAPEAAWMPFAVGSMKCPTASGYSVRLVAAVAGEVLRCVFPDNEEGANGLWWEMQGSE
WDEAAKMGEPLRAGREEYESVELVVENAT*
>CYP51179B1|9440|Grascr1
MAVPLSNLTHVIKTDISRHTADSIALMHEEIEVATAVAIGVPDIWTPIPLFATITSIVAQTTARVFVGL
PLCRNPEWLGLLINHTQAIIVAAAAARRYPLFLRPLVVPFTKEVKAVHAARRRMEALIAPMLKERL
EKWRADPDQEMPSDFISWTIKESEGKAWDPFIQSEFQMGIAFAAISTTTQLLTNVIFDLCTDPTYIPIL
RAELSNSKPLSSSHSPQTRASLAGLHKLDSFMKESQRLNPNMLLSLSRHIGQDLVLSDGKILPRGTSV
AVPLYAMNRDAAIFENADIFDPERFEKLREKKAMAGAGKFDEPDIGGEDKKLDEDGSDKGSPSLIY
THAKENMNFGYGRHACPGRFFAVNEVSWLRLSP*

>CYP548A30|9633|Grascr1
MFDAIGIENWSNTAYFLLLFIAGSASLSVASVVLYRLTLHPLAKYPGPLLGRVTDWYSVIQAWNGER
HLDFYALHQKYGKVVRYGPDKISINSNVALSDIYGYKSNVRKAQFYSAFPATKGSWSTHSAIDKNL
HARKRRVLSQGFSDAAMKGLQPHILSVIRTFTDAVGDFSVRNAFGNEKKERDWSTPKDMGLWAN
YMSYDVLGDICYGESFDTLEKPDNRFAINLVARSSQFHYLNAQMPGLKKLGLDRIFFRDLRANRLRF
MGYSRARLQKRMQLGTDTDRRDFFFFLLKAKDPETGQGFSTQELWGESNVLLIAGSDTTSTAFSSSF
YYLLNNPAYLSELTTLIRSKFSSVEEIVSGPVLSDLRFLRACIDEAMRLCPPVPSLLPREVLDGGIDVDG
MHFPAGTVVGVPIYALQHNPTYHPDPFKYNPYRWMERGSGSGSGEKGDGKGASAKELQLAQSAF
CPFSVGPRGCIGKGVAYLELTVALARTLWLYDLRLVGEVGKGREGEYSVGEGERGGVFC*
>CYP6148B1|9754|Grascr1
MLPSHYIEELTLLPDVALSATHALVDYFLGSYTTIDLPLFGRPLWSAVKNLSSQIPSLLRGMDCRCAS
RMEREFPYKDGVDTSETVDDNSLTGSEWSEIHPFAHMLAVVASLNAQIFVGEELAQDSRWLEISAD
FSRCIFLAAGYLKLCPDFLRPLAAWVIPHLYRIQAYRRDAKQLLLPEITRQREAAGKGYANLARTRN
MIEILEHVSPEKERSLEQLFDRQLGLLFAATHGASNHIVNVMIDLATRWDEYGEQLRHEVEQALEN
TEGSMTKAMLEKLPKLDSFMKESQRLNPASASLVPLQLSTGQIIPPSTHIAIAAAPMAQSSEYCKSPD
VFDGFRFYRMRQNEESVSSNAHLFTTPSLGCMMFGYGKLACPGRFYADAQSKIVLANVLLKYDLK
LPHNANRPQNVVFADASFPDPAATILIRARDDRR*
>CYP504A46|9917|Grascr1
MTIEILGRDYPVTGFLLLAIIYALLKYLNHADQPKIKGLPEVPGVPILGNLLQLGNEHAKRAAELAK
TYGPVFQVRLGNKRIVYVNNWKSVRELWIGNQSSLISRPTLHTFHKIVSSSQGFTIGTSPWDDSCKRR
RKAAATALNRPAVQSYMPIIDHECTASIRQLIRDSKNGQLDIDPIRYFQEFALNTSLTLNYGQRVDD
AELLREITDVSNRIGKSEKLTILRDYVPILRLGFKKNREAEEFRNRRDKYMTLLLETLKDRIAKGEDN
PCISGNVLKDPEAKLNEAEIKSIGLTMVSAGLDTVPGNLIMGLAFLSSPEGQHVQNKAFQEIVTAYP
NGDAWQKVVEEERVEYVTAFVKEVLRFFTVIPISLPRVSISDVKYEGTTIPAGTTFYMNAYAADYDA
SHFESPHTFSPERYLSPSVHASSGTPHYGYGAGSRMCAGSHLANRELFVAFSRIIMSMHLSEATNPSD
RPILDALECNAHKTSLTTDPLPFKLRLRIRNGAEKWLPE*
>CYP5043G1|10213|Grascr1
MEPLVDECSDLFATRMQESLGQTVDLGTWVQWYAFDVIGQITFMKQFGFMQNRKDDIGILDNLES
GSKFQTVILEMAMAEYDKTNDGIPRSDFLGFLRAAQEKDPNRYTDRQIISNLSVNIFAGSDTTGASL
RAIFYYLMKNPLRYQRLMAEIDAADEARKLSTFVTFEESNNLPYLRNQRIPPPLPRSPAVGIPLERLV
PPEGATISDHFLPGNSVIGINAWVVHRSSLVYGPDPDAFRPERWLVCESEHVDSANTGIDVKREGST
KSQLQEMEKAFFSFGAGNRVCLGKNISMLEMCKFVPQMLRRFEMEWVGPNGDGTGEWDVKGQL
LVKQHGVFVRMREREKMGNGKGEVGAGA*
>CYP544D1|10699|Grascr1

MDCGFCSHDISYSIGLLKCSQETFELAIPTLPPAILVNDPENLEYVLKNHELFVKGEFFRSNSWDLFA
NGILNSDGELWRTQRKAGLRFFSTNNLKYFIDDVLPPLVEKLNARLKAAAEVDATIDLQEELLDLTT
RLIGKIAYDMDISPDLPFSKAFDHASGVTANRFTNPLWRIDDLLQGFKFRACLREIKSFGLEVVNTA
VKRRTSTKTTLKDAPPPLHTILLDSLLDHISDPEVVADAAVNFLSAGRDTTAQSLTWTFYSLLRHPE
WKQKLLSSIKSSFPAANRASSIPLSYDLISGQHALPYAQAIFAEALRLNPVVPFELKECVEATTMPDG
TELPAGAVVIWIPYGLARSEKIWGVDAAYFNPERWLKEQADGSIQAVTRSAFENPVFNAGPRMCIG
KRMAEVLATSVLTQLVWQWDFEEDRGVGEIGGRRVMAESLTAPMQGGLPVKCHVIESQILVTPQS
VKDATLAPDRSRSLWFMDKGNIGCLQPSELDSDDDFYSSSHSTQASPPTRHPSGRAGSMRHSRPRPP
PTPNTGIPPSMPQNIPNPLLPSAPASPPTPAPSPTPLSRSSVWKRASVEVEEDIALKEFRLAFSRLDIAA
KKVWLESIVESCDNHLLSHLHHLVGPKLKKDPFQTLPNELCFEILTYFDDPKTFVRASRVSRRWREV
LSDDQAWMVLCEKHAYRRKSNESSPTSPNANNNLGFLQQLPNASTSTESSSAQSSDAKDSNLLRA
AEHVRNLKRPRPRSTHRSHFKHRYLVESAWRKGGQMTAKHITPDQGVVTSLHLTRKYIIVGLDNA
KIHVFNVKGDHQRTLQGHVMGVWAMVPWGDTLVSGGCDRDVRVWNMATGTAIHALRGHTST
VRCLKMADANTAISGSRDTTLRIWDIKNGTIKSTLMGHQASVRCLEIHGDLVVSGSYDTTAKIWSIS
TGQCLKTLTGHFSQIYAIAFDGVRVATGSLDTSVRIWDPVTGQCTAILQGHTSLVGQLQMRGSTLVT
GGSDGSVRVWSLESYTPIHRLAAHDNSVTSLQFDDTRIVSGGSDGRVKIWDLHAGLLIRELSQPAEA
VWRVAFEEERSVVLASRQGRTVMEVWDFSPPEELEGSSSAAWARPLSGLGTRSENSSAGGLVEDAV
MEDMLTAHDHTVAEIFRDQEKTAERDNRLLSQKNRLGIESSSASVPGSFPSDSSTETERADSEMPDV
E*
>CYP567AA1|10765|Grascr1
MAAFYNGLILGIAAIACYIAFRFTTALYQYFTIWSKVPGPLLAKFSSYPFCRSLLSGRNPFEILDLHEKY
GPVVQIAPNEVSCCSPESWQTIYNTKPSQPTFIKSDFYDAFSNGHPSIVSERDPAKHREMQKYVSGAF
SDRSLKEQEGLVVCVIDRFINQIGALPDNTGDLTKWFNLLTFDVAGELAFGKSFGGLATGSMHPWV
AVIMASMRESSLVDPFRRFPLFGLLYVMFRPGWLRSLLKGASENADYAMRAIEERKRNDSDRKDFM
TSMMSEDIRVKHQVSDKQLAVQADHLVFAGSETVSTALMVVVAFLGRDAKSKARLIAELDNAFSS
PNQVDMISAARLPFLRAVCLEALRIYPSVPIGLPRIAPKGGTVIDGYFIPGGSIISSHGLATSHYSQNW
QDPKTFNPDRWLTPSPADKLDACQPFSLGVRGCLGRSMAWMELYITLAKLYLTYDIELLDKDEDLL
ENVEVYLFWKKPELRARITVRHFKGEAIDVVAVPNARSQSMTSTLTPEHRVASKVEIDASKSRLPTR
SAKHDSVTIPKVGSTQSLKETEPLSYDFWTKNDLRQKFTIITPGRKYAALFFLGAKGGGLAAKFNLQ
SKVTITKSGQARIFYPPRAFDIARKHHWTDWLIIYAPDSKSALALAYEAEFDIPAVNIVKKLYNHVY
MSGGHWAFSIDTRSHQISETWVKGHGVFSGQEPKSIKTLGAEKQNFRQIVQNYIKRPWERKQENIF
ASTRQRYSGKEQMMDHVDKTQKSRTGEKALWSAPEL*
>CYP6306C1|10946|Grascr1
MSVLVYVAVIAAGAWLLCLRRISSPSKLNLPYVKFDRDNSASRYRVEYETILRKGYAQYIEKEQPFSM
RDPNDLERPLAVLPVKYLEEVKWIPEDRMSFWKHIDKQSILTQIGGPGITEEVALTARQGLNRALVY
LVQSLQEACEAAYAKEWPACPEWTPLHPHPLITKVFASMSACAIVGPKLDGLDSEWQTVSMGFVA
AAISAPSKVKNKYPRWLYWLSRYTNDGVKTMWKHQARAVELLTPVLHNRIKATEELKARGVKKS
KGPRKYEDGVQWLLDAHAAHGKDLTPTQLARDLFVIMTVSIHSTSGAGLSILFDMLDHPEVLSEIQ
AEILQVRSRLPAGIWTRKALGELRILDSFMRESTRMHALAQYTAVQRILTSTWTFKDGTEVPAATTL
VFPSYHHSLDPAVHTNPDTFDAKRYLRKREGSDTHKYHFASVSKDMLQWGSGRHACPGRFFAQET
LKLMMVRLLTHYEFKHTEQDKEVPRFIPNNLFIIPNPALHVVFRERQVPLQ*
>CYP6789B1|11355|Grascr1
MLDLHKKYGPFVRIAPNMVSFSDPLLLPQVYHLRADKTPFYSTGIAGEVPPLLQIKGDAEHANKLR
VLNPSYSLKHLQLQEDEIDSHIHRTCTVLNVRFAATGKSLDFAEWVRYFVYDSLIAFIYGKPLGMVE
EGKDLHGLIREWHAIFPLGGMVSTLPSIVHPLITSRFLKKYLMPRKGHTRGSGHIMSVSQSSQNASH
RCFGSLVIDIQPQVHEKLFRERLTQPQLSRPGNVFDSVRQTKFLDGRQMTLDEAERECFLVTVGGQD
TSPAFISAFFELLLSNPRVYAKLVEEIDAFEIEGKLSSPVPRYDETCAMPYFMACVQEALRLEPSASLIL
PRYAPKGGIQLGGHWVPENTEIAANPYVIHRNQDVFGPDAEMFRPERWLEDPARRKMMTKYSLA
FGYGSRKCLGKNIALFVSQKLCTQLLRQFTMSRADLQYPTVLNWGLNVYFGQNICLEARTPSQG*
>CYP5089C1|11421|Grascr1

MGAAFSAVLTLLAVVATIVLYRCLGHPLARYPGPASAASSNLWQIFTFFSGNSHIVVKGLHEKYGP
VVRVGPSSLSLSDLAAHHYIRGAGDAFERGPYYDFARDPSTGKGSILSIPNHSQHKPVRRMIGPALT
ATKVQSYEPTVLKNVQLLLDAIAAQHSEGDTDTVDVSTLIHCFLIKTTFETIYGTPLPGGLDAQEHISI
ALRDVSKFAFGSGYLPWLSWLINSCPVRAFTHRPKYEKDGSPIGMTAIVSKARKTLAAVLASSTTPTS
LHPSVLRSFGEAGAASEKRLSNEQISTECLNLMIAGQGSTTASLTAVFYELSTSQGQEWQHRIRKEAL
RAPDAPALALNAVIKETLRFHPPFACGFPLDVARNAEPSQIPGLTAPLPTGTTVTCNFYVLGRSKEI
WGEDADEWKPERWLSAGKEGEGTSGRDLEDRFVVFGKGPRSCLGREIAMKVLTRTVTEILGRWRIE
SRGELVGKNCFEMQYNSCRIAFLERGRSIDIQKIE*
>CYP51193A1|11487|Grascr1
MLPIVLRTGFPISISPVTPSNPLWLIFAATYQPLMAKYLPTAIYNRIRMTIFGWQFRERWGVNAELGT
TYVLTSPGGNELWTADPELMNQVLSRRNDFVQLEMASRIMSFFGPNITSSNGDQWLRYRKTIAPLL
SERIMHAVWVETVNQIQQMLREILSHESTREPESNEVIEGIRTIAINVIGSITYGNQHPWIDSHRQAKL
SLKSELTFIESLMWVVNHHVIAVFLPDKIMRHSMMPRWLRRLGAAVVAFSDHAHQLIQAHRMNS
DGRPTIVGSLVRLVDISSSQAVSGKDTSAWTEDEVVGNLFNLTIAGFDTTASTIAYALLALTIEPQWQ
QWISEEIDSAAYSNKETDYPVMFISLTRCLALMRPAAIRTDDSGGYERVPCNWMRPCVTRALWSRSS
RIPAVEVAAEEY*
>CYP6524C1|11489|Grascr1
MAALTVIVYLTTTLLIWNLYSLFQNYRLARSIGLPRFICPVGPANAAILIIGGIFGHRLSRYLPAFIYDR
VKLIIVGWEFYARHTMHAKLGPAFVLVISGSNTVIIAEPEMGQAVLTRRKEFPRVKISSRILGVFGPN
LVSADGEEWGRHRRIVAPVLNERIMSVVWKESCNQTRDMVEALGKRITATDNREANDDASDKIC
DTVDSLRTVTINVINSASYGSERTWSSAMTSSKPPPGFNFSFMDTVVIIAKNLMVSLLLPARLLRLPV
MPSSVQRIGQAVYEFPLHAKSIIVREREAPWSAVGSGNTLMGSLVRAADTESSQAGGKPLRSAIYLSE
DEIVGNLFNFTIAGFDTTANTLAYAVMILALDTQWQDWIIAEIDEVKRLHPEQGYPDSRPETLRLYT
PIPHIARIVEQPTRLLNHELPAKTMVQVSAAVIHVSPDFYGPDPLAFRPTRWIAPPSSATKAPRTLAC
EPLVTPDTRTFLPWSAGPRYCPGTKMSQTEFVAVLATAFAEWRVELAPKADQSVAKAAKELRSVM
ADSKLGLSTQVRRPKDVRLRWVKR*
>CYP51F1|11779|Grascr1
MGLLEDLGGYLSETSTQSLFALFGLGSAAFLALAVVLNVLRQLLFRNPNEPPLVFHWFPFVGSTITY
GIHPYNFFFECQKKYGDTFTFVLLGKKTTVCLGPKGSDFVLNGKLSEVCAEDVYSPLTTPVFGTDVV
YDCPNAKLMEQKKFVKFGLTAKALQSYVELIVKEVHDFTTNSPLFKGNEGVVNITDAMAELTLYT
ASRSLQGREVREKFDHSFADLFHDLDQGFSPINFMLPWAPLPHNKRRDFAREKMASIYLDIIRDRRE
SGKEKDSEDMIWNLMNCSYKDGKPIPDKEIAHMMIALLMAGQHSSSATSAWILLRLATNPQIVDE
LLEEQRRVLGSESAPLTHDNLQLLNLNSQVVKETLRIHVPITSIMRNVKSPMHLDGTPYTIPTSHWLL
ASPGASSRMEKFFPEPLKWDPHRWDGEQVIKAENDEKVDFGYGIVSKGTNSPYIPFGAGRHRCIGE
QFAYIQLGAIVATWVRLIKLSTVDGKKDVPDTDYSSLFSRPMNPAKLRWERKEKVEKS*
>CYP50230C1|11860|Grascr1
MESSMALESHSFLLASTAIVVCTITYLVSAYFTNGLNRYPGPILAKFSNLWYYYDVKSNHHHDHLIA
LHRRHRDVVRIGPNRLSIANPDYVGRIYSTQDAYPKSEMYDSFSLRTKEGRQQITLSQRDQKIHART
RKAVAHTFTLTAMQDYEPHMDEIIKLFLRRLKEKTEETSNFLCDFGLWIRFYVTDTILYLTFGSTFGF
LDAGKDSVEFFDNMNANMDKDSLYIPMPWWSLITKFNPIITLFSSENSFVPGWCMTQIRKRQDARA
ELEEQTGQSGIDVKSDFLDKFLDASKISTPPGIDYTALIDWCLTNLQAGSETITVELISTLYHLLKQPEK
MAKLLKEIRSTSLSNPVTYKEAQKLPYLDACLREGLRIHPAVGLGMERTVTSLNGLKLPDGSVVPCG
VLVSMNAWVLSRDRSLFGDDADDFRPERWLQGKEETEAAYMERMNAWKRADIVFSYGVHSCSG
KNVAMMEMYKVLPSLLLEFDIELKDPKKEWGVINRFIVRQTGFECVVRQRA*
>CYP682BD1|11925|Grascr1
MAQALLLGLSLLGLYLLCIVIYRSVVRIAPDEVHVLDHEFIDIHFGTKHRKDKYKPHEGIFGMPQSVF
TTASSDLHKVRRAAIAPSFSRRSILALEPAMKEKIGKACSRLESFKSSGKAIDLRLIFTCLATDIISEFTF
AECFNLLDTEDFAPAWRKQFSEGARNFQWFKHFPALWPAFRSIPNRLLAALMPDMELLRKWERN
AQKLMKESINTFEPSQKKENSPKVLYDLLASDLPKEEKHFDRLWQEASIILGAGVETTANTLTVILYY
LATIPEYNARLKNELRAAMPDTSQELKLCDLEQLPFLSAIVSEGLRKAYGTTTRMIRVAPEATIKFNG

HEFPPGTAISMSPIPMLDDPKIFDQPDKFQPERFLERNSRADLMVFGKGPRMCLGQNLAIAELYLTV
AAIVRRFDLELFETDYGDIEFICDAQVGLPKANSKGVRMIVK*
>CYP532A35|12065|Grascr1
MIEVLLRIFVGHWPSVALVGVALYFTLNYLNHGLNKYPGPLMASLTDWWRFLDVWGRRPDKTHL
RLHRQHGDIVRLGPNTLSFADPKAIKTIYGLNKGFRKCLFYVGLGPLVQYEAGVDVVTKQFLDQTE
KLYATKDSICDFAQWLQFYAFDVIGQITYSKSHGFVEKNEDIDGMVGYLGRLFSYVAPIGQIPFLDN
FFLKNPIVRLLDRCGVRTFAFPVVTFARARMNERILKKSRTTATFTSNDRNAPRDDLLSQFFKAKEN
NPAFFHEGRVLVMAVSMAFAGSETTAISLAAVFYYLLRNPHSLNLLRKEIKEAIASGKLESREDGLTS
WAESQTLPYLDACIKEAFRLHPAAGLPLERLTPPGGIEICGEKIPGGTIVGCNAWVIHKRPEVFGEDI
DSYRPERWLDVSADKRKEMDGTLFQFGAGSRTCIGKNISLLEIYKLVPSFLRRFDVRSPAVFWTFH*
>CYP6102B1|12145|Grascr1
MSFDLTMLDDAILRNPIHESVIRNQLTRTLGITLDSVREELSQSLDTFWGTSADWHDVPLLESLLKVI
GRSSNRIFVGLPLCRNEEFVEGILGFAYAVVISGILLRMIPLCIRRFVAWPLLFANRKNFNKVAKHLV
PLIQQRQANISKIDNDEKGASEEPADLLQWNIQNSMRNPDPREWDPTLIAKRTMATNFAAMHTTS
ITTAHILLDLASSSPETKVLEDLREEVRQVYAESGDKWTKASVAKLFRLDSVIRESLRMSPLGLFGLVR
KVVAKGGIVTSDGLHIPEGAIVAINHYGEQVIHERHENADTFDAFRFSRPRESLMTNDAVQNSSEH
EAANVIEEKKLASTSTGVGLLNFGHGKHACPGRFFAVQEIKLLLAYILLDFEIQYLKERPKNQFLGTS
CLPPAKATLRVRKLCSRP*
>CYP5077E1|12176|Grascr1
MLDGIVVAIFTVAFSLVTYGEVVRIAHDTVSFASEEAWDEIYAPRRGHPWAVRDRDFYLALGDQAH
NIVATSNTEFHQRAKKVLSHSFTAVSLLNQHVIIERHAETLVRKLRQAVYASVAPIPVNMTDWICFF
AMDVIGDLAFGEPFGCLELGDYHEWTRTLIEYLNAIAFAVIPRQCNLLGFLFNKNNRNFELMSHEEI
LSTFNIIIVGGFETVATVLTGIITHLIQNESILQQLCHEIRSRFRFGSEIEARNIANLPYLDAVINEGLRM
CHPTPDGFPRVVRPGGDDYMGVWLPEGTKISVHMLTVNRSKKYFTDPDCFIPERWLGGNKRPARY
RNDRLSASKPFTVGYHSCIGRHLGLVEMQLVLARLLWAFDLRKVPGCDVKFDDFPIVGIIQKQVVP
VLLKVRNINNDNC*
>CYP51196A1|12380|Grascr1
MQLLTASLAFAATCIAYYVYSFVSSYATARRIGLPILLTPVHPFNFVWIILGPLIIKLLQSIVPARLLFRL
DVAIRGYEFRLRNKLHENLGPTLLLVGPGCVQVVTRDAEVIRKVTGHGTSKGFAQSALIEQITGIFG
PSIIGANGEDWQRHRKIIAPIINERISLTVWNESVQQAEGMLDHFVKESFGNTQSATDQSIKGLRRIVI
NVLGSSAYGTPRAWTNHEEQPPPGYKLGYINSLLAIADHQAAAIFFSPRTLLLSIMPAFAKEVGTAK
VEFPRYVESMIAKERSSADVERHNILSAMVKASDRGRDASESDTKEAALSLAESEIYGNLYLFTLAGY
DTTSNALAFAFTLLAIYPEWQEWLIEEINLVFKNGDLSYEQAFPRLRRCLALMFETLRLYSPVEHLTR
TTIGPQTILTSSGSNHTIPSGLDVIVSQAGVHVDPDYWPPDPLMWKPSRWITDTSSSDDNEKFQGVK
HERLMEPEKGTFIPWSFGPRICPGQKMSQVEFVAVIATVFRACTIEPVVLPTEKTMDEARRRLAGILA
DSEPTVSLSMKRPDEVVVKWVRRH*
>CYP5105A6|12927|Grascr1
MPAEVRNKIWRLYLEEEEVYIGSRNKTENQGLAKPPGLLLTCKQVHTETIRLFYASACFLIGKKSCLR
QWLSGIGTDDASLVQNIHLKETHGWTEEDLHYLDMVKEQARMTRVDVVSLSKQCDLKAGVLKGY
AQLARAYTVNPLQLVREMLKVADYAYPYGWHWTEEPPEKLRRLRADFELISVLKEAGDKKEEEER
ARRRELAKMPENFMEWSRWILYGAIWRLFLSPLARIPGPKLAALTWWYEFHYDVVQPGKYVFKIQ
ELHKKYGPIIRVTPCELHINDVDFLDTVYAPSSQRRDKYEYQLRTLRIPTAVGTTSDYHLHKKRRETL
SPVFAKKNVLSFEPLIKSKVDQLCHLIEEFAAKGSPANLSDAFFAFTTDVVTNFLFALQSNVLADPTK
AAVLRNNSNQLLLGIKVNQHFPWIPDSFESLPLSWSKHLMPPGLIDIMELSGRTDEVKSTFQPTKLPP
VCLIGEHWEYFRGIRVRSELLEIIKARDEGEKQSSYDGKRSVFYTLLESSVLPPAEKAISRLEQEGTLLV
LAGSESPARSLTIIFYHLICNPGILQKLRLELQTAPIDASWSRLEQLPYLSAVIEEGNRLSWGVTARLA
RIAPEPLRYGSYTIPAGTPVSITTLAAHTNETIFPDPRSFVPDRWLGAEGATRRKYQMAFNKGSRKCL
GVELARAELYLATAALARRFDMALYETGADDVAFLHDYQVAMGKLDSLGVRVLPKMRNDSQS*
>CYP51179A1|12972|Grascr1
MHNEDGLLSTLAASPLLWAPSISVALIFIVNSLKSRSKPSTDFCADTISGDDTTDVRKSLVWAFEKYS
KNGKLFKIAMPDQDTVILPPKFIPEIGPLPEKKASLYAEFAKMFLTQYTLLGIAVPLDNLTHLIRTDV

ARRMVECIAVMEDESSFALTEKLGTSNEWQPFPAFLTVLGLVAQITARIFVGLPNCRNPEWLHLFAS
HTRSIRNGAAALRKYPKWLRPLAASFVKEMRDIHYARKKMRELVTPIMNERLAKLKANLDADLPS
DFISWTLKESRGQAWDPFIQTEYQMGVAFGAITTTYVPHPSILSQYSHPPRTQLFTNVLLDICAHQSS
IEPLRAEWQSMLDSTNGIISKPSLMQLPKMDSFIKESQRTHPNTLLTFSRYIASDFALSDGTILPKGTSV
ATPLYAMNFDRSLFEDPEMFDALRFEKMRKQAEIEAGGKKVQRWGLWGLTHSDAVENMNFGFG
KHACPGRWLAANEVKMLLGKILMEYDVRLEHPEKGRPDNILIEKALVPDPTKRVMFRRR*

>CYP65FU1|13049|Grascr1
MASNSVASLFYLLYGLGRAVYNVHFHPLSKFPGPPSRAGFWLVDLLELCVGSKIWEEKALHEKYGP
IGKKQLQKDPAFYSNPQSGSDIITANDKDHTRMRKLLAHAFSDSALREQQPLMTHYFDLLIEQLQK
QIDGPANGKVNVVAWYNFTTFDIIGDLSFGEPFGSLANGEYHNWVATMFAGIKFSRFIRVAYRYKL
FGVVLGIFYKLFTAVGEGRKKNLMYAHDKALERLSAKTDRKDFMTYITRYNDERGMTERELAGNT
GVLVIAGSETTATLLSGATYFLTKNPRVWNKLKDEVRNAFQSAEDIDIVSTGKLPYLHAVLEESLRR
YPPVPSRLPRKTGAEVEVIDGHVVPPKTSVGVTQYAANHSPANFNNPEVFAPERWLPNPPAEYAN
DDKASMQPFSLGPRNCLGKNLAYVEMRSILARIVWNFDMELCPESEHWAETETGNQKAYVLWEK
PPLWVRLQRREKD*
>CYP617AA1|21|Grascr1
MAFLGGTSFLVVYAAVATALIRHHVPEYLILGSTTLTFVTGILTLFGLSFTYNVILYPKFLSPLRDLPM
PPDPEFIMGHGRTIIKEPTGIPARRWINSVPNSGLIRYLHIFNQERIITVSPKALSEVLVTKSYDFPKPDR
SRTALVQLLGNGVLVAEGDAHKFQRKNLMPAFKYHHVRDLYPIFWSKSGEMINVLSKEISTAIESD
PSLDAKGGYGFEMGNWLSRCTLDLIGIAGLGFDFQAIQDPHNHLNEIYRTVFSPSGGLGMRLFLAA
TAFFPPWLMQRIPIKRNKQLAKARHEIRAISRKPPQTSPPPPAPTTTPTTTINPSDHDDDQPQRSQQK
DILSVALSSGAFTIENLVDQTMTFLAAGHETTATVSAWALLELAQRPSLQSALRAEIRAALPSFSDCD
EKGRPLKTNIPMTADLMDKSCPLLHAGTTVFICPWAVNFSTEQWGSDAGEFRPERWLVPEGEGKE
AHAKAAAGGAESNFSNLTFLHGPRSCIGMGFARAEFACLLAAVVGRLEIVLDLEEIKKKGLKVGEV
QTTGVVARPKGGLRVRGRVVGGW*
>CYP534Q1|138|Grascr1
MLSLALRALGALLLFWTAKNLWSWKKNIDAAKASGFRYIPVPVTIFSLVWLIVNGPVMRVLRLIPK
QHRPLWMDKMESMHSWKDLFSAYDIDPQAATYLTVSPQKCMLWTAHPEAITQITTRRNDFPKPG
RMYRSINIYGRNLVTVEGQLWRRHRKITGPPFTEKNNHVVWAESIRQATSMIKSWTGKDGNSSRTI
TTIAHDAMRLSLHVISRAGFGVRLSWPDDEDANESGKAGEMSATNLGTGHEMTYTEALETLLHN
MLPVVAIPHSLLKILPFKKTKLAYQSFIEWGKYMNEMMQAKKIQLASGEDEEGGMDLMGALVKSA
GYGASNTLEKGSSQAETLSDEEILGNAFVFILAGHETTANTVHFSLVYLAMNLASQRRLQADLDSIF
QGRTPEQWDYDFDLPRLFGGMTGAVLNETLRLIPPAIHIPKESPQPQSLIIEGKEYTVPGNSLVLLSA
VGVHRNPRFWPHGPPSNPDKPIHETSNRDNDLEEFKPERWLLTGEHATCTTANPTSTSNTNSGAT
KPSLALAPDSLPRSAETEDLAVNTAADTAASLYKPEKGAYVPFSEGFRSCIGRRFAQVEVLVVIALV
MYGWSVELAVDEWASDEEVARMRKGEKEEVYEKAREKALGKLRNNMRTVITIQLRGGVSIPLRFT
RKGGERFER*
>CYP51182A1|394|Grascr1
MSSTMLPTDIVFSNDRDALFLCLPLVILGIYFSYKFCLARRERLNFIQTHKCSATGPSYPGISFLFGYDF
LLENVFNVIKYRFTQGVAERFERYGPTFKSQILLKGIINTIDPENLEAVFKTHFEDYKLISAREYMMKT
FYGDGIIGNSGHEWKKSRAIVHPAIANLSPDQAIFDRHASHLVEAIRSLPAGPFDFSKYASEYMLDV
ASEIFFGESTNVLQGQQGDARLRQFATDFTMLGRMARVMTIFNRQFPFLRYLMFRNDYRPTTQRV
NEFVDIEVQKAFASKQTPPPSPAEKQEDFKTTRTPKTLVEGLASQCPDKQRVRQETLNCLLPARDSN
AIVLGETLYKLARHPKIWAKLRQEVSDTLHGSAAPTIADLNKMTYLKYILNEGKSIHTSHLPPPFSLI
LLPHPNLCILVLRLNPTISTHGRLATRHTFLPRGGGPTGLSPLFVPRGTYVVYSIHALQRNPAVFGPD
AESFRPERWENVSPKQFEYFPFGAGPRVCLGKTLGWTMVAYAVVKMAQGFREVLPRDESPYEEAR
AFGFMNRCGAWVECLGDEEEKGRGGDEGDGW*
>CYP52Z5|641|Grascr1
MLNLELAIAEQHGCKPVTPEVPYKWPLALDLVKRQIDILYSEHTFELLTPYINIVGTVRVETFGATGF
VTTDPENIDSILSTHFEDYGLGSRRLKQNLQVFEAHVDELIDGLRYAAETGGPVDMKPLFFEYTLNT

TTNLLFGEPHSSLSKGSSEKRPTDFRKTCKNVRDWATFFSSKAMKYKDDVGEEEPAEKYSFIIDLWK
KMRDKRLVRDQLLHILVAGRDSTAALLSWTLYVFDNIDLTLLALLIHTKLSPLLRLYPTLPLNLRFA
NKTIVLPRGGGPDGQSPILMPKGSGIVWSVYHLHRLESIYGADSKVFRPERCESGELFKKARSGAGY
VDFNGGPRLCLGKDFALMEASYAIIRILQAFSAIRLPRDVPNEPVGAEKQTYTIGVTPTDGVKVLLY*
>CYP505AL1|1642|Grascr1
MTTPIPSPPALPLLGNAFDLNSDYPLGSLLALAERYGKIYKLNLGGSDRLFISSNALINEVSDEKRFEK
AVSGGIRQLRALTHDGLFTAITGEHNWEIAHRTLVPAFGPLPIDAMFDDMHDIASQLVLKWARFG
PENKIVVTEDFTRLTLDSIAITAMGVRFNSFYSNEMHPFVGGMLAVLDEAGQRSARPTLVSRFMSSK
QYKYDQDIAFLRGIAKDLLDQRRQHPTDKKDLLNAMIFGKDPRTHEGLNEQSIIDNMITFLIAGHET
TSGLLSFLFYHLLKNPAALQRAREEVDALVGTSAVKVEHLRKIPYINACLREALRLNPTAAAFSVQA
KKDDVLGGEYFVHKGQSIACLLPAVHRDFEVYGEDADVFRPEPQLAVVMLLQNFDFTMDDPSYQL
EIKQTLTIKPKDFFIHATLRHIKPKDFFIHATLRHENPIAGGSGGVEHSATKPKTKTPTQSTPVGATGT
MSIFYGSNTGTCESMANSLASAAAAHGYKASVDILDKAVETKPKDGPVVVITASYEGEPPDNAVHF
VNWVRNLKGKALAGVKYAVFGCGNRDWVTTYQKIPKLVDDVLADRGAERLTERGFADANDGEL
FDTFDRWQDEQLWPGIQEAFGKQDGSDETENDGLTLELSTSSRSNLLRQDVQDALVTENKVLTAS
GVAKKMHTEIQLPTGTSYTTGDYLAVLPLNPSRTIRRAMARFHLPWDATVKIQPSSRTALPSGRDIG
VFNIFSAYVELAQPVTLKQATKVAASIPDADLRSNLEQKISTSFKSEIQSKNLSLLDLLEQYPTATFSL
GQFLDSVPTMRVRQYSISSSPLADPTKCTLTYTVLDQPLAHDPTKRYLGVASNYLALAEPGDRIHVA
VRPSAGFHPPKDDTATPILMICAGTGLAPFRAFIQERALKIEAGKTLAPAILFYGCGAPDTDAMYSD
ELAKWEEMGAVSVKRAFSKKPEKSEGCRYVQERLWKDREKAAGLFGQEAKIYLCGSGKVGEEVGE
AIKRIYGEFKEREKGEKVGEEEREEWFRGLRGGRYWADIFS*
>CYP5042D1|1654|Grascr1
MEYVQTGPAALILLFTVALWELSKPIDKRWISLPSRWKLPPGPEGRPIVGNLLQFFRARDSGGITPYL
DSLSKYGEMTTLHMGSRIWVILNSDRVVSDILVKYGKQTQERPYMPVGSGLVSNDKRWVFRQSPK
WQEGRQVVQQLLNGSYLHLYGDWYQTESIHLLHAYLHDPQHWFAHHFRYAVSVLYCLILGEPFD
KSKADLDEYQRVQTEFTLSLNRHYVDFFPWMAKLPSFLHYWAAPWKRMAANHRAFFASWWDPV
ASAIDAGTAPPSFTRDSLLHPDTKYRGTREEAMYLALSNMGAGSDTARMMLNTFVMAMISHPSAL
KQLQSDLDQVCNKDDGTLRLPELADMPSLPYCAALVKEVLRWRPTVPLNPPHELTVPLEYDGYYF
PPGTAFLINNVALKDPRWDDGGVFRPERFIDGKGGEMDPVLGLWAFGGGRRICPGYRVAQQALFV
AYARIAYCFDIVPRGDVDDTVLRREILGEPFPVEIRVRSEAHGKLIEEEEERTKVDGWWPGQVAVGG
LSVLK*
>CYP505AM1|4286|Grascr1
MSCPFAPDATGPAPHPLPDDMTESRTAQSTNSGAVVLPQPPEHLFGVLGNLPDIDPSNPPMSLWKL
ADLYGPIFKLKLRGNFVIVCDEKHINEICDETRFEKFVNPILQQIRALLGDGLFTAHQPEKNWALAH
RVLVPSFGPMSIRKMFPEMLDIASQMLLKWDRQGKDHAIDVADDFTRLAFDTIGFCAFSYRFNEFYS
DKSHPFAEQMSQVLLESGRRAHRTDIENSMHRWSEQLRQDNVKKMHAVCDEIVADRKRNPQPD
NKDLLNVMLTKADPVTGEKLSDESIRFNLCTFLVAGHETTSGTLGFCMYQLLKNPEKLLKAQQQV
DEIVGDGVLKLEHLTKLSYIDAVIKETLRLTSPIPAFSVHPKEDTMLDGKYPVSKDTLIEVLIRPLHHD
PTIWGEDVEEFKPERMLKGRFEALPPNSWKPFGNGMRACIGRSFAEQEMLISVALILQRFQIEFADPS
YELRMKTTLTMKPSDLKIKVSRRPGKSLMTGLPGGGLAEPTLQSTKGTHAGDNRSSKESKQFPLAVF
YGGNSGTCKAFAEDIETKAASHGMKATVESLDSATEHLPTDRPVIIITASYEGKPPDNAGKFVKWLE
LGAEEQRLRGVNYTVFGVGNSDWASTFHKIPKLVDQTMHQMGAQRFIEAGYTNVKGDMVGPWE
DWSDKLWEGLSQISGSNTETKTDNGLEVTVKLSSTKTTLGTEKIKTGTVLANYELANADIGPAKRH
MIVRLPEGTSYESGDYLVVLPRNPSDTVQRVLKRFELSDDDVLTIANSSKKFLPIEPKSAIDVFSTSVEL
GTPITRRQLATIATFAPENLKSTIALLGTDDAAYSSLLEKRYTILDILDEHPTVALSLSVYLDMLPPLST
RQYSISSSPFAHSPDQDPSIMIKIPPLQHAPLPTTSSMHPPGPLTAFSAASHHLTLLPAGPAIKLIASFA
QQLLASDYPPTLQHL*
>CYP51197A1 |4884|Grascr1
MPSLLSALQTLTYSSSTMPSLLSALQTLQVQIPSLPFILLSFFFIYLTSRLYHLRRHIRLAKSTNLPYILSP
LTFNSFLILLYRPILRLLSYFPSSLTHPWISVISPLSSWDNGHATYSSLSSDTFLIVTSSGITMRTCDPQVI
TQITQRKNDFVKPVETYKVTDIFGKNVLSVEGDVWRRHRRVVGPSFGEGVNRVVWGESLRQAEGM

VGSWVCVGGQEEGEGMGWGKVESLAEDCMRLSLGVISRAGFGREMSWSEEKSAGGREVGEGHD
MSFQDALAILVSSVYLVALLPRIFLRFIPLPSLRRGYQSFVEIKKYMLEFKQQAEEDPEEGGRGSSLLK
AMVNAAATADGDDEEDALKQGASKQSFTQDELFGNAFIMIVAGHETTANTLQYAMINLAMHPK
VQRRLQTELDEAFGSRGQSEWSYEDDLQKLLNGLPGAIMAEQLRVQSPVAGIGKLTLVDQALAVN
GDNHILPKGTLIYLDTTGAHLNPEAWKDVDSNDLGSFNPDRWLKKTADSADKDSERSLYQPEAGA
FIAFSAGARSCIGKRFAQIEFVAVIAGLFREWSVELDVEGQASAKELLSMTEIEKKAVWERARQDVK
RTLKDKMTIYLTMQLENGLNIPLKVVRRGKEMFGDWA*
>CYP6677F1|5185|Grascr1
MHFSLYAGVPVLTFIAWQTLALLKNYKTALSIGLPVLRLPFGLAKWAKYTYLDWQFDDKHAIHDE
LGPAFVLRMPSLNEVIVADPVAAHLALARGEEFVKPSIMYSYLNVFGPNLHTVEGETWQRHRRLIE
TSFDERISASVWTEANFQAGQMLDSWLSRGKQGTHDTVKDTTALALHVQTYAGFGIKYDFKDSNT
KLEAPHRLTYRDALLRVHRNFTLLKVGEAFSECVLYMQEMIHKGKTAINTGPKGEPDNLLSNLVQ
ASHKIFIAGIIDNFEAGLKNGGKLDSDPFFPTKGLYDSELYGNLFIYAMAGHESTANTLATSIAYRAA
EPRRQDWLHEEISQLQLPNTLPPPSPNLSSTPTRPYLETFPLLPRCLAIMHETLRLHGSTVFLPKPTPSS
NPQQHKSEPYNHPPLHLRSNPPPFPLYFRNNKLPSPPYRSRDLGSRRFGLATRSLDKPRCHNGQRK
HKRDERRGICRLGRRATGVSREEVWYGGVCGVYGCAGGEGEGGG*
>CYP51161A1|5870|Grascr1
MALLQALTDSGLLLVQLSLLLWLLQKVFVFVQRRAKRLSLGCKNPKTCPLPDGVALSLASRRRFQR
YALHPGQGDLHKKFGHTFRTTNALSGDTIYSMQPENIRTVWSSGFKEWGVENPRLELLGPLCGKGF
VTTDGELWKKTREALRPHFYGSTADAIDLDLLENHLQRLFLKIPRNGEALNLQPLWHDLAFDMAF
VYLMGEEIKDVPGLENLSTTNIRQQMDYGMVGCRIRYSKGPLKVFHKDAKWQTALKDIREFTSAV
MKRAIERKQALRKQALDERAHTSNDGKEYPESKLLIYTFMRRWPKRNDLSEGLIQYFVGASPAVAG
FFTNAMHLLSLKPEAWNKLKLEASKLSGPSMKEDCKQRRLPYTMSVFNETLRMYPFTTLEARSAKH
DTILPYGGGSDGQDPVLATKGTMFKANIWALHRDVSVFGPEPEAYNPDRWDEIKPDRWQFMPFG
GGPRVCLGRDKARTEG
>CYP578AA1|5984|Grascr1
MLFATIITSAILALLYQYIVYPLFFSSLRRIPSAHPLARFTSLWILYIRFAEWENRTVHAAHVKYGPIVL
LGRNEISVNCVDGGIRNVYAGNFEKDSYYEFFSNFGACNMFSMIAHGPHSVRKRILSHVYSKSFIQT
SPTMAAIERILLYERLLPLLRSRAGKALDAYLLFNTAALDAITGYVFGIRSATSRLSNPEAHQEWMD
KYFMRMPYRYYDQDISHFTSPLKRIGLNFVPKAVYEANDAIENWCHGLCESASEVIEEIRGGKYIKA
ENVPVVYDKLRGALEKERENKDQIENSIPSNGQSSQAPIPLQLEIASEVWDHVAAGFDTSGIFLTYA
AWELSRHPEIQRQLREELLTLSPPIVISSAPSLPRPQDVDSLPLLHAIAMETLRLHPSIPGAQPRSTPYP
SCTLGPYMDIPGGVKINAQAYSLHRNEAAFPKAEEWIPDRWLSKLPGETEKKERWFWAFGSGGRM
CVGSNFAMNSNKHILAAVYSNFRTVIEDDEGIEQSDAYTAPPKGQRLMLRFEGLGQEEK*
>CYP51158A1|6687|Grascr1
MCQYAEITVAIPLAARAIEKNGRGILALGVSKPCCLWCQYYLEELNKFYEHRKFEFVYKGTHGKQP
GGWLMPKEAPPIIANVMKAFVTAKIDQIFIRVGARRAPSAESIELDMRLKEQTQIQTVWKPVTWNS
MGQWDGDRLTSKRFQKQIHGIHIPYLIGRHLSLLRMAVAIPVLTHVLAGVFSLDLKTDENPEGFYDI
ATPYNPARSWNRRRDARKACSRLRGKTKAYLKRHLPHLPDSKPEDVGLWQKIAGFFQGPHFRAS
HIEKGTIRWYGLQVAAKVVAAVGDLDKAADILTTVAFAKSYSYLLSTNAQEQYQKLSTLSLGKGKD
VDNKIEGYMYEAFRLSDGLKAIRVARSDISVDGVKYKPGDVIVIPLGKNGAGANPEGFPDPTASKPD
RKRDSYILFGWGQHECIGKPISNQYVVAMLKAVTGLQKVQLNEGAFRRIDVRLQDYYISPDGSELER
HPSSAKLTFTTYTRDASKLRPSIYTIPPSTIGGERTTAPPPSKSQAPPKSNNGSLIPTVCTHAIRPQDIGR
EMWHVDHHTEYSTEVSISS*
>CYP547M1|6952|Grascr1
MAKLRDVLVALSIAAALETVLLNLFAPHLLLADRFSRSILRFFLINASLFTIWRIWIYPFFFSPLRKLPG
PKSGNILWGHGPAEFIKKPPGSDFLTWSTSIPNGGLLYFRGFFNNDRLLVTSTETLKAVIGDNAYDY
EKPAPVRSFLLQILGAGLILVEGDEHRFQRKHLLPAFQNKQIKDLYPVFWGKGRGLLEAVGEELREG
GAGSGSGFEGGTGGGDGGSGGANATARTGASGVVEFGEWATRVTVDIIGLAGMGRDFNTLKNSD
DVLVQNYNSLLEPSRANALYFASNLVFPQWFIRALPWHKNKDLARTTGNLHRYCIDLVHERQAE
MKSGRAMDKMDILTLLIRSNDFKDEELEELADQMLTFLAAGHETTSSALTWLTYLLSLHPTIQSSLR

HEIRTHLPSPSTFSSSSPSSTSSPTASDIDALPLLNAVCNETLRLYPTVPVTIREAIRPTTIPYRSTPSTSAS
GTTDGAGTGSGASEYGDRLYAPIATGTRILLSPWAINRSPDIWGEDASQFKPERWLGTDGTGDGVA
GKTGGLSGSGSNYGFLTFLHGARSCIGQGFARAELKVLAAAVCGRFEMRLEGGDREVERFLLRRFV
DGLK*
>CYP5042E1|8285|Grascr1
MTTFHMGSQLWVVLNSNQAVTDILLKNPRTTLGRPHMPVADGLISKGKRSVLYKEQQWSESRRM
MRSLLNNQALGTYANYQDIESIKMLKAYLETPKDWHRHHAHYGASILYRSVMGAPFKKSTAEIDE
FQLLIMSFLKDLMSSRVDYFPMLRYLPLQPWRRSWQAKGDHYERVVMRWSSDGAKLAQAGKIQA
SFVRDFELPEKPESQGFDNQVIFLANTIMSAGSDNMRMTMNAFIMASVCFPTPYARARQEIDSTCG
GGGGNDDNDDDGSETSQQSALRLPYLSDVGSMPYLQAFVMEVLRWRPVVPIGIQRELGEALSYKG
YRFPPGTNFLINTVTCCKGVEDPDDFQPERWMDATKPPEQFFFFGAGRRNCVGYKFAEQGLIIALSR
LIYCFDFAPVILCEEKQAGPIDSENLNPWTLGEPFPIKATCRSRAHAQLIKSLLL*
>CYP613T1|8614|Grascr1
MRQYPIHHNATRDPLPTCPYTFPNGQGNIAKFPSGLSNSTLWSQQHGSVYRIWCGTKPEIVLTRAA
HVKAVFRDSDRHTKAVNNNSGYFMQQLLGQCLGLISGAEWADVKDVVHAPFARAAMEGYEGFV
VQEVESFFARAYEGKKEKRIAADGKSEGDLMLPFFIIARILYGELTPDVEAQLQQLAVEREALFHHVI
QGGITRFHLSRLLPTKANRELGAFKHKWRAFNMSVVRQCSGQQSEKQPVPPIVPMYAEVERGRITE
EQLLQTLDEILFANLDVTMGAVGWTLVFLATHSRLQEKIRTEMHEWPQQRRQEGGSGTHKYLLST
STLLHASILEAARLKPLAAFSVPQAAPTERVVDGYAIPRRTDFVIDSHLLNWDVEFWGKDADVYRP
ERFLEKKGTELRYHYWRFGFGPRQCLGKYVADLMLHVLVAQMVENYRLENETEREKWTRIEKTWI
SHPKLDLKWERR*
>CYP6839C1|8645|Grascr1
MLAVRRLEKKHGYERVARYPHTDWLLGSDLYRLRKQALKHGRLMKLYESHFAMCGKTFEENFLG
VRVINTMDSANIQKVAALSFQDYCKGFTRKGVTSPFMGKGITSSDGMEWKHSRSLVKPTFSKSELSD
TEGLGQFVDRFLGLIPRNGETVDLQPLLHRLFLDISTEFLFGQPMNSLDPEALFDFTKFLQALDEALA
GVGKRRQAGRLRFRYYFDKSWKRAYDTIHSYINEHVQRALHTTQDKAPPNTKYSTKWQKKFIDLC
KTSATLRETPLTLETLRLQGPSGRIFRQSVHEIILPHGSGIDGRSPVLVPKGTAVALNLWCLHHDPDI
WGEDVHVFKPERWNEKRLSWEFVPFWGGLRICPAQQQVLTHAVYVLVRLTQEFARIECRDDSGQ
DYVELVKMTTQSRNGVKVALYRDKMKF*
>CYP6001C41|9786|Grascr1
QRGKWAEVFTRSEQYRRRTAAAAAARQWSEPDHSVTRGDVESSFEQFAQLIHSSARPLPTQTGDGS
YIEHEASSSLFQDLRHLGFKDVKTLVQTVSNKASGALTDDKTMLMEHIIQLVAGLPQNSKSRMDLT
NAFLNELWVNLDHPPGSYLGDKYQYRQPDGSYNNIHMPMLGAANTPYARSVRPETMQPGSLPDP
GLIFDSIFARTEYKEHPNRVSSILYYWASLIIHDLFQTNHRDMNQSLTSSYLDLSTLYGDTEEDQGWIR
TWKDGKIKADCFMEERVLGMPPGAGVILIMFNRFHNYVVEQLAAINENGRFSKPKDGLSAEASEK
AWAKYDHDLFNVGRLITCGLYMNITLLDYLRTIVNLNRSNTTWTLDPRVEMDKRTTKPEVYCPRG
TGNQVSCEFNLVYRWHSSISKKDEQWTEAVYNKVFGKRASEISLPELLKGLGQWEASMPKDPQKR
EFGGLKRNADGSFNDEDLVNILADAIEDVAGCPGALNVPTALKAAEILGINQARHWNCASLNEFR
KFFGLKPHQTFEDINSDPRVSEQLRHLYEHPDFVELYPGIVAEDHKMPMVPGVGICPTYTVSRAILS
DAVVLVRSDRFYTTDYHPKNLTSWGYSTVQYDLNIEQGCCFYKLFLRAFPNHFKQNSIYAHYPMTV
PSENRKIMQSLGREGDYDYKRPTFIPERIVLTSYKAAKYMLERSKEFNVLWGEATAFVMGPRAWDF
MLSGDNALHRQQKKTMANALYHSNWEKQVQAFYEYITLRLLHENSYKIAGVNQVDMTRDVTNL
AHCHFAANVFSLPMKSKENPKGILTEHELWSLIALLFTAIFFDFDPPKSFPLRHAARALAKQLGKLIE
INVKATNATGFISGIVDSFRENHNALSEYGVHMVRKLLESGLGVEEITYSQILPVATAMIPNQSVVFT
QLLDFYLSDAGKQHLPAIQKLAQQDTPEAFDKLMHYAMEGIRLHGTFASYRTSTISTTIDDDGKQV
PVSPGDRIFCSFVSASRDPEQYPEPDKVRTDRPLESYIHYGIGDHTCLGRDASRVALTAMLKTIAKLK
NLRRAPGPQGQLKTIPRAGGFYVYMTENHGSYFPFPTTWKLQFDGEVPPLKE*
>CYP51234A1|10738|Grascr1
MAMNPIIIAILATLAISLALSWRKSNALAKKSNMPVFWSPLAQLWSLWHPSHTPFHLAHSNTFLLT
HPLSLPSLWTCDPKICTALMTSYPTSQLVPDMIEFFNLYGPTVGSVEHEEWKAYRRIVTAGFNADTH
AAVWKETLGQAREMVGVWERDGDGNGEVKGLMKGWCCRLTLHVISRVFFGRGLRWKEEGKEE

GDGGEEMPKGHRMRYEEALFMVLKRLGMLYVVPHVLLKRVPLRRFREAWVAFEEWTKYMKEMV
RETREKVEAEIEMDTKSQKKGGKKGNKRSLLGTTACISLCLTPWHNILDNTTTTLLNPTNTTSAESM
ILASKSPSPSSSTSSSSKSPLPPISESALLGNTFFLLLAGHETTGNTLSFLLLLLALHPAHQSSIHNDLLA
LLSVRPLSAWTPDNEYTTLTRNKSNLANFIKETLRVYCVVQFIARRVVKDMPVVDAQGMRRIIPAG
TVAMLDFSAALRNPELWYGGNRQGDGVAANLVNEVLDSPAATEFDPDRWLEDDIDTDTNGKGG
EGRGGGEGGRRAKAKAMEEAFFPFAQGPRVCPGKQFAMVEMVAAVVAVLKEWRLELVVGKDVL
RKCGGDGQKAWEEVRREGFRKLKRGVKSNITLEIGEVVPIKAVRR*
>CYP51184A1|11240|Grascr1
MLPFYVILLSGLAALFGYLVLSKQRYRANQLTHGCSPPRQYPHKDPTGLGIDLFLDTGKITTFEVSTL
STPSFCSCEPANLQSVFVTNAKSWGVSYRLLALAPYYFAHYLDLMLRKIPRNGEPVDLQNLVFSLYL
DTATLWLFGESFESLSGKGDKADEFTRTFAYSLGVGGFRMAIGPLQFLHRSRNWEESNRTNQAFIEQ
HVDRAIERQSKGDNEKQGNKRTAVLDLMAQQLHDRTQLRNESMHAFIAAHETTACLISNLFWVL
ARHPTVWARLRSEALNAFGSTSSPYLNFEASLKLDYLRKVINETLRLYPVFPYHVRIALADTILPNPP
PIPRLPLPRPHRAGRHYPPYRRRPGRCTARSSIWGTDAGVFHPERWDDFTPNPNQFGPFGGGPRAC
AGRQKALMETTYAGHNEAYGGQTDPFTHLTQVNTTLLKNALAAITNSCPHLQLWTLQTGGTNYG
FKLLDQPSFLETATYSPPHKESNLPLPPP*
>CYP584AR1|11535|Grascr1
MFLFSLPWKFQLPLYPVVLWLLLRLNYSIRVAPARRRKALALGCLPARRCLRRFLGLEEVYLQYRYV
KEHKLLFNGHRELSEYGGTFSVKVMGTDVFSTNEPANVQHMLATDFANWGVAILRAPMVPLLGE
GIFSTDGPKWRHSRDLLRPNFNRAQIAEVSMFERHLENLVKCINRERRGETVDLQPLFLRLAMDIST
EFLFGESTNDLAAESSKPKITQFVESWDRCTKVLGGGANWKSIANVFWKDKQFANDCKIVHNFAD
SIIEDARTRQSEEKSNTGVESSSRYLFLNELVEQISDPVKVRAELLNILIAGRETTASVLSNSFFELARRP
DIQAKLRAEITGLNDLEFETIKNLRYLRAFINESLRLYPIVPMNARQAVADTTILVGGGDDQKSPVFV
PKGGLVSWNLYSMHRRKEIFGKDAEEFNPERWLDEGEQKGIRPGWAFLPFNGGPRICIGQQFALAE
ISYILIRLLQEYSSIESRDSEPWRESMQLTCVNFGGCKAALTPRS*
>CYP52AA12|12097|Grascr1
MQTQYLMAVWALVAYVLYRLITSIITERRYVAQAKNLGCEDAPTMPNNVPWGLEHIMAARGADK
RKQFPEWLQSRYNRMGNRTFQHFVLGNQAYTTNDPKNIQAMLATQFKDFGLGERRQGTFKPLLG
YGIFTTDGKFWEHSRAMMRPQFNRDQVSDLELEEQHVQNLMRALPTTSLSSSNAPTAGRWTGPVD
LQVLFFRLTLDSACEFLFGESTDSQILNLPENAQQQIQQQQSHSKTTAATDEKTFAGAFDRGQNWL
SRRSRLMNLYWLVDGLQERRDTKAVHDFVDHFVQLALKHQATLSGSKTNSTDDPSLEKGPGQPYT
PRRNSSGKEKYVFLEVLAAQTQDPLELRSQLLNILLAGRDTTASLLGWVFWLLARHPDILAKLRSAI
LESFGPYSPSNSAEITYTSLKSCTYLQHVLNETLRLHPVVPVNMRQALRDTTLPRGGGVDGSKPVLIR
KGQCVDYSVHVMHRLEGLWGDDAASFRPERWVGRKPGWEYLPFNGGPRICLGQQFALTEASYVI
VRLLQRFDGIQNLDGSVEATHNLTLTDCPSKGVQVRLREADKVEA*
>CYP584J7|1995|Grascr1
MVNILQSALGAAGFYLLYCIYWELTVGASRRKLAREHGCKPIKKFPDWEPFYGLRGFVQNQRWFK
EHTFMESVRQERFKKLDTNTFSMKQLGLKVVATIEPENLKTIQALDFKKWSLGQRRITAFTPFLGPGI
FTTDGQQWAHSREMLRPNFNRSQVGDLATFEKHISHLIQAIPRDGSTVDLQDLFFRLTIDSATEFLF
GESTNCLAPGTTTVSSNAFATAFNRGQESILERARWGKLGGLMMNHEELHKNTKYVHEFVDNFVS
KAITRRNLGKTETSRYVFADELVTRTSDKFQIRSELLNILLAGRDTTASLLSNLFHMLSRRPDILANIQ
AEIAPLNGNPPDFAQLKEMKYLRAFLNESLRLYPVVPGNSRQALEDTVLPKGGGEDGQSPMFIEKG
TMVSWSLWTMHRRKDYFGEDAEEFKPERWLGEKGLRPGWEYLPFNGGARICLGQNFALNEASYT
TVRILQNFKAVESRDDRPWTEWLTLTCTGLYGCKVGMVPV*
>CYP584AT1|2528|Grascr1
MQLFQSHFQEKPFICTLIAACLSAILYFIYRSLRRHIEVSSFKKQHGCYDPPRYPHKDRIWGSDLVKER
ATALKEGKYFTLYESQFERYGRTFEEFCRRQKTINTIEPVNIRHVLALACDDYMKDPARVRAQAPF
MGPSVFSDGPIWKPSRALVKPIFARAELSDMDHLAMFTDRFIELLPSDGSMVDVQPLLHKLFFDVAT
DFIFGEPLGALQPSMPASSADFIQSFKKALMWASKRREAGWLSWRYNHNKEWIDSYTKVHRWVD
RQVARALKETEKIVDDIDGDTKASQPVQRRYVLLNELAKRIRDPIDLRYQVLGVFLPAVGGTANAV
GNVLFQLARHPEMWKKLRQDALKLGDEPLTFEKLKSLSQFRYVVLETIRLIGPAARVVRYAKYDTT

LPSGGGPDRKSPIFVAKGTSVSMSTWGVQHDADIWEEPQEWKPERWRNRKIGWEFVPFSGGPRICP
AQQQVLTHTVYLLVRLVKRFEGIENWDGVREFVQRVTVTVESGNGVKVGFKPAISESRGS*
>CYP52P14|4646|Grascr1
KELLAQGAVLTPHKRLSYRWPFALDLLLEASRADQSGGILNFFLAIVGRTGNTFEQILLGFRGIDTQ
DPENIEAILGTQFTNFSLGSRSDNFRPLLGHGIFLITSGLFSAMFMQTRSKSFSTIQNEIEKFVASVKSQ
TSKIVDLQPLFFRLTLDTTMAVLFGRSLEDLQAQGAGDESVFAQAFDYAQHRLAVRGRLGDLYWLI
GGRKFRESCKLVHDFVDEIVIRALEQSSKTCSATSVDSGDRYVFLDALISKTKDRSVLRDQLINVLLA
GRDTTACLLSWTFKFLAQHTDLQDQLRRECRELPSFTQGDLPTPAEIKGMRLLQNVLSEVLRLYPSV
PVNSRSAIKTTSLPIGGGLDGKSPLIVRKGEAVGYCVYTMHRRKDIYGEDALEFRPSRWDEDRMKNL
GWAYLPFNNGPRICPGQEFALLEASYTVIRLLQTFEKVERPADEPALQSHTLTLVVASANG
>CYP584AX1|5875|Grascr1
MDLRGATLLLFCIAVLVKVGLHVRTKAEQRRKARSWNCMQPVYLPQDDPILGLDLVKKRNAAIK
AHRYYLSNKLNHEKFGHTYGARPMCTTIISTIDLDNLRFMYGTNAFDWGIEPVRLFAFEPIAAWGI
MTSDGHYWKTTRTLMKPLFSNGQPTDLPALERHVKKAITLIPRNGETIELHEVMNYLAFDSGTEM
MFGKAMDSLDPAAVETRDLLKCFSRAKTEVFNRMMRPKVNALTPKPFWGWCQRAREYIDQETKR
LWENRLDSKTKDRHVLGADLARRVSSFEDLRAQVTNLMFSAYDGTGASANNVFFHLARHPNVVK
KLRQEILSLGPVELTAERLRDMEYLQWVINEAYRLTPPVAMGARTAVRDTYIPRGGGSDGSSPIFIAK
GQTVFTPFFALTRRKDIFGEDADSFRPERWDKLDLPRWSFIPFGGGPRVCLGQQMVMAETAYIIVRF
LQEFQSIENRDPVIDFVEHHQVTLISKNGVKAALIPA*
>CYP584AU1|13055|Grascr1
MALLLRCTIGAAACGLLLWIYQQVGVYLAFVSKRRETGCFQARTYPHRDGIFGYDLYKQRKRALEE
GNMQALYTREFDQLGKVFRENFLRQPVINNMDALNHQYIHALGWKDFGKPLIRAKANGPILGNG
IFAAEGSEWKQSRDIIKPIFAKAEVGNMAMMAKHVDHFLDLLPRDSGTFDIQIMFKKLFLDLSTEFL
FGESIDAQTPKGAPKADGFLKALDEALLGWNKRRQAGILMILHSFDRRWPRACQRLYNYFDKHVY
RALETTSTVDLTREGGKEANEDEGKARVDPSRKYILLYEMAKQVRDPVQLRFELLNVFIPSHGTTAA
LLGNVFFLLARHTHHWTRLRKHALSLPFDPADPSALDFARLKSLLPFRYVIQETLRVLGPAGRVFRA
ARYNTTLPRGGGPDGLSPVFIPKGAVVMSSTYHIQHDRDIWGEDADEFRPERWEEPHNRGPWDFV
PFLGGPRICPAQQQVLVQAIYLLLRMVREFESIENRDNMLEYVEQQRMAIESRRGVQIALRPG*

Umbilicaria pustulata
>CYP5087A4|99251|Umbpus1
MLAAFLAAFSILTVPITVKVISLCSKEKQLSKFDGCQDPPCERPYDYFGVVNIIAATRHLLNKTALAN
TVDRFRTYGETYVTRFLDQRIFLTCDPRNIKHVLVSRFIDYDSSTVRVHLFLPITEHGIFAVDGPEWKR
ARNVYRNVFSRTRSIIDLNVQERQFQRFLHRIPPTGEPFDLQALFLKLTLDLTTAFALGESVDSLSPSQ
SDEKKQMVQSLLYAKKIIARDGFLGPLRLLFGRRDFYRACSDVHRYVERRIEGVIEEKRQQGEDGGL
EKHLKGFNLLQALADNTDNVLELRDGVISIMIAGIDSVASLLSATFWLLARDERVFAKLRASVLDSF
GQELPNYDQLKGLTYLRHVFQEAMRVYPPVPFNARIANKDSTLLYGGGGDGNSSVLVKKGDKVIF
SSWASHRSLKTFGEDSHEFRPERWEHLTAEELMGYIPFNSGPRACPGQQYAMMEASYAAVRILQNF
SEVKNRDPRGWTEHIALSLSNGNGVLVELVRKSEVQNVT*
>CYP6677D1|99316|Umbpus1
MSPMWALSNRRLRPWLQQLPWGLSDLFRYNYIGWGFDDKYNAHKKLGDLFVHVTPNEIEVYVA
DAGAADAITSRRKDFPKPTVMYKPLEVFGPNVDTVEGREWQRHRKITTRPFNERNSNLVWVESLR
QAQGALQWWTEQPGLGANTTAEDAKSMALNVLTCAGFGKPYPFASRLQRTDENQLDRDMTYQ
DALSIVLENLLFLFLIPHRLLSFSFLPKNLRTLGKAIQVFKNHMVKMLHRERGLMSKHDPGADNLVS
ALIRASEEVQQNKNVDEPHQGLSDDEIFGNIFIYNLAGHETTANTLAYSILLLAAYPEYQDWLAAEI
NKVLEGQGETETWKYEEAFPRLKRSMAVMYETLRLYGPVTAIPKCTTNTQQLSIHAKDYTFPSNTYI
YINTSALHTHPRYWGSDSLTWRPKRWITPNPPSNPPTQSSLAAETIADPIKGSFMPWSEGARVCPGR
KFSQVEFIAVLAVLFRRHRTTSLENGRRLEGGDATADDAAYEYFVSMDD*
>CYP5105E1|99411|Umbpus1
MQLAIPVYEWIEACNDRAFLEQSSARAPPSPFMSLPLEIHRMIFSYLGDYADVFCLSLVDLYFWRIGR
AVLMYEVLSVLCPLANRALICVGDETSTEPPDYPAGLLTPDEVEELQAGLDSDDDDGEDDESCTYN

RPENLYHLAAARYNYIARYIFSTFPELYFSPIAKIPGPKLAALTSWYEFYYDVIKPGQFVWHIKDLHE
QYGPILRITPWEIHINDPDFLDEIYAPSFCNREKYSFQTRTLKVPMSMGGTIKHDLHRKRREALDPFF
SKKSVTVLESMIRHKVNQLCELMEDHSKQEIVVSLSDVYFAFANDVVSHYSFGHDNNLLGNEIKSST
QRNNISRLLLEVKVINVFHGSLMRALNMVAFSDNIRSEIQQALSDKDRVNDGEKRSIFYELRDNPTL
PQSEKELSRLEQEGTLLVMAGTESTAKSMAIAHFHLLSNPEDMSKLRAELQTVPKTASWTQLEQLT
YLNGVIAEGNRLPFGVMGRVCRIAPNEALKYKGHVIPPGTPPERWTGSEGVARRKYQMVFNKGGR
NCIGMNLAHAEMFLALAAMARFDMTLSETDFSDVEFQHDFHVAYPKLDSKGVRARVQAKAVEA*
>CYP5192G1 |99775|Umbpus1
MMDNASPLSVATLIFIATLGVVFVVKLVRVRLFYRSLPGPPHNFFLGHLPAVARVATTLPRDAWDW
TIMTYLHQEMKLGDFFYLDTWPFGPPMVFITSPELAAEIDSAAHYTRHPYVTNIVGPIVGSHSILTAS
GAPWKFMRSMFNPGFAHKNLTLLIEDIVEETTVFRNKLSRLAEQRELFEMEDIATLLTFDVIGRAVL
DEKFCSQTSANELVVAFRKQLSWLADTNPVNVLDKLNFLRPVMNWWYHRKMEIYLRNQLAARK
PNPDSKKKYVIDLALDTYRRDKKQDSLESGFMKDSNFTDVAIGNMKAFLGGGHDTTSSTICYIYYLL
SKNLAALQRVREEHDTVLGKDLDKIAELLKSQPHLLEQLPYTTAVIKEVLRLYPIGGSGKMGADNIV
LTRDGKSYPTTGMLLVTLNPAMSRRPDIYDNPEVFNPERFLAPSSVGKESWRPFGRGAMRCIGQEL
AMMEMRIVMVMTLRNFDVADAYEEIDRMKGKTNTIKEAFGDRAYQIGRVIGKANLGMPMRVAK
RDISA*
>CYP5792B1|99987|Umbpus1
MPHLCSPPRSVQTAILQSPTGTLTIASAPTPSPSPTQVLVRTHAVALNPTDHKMPQLHPSPGALTGC
DFAGAILSLGASVGALRPDLQIGDRLLRLAGYTPLATCSPASAALVRDYGASHTFAYTSPSCADDIR
AAVAAPLRYALDCITDAESARTCYAALGRTGGRYVSLEACRSEWKTRKAVKGEFVMGYEIFGKEV
ALGGEYGRRADEGKKERAVKWCREVQGLVKEGKIRSHPATIAQLSPQQWLSYGAVITISYAISRCIY
LLYFHPLAKFPGPKVAAVSNLWYAYHWFSGRYPWTIEKVLEQYGDVVRIAPNEVVFMRPQAAVDI
LMSGTKQRPTFIKTDFQHIGGEHAGIAADPDVEKHRAVRKMLAPAFNPRALKEQEPALHEHIDRFI
RQLEKLGTGEQGVDMREWFDWLACDIAGDMGYGHNFNNVRDSKCNPWTAVPSVCQCIAAKAH
LFLRTFRGIGLWGTLNQVMKRFPLFYPLVFLVLPPKLAATLPTLLKANLTIVRDRITNRHSLKHPDYF
SLLIQDNEPVPSEGFLVAQANHLIVGGFDPDTNLFTAAVHFLLTNPEKMEKLNREIRTTFASYEDIR
NDALQSLPYLHAVIEETLRLHTNGAFGLPRISPGDTVDGHYIPAGCVVQTAAFATTHSARYFHHPR
SFHPERWLPAEHAEYDPVFNADVKSAFKPFSMGPRGCVGQNMGYMQARILFAKMVYRFDWELV
DRGVDWERDLRLYAIWEKPPVVVRYRVVGTAKE*
>CYP5128B7|100047|Umbpus1
MGDETTTVHRLHKAYGPVVRVAPNDVDISDGEAIAPIYVDKGGFAKSACYVNYDIDGHASIFSTLS
SAYRAPRAKAVVGIFSTASIRSGSDRIYECVDKFVSRLQAEASTGRPVNVLNLSRAVAIDAVSSFLFH
KSYYGIEEKSSQLSVSPFVDAFVAVGRFFYLPNGVLLLLEWFIATFMPDKHVNESFIAVDGFISQLVDS
AAKDTETYPGRLLDRGISKSETIAQCKDVIFAGTDANGLNLATICWYLSWHSERYAILRKEIMENQA
TQADIQTLPYLKGVIKEGLRLAMANPSRHPRVVPESGWNFRGFHFPAGTNVGVAAFEPHQDPNSF
PNPERFLPERWLNPTPEMQRDWVPFGNGPRACIARNLATAELFMAVEKLVQADVLKGARPVKDR
IEIYEWFNSKVKDGRIELVWPAGVE*
>CYP6001A26|100363|Umbpus1
MAEKDSLPNAPQEHTSTDQEIEDFFASISGVIKAASRPLPTQTGDGTYITPPSTNGVFDDILHTRFKD
WKTLLDAGLSEMTGGPVNDKTYLMERIVQCASSRKSNSKIGTHLSDQFIDTLWDDLPHPPMSYLGD
QYAYREADGSNNNYLFPKLGAANTPYAKNVRPQTLMPANLPEAGVLFDTVMVRKRFKPHPNEISS
MLFYLASIIIHDLFRTSHEDSNISKTSSYLDLAPLYGSNQHDQNLMRTLKGGKIKADCFSEKRLLGFP
PGVGVLLIMFNRFHNYVVEQLALINEAGRFTKPKEGLPDEQTKKAWAKYDNDLFQTGRLITCGLY
VNVILKDYVRTILGLTDPKKPWDLDPRIQVKKKGAAQGTGNQVSAEFNLIYRWHAAISERDDKWT
NEAYSDMFPGKDPKTLTLPELLQGLGKWEQGLPEDPQQRPFAKLHRGPDNTLDDAALVEILTASV
KDVAGSFGANNVPEILRAVEILGIKQGRSWNLATLNELRSFFNLAKHETFESINSDKDVVEQLKHLY
DTPDHVELYPGVVIEEAKQPMIGSGLCASFTTARAILSDATALVRGDRFYTIDYTPKNLTSWGYNEA
SSDTAVDGGHVLYKLFLRAFPNYFQPNSVYAHFPFHTPSKNQEILTKLEIDNEYNFDPPALIPPLVFI
NSHKAARDILSNQKDFKVTWGTAIKYLMHNDGKEYGSDFMLAGDDPPNAKSRGLMDKALYRDK
WHDEIQKFYEEITLKLLHQNSYKLAGVNQVDIVRDIGNLAQVHFSADVFNFPLKTEEYPTRLFSEQE

LYLIMAVVFICIFFDADPAKSFPLRQAARNVTKKLGETIEAMVGPMAPAHISGFFASRFHKHSPLSQ
YGMHMVQELLKSGLGVKDVVWSQILPTAGGMVANQAQLFAQTLDYYLSEEAEPHLKEINRLAKL
DTPEADETILRYFLEGSRMRATVGLYRDVANAIPIEDGSKTLNLKPGQRVMVNCIAASMDPDAFPE
PEKVDLKRPLDSYIHYGMGPHSCLGYEASKVAMMTMLKTVGKLDRLRRAPGKQGEMKKVVINGF
TMYMTADHSSYFPFPTTMKVQWDGELPPLKN*
>CYP5328J1|100378|Umbpus1
MGDIWMQITPANNWIYVANPEAVVEIFNRRHEFRRPLQLYGMLDIFGANVATTEGSAWQIQRKIT
ATPFNEYNNNRVWVESLRQSKDMLQWWLSHHTSGVRSTAKDTRALSLNILAYVGFGRSYPFHGAF
SMSETSYAVSFRDSLSVVLDNALLTLAIPPSILRLPIFPKSWSRVGWAIKTLKEHMMTWFLDEQSRFS
QGKRGTGTLMSSMVDASETITRDPAHTSPKHESGQLQHRKEGITVDEIFGNIFVYYFAGHDTTASVF
TYAVLLLAANPECQDWIAEELQHVLPTEDNEAWGYESKFPRLKRCLAVLLETIRLYDPIPGLPKWT
GEGERLLQVGDQSLVIPPRTMVLPSLMALHTLPRYWGEDSLVWRPSRWIIPAASLETEGPVDLGTQL
EKESILVPVKGTYFPWSEGQRNCPGKKFAQVEFVAVMANLFRDHCAQPIPMLGESQDHARQRLFD
VVNDSQLRLLLQVRDPSSVPISWARRQTAGLDYCRDTVE*
>CYP59W1|100377|Umbpus1
MSKPILHVNGRVLLNTLLFLLFSAIATFIFRLSQIRRKMKKLKDQGLSITPYHSVFGSLLVTNDVVSQL
PADVNPQYYPHEIRRAHPKLGSIYYLDNWPYIAPILVVASPSTTSQATQEHSLPKFPALANFMRGIA
GTRDLVSMEGQMWKTWRGMFNPGFSGSHLTSLMPEFVKEVTIFCDILREQADEGGMFPMKNVTD
NLTIDIIGKIVLSTNFGAQRKYVPMVAALRRQVPWLSFGTELNLWQRWHPLRPLMYWYNQRVMN
KYVNKEVDARFAQQKAKIDGSKSITDLALKSYLQSDTQDANASTADSIDPTFKILLIGQIKLFLFSGH
DTTSASICYVFHLLSQHESALQKLRDEHDAIFTTDLSAAASLLSESPHLINQLPYTLAVIKETVRLFPV
ASSTRAGQPGFSIVSEDGTSYPTDGCLVWSIAQAIQRDPTYWPSPDDFIPERWLAAPGDPLYPVKGA
WRPFEYGPRNCIGQELAMLEMKVAIVMTAREFDIKTVYDEWDRLHRPKGPKTVSGERAYQAKSGG
PSDGLPCRVKVAVRAN*
>CYP617AD1|100379|Umbpus1
MANTSEYAVTAVLSLAAVAGFVFLLRCQATKHDPREPPLIPQPVFWIGHLLGLIRKGSRYYANVSA
RYPLPIYTLKVPRSKLYVVNSPELVAAVERNSKTISFAPYVLQFAKRVVLPSREAEDALSQNLNGEAG
KWGCRIETHSAMHEAMAPGDGLDHMSSTMLHSLFGLLDSKTASTGPRRFNLFMWIRHIVTLATTN
AAYGPSNPFRDPAMEDAIWTLNDKFLFIAFNVYPQLTAWKAHRAREKLFRGFRDYFAVKGHEHGS
RLIQGRYEVNKKYVSVEDIGRFEVSVSIALLVNTAPTAFWTITSIYSDPLLLTSLRDKLSAFVSASPSPV
TPNKTVYRLDITEVIRGCPLLVSLVQEVLRMQSTNAAGRMVLEDTLLDGRYLLRKGSTVLIPAAQLH
NDGSIWGESVGEFKPSRFIKQAGRHRETRIPASAFRAFGGGTALCPGRFFATTEILSLVAMLVLRYDI
APVGGKWNVPKGRSHIITSVLSPEQDVEVTVTEREGYEHVVWDFVLTESVSRPEPLL*
>CYP5077C3|100420|Umbpus1
MATFPAITIFDKLWAPSLALTLSLAIFSLGRIWIKQSHISGPILASISNFPRLSWAYSSKAHIVHIKLHEQ
HGDLVRIGPNCISVGDPREISKIYGIAANFKKSDFYKVLQPMSKGHVIQGLFNTQDDNLHRAMKKPI
AGIYSMSNLIEFEPYVDSTIAFFFKRLEEVQSKSRQSCDLGTWLQWFAFDVMGEITFSRRLGFLDEAK
DVDGIMASIWKLFQYSSWVGQMSWLDKVWTKNTFLSRMLPAKTSPVVAFAVARARERTEKTGDE
EKSAILQNSRDFMSRFIEARAKDPSIPEWFLTAWATSNVLAGSDTTAIMLRAIIYFLITNPGSLKKLM
AELAQAQHQNRLSDIVTWKESRSLDYLDACVKEAGRLHPAIGLTMERVFGIGHRTCIGKNISYLEIF
KLIPTMFARYDMELADPTREWDLSNHWLVVQTGLEVKLRQRQAYQNGTLSGHR*
>CYP577A28|100791|Umbpus1
MKCNDVQTISIAIPSWFAALVIAAISFTAYQFLYVPLVLSPLNRIPGPKSFAITRWRLMYEDWKGTRT
TKIHKLHLQYGSAVRVSPNEVSFNSLAALRTIYGAGSRFERTEFYRMFDVYGRQNLFTFSSSIKHAAR
KKLLTNAYSKSRILKHPTATAVQDKTRQYLQLLELDPDTASEIFTSLHYFSFDVITNFLYGNDYGGTS
ALTGSVADRALLQDMVDPARRVLSWWAVHFPRYTKWLYTRTGLMDRVLTRAGVLPMRKPTVYS
GIRAHALDAWVSFKDAAIKESNTSAQDSILGMLWKHHESQKEDGLEDMDIASECADQLLAGIDTT
SHTLMFLIWALSLPENRAYQEKLISEVSMIDASLLDRNGIPSVQATDTLPYLGALLKETLRLYVTLPA
SEPRSLPVDSVIDGYHIPARVIVGISPYCLHQNAQVFHEPLRFNPERWFGPASDVEEMKRWFWAFSS
GGRMCIGLHLAMAEMTTLMAAVYRNYSTTIKPGYEGVSPGVTSRFEVFFDETFKQIESSSTLRRLAK
DHKSLHADLPPNFLFSPSSASESVPDDLTRLTILLAGPQGTPYSRGVWKLQIKIPDDYPRSPPKAAFRT

RIWHPNVEESTGSVCVETLKRDWDPKLTLRDVLITISCLLIQPNPDSALNSAAGQLLQEDYEAYARQ
AKLMTSIHAMIPTELRDAVVEAKKRGEEAGTEIREDEEQELKTTRHAFSSSLNSRQKLLIPFISMFA*
>CYP53A62|100904|Umbpus1
MFLSFFFNPYTLLLLPLLYYLLPYIRNWAIRDIPAPFPAAFTNLWLLYQCRRGRRYLAVDNAHKKYG
TLVRIQPDHVSVADADAIPIIYGHGNGFLKSHYYDAFVSIQRGLFNTRDRAEHTRKRKTISHTFSTKSI
GQFEQYMHYNLQELVKQWDRLSSTASSAGQEYAAIDSLHWFNYLAFDIIGDLAFGAPFGMLPKGK
DIAEVRKTPSSPPTYASAIEVLNRRGEVSGTLGCLPTLKPYAKYLPDRFFRDGLEAVENLAGIAVARV
NERLRQTEEGQGANQRVDLLARLMEGRDEKGNKLGQQELTAEALTQLIAGSDTTSNTSCALFFWV
LKTPGVMEMLQRELDGAIPEAVTIPHYEMVKDLPYLDHVISETLRIHSTSSLGLPRLVPPGPGITLSN
HHFPAGTVLSVPAYTIHHSPSIWNPPHSPLSGPEFHPDVFNPDRWDEALLTEKQKIAFIPFSYGPRAC
VGRNVAEMELALIVATVARRYAFELREAELRTREGFLRKPVRCRVGMRRRR*
>CYP5076M1|100984|Umbpus1
MVSWLSNGLLAAAGIATHLGYFNRGEHHLYSTIYLQIFFAVFATALAFAVHVDGEPLAQAIAKVAS
SAGFYLGGLYTSLLVYRTVFHPLNKFPGPFGFRISNLWFSLQLGNGDGHKKLLKLHEKYGDFLRTGS
SDLSIANPNAIAVVYGTKSKCTRAAWYDNLYPMVSLQTVRQKNVHSRRRRLWSTAFSDKALRGYE
TRIMGYQDRLLAQLATIGRQPVNVTKWFNLYSFDIMGDLAFATSFNMLESTEEHFAVKVLDKGLEP
VGWMLPTWMFRLLVAIPGLANDFVRFVKYCNQRLDERMKMTVNTPDVMATLLGPWKEKTPTGT
GLSDLQGDCQLIIVAGSDTTSATLTHLFYELAKNPDHVLKLRDELASHRASGSDLSHQQIKNLDHL
NAVINETLRLYPVAPMGLARLTPPEGIEIEETYIPGNMTVSCPQYVIGRSENIYADAKAFVPERWYSK
PEMIKDKSAFAPFSTGTYDCIGKQLALLSLRTVIAKLIMDFDVSFAPGDNGEYFAKNTTQHFTWGLA
ELNLVLTKR*
>CYP52K10|101154|Umbpus1
MELFLFHFRQTGYTLEQVFLGTPAFGTVDPANLEAILSTNLKEWGMGLRRAITFPMLGDGIFTQEG
APWKHSREMLRPHFQHRQYDDLEVFRESVDNLIDSIKGGVIDLQPLFFRLTLDTTTAFLFGESVNSL
KAPKTVGEQNFTSAFNTAQEYVAKRFRLLGLYWFIGGKKFQEACDNVHRFADQIIDCNLGRGSKD
EESFLDNVAKNTPNRDALRGQIINILTAGRDTTACLLSWTFFMLVRHPNVLGKLRTEISSVSQGRGS
ELNRTDLRNMKYLQNVLKEILRLYPSVPVNSRTALKTTVLPTGGGPDRRSPVLIPKGSAVAYSVYTM
HRRPDLYGMDAELFRPERWDEDMPLNQNETNAKWGYLPFNGGPRVCLGMDFALTEAAYTVVRL
IQRYPTIKLPEGMVPELVGVERQTMTLVVSVTGGCKIELGGEDPIVDVVAVHGLNGDAFKTWTTNK
TKKFWLGDSNMLPAHMKRSRILTFSYNAAVTALFGKTSSDRIMQHAHTLVAELVADRELENAAER
PIIFICYSLGGIIVKRALAYSASRTSKLVQHLHSIFVSTYAVLFLGTPHAGSSKASLASTGRKIIDALVPS
KIWDTEGQLLDALQEGSETLQNITDMFAPLMKNFRIYFFWEQEKTDLGTSKDYVVEESSAAPILDDT
ERAGLPYDHRTMCKFESNRSPGYRVIVAAMIRYSKGAPDVIAPRWVAVKEMLKAKRMNEAAELM
Q*
>CYP540G3|101281|Umbpus1
MLKEFFIGSPSTGRCVQINVSAAETLQELRSGIANAFSILPKDSISFENTQRQLSTLESVIKSNEPVQVR
VDEHHVREPLGPKALPLVGNHYELYPDHVGNHERLFARYGPVIKTVNMGTTIYLTNDPNVSRYVL
SETEYFTKTTTDPQHPLHYMRDNTALFTCDSDAPVFKVAHKFVPPSMSPKAVRHYSPIMQEAVERS
FKAFDELDRQDLAFNCYQYMFKLAGQIIYKMVLGMSLDHFENINSPPHEIIRLLGEYLILMKKVSLR
PQWYSYLPFGPPKRLAWVKDRIWKLVDQAIEQRPRAPDGDQDLPLHDAALKATCVADYLCRAID
EKGQKLSHEYMLSNCVVLVGAGFITSASLLSWLIYALVKYPGNQERLLQELVDHGATADTKWRYD
TIQSMKFLDKFVKEVQRVHNPSFQTARNAKKDVVLPGGYLLPKGAVIIPTFPSLHMNEAYWDNPN
KFDPDRWDSDAVKQRHKMVYTPFATGPRGCIGFNVALQEAKLTLANLVYRYHWEDASTEPMVY
DPEFLVMRPLNFYARAVQRTTWPQKSHE*
>CYP65AU4|101283|Umbpus1
MTVNDATTIPIITSPQHLLVIGVFAFLSWYLCSAIYTAYFDSLSKYPGPKLWATSRIPSQRSVVSGHHH
HDMLALHKQYGNTVRVGPNELAFFSPEGFKDIYTKRAGHKLPPKDRSHYPLPPNGVDNIVTANDE
AYHARHRRLLGYAFSEKALKEQEPILQGYVDLFVARLRDQAARGPVDIKSFFNFVIFDITGDLMFGE
SFGCLEESKLHPWVELFFSAAKAYSYLIAVSQFPWVKSILEPLIPRKVVQEGLDHFKLTAEKVDKRLA
MKTERPDIISFALRNGMREGHGLLASESEKTMSRAEMHSNAYVMIAAGSEEPATHLSGCMYYLLV
HPQSLNRLTHEIRTTFASETDITMSAVANLPFLAAVIEESFRIYSPFVTSLTRVVPKGGDTIAGEYVPEG

TIVAAHLYASFHSPNNFASPESFLPERWLGTDARFKNDRRDVLHPASLGPRGCIGKALGYAEIRLVL
SKLLWNFDLELCDESRNWVHQEVYIIWDKPALMVRLRDRRVAGKGY*
>CYP6308C1|101476|Umbpus1
MDYSTLPNDPDHPAGSSPWQSSPQPTSRQSFTTPRVQASTSPTPGQSPYNTYDQRRSQDDVSDQDTS
TPRNENYGGAEEYPGYMSAPAENGNSSSYSERLQGPPSTEQGYSGQQYQQQQQQYQRQPQSQQQ
RPVGPHRYHSAGRPAQRQNIPQYKLQAKITGLERTGRKDPILRFDVHTNLPKFRTTQFRDVRRTHSE
FIKLADHLISSNPEALVPAVPPPLTAAGAGTDEDEARVKGSMQRWLNYVCGNDVLMRDDEMVFF
VESDFGYSPVVRMKQPATGVRRRVLKQFAPPPDDTPELHEARPVVKLFYLGTMETGQKVDRVVKA
RRGLGLAESDLGVKLGSMHVQEIHLGLANAYRKLGKTIQTTGDYHAAQGTAEATTLADPLNYHSS
DAFIVKETLTNRHILLRELIQAQQATRSKLSAADRLKASSSVRREKVDEAISALDEARSHELYLSQKT
QRVTSNLLQEKRRWFARTSADLRSSIREYVLREIEAERRTLATLESVRPDIRAIDSSGGLSRLGRESHP
AARRASMAASQGPRGDAWSGVPRRPDGLSRSISGSFVAPVGEGEGGEGEESVGRGRAVSGGTLKSL
KEEDDDDRVDAKNAAISPSSPAVTKQLYGYQAASFLNPALYTHFQLGDYPGLFECRDRALHRRLR
RLVGRTFTTTATTAAERVVAAQIGKFLAWVDEGARAETPRPFDVDFGLCMLNLDIVGALFLGAEFG
ALATRVTPQLLRDLDLYFVEPFVTLKVPFVAPLTSWVLVALWRRFWGSSCRMHEYTLAAFRGYVSR
KGRGVDAAEEGMEQAELLGKLFEGKAAGGAEPLSADAIAAVNGSIIEGGTDTASNVLTFTMWELS
RWAGWQARLRREFEEEGVVFEGGLAAYEAVRGLEVLEAAVREGLRMFPAVVGGLPRVVPEGGRE
VERVWLPGGTIVSMEAYTLHHNPDVFPPPHEFDPGRWLRADTDLANMRESFFAWSSGSRACMVSP
PLLERRVAKHRSRLSMRPLLPRPLPLGPLWLGLSKGPKPNFDMTVERYIGGFEPGDYGRV*
>CYP611B5|101558|Umbpus1
MDGVSQAMLLVALAVGVGTYLMLVRILGAKQDPQEPPLIPATIPYIGHVIGFMRSKSNYYVQLSHQ
SVLPIFTMTMPGTKMYVVTTPELIQAIQKQPKELAFPPIEAKFASKVCGSSAEAHKIMMTNVNGDEG
DWGLSMETYAAMRAALAPGPDLDNMNRVMIQNIAAALDHLTAAGGKHAKIGLAKWLRSSVTA
ATTNSVYGPQNPFKQQAVEDGFWDFENDLVAILIGVLPSITARKGVAGRAKVTQAFERYFRTGGH
KESSILMQNRYETGAKNGLSIEDIARFEVGGAIAILVNTAPAAFWTLLYVYSHPGILEDIRNEVSSIMT
TTVDETGNPIWNLDITNVKTKCPVLTSTFQEVLRHRSMGTSIRQVMQDTLLKDQWLLKKGCMIQM
PSRVIHKDSSIWGSDVDEFNPRRFMKGEVPKTENGKPPNPAAFRAFGGGTTLCPGRHFATTEILAVV
TMFVMRYNMEPTAGAWSLPKTDNTNVAAVVMEPDSDVEVEVSTREGFEGGQWTFSLRNSGMIFA
VVAEDI*
>CYP548AJ4|101628|Umbpus1
MLDLQRLTTTLWRGLLIALSCLALRLLAIWIYRLTFHPLAKYPGPWLAKVSDLYGAYHNARGQLHI
ETEKGHRRWGPLIRQGPDKLVFNSATALHDVYSSNEVQKSYGYTTMIPAPGAYNIFTAVDKGIHKH
KRKVLSQGFSDQCVRAFEPKILDHIDIFLAKLVGSPERGSGDGEWSTPANMTDRCRHLGYDIMGEF
GFGQSFELQLKDDNRFLIEAVTATTNKAGVYVQYPALANLKLEKLLYQRGLGMREKYLQLMSDLV
RSRISKEKDAKNDLLYFLADAKDPETGLGFTEDEIWAESRFLLIAGADTTSTALTTAFFYLSAYPACY
EKLAYEIRNTFSSGSEIRSGPKLAQCQYLRACIDESLRMSPPISGTLWREVCAGGHVIDNEYVPAGYD
VGVNPYAVHHNEDCFPDSFTFKPERWIPSADNSEKDIERARRAFSVYSIGSRACAGRNMAYTELGD
SIARTMWYSDFRRPEGPLGVVGAGAEGSREGRHRVKEFQLMEHLTCSHDGPFLQFCGRKGLTDELF
HDRRAPPASY*
>CYP6456B2|101820|Umbpus1
MDGPFSAITILVLAAIIGLWYILDYLYAPRHSSGEPPVLPSSVPYVGHIIGLLRHGTRYYQTTSAKCKH
PIYTLNMLNGKVYIVTSPDLVSAVNRNSKSLAFNPFIAQLGKRITGHDDATSQIVQHNLNGEQGSG
YVIEVHDGLVAALSPGAHLESMIAPMLQKASTYIDDLDCSNEVDLFAWMRRMVTMCSTTAIYGPD
NPFAKNSDYTDFFWDFDHDLNLLIIDVFPTLTAPKGSRARSILGTEFQQYFENYKPGQTQSSAMIQA
RHAANTKHGVSLWNQGRLEVGTLLGILANTIPSAFYMLVHIYSDPSLLKDIRAELETTAVSTAPPDA
KRTLCILTLRDKCHLLHSTFQEMLRVHAHGAGSRFVREDTILDDQYLLKKGMVIQMPMAVMHSDP
SIWGPDVASFQPRRFLKPNEASKGLKANSPAYRPFGGGASMCPGRHFVALEVLALTACMVLRFDI
MPVVGEWSVPPQRQESLATNVFPPEKDVRVKVTRRAGFEAVVWDFAMK*
>CYP682AV1|102249|Umbpus1
MSLLASFQLYWIVILGAAVLFYLIGRTFYRLHFSPLAKFPGPKLAALTLWYEGYYDIVKRGQYTFEIG
RMHEKYGPIVRISPFELHINDPEYYDELYNGVSKKRDKYPWALKIFGAPDAAIATTRHDHHRLRRG

AMNQFFSKASVRRVEPIIQKNLEKLLIRLRGFQHSCKPIALNTAFTAFTSDVITEYSFGRSYNYLDQED
FNLAFSELMLGVHKMTGILKHVPGIIPLMEALPNRLVTRLNPARNAFLDLQSNIKAQIKDIKDRGSS
DCEKASLSIFEALTQSNLPDGEKTVKRLSAEGQVLVIAGTETTAWTLSVITFYLLSNPPMLRRLRDEL
DQVAAESSEPATCTRLEKLPYLTAIIKEGLRLSYGVSTRLQRVSPDKVMKFFDGKHDWEIPPGTPVG
MTSTLIHQNPTIFPSPHEFCPERWLENPRLDRYLVSFSKGSRQCLGMNLAYTELYLCLACIFSEYGLN
DQKSPGGEMALYETTKEDVEIQFDQFVAIPKADTKGVRVLIT*
>CYP59X3|102303|Umbpus1
MAKLPLDCHPHYLPGQVMRAMPELGPIFYFDAWPMADPMLFVASAAGAYQITQEYSLPKFRVMR
DFIDPLAGEYNLVTMEGDMWKRWRSIFNPGFSSSHLMTLIPDILEDTLTFCGILRQHVQKDDMFSLE
ELTISLTVDVIGRVTLDTRFNQQRSKHDMMEALRSQVRWLPARTILNPLSPVTVLRPLVYWYNTRR
MNDFISRELDTRLATHQNGHVVDKAKRSKSVIDLALQRYMAEKPADKAIKGMDAIFKTVAMCQIK
LFVFAGHDTTASTICWLYHLLSKNPPALSRLRAEHDSIFGTDLAQTASLIASNPHLLNQLPYTLAVIK
ETLRLYPPASSTRNGEPGFSFTDPSGRHFPTHGFLVWSVHQATHREPSQWPQPDTFLPERWLVAPG
DPLYPIKGAWRPFEFGPRNCIGQELALLEVRIVLALTVREFDVRAAYEEWDRLNPGKGGRVNGERA
YQVLMGSAHPQGGFPCRIKMAER*
>CYP5077A4|102583|Umbpus1
MVTFPLFASERSYLPLSLLALSSLLLALYYIRTYAKLRHIPGPFLASLSNLSRRSWVLTGNSHAIHVNL
HRRYGKVVRFPNNVSVSDPSAIPEVYGFGAKFPKSEFYDAIMPYVRGKSIPDVFATRDETVHRTMKR
PIANIYSMSNLMSFEPYVDSTMRYFFERLEELFVDQGKAFDFGHWLHLFAFDVMGELTFSKRLGFLE
NGGDIDGLLANNWNYFRQAAPATQMPWLDFLWKRNPLLPHWTKVNLIVAFGVARIKERQGRSE
KDTADVNNRDFLSRFMEAKSRDAQIPSEALITWTNSNIQAGSDTTAILLSAIFYQLLKHPESMRHLQ
SELDIAAKQGQLSEFVTWKESRNLPYLDACVKEASRLHPPIAFPLERVTPAEGAVICGQHIAGETLIA
MNPWVVNRDKDTFGVDAHSWRPERWLCDEDGKRKMYNNLLTFGAGHRACLGKNISYLEIYKVV
PTMLHNHVLINRKQQGALPIPESPSTPNQVELQEDSSEDGDEDEVEDEDEDEDRQQHDFLNELGFE
DFLGTASNGNSSANVPPTYTPQEIPAVPDEQGAVFNILSLDHQQHHYNLFSIPQDNDFPFSTPSTEFL
WEDRSMITSQGGTGNASDATATPESFNQSQVCLGPEQGHLARGESTSGSTLILEDVQPETVSLILTTL
SNSRAKFRMRLESKEN*
>CYP51199B1|102865|Umbpus1
MERLWDKTSTNPVASAVLLLLALTICVTRILTGLAGKPKYLNGEGLRSVGMLPYWIPFLGHLFSFIA
KFEEFLQQTSYRASHGIFSVNLAGSVHNVIYTPSLIQNIFQQRAPIVNSEAINWYIMVNVFGADRRYK
KDYFLAFEGLIAAVRNNLLREPSLSIAVDATVKAMQEEIPNLVSFCQSPVDQNLWERTSSPITCANG
SEAMGSSVIVEVSLFPLIRNFVGHISMPSLMGEAFMEYYPTVLDDMWALDEGFNYLAIGLPRWFPVR
SVTKAHLARARLNAALAEIHTALDTLAAGGEPGPEWRDLSDVSRVMLERNAAWRSHCIRPNERG
PGDLGILWAMHANANNLVFWILVRMWATPGLVERVREEIEPFVQISQPMQTFGIHEAPRMKIGLA
GLLKECPLLKSCYLETLRLDSRPASVKKIERDFIVRETRQDVRAGAQPESYLLKAGTYINIPHAVHQL
DPRFFEDPTVFKPGRFLVRKGQDGQKQRPRRQQEQQEMTAQVGTLKPWGGGASMCKGRMFAER
EVLAFVAGILVMWDLEPVTPRGWVVPAHRKTTGVAVPSTDVRVRLRRRHRSSMI*
>CYP5282B8|102882|Umbpus1
MDFTSNTTSTNLLSGMDLNSHDFQLAGFIATCISFLAYLSYSPRVDKKSPAFTSDTVLFVGSWRFFTH
KLMFWMNSMAKSKTGNFSFWLGKNHIVGVSGEAARKMYLDNRDLDLIKGITLIGHGPDFIPGRAT
VVHDIWKPTFPNGRSYSQRRLLDLQKSEQLAKRLPRVTTDARAAFEAMAKNPSGVTNPAKACYRI
VVTQASRVVCTDEISDDPKLLESLLGYLPILQSTSSLHLLAFPWMSYVSISYWKRRYGRYGLSSTVTPI
VNKRMKKGSPRVDDALQTLIDNGDSKDYIINFFISMLFIAGANAGVLSGAMLNIVAHHPDWQEKI
YGEIKAAAKAHSTNKDAPLVEQLDSIPLEAWESSFPSIDLCYKEAIRMWVAFPMGRLNDTPNPIPIP
GTDEVIPPGSFACYNTIDVHYNEELYPNPMKWDPERFREGREEFKKQTYGYMGWGQGRHPCVGM
RWAKLQQNIILAYALAMFKWSGCDANGQPNPHFAQPTTALNELAPSLPQGLYCKYVPREKM*
>CYP61A1|102924|Umbpus1
MAAVANGSFRSLAANADYPSAMKVEHAGMLSHVYHGINGWSIALTLFLILVVYDQFKYVWNKGT
IAGPAWKLPFIGPFLESVNPKMEEYKAKWASGELSCVSVFHKFVVIASSRDMARKVFNSPMYVKPC
VVDVAYKLLRPTNWVFLDGRAHVDYRKGLNGLFTRQALETYLPGQEQVYEKYFAKFLDISQKQN
NGKAVPFMPQFRELMCAVSCRTFVGHYMPDEAIKKIADDFYNITAALELVNFPIIIPFTKTWYGKKA

ADMVLDEFAKCAAKSKVRIAAGGHVTCILDGWVSSMYQSELYREKVEKGVAVDGSEKPAMLIRQF
SDFEISQTLFTFLFASQDATSSAATWLFQLMADRPELLDRVREENVAVRNGDRNVRFSMDILEKMTF
TRAVVKETLRYRPPVIMVPYVVKKDFPVTDSYTVPKGSMIVPSVYPALHDPEVYPDPDSFNPDRWIT
GDAEQASKNWLVFGTGPHYCLGQTYAQMNLMAMIGKASMFLDWHHQVTPLSEDIKVFATIFPQ
DDCHLAFSERP*
>CYP527W1|103024|Umbpus1
MAAEMAFPLAGPERASTGLRLSAIVVACAIAALVVSAVISAVRSYRRLQDFKGPTLACFSDLWIFKA
TLLGNLNQQTSAVLKQYGPLARIGPNHLVTEDVDLLRHMSAARSVYTRSDWYDGMKLDPNINNV
ISERNDQRHTALRAKMAAGYSGKENPGLEKSIDDRVQDLVNLIGRKYISTGTTLRTLDFAQVAQYF
TMDVLTDVAFGAPFGYLNRDEDVHDYIKTIRSFMPVLELKTNHPVVNALMGSRLVKALLAPTAHD
RTGMGKVMGVAKQAVQARYGPKKIQRDDMLGSFVRHGLTQQEAESESLLQIMAGADSTATAVRC
IFLHLLTNPRVYSRLLAEILSAHRAGSISSPIRDAEAKQLPYLQACIKEGLRIWPPLTGLMTKRTPPAG
DTFKDVFIPGNTDVSYSAWATHHNTRTFGEDAAVFRPERWLEARGEEAQRMERSVELVFGSGRYG
CLGKNVAFVELNKVFVELIRRFDFSLVNPIAPMKTRCNGIFLQWDMFVRVTELDVYGG*
>CYP530A43|103132|Umbpus1
MPLSLLVVIAGALALAVGTVKLLRVGRRPAGYPPGPPTLPILGNLHLMPSRDPHLQFQKWAQEYGP
VYSLILGTKTLIVLSSDKAVKDLLDKRSDIYSDRPDAYIAQDLIGGGMRVALMKYGPTWRMIRKMV
HNILHVNAAKSYVPYQILENQRMLVNLLDTPDRFFDHIRRYANSLTTQMIYGFRTTDVDDPKLLRIF
EVCDCTPPECGNAWAKMLMNCQQGVEKVSQAASLGTAALLDVYPILRYLPNICVPIRPYAKALHK
SEHDLFVGNWMDAKNAIKNGTAKHSFCVNIIKAQEVSQFSDSLAGYIGGNLLEAGSETTADTLVGF
VQAMLVFPDVQQRAQEEIDRVVGPDRLPTMADEPNLPYIRACVKETLRWMPTVPLGIPHAVIRDD
AYMGYTIPKGAGVALNIWAIHMDPARHPSPRTFSPLRYIHDVQTAAAAALNPDPAARDHFGFGA
GRRICPGMHIAERSLFIGIARMLWAFSMERATDREGRQVVPDIERFTQGLAMMPEPFEARFRVRGK
GREEVVRREWREASGGLDERGQWRVVPKGMALGWGARD*
>CYP544C2|103307|Umbpus1
MLTIVICCIVAVALWFLQGQRSDIRRHGKRLPRPPGTLPFAGNGIWFLQPRHKLLDWFTECQDKVG
FDTFEISVPSLPPGIVINDPQNVEHVLKNNDLFIKGAFFRQRSWDLFGNGIINADGELWRIQRKAGL
RFFSNANLKAFIDEMLPPFLDDTKQALRETAKQGAAVDLQNVFHELTTRLMGKMAYDMDMHGS
LPFSKAFDFASGAIGERFQNPFWKLKELFLGAPLRKAVSEVKSYGDAIVSAAVQKKKHEGNESRITA
FDLLRTNLINSLLDNIEDHQVVADAAMNYLSAGRDTTAQSLSWTLYLLMRHPGAITGIREELQASF
PNADQTLPLSFDTVQPPFLPYTMAVFNESLRLYPPVPVELKECTAPTTFPDGTTLPIGAVVMWAPW
AMNRSKHIWGDDAEGFRPERWFEDGSDPTKPTLKTKTAFEFPVFNGGPRSCLGKKMAELLAVYVI
ASLVWEFDFDEVLDEKMGGCGVGKSRVSQNSLTLPMEGGLPCHVRNT*

>CYP677A7|103344|Umbpus1
MFESIDWVVVRPSLSSVLVVAFIVPFFFIIQKIIYNLYFHPLAAFPGPRVAGATRFWRAYVECVLNRSFV
HVLEELHKTYGAVIRVCPNELHFAQPQAYHDIYNNKNRWDKERRLYQSAGADTSSFGFLTYSDAK
ERQDALRPMFSTKAVKQAQGLIIDKINALSAAFQRLSEAGKPADLFYGYRCLTIDIITYLCFGHSIDAI
EEPEFRAPLVLAMEPLLLEAIRIKHSDTYKAIMTHCPPSLLSKFNPATAGFVDLQQFLQQQIGNLAE
NPGQISHLPHSMTMYHQLMNEEAYKTKTVPSERSLYEEGVNFLFAGADTTGNALMLGSYYLLKSP
KAYHTFKQELQELWPVLEQEPNLQDLENLPYLNAVIKESLRMTTGVVTGLLRIVPSGGAKICETYVP
AGTIVSCSSVFVHYDPNIFSEPNEFRPERWLEQANLDHWLVAFSKGPRMCLGMNLAWAELRLSFG
YIFRKFDMSLDQSSPDELLWREGFVPKFLGNHVKANMTILTLASAIKLDRGSIAKCQPHLRMASAYT
PRPSAATGSIPPTLPPSVETAYRNKCIDLKRRMNEVEESNDAYRLRKVRLMRGIRKMRLERAFLLETL
GKRMRKNGHGLNGTYEDSEGSSEGPPTFSHGAVQPADALGRSEDEIRDVAQRQWREMDEEDKRL
WNGRYEERMRRWQAETEAWRRRGRAAGRADRDAGDDGDVDMGEGEGRGEGGFTAVNG*
>CYP5328G1|103368|Umbpus1
MADPRLWSSLLLLPIAWLVWTGYRLASNYAIARRVGIPVVVVPINPESPVWMLTSDFLGPYIDHVLS
WIPFGSGSFTRYAHRGWDVHDRAKSFLELGDAFILVTTGKNWLYVCNAETFSALLQRRSEFSRPLEI
MAVLDVFGPNLSTACKDWQRHRKATGPPFGEPNMPLVWNECIRQAKEMRDYWKGIPDVRHTVN
DVRRFSLHVLSATGFGKSYPFDRAAEEVPERGSLTYKESLSLILENAILIMLLGPKLLTGSLQHYLPKH

WSLVGHATSTFKSHIAEIIKEEKCLIDEGKANTGNFIGAMVRASEEAAAAAEEDHGKTGGRRSNLG
LTESEIFGNIFVFNFAGHDSIAITLTYVITHLAANPGVQDWIAEEIQHVCRDQEEGATVPYRDAFSLL
KRCTAVVYETLRTSTPFPAIVKTTGSTPRTVKVGGKSILLPAGMNIIFTFASIHSHPRYWGDDADVWR
PARWIESTPLPPNAGSDGTGRSVFDSESLFNPPRGSFLPWAEGDRVCPGKKFAQVELVGAVSALFAG
GWRVEPVREGKGESMEAARGRVKDAIKDSGMVLLVEMLHPERVGLRWVNRGKGI*
>CYP51194A2|103370|Umbpus1
MITAPQDAFAAYKAATVLSFDSFLDEALRAFGVDESSLKLAWHKPSPGDACYRDPNPVNPKQKSFI
HWIRDTYRQALLPGEEMNHMVAHFHSYVESNLQWERVSSFARSPGETKGPIRLSLKDFTTTIMLEAI
TDSLFGDQLVKVEPNIIKYAAEFSDEVWMLVYRLPKYFAGRVLDKRMKIMNALEKYRQIPQEERTA
GGEAWPVEMVLKAQDILGMSSESNNAFVTVIHWAANGNTCMLCFWLLSYLLRDPSLLARIRREIA
PAFQTGTLDLAYLVSSCPLLESIFFETLRVSTGAISSRRVTSPITIGGKRLKPGREVLIKHRALHLNEQV
WGDSAGCFDPERFVKNGALTGHSSYRPFGGGSTYCPGRVIARQEVCIFVALALERMEMVLVPGQA
FPLLDNTRPWIGVTGPMPGMDLYVDVSERRRSVGGGEN*
>CYP682AZ1|103907|Umbpus1
MRVCSKPIVTAALTLLAALGLAAPAISDESPPLFSRQEFCYNAPGTVAGTLLGSRALVAKNPRPYRM
SKEFVNPQLWCAPHCATYIRATYISAIPGQSWGLVQYLMGDAEADAQKQLRALGDVNIRGGLYVY
RAYGLDMFIRDSNNHQLTWGVLREALRALTDYFGEIQRTALPGSLTFIIYDGPIVRVNPFELHVNDP
EFYDQIYTGPSKKTDKWAWSAIAFGTPNGIFGTVPHELHRKRRGVLNNFFSKQAVSRLGPNIQALV
DKLCDRFRKNYETKQPIDLHHIFSALTTDVITEYCFAASDDCLGKEDLDAHMTVVWMKLSEVSFTI
QQFPWLLPIVNALPVWAVRLINPRLYEMVKLDRGWYRQIDNIRVEEKVSDKSTENTIFHKILRSDLD
PQERTTVRLVEEARTLIGAGTITTANALKVASYHLLANPRALQLLTAELKTAIPDPAHPPSLPQLEQL
PYLSAVINEGLRFSSLAHRMQRIAPDRPLLFHEWVIPPGTPLSMTSFLVHHDASVFPSPDAFMPERW
LGRSEKRLDRYLVPFSKGTRACLGMNLALAEIYVALANVFRRFELELWETEWERDVRVTHDFVNPF
PSMESPGVRVLVKGVVG*
>CYP617G18|103931|Umbpus1
MAPWQLSALVSFAASVLLVQLAPQLSISASYVRAFLATFGLQLLIWAVWTVLIYPKLWSPLRHLPHP
TNASFFNGHFKLIASETTGKPQQEWINEIPNDGLIRYTALFNRERLLLTNSRALGEVLVQKNYDFIKP
DQLRQGLGRLLGIGVLLAEGEEHKSQRKNLMPAFAFRHIKDLYPVFWTKSCEMTQAIMSEVSEKSN
AEQKSSSAAVEVAQWASRATLDIIGLAGMGHDFNAIQNPNTELSTTYRKVFQPTREAQILGMISLFV
PMWIVRALPVQRNDDIAAATSTIRRVCRQLIHQKQEKLDKEERVDIDILSVALESGGFSEENLVDQL
MTFLAAGHETTATALTWAIYMMCKHPEIQTRLRAEVREYLPSPEDSSSTVTDKVLDKLPYLHAVCS
EVLRVYSPAPLTLREAAKDTSIIGEFVPKGTRIIIAPAAVNLSRELWGDDASKFDPDRWMGPRRTNN
GGAESAYAFLTFLHGPRSCIGQAFARAEFACLLAATVGRFEMELEDKDFVVEVKGVITARPKDGLR
VVMRPVEGW*
>CYP5104B12|104243|Umbpus1
MIALHQRHGKIVRTGPNEVSVSDLGAIKKIYGAGTRFKKSDWYSVWQGHRKFDLFAERDERIHRTQ
RRLVSHIYSMDSLKDLEKYVDDAVHVFIKKVEGMQGQSMDMGLWVQLFAFDVIGEVTFSKRFGFM
DVGQDDGSFAAIEGALQSAAWIGQVPWLYWAHDFLMPVLGNHLGINARHGSLRKFAAQEVANR
RDRGSDHQDMLDKLLEMQKERPDEMNDMAVLSMATSNIFAGSDTTAISTRSIIYYLLKNPGYKRKL
VEEIDQRKREGKLSTPITLEQTKQMPYLQACMYEALRCHPAVGMSLPRVTPSGGIEIDNRFIPEGTVI
GVNPWVVHRDSQVYGEDVEAFRPDRWLKDDTGDTERFFFAFGSGARMCLGRNLSWMEMSKLIPT
LFMHFELELTQPDALWKETCWWFVKQEGVHVIVRPRSSA*
>CYP5472E1|104470|Umbpus1
MPVLHNLRLPTLIGRLSPSHSILSSLIFTLLGIHLFITWHSARQRLRKHGEKAPAAPSWAPFGIDLLAT
AVRRLISNDFFTWSRELLSVPGRTVSISMIGQDLLMTDAPENIKAVLSTHFSDWAKGETFHTIWEDL
MLDSIFASDGALWHANKDMLRTFISKLRSTDYDVTEKHISQLFQNLEDGESYDFFDQIDRFSLDVVT
DVFFGESAGTLTSEKQPVREAVEDMYVWNTKRVLLGNLGPFLPGSPKASGVMHSYLDRVIDKARA
TNEESDPDEKSRREDSMLGSLLRQGLSRKLIKDAMLAIMLGGKDPSSITLVWVIYELARQPEILVKLR
EEIAHTVGFAPLPTPGQLKQMPLLQNIIKETLRLHPPVGYNIRVAAKDTSLPTGGGLDGTKPVGVLK
GTHIITSNLGVQRLHSSPAPPSLPSFDIFAPSRWQSWTPDLWTYLPFNHGNRVCLGRNFAMMQMEY
FLCRIFQEFESVQLMDEEMDRKCLYKVKGVDGREERGEMKIKMALNTKPAGAVMVKFVKTGKRQ*

>CYP6003F1|104516|Umbpus1
MAAEDSASRKVSQLGISNLLLCHANIIIHDIFRSNSDDKNISDTSSYLDLAPLYGRDRTHQLEVQKLE
KGLLKPDSFAENRLLSQPPGVCIYLVMYNRFHNYVAEQLLLINENGKFSLPPPESDYYRDLSPEDRT
KAEEAAKEKQDEDLFQTARLITCGLFINVSIHDYLKVLMRLHLKDTSWTLDLRIEMPGDDNKEGVQ
RGIGNQVSVEFNVLYRFHSPISKRDAKWTEDFFHENFRHFVNKDNGKGGVFLTQAQFDNLDIPVEL
MRMAIEGHPSRPLSPKEKEEAEKTRHELEMAKPFIPEGLGYRKLDNDGKAIGMFPFARDEATGKFD
DAQLVKEMVAVMEDPICQFGPRNVPKAFRAIGILGILQAWKWELATLNEFREFFGMSRHTRFEDIN
GNVEIQDALRDLYNDPDLVELYPGLFCEGNGDWDAKKETGSGLDPGTSCPNGRGTALWRSVFSD
AVTLVRSDRFYTIMFLWQDWNVASLTAWGMHEVTSDPKMLKGSVFHRLFQRAFPGYFKYNSIYL
WQPLYTPAKNMELAAEQGYLKDLAETPEDLDPAIPNDPDRIRRITAGKRPAKAPAPMEISDYNTIQ
NEIHAKKDNYVNPGVCDTTPFPDGLLKNTPTSKVRSPDNGTDVLQEMATKEKQRLFLDYFVNMSR
EITIRERRKFQGVTYQIDITISYAIPIVTRFIADFLGFGDQIRTQLDQRDRKYCENQIYQHFTNCQDYLIF
NSDETTTWKRRMVYRDSMKFLMGITEQGVRDAQKGLFEFFERSYGTKEDSGHVKELRKFGTMVAR
VLLKEKGKTTEEAAGIMLATALDATHKSILVFTEVIAYLLDHPKYWQAIQDLASQDTPAADEKLMR
WVLEAQRIAVDLWIVRTPTKDDPNTLLRGTEGTGEDKKEVTRTVKKDDILVLKVASKGKGKRKRA
PTAVVSGVMPDWASPGRPGDKLQTTQRVSKNFLKVLVSPEDPDVDIIAVHGLNPTNTEFHAEATW
TVEDKLWLRDFLPPQLPSARVLLFGYNANVAFETSIAGVREQAINLLNRIASKREEAEERPIVFVAHS
LGGIVVKRALVEAKLDDSYKSIREATYGIAFFGTPHQGGNFXXXXXXXXXXXXXXXXXGLQGKGQA
KALPYRGGPSIIAVHGLNPTNSEFHAEATWTVEDKLWLRDFLPPQLPSARVLLFGYNANVAFETSIA
GVREQAINLLNRIASKREEAEERPIVFVAHSLGGIVVKRALVEAKLDDSYKSIREATYGIAFFGTPHQ
GGNFAKLGDIAASIIRGVLRNPSSTFMEALKKDSLFSDTLVGDFRHQLEDYHVLSFFETLPMGRLGLI
VDQKSATLGLAGLRERQIPTDVDHTGVCKFESAEGDDYEQVSFNLVRLVKSAVKAAAERARIASLS
VPSSRPLSEAASVDTVTKSLLSHLPYAVDAPFNTYSRQRDPTCLPDTRVDLLREIYTWADGKDGRFIY
WLNGLAGTGKSTIARTVARKYFENGQLGASFFFSRGGGDVGHASKFFTTVAVQLARKSQSLQRYIS
DAVRKNADIATQSLGDQWRQLVLRPLSKLSGDSYPSSYILVIDALDECDNDDYIRIILKLLAEAQTFQ
TIRLRVLLISRPEIPIRHGFYQIPEAEHQSFVLNHISPSIIDHDITIFLKYNLGLIGQKKPSLSASWPGEET
VKQLVQIASGLFIWAATACRFIRDAERVFLIRKRLAAILQIGSSISSGIPFLQNVLKKKNKSS*
>CYP682B9|105133|Umbpus1
MASIASTAEFSLHHYVILLASLIFAILIGGSVYRLYFHPLASYPGPKLAALTLWYEFYYDCIKGGQYTF
EIGRMHKKYGPIIRISPHELHVNDPSFINELYAGGNKRRDKYYFYARQFGPSKNIFGTVPHDLHRAR
RAALSRFFSKASVAKLEPLIVNTVQKLCAQIETYAGTGKPVELSTAFSCMTTDIVTEYAFARSPHFCD
SPTFEPNFKKAILKGTGLVPFVKQFPWIMPLAKAMPVSVTSHMDPQLGAYVIYLNSIKTQIIHVMEE
QTEKKNQNAAHPTIFHELLQGDLPEEEKELDRLWAEGQLVVGAGTETTAWALSVMMFYLLADPTI
LSRLVEELVTAIPDPSQLPSGAVLTQLPFLITVISEGLRLSYGAATRLQRVSPLTSLHFKSSDGKVESYIP
PGTPVGMTSVHIHQNPDIFPDPLAFIPERWMNDQNRKREDRLDRYLLSFSKGSRQCLGMNLAYTEL
YFGVAAVIRTFGNRLELFETTQEDVEAHRDCFIPRAKVESKGIRLSPAAEYMSQSLGVV*
>CYP534D7|105170|Umbpus1
MLSLIAVALLALLVLYAINIYRSFARNLAAAKASNIPYIIAPIYLFQNAWLVTHRIWMPYLRRLPQR
WTNPWLDLIVPDWTWVHRHAPFRKIGSDTFMTVAPGGIVLWVADASAIHQITTRRNDFPKPIHMY
RSIDLYGKNVVTTEGQIWRQHRKITSPPFTEKNNHLVWAESIHQAQAMLKSWVGDDSDAVKTIRK
VADDTMRLSLHVISRAGFGVRLLWPGVEDGDPPVKGRDAQVPGGVAPSCVAVGRGHTMSYTDAL
SSLLHRLIAVLLLPKFLLKHIPFKVVRNAYESYVEWGKYMNEMYQDKKAEVAAGKEQEGMDLMG
ALVRGAGLNIDTLNRSPSPEKGQKPPKQALTDNEIIGNAFVFILAGHETTANAIHFSLIYLALNLSSQ
RHLQRDLDCIFHGRPTSEWDYERDIPKLFGGMTGAVLNEVLRLVPPVVNIPKSTLKGQPQTIILDGK
KCVVPGGVMVNLISAVVHRNPRYWPTGPPTDPAHPIHPTSNTDNDLEEFRPERWLTESPPDHAKA
ANIVNGHAVDAQAAKPSDTDDLGVNAAPDTASTLYRPPKGAYIPFSEGYRACLGRRFAQVEVLAV
LAVIFAHYSVELAVDEFAHDEEVEGMTAEERRAVWDKAERKSKVLLRDHMRSIITLQLRGCSVPLR
FVKRGKERFDYAGAS*
>CYP5328H1|105800|Umbpus1
MLVTPDRNWLYVAEAEAAYDIFSRGRDFGRPVWMLEMLNVFGSNVSTAEGSDWQRQRKLTATPF
NEQKSSLVWAESLRQACDMVDSWLLLEKEGLKSTSEDTRTLALHVLAYAGFQKSYPFRSIAKDNEA

RQPSTYRDSLSIILKNVLVILVLPPWAFRIPFLPSKWTQIGWAISEFRRYMMGQLADERRLIAEGKPGS
GTLMSNLVRASDTQPDAAAGDAQHRRAHDLKALSVDEILGNVFVFNFAGHDTTAISLAYSILLLV
AHPETQDWISEELNFYLKSDSSEAWRYELVFPKLNRCLAVLLETLRLYNPLPGIPRYTGNQPHTLQV
GHKQIEIPENTLVVPNLMALHTHPRYWGQDSLTWRPSRWIVRSSNPVNADLGTRLSQETLMVPRK
GTFIAWSEGARNCPGKKFAQVEFVATMAALFRNFHAQPVAKVGETSDAARKRVLEVVKDSNVEL
LLQMRDPESVSVSWSRR*
>CYP51F1|105802|Umbpus1
MGFPAMLFGPLSEQLSNQSTGVVVGSALISLCVLAIVLNVLSQLLFKDPKEPPVVFHWVPFVGSTITY
GIDPYKFFFSCREKYGDVFTFILLGKKTTVCLGTKGNEFILNGKLKDVNAEEVYHPLTTPVFGAGVV
YDCPNAKLMEQKKFVKFGLTSEALRSYVPLIVAEVEDFLKRSPLFRGPRGTVNIPSVMAEITIYTASRS
LQGKEVRDKFDSSFADMYHDLDMGFSPINFMLPWAPLPHNRKRDYAQRKMAQTYLEIIKARRESD
EQRNSEDMIWNLMGRVYKDGTPLPDMEIAHMMIALLMAGQHSSSSTSSWIMLRLATRPDIMEQLY
QEQISVLGPDLPPLTYDALQNLPLNAHVVRETLRLHAPIHSIMRKVKSPMPISGTPYIIPTSHVLLSAP
GVTSATPEHFPDPSEWEPHRWHGGAGLTAEEDDEKVDYGYGLVSKGASSPYLPFGAGRHRCIGEQ
FAYVQLGTITATMVRAFKVRNLDGVKGVVGTDYSSLFSRPLAPAVIEWEWRGKA*
>CYP531N1|105844|Umbpus1
MTSSISSALLHVVLGLVFIIFSTSLWSYLRSPTLIAIPGPLVAKFTNLWRLISAYRGLTAVYSRRLHERH
GTAVRLGPNLVSLSDPKLIKTLYSFKGDYKKSAFYNASHVLSPKGHVVETQFSTQNEQYHGQVVRPI
RKYYSMASLIGFEPLIDQTINNLCRRLKQEFVDGPHAGKTCDVKDWLTYFAWDTMSQISFSQTLGFL
DHGSDVHDLIHGSSKFTDYVTVVGQMPRLDRYLNKNPYCRSILPQPSFAILINFSIKAMKQRQSGFD
KERTEIDFLDRFFEIQATNPTEVSDNLIVAWVMNNVRAGSDTIATTISAIIYHVLKEPAVLKKLQQEL
DDAAVKAPVSWKTAQTLPYLGAVIQEGLRLHPAVGLPLERIVPDGGLTLPDGRFIAEKTIVGMNA
WVVHHSQEVFGPDTDSFVPERWLIHDGEEEEAFRQRRAEMMAGMLSFGGGKRVCMGSNLALLE
MHKVLATLFAIFDINFVDPTQEWRLKRTWFVQIEGINVTLKPRGRHADHAELAGLSGDRAGS*
>CYP676E1|105895|Umbpus1
MSEVNPVHCTTRKKKLQAGYAPKSALKRLDCQHASTFVHIFDTSRRAIRSRSVIQASKEKPTEDDML
DRWLRTKQEHPESHYDEMEIETAGTSAIGAGADTTSAALQAFFYHLLRSPEHLDRLRQEIDTAQSQ
GKLSKIVSYEDVQELPFLRACVQETYRMHPSVSFPLPRVVQKGGLTVDGRHFAEGTSLCIHPWVIHR
SEKAFDSDAKKFNPTRWLGPKAKALQRYMVHWGKGYNMCPGQHLADIEITKITATLIRDYDFELV
NPEQE*
>CYP677H1|105927|Umbpus1
MAGDQRWISIATSVLGAWLLTRIVQAVVSVYFGPLSHVPGPKLAAATTWWRAYIEVVKQVSWTDK
LLELHSVYGIQEQLYPLLMVILLVKSMKLMWNLRAGDALHFSRPSTYHEIYNNTLRWDKDEILYHS
FGEDHSSFGVLTYNESKKCKDRLTPLFSRRAVVNMQGLIQDKVNQFCEILKKNDADGKSSDLFLGF
RCLTNDVIMCFCFARSVDALNAPGFKAPIIEAMDASGSSFPVFRNFALIRRFLFALPPWLSIRLSPATA
GIFEVVQMLRAQVTEVVANPKLLQNAPHPIIYHELLKPREDAAEVPSFVSLCEEAQTLVFAGSDTVG
NTLMLGTFHVLENTPILWRVKEEVLQAWPVLSECPKFEQLEKLPFLTAVIKESLRISPDVSSPLTRVVP
SNGAKISGTAIPGGTLVGMSSIFVHNNESIYKDAFTFNPERWLGPDSKGLDTWLVPFSKGPRSCMGS
TLAWCELYIAFAAMFRRFDMKLDDTSRAIQSLSSLKDYKKEALSSTLRRIESLQPLLDQRVQDADEG
TPTTLPKGPLPVPSLCTLHNGGREFDNVQDHISNLEARNLPALQPGSASRAQPGLLDSAATFPAVPL
GSAGDDEITHHPVHRAAARRAEYGQDGRRSPPRFAAGRRWRCAPSNPLLAGIDVARAGLWHRG
HDGHGAGAAGPAPRRAGAQVEAGKTPSKEAAKTPGVDGSAVEGDPLPGFQVFALNWETTSVSSL
APTLGESASGLNLVLTCGCNYSPYLFEPFVRACAENTARPPVACLIAQQLRPEPVFEAWLASSMREF
AVRKVPDELLDEDLQSSKVFVLHSGILKTAAEGWPLCR*
>CYP6001C39|106146|Umbpus1
MLRRFSTVFKKGKKDESKGDGFNGVNGVNGSSSSAKPDPQRRRSNLTPQKKEKEPEDHSAGRAEV
ASTFEQYAQLIHASRRPLPTQSGDGAYLDHDIPSGLMDDLKHLGFKDVGTLMGVMKTKASGELAD
DKTYLMERVIQLVSALPQSSKNRVDLTNSFLGELWDSLEHPPLSYLGDKYIYRQADGSFNNVMFPRL
GAANTPYARSVHPNTIQPGALPDPALIFDSVFARKQFTPHPNKVSSVFFYWASLIIHDLFQTDHRDF
NNSQTSSYLDLSPLYGDVQEDQDNIRTFKDGKLKPDCFSEQRLLGFPPGCGVLLIMLNRFHNFVVE
QLAVINEGGRFTKPNPGLPPEIAEMSWAKYDNDLFQTGRLITCGLYINITLLDYLRTIVNLNRSNTT

WTLVCRASPNSTSSCSLRQDPRVEIGKVFGQDGAPRGTGNQVSAEFNLAYRWHSCISEKDDRWTQ
DMYKELFGKDAHEVSLPELLKGLGQWEHNLDKDPQKRPFAHLKRQSDGTYNDDDLVKIMTEAIE
DTAGSFGANNVPTSLKAVEMLGMQQARKWGLGSLNEFRKFFGLKPHATFEDINPDPNVADQLRH
LYEHPDYVEMYPGIVAESAKVPMVPGVGIAPTFTISRAILSDAVALVRGDRFYTVDYHAKNLTNWG
YNEVQYDLNVEQGCVLYKLFLRAFPRHFQANSVYAHYPMTVPSENKIILRNLGREDHYTWDRPAA
IPPRVNLTSYVGAKYILERPEQFKVTWGAATGYCMGKGGFDFMLSGDSPFHTKQRELMSKALYRD
QWHQEVKDFYEHVTLKLLREKSCKIAGINQVDITRDVGNLAHIHFAAQMFSLPLKTAENPHLIYSE
QEMYMVLAVIFVCIFFDLDPAKSFPLRLAARAVTQQLGKLIEANVKTVNMTGWVANLVDGRREN
HSPLKDYGVHMIRKLLESGLGVNEVTWSQILPTATAMVANQAQVFTQLLDYYLGDEGQVHLPDIN
RLAKLDTPEADDCLLHYCMEGIRLNGTFGSYREATAAMTIDDGGRPVSVKPGDKVFCSFVGAARD
PKIFPSPDSVRIDRPLESYIHYGDGPHACLGRDASRVALTAMLKVVGRLDNLRRAPGPSGQLKKIPR
EGGFYIYMREDHGSYFPFPTTMKVHWDGDLPPLK*
>CYP51235A1|106159|Umbpus1
MAATYNSIALWVLVPVLAVYFHYLRXLRVKRDCREPPVIRPTIPLIGHIIGLLRYGTDYYAIINTKQQL
PVYTLEIFNTKTYVVNSLDMVAAAQRNTKTLSFNPFLLFSSKRMSQYDKAGMAVVNNNIEGAHGK
WGYIPEMLDGVHHTLAPGPALDKMNHTVLDKLCPAINGLRDGETFGLQAWIRGLFTVCSLESIYG
PDWTSSPELTQAFWDFERDITTLCADVFPTILAPKAYKGRQRLFDAFTEYLRKGGQERGSDLIKVRY
ATAVRNGLTLYHVARGETALLLGILPNVTASTFWILLYIFSRPTLLQELRTEIAHAISTSPSTPTDPTTH
RIDITKLRAQCPLFVSVYQEVMRLKSTNASVRKVLADTLVADRYLFAKDAIVQMPSASIHNNAAFF
GPDPKAFDPRRFIKPDHATAKGAKRPPGTFRVFGGGATLCPGRHFASTEILGSVAVMAMGFEISPAE
GGKWVWPRTMDSRLMTAILGPVGDVKIRVERRKGWEGVRWGFATSLVATEVEMDTL*
>CYP6267A2|106265|Umbpus1
MSLLAIVAILLALWAIGTVVYRLFFHPLASFPGPKLAIATYCYEWYYDLIRGGQYTFKLKELHERYGK
STQPKVLHFNDPDYYDEIFNITNGKTEKPYKAANTFGPYPAAIGTVDHDLHRVRRGALNPFFSKRS
VVEILPFVQNIIEKLCHRFDAASRTGEPTNLKYSYAALTLDIMNEYCFSTDPQNIMKSDYGRKSLDD
VDSFLAISLVNTHIPWLMRVMYSLPDWFSRIINPAMVDILELREGLAKQVEDIRHGNDLSYEQSGHR
TIFHSLLESKLPFPEMKRDRLRDEAFSLITAGSITTAHVLRAVSYHIAADPAVRQKLFQELRTVMPKR
ADRPKLQDLERLPYLTAVIREGLRINNPVTHRQCRAFPDKALRYNGLTVPPGTLIHVTALLIHENERI
FPEPRAFKPERWLGPIEQQPQRYLVPFVRGTRACLGINLAWAELYLILATVFRRFDFDISSVIRERDID
VAADMIIGVPASDSQGIIVKVLATQD*
>CYP504A47|106500|Umbpus1
MAHQIVGIALIAVFFFVIRYLNRTDVPKIKNLPEVPGVPLFGNLLQLGDEHAKKAAVWAKKLGPVF
QVRLGNRRIVFANTFESVKHLWITNQSALISRPTLHTFTVVSSSQGFTIGTSPWDESCKRRRKAAATA
LNRPAVQSYMPIIDLESNASIKELLADSDKGQKDIDPNPYFQRFALNTSLTLNYGIRISGSIDSALLKEI
VHVERVVSNLRSTSNNWQDYIPLMRIWPGRDKEAREYRERRDKYMTLLLDMLKDKIAKGEDKPCI
TGNVLKDPEAKLNEAEIKSMCLTMVSAGLDTVPGNLIMGIAYLSSPHGQEIQKRAYEDIMRVYPDG
NAWEHCLTEEKVPYITALVKEVLRFWTETPPDAMPHGRNTNADNAALAKYHKITKPVPPAIQPAT
ETPREPKSLNTGIPKGGMQAGENGAGTRR*
>CYP532A32|106652|Umbpus1
MLEPIVLLARQHWVTAIAVALVAWLVKNRFNHDLQRYPGPFLASLTDWWRFWDVLGRRPDITHV
KLHREHGDVVRLGPNVLSFANPKALKTIYGLNKGFSKSEFYPVQMALSKGSRLPSLFSTTDDDYHA
ALRRCVNSAFSMTALVEYEPFVDDTTEKFLDQTQSLFVSKNAVCNFAEWLQYYAFDVIGAITYSKR
HGFVDRAEDVDGMVKKLGRLFSYVAPVGQIPILDLFFYKNPILRLLDRCGILSFTFPVVTFAKARMSE
RLSEVHQAKVDGMDPEKTVLVRRGDLLSMFLKAKEDRPDFFHDGRVLTMAVSMAFAGSETIAISL
AAVFYYLLKNPNCYQKLMHELDTAISDGTVEDRLNNLVTWAESQQLPYLGACIKEAFRLHPAAGL
PLERVVPAQGIDICGEHIAGRTIVGCSAWVIHRRPEVFGDDVDTYRPERWIEAGKEKRKEMEATMF
QFGMGARTCIGKNISLMEIYKLVPSFLRRFEVALANPDQEWKLHNAWFVKQLNFDTTFRPRRRDV
AAAA*
>CYP65FF1|106952|Umbpus1
MAGTESIITPEGVLMLGSLLLGVGLTYLFARAVYNVYFHPLSSFPGPRLAAASELTKSYYGARGRMIP
WVQALHQQYGEVVRIGPNELSFITDSAWKDIYMHRPQFPKHFNFTNPNETDALISADDASHSRQR

RLLAHAFSDKAIREQEALLHVYVDLLVSKLQEESHARDGVVDLVDWYRYTTFDVIADLCFGESFHG
LENKVEHAWVSSIYSSMKATHFLDIAAREQSFKYPQKKVTARLNRETDRPDFMSYILRYNDEKGVT
RDEIDSNFETFTIAGSDTTATLMMGYTYHLLQNPKVLERLNAGVRTIFDSAQDIKLAALGKMPYLIA
VLEESLRIHPPVPGGFGRRVLPEGATISGRWVPGGTAVNIPQKAANVSHHNFAEANFFIPERWLEV
HDPIFDNDRKGVVQPFSAGPRNCLGKNLAIAEMRIILAKMIWHFDMELMNVDKDWTDQRLFVLP
ESKPLLVKLKPRVL*
>CYP539A52|107164|Umbpus1
MIDELLKSLSFGRVFSAAIVIYSIVFYSRRLRVDREIRALGGHAPKVRTYLPLGIDFVYRGITATVKHR
NLELWEWLFKSGNANCPYTVEVNVASERLLFTADVENIKAILTTQFADYGKGEPFHKDWRDFLGD
GIFTTDGAEWHKNRQLIRPQFIKDRVSDLETFEVHVQKLIGLMGGRGEEVDVSELFFRYTLDAITDFL
LGRTINSLANPQVKFAEAFAEVQRVQNIISRAGPFNWLVPRRSFFAGIKILDSFVTPYIDDALRLSPSD
LEAKTKSSTGYTFLHALASFTRDRTTIRDQIVSVLLAGRDTTAAMLSWLFYELSAHPAIVRTLRAEIL
ARLGPRTPPTYDDLKNMRYLQHVLSETLRLYPGVPFNVRLALHDTTLPHGGGPDGLQPIGIRKNTP
VGYSTLLMQRRRDLYPEPSAAFPDVAVFAPERWEHWTPKSWTYIPFNGGPRICIGQQFALTEMGYT
VVRILQRFERVERYWKDGEQVLKAEIVLQPGAGVRVGFWEAKGEA*
>CYP584AS1|101694|Umbpus1
MTISPENIKALLSTQFADFRLSHRLPFFAPLITGGIFVSDGPAWQHSRALIRPAFTRDQVADLPRFDR
HVQRLVSQIPVDGTTTVDLMPLFFRLTLDYVTEFLLGESMESLVSAPGSEQQRFGAAFDYVQRQLAG
WNTSGWGAWVWPNRRCEEGVRVVHGVVDGYVERTRTKARAARGRAAGREKDAETEVESGGGA
KQQEKDGEEESGRYIFLDELALSTDDPVEMRNEVLNIIFAGRDTTASILTSTFHALARRPDVWARLR
AETAPLEGQPPDYKTLRNLKYLRYVMNELIRLYPPVPANHRQAVGDTTLPHGGGPDGTSPIFVAKG
QVVGYSPWSMHWLYATFQPDGEALRPERWADGEGEGEKGI*

>CYP51191A1|102665|Umbpus1
MDRRAQAPSCGLHATSQGGERPIRRLSQHPYLFGLDTLYQDFIDLHCHNSTRRALSRHNAYGQTYI
FTVFGHSTIHTIDPANILAVTTNDFADYEKGDWAQTIAKYMGKGVLVNDGKEWNASRTLLKPLFR
RNRAADVRMFERHVDCLIEYLKRREGQFIDCRKTAQMVVLDITTEMLTGQSAVSFKRSGSEASMED
DGQSMRGPALLDLIDELEPYGNMGIELGLFALPVFALRYRKIMSLIKGIQRFFETAISDINADMGRRH
RDEQNCERNNIIEEMLAQGLAPAQVQGELQNIFFAAFDTTTALLTNVFDCATRQPDLIKRLQVECT
AVVGSRLVDEADITCMPLLRATIFETLRLHSPITYHTRKARTDTRLPRGGGTDGHSPVFLPRGTSITW
STYALNRQAWAYGDDWAAFKPDRWLRDDGKTVASTETFMPFGSGPRNCLGQQFAMLQVTYIAA
RLLTAFEHFELKEWAVPFQEAAAVTHYNGQGTWIKFR*
>CYP584AS2|104412|Umbpus1
MAYLTTLALFALAVLTIYGAYTKTRRYLTIRAFKTHHKCQPPRPLPGSGLIGYRVIKELWTSHRAAN
SLEKHTAHHRSLGDTWELSFFHVDLIMTISPENIKALLSTQFADFGLSHRLPVFAPLLTGGIFVSDGP
AWQHSRALIRPAFTRDRVADLPRLDRHVQRLISQIPVDGTTTVDLMPLFFRLTLDYVTEFLLGESME
SLVSGPDSEQQRFEAAFDYVQRQLGGWNTSGWGSWVWPNRRCEEGVRVVHRVVDGYVEKARAK
ARAARGREAGKEKDAETEAASGGDAKQGKKGAEEKSGRYIFLDELALSTDDRVEMRNEILNIIIAG
RDTTASMLTSTFHALARRPDIWARLRAEIAPLEGQPPDYETLRNLKYLRYVMNEMIRLYPPVPANSR
QAVRDTTLPHGGGLDGTFPIFVAKGQVVGYSPWSMHRLHATFQPDGEALRPERWADGEGEGEKG
IRAGWEFLAFNGGYCLYGIMATEKLRELEKLEAKPTVGLGFLPSTPSLSTATAAVLARDQACTLSGD
SDYMQRAHLCPRNELIWFRKNSMRRYNDRCELSGDNLMDDSANALAMRPDIHQAFDACKFVIVP
KNQRWVVHFLGKTNNLGSSFHNMPVSVLATAKPEFILARLAWAVFPLVKSFMELGPSRLVRVRVE
AEDGAQEVQRTITPVEFSKLESKRSRSRSHSPKKRKASLPVIPEDTSSAKSRQLDDSSLTDTPSLYYDD
EDGQETQHESLGEHEDDNSEEGRIANLRTHELRKRQPIVILA*
>CYP52AG3|105887|Umbpus1
MPEDPQSPFHAPIITPRSGASHTQLTVLALVLAYCFYRYVQKQMQYRSETAFGDAHGCLPMKAKIP
FKWPLSLDLVKQTWDANTDQRLLAFYQQFFDNLGPNLEQKLLGGIGYVTIDPENIETLLSTKFQDF
GLGSRRPAFMPFLGEGILTQDGSAAERSRDLLRRQFVRAQYQNLEGFSEHVDNLLACMSSSSSGAV
DLQPLFFRFTLDTTTAFMFGQSIGSLKSDVLESFGSSFNEASLISSMRVRLSDFYWAYTPSRYSRACAVI
KRYTDDYVNLALSEVKGHGADATSNRYVFIKELYEELHDPALVRDQLVNVLMAGRDSTACLMSW

AFHLLLRHPQVLERVRREITSVVGDEQNLTRAHVRKLPYLKCVLDETLRLFPSAPINIRTSLRTTSFPR
GGGPDGSSPVLIRKGMGIGYSIYHMHRRRDLYGEDAAIFRPERWEGSKLAHIGWGYLPFNGGPRTC
LGKDFAAMEATYGIVRILQTFPNMRLPPGLQLEEVGMEKQALTLTVSIANGCKVQLY*
>CYP52P13|105889|Umbpus1
MVWDFRVLSSQIAPRWVLLLVLGFVALGLLHIHRWLDRVQREKRLEATLISDTRCQDPPRLRYRWP
LALDLLIEAFKADQAGSILQFFLSVVDRTGHTFEQVLLGARGVDTNDPKNIESVLSTQFENFGLGAR
SANFRPLLGHGIFTQDGEPWRTSRELLRPQFMQTRSKSFTDIQEQIEKFLINVKASSAGVVDLQPLFF
RLTLDTTMAVLFGKTLDSMKTKVSGDEAFFARAFDQAQHELARRGRLGDLYWLLDGFTFRRSCRI
VHEFVDKIVVDALNETRPGMASEEPTGRYVFLTALISKTRDQRVLRDQLINVLLAGRDTTACLLSWT
FTLLARHPEIQQRLRNECEDLPSFREGGLPTPAEIKGMKFLGHVLQEVLRLFPSVPVNSRSALKTTTL
PTGGGPDGLSPVLVRKGEAVGYCVYAMHRRKDIYGDDAESFRPSRWDPDNKEGPDLKGVGWGYL
PFNGGPRVCPGQDFALLEASYTIIRLLQSFRHIEKAEPSTAKERQTLTLVVASATGCKVILTPALLEGG
TLREAEARF*
>CYP6544B1|101378|Umbpus1
MLSLTKSLDAVHEKCMSQGETTLSIIQLTKATLLDAGTAALYGDQLISISPDFITHFSAFDTNSWMLL
YHYPAPLAAPCNIPKRKVLSAFTAFFSLPHSSRSEVAQFLQTIEAEQRKISLSNADIASSAFIFYWAINS
SPWKLAFWLLCYILYDPQLHANILAETKPAIRPDAPGLVDVQHLVTHCPHLEATFNETLRLVAAST
SIRNVIAPTSIGSKILLPGSRLLMPYRPMHFSSKIFGPHTDQFNPSRFVRNPGLSKHPGFRPWGGGSTF
CPGRFLARREICAFVVGLLHRFDVEVVEGETFPRRDEVTPNLRIMEPMKGDDLKVRFRSRHFFSHQE
QIPSSVVAVRFPAEGAIMSSEPEHVQARPKVNLNDASGAEKREVNDTATAILKKKKKPNSLIVTDA
VNDDNSIIALSNNTMETLQLFRGDTVLVKGKKRKDTVLIVLADDELDDGSARMNRVVRHNLRVK
HGDIATVHPCPDIKYAKRIAVLPIADTVEGLTGSLFDVFLAPYFREAYRPVRQGDLFTVRGGMRQVE
FKVVEVDPPEYGIVAQDTVIHCEGEPIQREDEEGNLNEVGYDDIGGCRKQMAQIRELVELPLRHPQ
LFKSIGIKPPRGILMFGPPGTGKTLMARAVANETGAFFFLINGPEIMSKMAGESESNLRKAFEEAEKN
SPAIIFIDEIDSIAPKRDKTNGEVERRVVSQLLTLMDGMKARSNVVVMAATNRPNSIDPALRRFGRF
DREVDIGIPDPTGRLEILQIHTKNMKLSEDVDLESIAAETHGYVGSDVASLCSEAAMQQIREKMDLI
DLDEDTIDAEVLDSLGVTMENFRFALGVSNPSALREVAVVEVPNVRWDDIGGLDDVKRELIESVQY
PVDHPEKFLKFGLSPSRGVLFYGPPGTGKTLLAKAVANECAANFISIKGPELLSMWFGESESNIRDIF
DKARAAAPCVVFLDELDSIAKSRGGSQGDAGGASDRVVNQLLTEMDGMTSKKNVFVIGATNRPE
QLDNALCRPGRLDTLVYVPLPDEAGRASILTAQLRKTPVADDVDIKYIASRTHGFSGADLGFVTQR
AVKLAIKQSIAIDIERSKEREAAGEDDKMDEDGLEDDPVPELTKAHFEEAMQAARRSVSDVEVRRY
EAFAQKNEWNR*

P450-fragments
>CYP505A-fragment1|118844|Acastr1
MGTPIPTPPGLPLLGNINDVDPENSIASLMHLAELYGIVTTLGYNTDACADRNSPTGPIYRVVLGGQ
ERVIISSHELLDQICDERRFSKAVSGPLGQIRNGVHDGLFTAYPEEHNWGVAHRVLMPAFGPLMIRS
MFDGKLTPASARLHR*
>CYP630-fragment2|119576|Acastr1
MLYSKRSHALLQIHQTHPIVQIGPNSLSYGDVKTIKDIYAHNTKCLEDTFYTLLSGTHNRLADVVDK
PEHARKRMVWSSAYALKNLEGWEHKVADKTERLINGFDARCTARYRKDIRARKLNS*
>CYP59U-fragment2|119928|Acastr1
MLDRLPRNAHYQYALGDVAREHFSKEGVYYFDLWPVSDLFLTVVSPNIANQIHANPEISMGRPSLL
PRFFKPICGGPSMFDLREKDWKPWRAIFSRGFSADHVLSLVPGMVDETAVYCQTLRSLAVEKTMFY
PDLTTLRFTVDVIGKTILCVVSVISSLSQN*
>CYP682-fragment1|120003|Acastr1
MELPKAFNHIRLSPSDIPLFAICVLLVYTVYGAIYRLFLSPLAKVPGPRLAALTDWYEYYYDAVKKGR
YTWKIAELHEKYGPIVRINPSAVHIHDPNFIDEVYPGGGKKVDKPSRYASMFGIKVATFATDLHEKH
RVRRTLINMFFSKRSIAKLEPVIQSVVDKFCNRLEGFRKSGEPLNLRNAYMAMGTDVIHQYCFAAP
NYYLDEPDFQPSLYQALIEASQMAASKAISLKKDWTIREAQIQELMDAGEEARNKTEHATIFHVLL

DEKVPPQERTLFRLGEEAISLIGAGGITTQHTLQTMTYHILANPPVLDRLTAELKQAMPDPNIPAPL
QQLEXXXXXXXXXXXXXXXXXXXXXXXXXVFV*
>CYP527G2-fragment1|122578|Acastr1
MAILKLTELDTTILLKYSATLVVSWYIISSITTWYKLRHIPGPFLGKFYHLWSIYHQLAGDVGPVHLEL
SKYNSPLVRTGPHYLVTTDPNVWRHVNGARSIYQRDAWWSAGRIDYQRPSLADTLDAASHDKMK
AKLAGGYSGRDVDLERIIDGQIAKFADVMRRKHVDRNKVDFADRSRFFTLDVITRLAYGKEFGWV
EADEDLYGYGAEISKFAALGALISDVTWLHPFVRWSFINSRFKPKPTARHGVGKVVGLGREEIQSRF
AEDKKDDRDMIGSFMRHGLTVTEIEDEALIQIFASSDTTAIVIRSVMLHLIATPRVW*

>CYP5328-fragment1|126188|Acastr1
MTNSILLLLLLLPLTFVIHNLYCLLNYRIARKIGIPVIVLPASPDHPKHFELGDAVVFVTPAHNWVYV
CNAQALKGIFQRRNGFPRPPEMFLVARRAVEAGPQVQLQGLARFDTGKLVVDSCPWSEVAHKVA
PTQDGCSLRGNLAGKISSALCSEPRREWPIQQQLLAPKAQGSEVSLSPKYTAISSFIISRDTIQLPLHSS
YALYLLAAHPEIQVLISEEINAAIPDDHSSTWKYNEVYPKLNRCLAVLLETPRLWDPPIGIAKSTGAN
PQVLPLDDCEILIPANTRVIPNNNAIHSHPRYRWC*
>CYP5328-fragment2|126855|Acastr1
MPSLLAVQTHPEYWQDPLLWQPSRWISVSSLLAVQTHPEYWQDPLLWQPSRWISVSPVPRSSKPEL
DLTSRLRQEVVFTPAQSTFFPWSDGPQNYPGAEFAQVELVAVLACLFQDHRVGIIPTPNESFNDARK
CVLDVTEDCDLELLLRMRNADKVRPAWRRV*
>CYP5334-fragment1|127116|Acastr1
MFAFGFGPRKCLGQYLAEHSIKTLIIHLIDQYQLGLRHGQNKEADYKVEEGNWIPLADVEIDLSRLR
*
>CYP548W-fragment1|118920|Acastr1
MTAVSFGADYRTMEEPQFRYVVEAIEKSNVRLGVLMQASELTFGGLDRKLFSNAAKAGARFVKFL
RKLLQKRLQQEPADTVKDIFSFLQQLEEVRTTFSSADEIAFGAKLNSCVFLRACLDEALRITPPGGGP
LWRVVELGGTRIDGEYVPAGCEVGSGIYAMHRSPRNWPNPGNYIPERWLENKEKDKSENQENKN
ARQPYFPFNIGPRSCVGKPLALAQVMLTFAHILWEFDLRSANAGEPRLETDDTEPPEYMLQDHVTG
QKEGPILHFRPRF*
>CYP684-fragment1|119584|Acastr1
MLEKGEEDRYTQDLKKNAAYSNIRTELSTLCKMLSVLPLPIMRELQETPLRLLGHARQSLQRYKNLL
ATNPESVTGKMLFSKLFRAKDDGGLTDADMEVEAAAYIIAGSDTTAITLTYLIWVVAQRPDIQEKL
GVELAVLSDDFAYDDLRHIPYLDHLIDEALRLYGVATGGLPRIVPPGGHTLAGHFVPGGLTVSTQA
YTLHRDPSIFDEPEAFRPSRWEKATPAMKEAFLPFGAGTRICIGIHLARMELRIAVATFFRSFPRTMV
ADAEGMSEKDMEMENCFLISPKGRRCLISSR*
>CYP59U-fragment1|119927|Acastr1
MDKYIGDELDKRYDEYKADPEGQQTKAVIDLVLQAYLPQDGKTRPERLDPEFRAFAISQIRLFVFVG
HDSTSSTICYILHLLATNTDALAQIRAEHDQVFGTDLSAVPSTMKEQPHLTNTLPYTTAVIKEALRLF
APAGCNREGKPTATLVDDQGNHCPTEDAVVFTIHTELHRAPTYWIRPDEFLPERWLVESAPSIRNC
TAHPPTGFDPTSSSRSDGWSSLNTSFTR*
>CYP682-fragment2|120004|Acastr1
MERSYPDRAFKYGDYVLPPNTSVSMTTMLIHDDPSIFPEPRKFMPERWLDSEQDQDCHPNPSNNYD
GGEKPLQQEQSQQQRLAKYLLPFGRGSRACVGVNLAYAELHLALANIFRKFELELYDVVPERDVD
VLHDFFNPSPALGSVGIRAKVVGRVA*
>CYP676-fragment2|120182|Acastr1
MEDSTLQGVQSTVDAKDASRRQRNIGNAYTLSSLLKSEAQIDDVLRKMRECFDKVADTGKAVNLG
EWWAWAAFDVLGELTFSHRFGFLEAGQDIDGAIASTELMMIYGVTMGYALKLNTLLVDNPIIRKIN
RWFNITDKMHLAKITMSALAKQEENPEASHNMVSQWISNLAKNRDTMSPDEIKAAAIGNVGGGS
ETVSATLQSAIYHLLRTPHALHLALAEIDAAQLSPIPLHTEVAKLPYLQACIKEGLRMHPAGPWNLP
RVVPPTGLTLGGEYFLAGTILSVNPWVIHHHPSLFKRPESFEPERWLDEQQRRVEEPFWIPFGAGYN
RCPGRQLALMEVPKMAALMLRDFEFERVQGGRDWVWRNNFTAVPG
>CYP6798-fragment1|120348|Acastr1

MTNNEFNSQNEDMPLLDSFIKEMARLNPPTINGTHIPAGNWACVPHQAMMQDLANYSEPTIFNG
FRFVNGKDGSAKSDRGSLIPVGSSHTGHLSNKCGQYLLLPLGLTVQRRPSTPMWKPLGQDTEAVKT
ICPARFYVSMVVKMILVQFLTCYEFKLADENVPPAFAWGIQLHAAPTEAALGRRARPTTIKKVSPTC
NTARGSTVEIAVLVAHRRKWRKLRQDIDATSLHEEEAYEMPSPIPNESPVCVLPPALEKRSTRAVKA
SSLARELVDHVSRTGGVEVLKELGETLFRGVEVLKELGETLFRLRAHSGRSQVTCKS*
>CYP65FT-fragment1|120400|Acastr1
MWKGRMPHVARSLHDQYGDTVRISPTGLSFSSSQAWKDIYGIRPGKPQLPKDPAFYFPQKEGPSIFIS
GDADHSRMRRLLSYAFSERAMREQAALVSEYHDLLIQKLHEEIQGPAGGKVDVVKWYNCCTFDII
GDLTFGEPFGSLEKGGYHFWIANIFKALKMTQELHPTSSEARAKHARFSAESAEKRLALNTDRKDF
MSYILQYNDEKGMSVSEIKQNSKILVLAGSETTATLLSGATYFLCTNQDALRKVTAEVRRAFSNSSEII
LTSAAHLPYLQAVIDESLRMYPPVPSTLPRRTLPGGDVVNGQFIPSQVT*
>CYP5148B-fragment1|121990|Acastr1
MELYTRYISDHVVAKTFATTFGFIASAALCVLFLPPTLSSKKRTDESKIPPGPRGWPVIGSFNSLTHYP
ELVLDKWAKAFGNIYSVWLGNQLFVIVSEASIAKDLMVTNGNVFSSRKEMFLKSQTIFAGRGITATP
YNNRWQAPKYIKRN*
>CYP527G2-fragment2|122577|Acastr1
MPQIAVVVCTGMRTTNYGRLRVYTILTFPPPFPDSRLKTDIRNAVSSGKVSIPITLAQAQSLPYLQAVI
WEGFRMRPAVTYGHYKVVPPGGDTIAGIWVPGGTAIGHNHFGMMRNERIFGDDVDVFRPERYLD
KPNGDEMQRTVELVFGTGRWMCVGKQVALMELNKVFWETKVWELGSRLMLDEQLLRQFDFQLA
HPDKAGWKETSSVTFTHRDMWIKITETEK*
>CYP653-fragment1|122722|Acastr1
MFPGFLHPLVSKVLPSVWRLKKQLKAVKEELLVSIINERRTAQRSSVLDYEKPDDFLQWMMDLAEN
QTDAEAGNLAHRLLGILSMAVVHTSAMAVVHVLFDLITMPEYLEPLRTEIQHLLPQGWDKATQSE
LLGMSRLDSFLRESQRFNPPGHLSFNRIVKEPMTLSDGLKLPEGTHICMPSGPISMDNTVVPNADTF
DGFRWFRENKPTAAFANTSPTNLHFGIGRYACPGRFFAVYMIKAILSRLFLDYDFRFHEGQRGRPK
NILIGDKVMPDIYTELLIKRRTV*
>CYP59V-fragment2|122889|Acastr1
MVWTLIQAIHRDPRYWPQPDTFLPERWLTSKDDPLHPVKGVWRAFEFGPRNCIGQELAMLEMKL
ALTMTVRKFDFSARFEEWEQINRRKGPKTINGESAYQTLDGTNRPRSGFPCRVTMRGS*
>CYP5104B-fragment1|124265|Acastr1
MHRTMIQLHKKHGKLIRTGPNELSVADLTAIKRIYGPGTKFKKSDWYSVWQGHRKFDLFAERNEGI
HGSQRRLVSRIYSMDSLKDLEIYVDDAVSHFMTKMRELQGQSVNMGLWVQLFAFDVIGEVTFSKRF
GFMDAGVDDGSFRQIEGALRSAAWIGQVPWLYWMNDFLSPVIGNHLGINARHGSLRTFAASEITK
RRDRGSDHQDILDKLLQVQKEKPEEMNDMAVLSMTTSNIFAGSDTTAISIRSIIYYLLKNPEYKRKLV
QEIDMRKSQGKLSTPVTLEESKHMPYLQACMYEALRCHPAVGMSLPRVTPSGGIEIDGCFIPEGVRI
DFYSVLRLADPQPTLDNGWSQSLGCPSQQGGLWR*
>CYP5105A-fragment1|124382|Acastr1
MAGTESTAKSIAIVHFHLLANPPLMAKLRAELRTVPENASWPELEQLQYLSAVIAEGNRLSFGVTA
RVCRIAPDEALQYKQHTIPPGTPVSMTTLCVHTDETIFPDPWTFNPDRWLGPLGAERRKYQMAFNK
GGRNCVGINLAHAEMFLVVAAVVRYDMELFETDISDVEFQHDYHVSFPKLDSKGVRAVVRGKAA
LV*
>CYP676A-fragment1|124880|Acastr1
MFFLTDLRSCVAFAAGTFVRITYNEVSVSDPEAVDKLLLTRLRKGDFYQAFALPDHNYGNQMSELD
PKEHVRKNKNIASGYALSNMMKAEPYVDTIIELFERRLDQMSQMGEKVELDRWFNFCAFDVVGEI
TFSKRFGFVDQGRDIGGAIANSRKLTLYISLIGHAYWLHGILLANPIIGWLNLQPSNHVFETCLAAVE
ARKKNDKVRKDMMELWLEQRRTHPDRMQEKEILAAAVSNIGAGADTISTTLQSVFYYLLRNPKHL
NRLREELDDAQSHGNLSPIVAHMEAQKLPFLQACIKEAYRFHSAVSFGYPRVAGKEGITIAGRTFPE
GSLLSVNPWVIHRNTDIFGEDAESFNPERWLDQERLRMMDKHLIHWGAGYNQCPGRNLAHLEISK
ITATLLRDYDIEQVDPKQEWTYESWFVALPSNWPCYVKRRNVAKVE*
>CYP50105-fragment1|127121|Acastr1

MEACTRYATTAFISGVIWNFLPLGPFRRWVYWIGSLKHRMDLHRATSYLLPIVEERMAKESTSDKKP
VDAIQWMIDMPPTSLSELDARRHSYRILHLTFAGTGTSISLTQHLIWQILLFPEYLAPMREEIKGALA
EYGGWISDKALSHMPLLDSFIRETLRMHPPGVCKSDSPVLTITSTEAKSLVGGARTVMQKPYTFHDG
LSLAPGARIAFAVLSTNLDPDNYDDSQKFKGFRFAGPEVSSESQARVSAATIDEKFLS*
>CYP613-fragment1|119548|Acastr1
MELVNLRDPIPEQVEKQNFFNLQVRGQYAQYRCLSKPEHVQIYYRDSNRHTKAINNNSGWLFGEV
LGVCVGLVSHAQWTRLRRHVEKPFSHPSAASYTGAIIRQSREFIDDLPLKAPNFQQNHTINPADDL
KLFPFFVVANIFFGNLSPEQKKSLSELAPLREELFKEVIKGGINRAALSRYLPTRGNRLLSAFQERWG
NFTQNAYDEAKRKGADVPIVSLWEASLEGFITKRECLQTLDESLYANLDVTTSATSWNQILLAQNP
TAQDELRREVLRFRNDSSDDIYQQYLNRGDTFLAACILEASRLRPILPFSNPESSTENREIGGYVIPRN
KNKTDVIVDSYAINIDNPFWVNGSQYNPFSAMANTSDGANSADSVAQLVHLLESLNGKVEEVLQQ
LESPKFTTLLDEKLHNPDHLPDRQLEVMSSQVVDSMDKLQLRLVPSVMLLADGFFGYLFSKALWTV
VDAKVPDVLDEKGPLSAAEVGLLVGIQPKRLSQLLDTLINNGIFLYDSNTNRYSSNRTSRLLCRDHW
TQWHLWADLYPNDFFDISRSMPQAVKIGEARNAYQIEHDTKKNMFVHLSEQGKIEKFHNTLGAG
AVAQAQGFSIDYPWDEIGSEHFIDIGGGSGAFLASILREHPKMTGSLLDLQHVVDAITPEFRQPTGKF
FDVGAQVRNLLVGDFLKHVPPASVYTMKWCLHDWSDVDVVNILKNVRRSIVPSAVARFVILESVK
QQGRSGRLPRYGDLIMMITVNGEERSRSDWERLAQSSGWRVEQIVYLRRAWPCAIDLRPTWS*
>CYP630-fragment1|119577|Acastr1
MNEWLAPRSQLPRMSPCYGSAVRVGLGLRVVSPAVEDLQPHVKAVSEDVEAQPRLERHCPSPRHA
TPERYQAGEKLDDFFSALMEDKEGNPNNLEWGEIVAEIAIMMNAGSVRHLPYLCACLDEAMRLLP
PSTFGLPRRTPPEGTTIFDTFIPGNTSVSISAYVAHRDPTVFPDPETFRPARWLKEGAKELQPYFITFSA
RARGCIGRNISYLEQTVLLASVLHRCELRLPYDGWEHERWQAFNLSPREMPVKVYNRDFAKVLHIT
QHVNEAKRAVEGKSTFWLIKQLYVVPSNLRALIAACGLMAISQPGGFNSLMYYSSNLFGIVGFSNP
VTVGTLIAGTIFIFTWGFLMLVDRIGRRRILLCTMWAMALFLALAAICFHWIPVHQDLIFVNAKVSW
PADVVLVCMAFYVAFYSSGMGNTAWLSSEFFPMETRALGTMMLTCTCWGSNVIVASTFVPQMEN
TTPSGAFGSKPPSASLAGLSFISATPKLRA*
>CYP51188A-fragment1|120821|Acastr1
MLTSHYLPHWTDRFVVPGILALGGAMKKLSSYLTLEVEQRLRDMQEGRKDIPRDGITWVIEASTTE
DQRAVRRIVQQVISVFFASAHQLPVLLMFSIYRLCEHPEYTKFLLNEIEAMLKLPTTDHYKHLPLMES
FLREAARHDPLDSLSVQRKVLKDFKFSNGSYVPAGNVICVPQQAVMRDPKYYDRPDEFLPFRFVRE
HGDGQDDGPVQKFTDLKPHFYLWGAAAKPCPGRWYASAVMQQFFAHLLTKYNFKLADPNASLT
FTYTTIVFPRPRLEILLQER*
>CYP5148B-fragment2|121991|Acastr1
MVPVNPQLIPTTMQRRGKKLHQDLVKTYGGLVNGIKQRLDGGAAVEDCLAKSMLHDAGEEGLD
DLDMSILASAFMVGGVETTAAIMQWFSAIIPAYPDIQRKAQEELDRVVGRSRLPGIEDEKNLPYCHA
IIKEVRIPITTLSVTLVERCRNPFWLGTPHFTTEDSIYNGHFIPKDTVVVLNTWTMHHNSERYPDPKA
FNPDRYINDPLTSADSANLANPNDRDHWMFGAGRRICPGMLVAEREIWLTIARMLWAFDMTEVP
GERIDLNEYDGLSGRSPMPFRINIKPRDAEVEKVLKRESGRWAVCA*

>CYP5082-fragment1|127117|Acastr1
MSCGGNMDGLDAGEALNSLSWQNSAGVMLSEMMEMAGYHLQSQRSHLDFFARHVAPALGSHP
EIDRKPPPWRSFMTDDGSPIELSWSWSVQEPAPIVRYSIEPIGDRAGLCPDYFNTHTSNELVHIIQRSY
QGVDLTGYAHFFKELVVCGEGTFVPKITKEDGSNSQIFLAFDLLDEKIMLKVYFLPALRARETGQCK
LSMVEKAISTLSPHGQSLSGAFSLVCEYIRSLKVGNRPEIEIIAVDCVNPTLSRVKVYLRSRETSFASVV
SMMTLGGRLKELSKEGFATLEELWRLVLSLDPTISTSEPLHLNNRRTAGILYYFELQPSRSYPKPKVYI
PVKHYGKSDLKVANGLSSYLKEKGKRLNGMDYRDALQRLCKHRPLDQGSGLHTYVACAIEDISLA
VTAYINPEIYHRPRPAGPQTGHQHIAQGTSTSRNVAHESVTVLKLKAKEFQHGFKIDAGLQARTKS
ANGGWYFHQLLGECMGLVNGTRWRNLKSEFDLSFSHRAVLQRSVDIEGEACRFLRKLEIHHGQEP
FTLNASNAFMTFPFFCTSHLLYGPMTESERDRLWSIGQTRLALMRHVIKGGIMRYKACKWLTSTAV
TELSLFQKEWAVFNRDMYRSRMELDPPPPIVSLKRSETEGKLSKVESLGKILQTLDEMLFANLDVTT

HVLTWLITLLADKESTQQKLRDEIRNVQGRPDVYCNRKDTFLHFCLMEAIRLRPPAGIGFLLTQSDG
ER*
>CYP65-fragment2|1800|Clagr3
MPQGHRSAARTGWFERDPNLHPTLPGRAHSLIHVLRCAHLSAEKALREQESLIQSHIDLLIARLHTE
INSPNNGVVDMVQWLTFITFDIISDLAFGKSFNCVKEGPHPWVELILDFMRKVAYLAALGYLPTPIF
KMIISTLQYFMLEEIQKGNEYVDRSVAIRLEQGTSRKDFVSPILELVSHGRNSSKQSG*
>CYP51164A-fragment1|3613|Clagr3
MSLRDALHDPTIYSSPLSFDPSRWLTTDPAEKKLMDMAFLPFNKGPRIKPMPHSYPVPTYPLHSDGS
PSAIPFNNLGSRRGSEIQLTLDGSLAFANIYHVLSALFRRFDLELVDTIRERDVDMARDKFISKPVKES
KGVCVRVVGEAEI*
>CYP5069-fragment1|6212|Clagr3
MPTQAMRTKTITPSERVENGLFLAVANLIWGYDFRLPLGKDGKESSMDLSDEAFLDGAIRIPKPYEV
RIIRRNPERLALVKSTWKRAKEEGYVMRGLTVTESEGIAVNEKLKLDGTKDRTLNAFQEDVRLPTG
VLYCSYQVPT*
>CYP682AZ-fragment1|10834|Clagr3
MCSVVSLLLYTAYGVIYRLYFSPIAQFPGPKLAALTFWYEFYFEVFWSIHLISPTGPIVHINPYKLHVT
DPTFANMLYPTSAKNVERWSWSAGMFGSTEMTFGTVGHMLHRRRRGAFSHFFSKASVRRLEPLIQ
TLVDKLCEKLSQYTDTREPVNMVHAYSALTQDVITEYCFSECRNVLEMENFLPWYYDLVQKPCELC
QMFVPSSIAITRSS*
>CYP548-fragment1|1786|Clagr3
MRRIRLLCCLTCYLPDYSAPSREVPRPTSRSSDGAISTVAVNCRGPTPDVLETKDDIWLQGECSEIELV
PRVSSSEGRMERLDMHRQSSPCSRTLAHAFSDTALRDIEHYVTSNVQIFCDQLFSKDAKHQTEWGH
ALNMSSMSNYLNFDILAELCFGKGSFRMLTESNHRHVVDLMLHSAWRGMICGTLPVVHDLRLDK
LFFPTLARDRQRYKNFSKSLAVERTKQGEDDDRKDFFHYFLNARDPETGLGFSTPELWAESNLSIIA
GNIPTGTTVGTGFYSLHQNPQYFHGPFSYKPERWIVGSSAEVTAESAELAQCGFFPFSFGPRGCIGRN
LAYMELRSVIARTVWLYEMGLAPGSMVGNVEAGAEDRRRQEFRLLDRWQAEKDGPMMQFKWRV
RQKHRMMFGGKQVKM*
>CYP65-fragment4|1799|Clagr3
GSATTNTLLSGALYHLLTHDSILQSLLAELHSAFPSRTGVSMAAVGSLPYLNAVLEESLRVYPPSAH
GHQRIVPHGGATICGKVVPEGTSVGVATLAASLSERNWTEPLAFKPERWLGEAWAGDDYKSFQPF
LVGPRNCLGKNLAYAEMRLILARLLLEFDFELCEESREWYKDQKFHFLWAMPPLKLRLRERAF*
>CYP5570-fragment1|4546|Clagr3
MGDITFSRNPNVLRHDENRHVVEELPKGVVGIHLTEQTGHMPWLLKCGLDRLLFNDFIRGITPLKF
LSESFAEWRVTQGDSLPGRDLFAALLDAKVSQTGRGLDLKDLISEAALFIVEGSDTTITANTTTISYLL
HYPNTLERLQRELRTKFKDAEDIRMGTDLASCTYLPACIDESMRLANPIGSLLPRETLPGGLKIDGE
WFPPGIDVGVPHYTLHHNEEFFPDPFEFRPERWLPMDKRSQTRAESFTTSTTAKEATLDMDLEVAQ
SAFMPFSIGRTSCIGKYLAYQEISLIIARTVWQYDMRIQPGSTVGEGHSGLEWGRKRKNEFQLQDRF
GNPKIGPLFCHAWKPWHLWRYEAHLCVMGLSTD*
>CYP5406A-fragment1|7175|Clagr3
MGWGVTRKKVFNILLWITSQTLENKVSRDEKPVTQGHITQWTAEMTRLRDAAAVAKITLGILFASA
FQVPMLAQFCIYNFCKHPEFYERLRAEAVECKDTSFGSLNQETPYLDSFMKETARLSPGPILSAPRTV
MVPYTSPDCYHIPTGNWLAIPQLSLMRDESIWSHAFAFDGFRFVNEKDGTSKSRLTHPSYEFPFWGS
IRHACPARFYVSVVMKMILSHLLIDYEIKLADPTARPFLTLSKMRLPSLFMTILLRRRGVTGDDKSDQ
AATEKAD*

>CYP6789A-fragment1|8063|Clagr3
MPYFMACVHETLRLSPPISIALPRHPPTGGIYVNGEWISEKAKLGANPYVIHRSTKIFGSDADTFRPE
RWLGDHEQVRLMHKFSLAFGYGSRKCLGKNIALFESQKFCAQLPRDFDTRSCSPERPFEAQNWGV
NVYSGQYLKLTTRTKRSDGTKSQ*
>CYP5042-fragment1|8898|Clagr3

MYLALSTISAGNEDAMYLALSTISAGSDSTRIPMNALVAAALCHLDAMRKAREEADAVCGNSAGR
LPGTGDMHKMPFTCALLKEVLRWRPPIPLVPPHQLTQDLEFEGYYFPAGTEFVINSFPVAHDIDAPE
AFYPERWMGDNEASIIQGLWVCGGGRICVGYKLAQTQLFVAYARLLYCFDYAAAGEFNSMRIGYT
SLEEPISSEGHGP*
>CYP65-fragment5|10067|Clagr3
MEITRPDFMSFIMRHQDEKGMSIEKIQRTLNSLMVAESETSATTLSSTTALLIKHPEKMKKLMNEGR
GAFKRDSETTFEAVYNCPYLIAVLKESLLLYPLVPTGFPLVDPEGGNVISGYWVSGKEHPRSSRQAM
QVITHDTMFRSGPQIVLAVTLSFQILSLRSAGLATHDMPMTTSLCSTHDHIGRGIVLPAFETRLFMAK
VNWNFDVEVHMDAASNWFDQNGFTL*
>CYP682AZ-fragment2|10833|Clagr3
MHDDPSIFPEPRMFDPRRWIGPERDIRQKYLFNFGRGARQCVGMNLAEAEMHMALAAVFRKLGR
QMELYDTERERDVDVRHDFFVTNPNLDSRGVVTSAGDERFARSLMRMEFERARHKPTTEPNAARD
EL*
>CYP52-fragment1|10873|Clagr3
MLSWQVGIQLHVFFRRPLPINMRQACKDTVLLVGSGPDRKAPILVRKGVAVSYCVYAMQRRRDLY
GEDVNEFRPDRWDANEASERDLRNKNLGYLPFNGGPRSALDEFAILEASYTIVRPLQPYQTISVGEE
ETEDEQTHPRHTITLVVASAKGYNVRLGV*
>CYP-fragment1|11111|Clagr3
MSQSACIKEAIRMTSPVSSRLPFTLPTPLVYTFSAVKDDMGRSQTYEIPPETNMGMSPRLMLYNAEV
YPDPECFGHSRWLNYENISPSKAQGDGRMLKPARQTDLKAMEHSFKAFGGGPRRCIGMNLAYASL
YYILAAVVRRFDLEITDAVRARDVDMNRDNFSSVIARGREAVRVRVVGDEAENVQ*
>CYP5095-fragment2|5456|Clagr3
MTSMFTSTPPAPFLTSHKGKIVRIKPRDVHIDDPEDFETIYTPSAPYSKLEFFENRFNMPQATFATADF
HLHKLLPDSHAYKPERWLDNPKAPDGVKSLSRYMVAFGRGSRMCLGMPMAYAGVCITLATLMRR
FKFSLFETGSENVEFWQDFVMPQPKLGSLGVRVLIN*
>CYP5168-fragment2|298|Dibbae1
MDTLEQDKLMLNGDDVTMIIAGGILNAHRSSKRTSGLDQAESTDNFYELKKLPRLNGIVNETLRLH
PAVPTGGLRDTPNKALKFLVYLHQEISRYVLQSTP*
>CYP5095-fragment1|1169|Dibbae1
MDKLCEPLEKEYAGTGKILTINDMWGYWTPDIIVGYCFERFYEFIWQPNFRAFFTTAMIDLLETVHF
VTQFPSIIHMNRMPASWIKFLQSGMASVIQFNKQMEDQIAEILRCPNAGEEMVSQDTVFSAILQSDL
PPEEVSLVLLQHEAISVVGAGIETTLRALSVAGFH*
>CYP631C-fragment1|6185|Dibbae1
MHVFRKAPAYIHHLRFPLSRRTGKYLSSVTVLGHYLPAGTVVGGSPYVANRHKPTFGQDAEFWRFE
RWLEKDAAHKKELE*
>CYP5129-fragment1|6190|Dibbae1
MANPTRFTRVIPDSSRGLHIGPIHVPPGTPIGCAAYTLHHGASIFADPSAFRLERWLEDDDRLDMEK
SMMPFKG*
>CYP5369-fragment1|6630|Dibbae1
MIKEGSTLPKKLPPKDGPVQELPKFTKLKFSMDGLAGGWIPYGGGPFMCPGRHFAKQESIGSFAVF
WANYEAELQTPSGFCPEPGMKFFVLGALPPKDRTPFRVTRPSAWKA*
>CYP682-fragment3|7518|Dibbae1
MFTLINIVQSYCLALAVIALSYATGAVVYRLYKSITVSSNRALQGDCKDAPELARDMAETRPVVEMI
YDELYSGPGTRRDKSVFMFRALGSTRSMHLTAKHDVHRIRRAGSNSYFSKRLINHLEPVFQLLASKL
QSRVQEFRHSKQPPILNHAFAAPSTDGIITLYSFGKSYNELEKPEFADGDVQDYSRFWGDDS*
>CYP65-fragment9|9026|Dibbae1
MEEVLRTFGAGSSHISKAVATLGLLLLTSTRLAFVRSLRKGYLAHDIQKVHKRYGHIVRIAPDDISFA
RADAWNGIHAHRPGHSPFPKNPVWFAPTPEQPSSLIIANITDHARKRRILKHSVTEKALRAQTPIIN
SYVDLLIIKLTEKIAASDAKKSQHCAGHSAMVQLYDI*
>CYP65-fragment6|10330| Dibbae1

MPGKDIYVQKPGQATFPKYPSRYGKWMWIKGALDIFSAENEADHAQLRRLLIPAFSDIAIGELEAL
VQKNVDILVRKRVTDSAVKGRVDMSA*
>CYP542-fragment1|10990|Dibbae1
MKSQVQEIHENKTSIKQQLDTRSTIFEELVFSKELPEREKSIDRLFQEGQTVVAAGTETTAWASSVIIF
YLLTNRDKLLKLYAELFQLMPSPEKILPPTSVEHLPYLIRLPLRKDRF*
>CYP50334-fragment1|11141|Dibbae1
MITAEALRLYPLVRRIEREVGEDGLGEPIIATLNPYSEPSWVWGDEALHLRPSRWINLSREALQHYM
AFGGKPYTCPAIDNFGPKMIGLLVAALWKSLKEEWNPNLQRTTRLILDWQVESFWDDFRDEAQLQ
REGMLIIP*
>CYP6716-fragment1|12561|Dibbae1
MVLFLKAYLATLLGVLSHLGFFLHGEHHLYAPRIFRLYASLPIIAVLLQIGAGTSIRDASVKSLLLVLS
YAVGLFGSILLYRIMFHPLRNFPGPFMAKTTKLWNTVHTFNSKNYLLMDGLYRQYGDFIGPNEITLF
RAEAVRALDGLGSKCSKAPWYDIMKPRVSVATTRDKTVHSKRRKIWQKAFSTQGEWS*
>CYP573-fragment1|1691|Dibbae1
MKRSPKKSRTDILHFLETRTWEEDLLHLTFVKVEAVLIVIAGADTTATSKWKTEIDDVTAAGNLSAI
TQYDGVLEHCSYYVPRIRKTRRLYPSVPNTFPRLVSKGGITLGGKLVRAGMEVTIHAWVVHRDAAL
YDPDANAFEPERWLNSDVSTVKQFNKYDITFEYGNRARLGEGYGVYGVV*
>CYP540-fragment1|3289|Dibbae1
MFSQSFIGAGFTTTSSLMSWVIYCLATHEGMQDRLLQELIDQRHQWRHRLHTRIITLFLSLISSRKKS
GAYIIPHSSLPALQKPTASFPIPAGSVLTAALHHIHNNPALWDNPTQFNPDRWDTEKVKNRHKHS
HIPFAAGPRSCIAFNFALQEVKILIAELAYRYQFEKTGEELVDLMRPMNLYVWAKNDQTGLMEPKV
QKEFGFALL*
>CYP5472C-fragment1|3550|Dibbae1
MSDLHKLKYIKMVIKETLRDHHPCKESMVTDLPEAKYLTVGFNARVPIKDITLPRGGGPDGKSPIAV
LKGTQIIYGLLSLQHRSDLGVEDVNVWRPERWETWKPNSQWEYVPFNHGPRICIGQQFANFQMEY
FLARLCQEFESVTLLPESPSQEGLVKLELNTKMAHPVYAKCVRRARV*
>CYP5042B-fragment3|5583|Dibbae1
MVSPSLSIFQLDSTSNSIGISSIVDFFPQLASIPILFQLWRRHWEETGQFHYDAFQTRWKPVKRAIAHG
IAPPSFLRDVLLHKDTKYSGDDEEAMYMATSIIAAGGDNTRMAMNTFVMAALCYPDVFRRAKDEI
DKVCSPIGKAKRLLRLVDMAKLPYICAMVKETLRWRPSVPLIPERQLTQDLEFERYWFPKGTNFVIN
CVAVSRECEEPDIFKPERWLDGTESSIIHGLWQFGGGRAYLAWL*
>CYP5091-fragment1|5965|Dibbae1
MPIVEKRLTSRHRDTYNSPTDKSMDCMQWLIETSPLSNPWTAERMISEILAIWFSSVHQLAMSTVFA
IEDLCAFPEYSDTLRREAAMQSVTPDGLPLLDSFLKESARFTSVDAVTCRRKALTSFKFSDGLSIAQG
DWVCIPQQAMMHDPLHFRDPLKFDGFRFVQERGGEDIVAHSSKASLFTDTSLDWLVWGLGKTVW
YDH*
>CYP5947-fragment1|6921|Dibbae1
MTFCKAAIDPTIFPEPYAFRPQRWIDGSQKQFDYERYVLPFGRGSRICIGQHCVLSLSYAQLYILLAGI
FRRVDMELFETIRERDIEIRGGGPLGEPSRKTKGVRVRVIGIVP*
>CYP65-fragment10|7702|Dibbae1
MTYLLLTQPSAYQKVENEVRNAFQMIEEINGTTLLELPYLQACINESFRMHSPAMIGSARRISQSGGI
AGHWVPAGTGVQMNVYACGRSSLHWRDPDSFLSERWLGDEKYKDDRRDALQPFSYGGLGTALE
DSKSFIHNDVSALGVPEFSPTELVFPLLALLWPRFA*
>CYP6389-fragment1|7805|Dibbae1
MTSLPIREKYYSSVKVDCLQWIIDSSPRKRTWTTEKIIQEFLALWFGSVHQMAMSFVYALYDLCAHP
EYIEPLRYEVEKQVQNQWVDSLENMPVLDSFLRESSRMNPSDAISVRRKVMKTFAFSDGSQIPIGNW
ACVPQKVMMRDPQIYVEPDTFNGFRFISTDNDNNEGKLERSSSTLFTKADAEFPFWGLGSHTCPGR
FYASAAIKLLMAHILLTYDIKFANEQQKRTFSWRTAIVPVSTVAILFKKR*
>CYP5127A-fragment1|8623|Dibbae1
MHSALDYIEDTLKTAEVVRLLPKFITPFVGEALAARLSSHAAVYNGLLPIAEQRLQEKTLEKLGQKV
EKHHDCIQWIMETSPKQNPWSAQRIVHELMAIWFGSVHALSTTISFAIHDLCLHPEYVEPIRKELESP

QYTAFEQTAQGLPLLDSFIKESARLTPVESMSTRRHTLQPFSLSDGTKVDVGDWLCTPVRAMMQNS
EHYPQPLEFNGFRFANPALLDNATSPAFKFPQLDSPSKLTDVSESWHVWGTGRMAW*
>CYP65-fragment1|9025|Dibbae1
MKVALEHFQLAIDRVNQRVNSESRREDFMTNVLKFNESEGKGINDNGGDTRNEIRSFAKEEELTLD
SIQNLTYFNAVLEQGLRLCPPVAAGIPRVVPPGGDTVCGKWLPENQFQSTNGLYPLLLDGSLAQRN
LKPGRWLRDPPPEFASDQREASQPFGVGPRSYIGKRLACAEMRLVFARLLWNFDISIPLEEQSVLSW
ASQKTCVLVEKQPFEVRLKNIRERSWEP*
>CYP5042B-fragment1|9198|Dibbae1
MFVVGASLILFLLCGFVVIRERLKPVDRKWRLRGRRWKLPHGPSGVPILGNMLQFFEARDTGGLTL
YLQSLRSYGEMTTLHMGSHTWVLLNTGYVASEIIAKRAKVTNERPYMPIAGGLVSNNKRAVIRQA
AEWAEGRRVIHHLLSGSALKLYGEWQELESVQLLLSYLNEPENWYAHHFRYSTAIFYRIVMGERLF
KSQEQLDNYQKVTMEFLFSLFRSPIDFFPTMDDLPILLQFWR*
>CYP5042B-fragment2|9199|Dibbae1
MGRFHRRIFQEWWTPIRAAVVDGTAGASFVRDVLLHPDVRFVGDDEEAMYLATSVMAAGGDNT
PCAIRQSTTRARAEIESVVEVGDTTRLPNMEHMASMPYLCAVIKEILRWRPTVPLIPPHQLTEELEYG
DYIFPPGTDFLINTVAICSECEAADEFKPERWLDGNEGNIIHNFWGFGGGRRIYVGHKVVQQALFM
AIGRLIYCYDITPNHFPSKSAYGARDMRAQYLQRLQNVD*
>CYP5571B1-fragment1|9922|Dibbae1
MAITKPASKFIKDATYYVGIGFPEATFGMIDPVQHRIRKQVLTPAFSAAHVQEIAPGIQARVESMCK
AFDKHAVTSEPLNIAAALKAFALDVISEEVAGQDYGALQYPGFRHPKLGVMRECIEGAWICRAFPT
ASRISLALPTAVTSPFFKIPMVEVIKTARSGLDQYLQDRDEGLLEGMNISNAKVTNTQKTMVSSRRK
VSIPIERLLDPTSAKDHKVPDSHSLNLEILMLLGGGNDSTSNTMIFGVYYICKYTEVQKRLEEELRAA
YPIPGQTISYTTVKHLPYLVKGPNFYRREYIY*
>CYP5042B-fragment4|10072|Dibbae1
MGVDHYKVFRAWWDPVMQDMAGGVAPPSFTRDVILQSNGKYATNSDEAMYLATSIVAAGSDNV
RMAHNVFMMAAVCHPEVMAKARTEIDSVCGPDANRLPNLADMESLPYISAILKECLRWRPVVPLI
PQHHSTKEIHFDGFVFPAGTDFVINSLAVCGEVDDPADFKPERWGNREMNITDGL*
>CYP676-fragment1|10380|Dibbae1
MPDSSWANQMSELSAQKFSVTQKKIAGGYALSNVIKAEPELDQAIQCFQYQIDRLIGAGRFINLDV
WFTYFAFDVVGQITFSQEFGFLQQGKDVGKCIATSHILVPYLSIMAHFYQYHDLIMSNTIMAWLDL
QPMKHVMETTVRAIQERERNGNSRIDMIEHWKSHSEPLTDKEILSTASANVAAGADTVASELQAF
VYLLLRHPDCLRCLRAELDAAALNHRISVPAQYNAVQDLPYFQACVSYSVERYMKRVSDIASFQLK
ETYRFHPVSATSYPRVTPKEGIEIAGRYFPSGVRKSTSFIGLAQALLDCY*
>CYP6544-fragment1|10725|Dibbae1
MNAQHVLINAGTTAFFGETFLKSDPNLLRNFIHFDDNNWMVWYKWLDATLMRTPKAKVLKTLE
RLTSLWNEVLRLTNSSAAVRTILQPTCVGGKILKAGHKVMSPFRQLHFNEEVFGKNINDCDPDRFF
RNRSLAKDASFKPFGGGSTFCPGRFIARQEVFVFIALLVHRFDVEMAPVGDVEKGRQAFPKLESKKP
TTRIMSPQTGEDLILKVRKASVI*
>CYP51200A-fragment1|12560|Dibbae1
MLNILALSEYEGRLLYFADQLQQWVLRSNGATVNVSSLFYLYSFDVMSDLAFGEPLKMLECSDFHY
AVYLLHSGMAFLGPFSSVPWLARIGFSIPGVAKDFKKLLGWSAKKLEYRLKIMSWLIEASRENGTM
QEDQHWLRGDCFAIVVAGRSSDTVASTLTSIFYYIAASSEHAYKLRAEIDSLTSLTDIHALEQMHHL
NGMINEALRLHPALPSRGLRVTPSDGLTIGGRYIPGGVTVVAPRYSIGRLESSYEQGDKFIPERWYSK
PEMVKDDSGFSPFSMGIEFSIYLENAVNKTKDATIA*
>CYP52-fragment2|12644|Dibbae1
MGDLYWLFDNKEFRENCKETHRFVDQFVDVALKRDKSTQKNQKFLHSLLEQTSDRVEIRSELINLL
LAGRDTTAGFLGWLFYILVRHQDIQSKLRSIILQDFGPYSHPKEITLASLRECKYLQYCLHETLRLYPP
VPVNFRQAVRDTTLPRGGGPDGTGKVFVPEGTQVNYTVYVLHRRKDIWGEDADEFRPEGWAERK
FGWEYLPFNGGPRVCLGHPMLVTNDGVEQFAMTEVAYVAVRLLQRFDRMENRGDSGVVVKHNL
SLNSSPFPGVNVILHKAVHN*

>CYP52-fragment3|12645|Dibbae1
MDSKLIIIGVALTTFFFYLFIHKLRKSQRLAAAAERLGCQPLRILHYRLLGAVDLIYELFNADRQKVLP
QYLKERFARADATTFGHIFLDSKDIFTIDPRNLQAMLSTQFQDFCLGPTRRNSFVPFLGHGIFTEDGD
AWERSRAMLRPHFSRSQISDLTLEETHVRNLTRALSVDDSGWTAPLDLQIFFFRFTLDLLLSSYLANL
STLRVRV*
>CYP6259-fragment2|12690|Dibbae1
MDLYSLQRDPKIWGTDAEEFRPARWSDGRPLWEAKWQYELFFGGIRMCPAQTQLLTQVAYLLVRF
AQEFQSVEDWEEVFGYLERIAMMVESRNGIKIAVVPVEKK*
>CYP5472C-fragment2|3549|Dibbae1
MLEEYNHTMELRMLGTRLIMTDDPENIKAIQDTQFWEVAKSEEQHQIFKHILGDAIFAMNGEEWK
QEVALYRAHMSRVRDSDFAVTERHLLNAFDLLAKEGNDGFDIIDRLQLDIVTEVFCGESTDSLTSDQ
QPFRNAMETLQKIASFRQLLGQVGVYLDDKWIAPQAVKFIDDYQDAFAGKAFARTDANPVSGSIC
LIDDLIQKGKTREDIKNAVTSTLLAGKDPSTTTMAFGFYEIARHPDVYAKMKAEVEEQ*
>CYP52P-fragment1 |4302|Dibbae1
MAILFGRPLERIQAKRGADEKQFAKDFDFAQHMLALRGRLGDLYWLLGGAEFKYSCKTVHDFVD
KIVLDALEEKKSTPEASNERYVFLNALIESKEIDPIAIRDQLVNILLAGRDTTACLLSWTFHLLARHQ
DVQRELRSECLALPSFKESKLPTQAEIDGMKYLHVVLREVLRLYPSVPINSREAFQATTLPFGGGPDG
QSPILIRKGEAVGYCPYVLHRRTDIYGADALSFRPSRWYEPSLKKVHRFAYIPFNEGPRICPGQKFAL
LEASYTVIRLLQSYQHIEPAVDSLVDEKQTVTLVVQNANGCRVRLTPYSK*
>CYP680-fragment1|6558|Dibbae1
MFNYESNKAHRIRRAVFDPLFSRRTVLLLESVVQSHVSKLLNKVKALKTKGETISLHRAYACVSVDV
ITEYATNQCWNVMDKEDFGDEFFRMIDTLGKGIWLLKQFPFLKFMTALPPNIARLLSKEAGGVGDL
IEDCKREVRRIAATWSKEEKEKLEHKTIFHQLLDPEAETGHVVPSVNEMAAEAFAFVVAASGTTGN
ALEMTTMGILEDPKIYKRVMEELTSAFPDGEAMAWQEIEKCEYLTACINEGLRLTYGVIGRLPRKVP
DEGVVFYNYHLPKGSVISLSTIFIHNDESIYPDNKKFNPDRWINPTRPNLKKYLVPFSRASPGANYMS
HWDDSYATVT*
>CYP52K-fragment2|9168|Dibbae1
MSSAKGRAVDLQPLFFRLTLDTTTAFLFGESVLSLKASDREERFEGAFNTAQRYVAKRFRLLDLYWL
IGGKEFDKACRDIHNFADEILDRSLKPTNGNKSRDSRYVFLDAIVKDCPDRMALRHQMMNILVAG
RDTTACLLSWTFFLLVRHPRVMDKVRKEIAAGIGDAENLHRTHLKSLTYLNNVIKETLRLYPSVPV
NQKTSLKTTTFPTGGGPDRQSPVLIPRGTSCAYSVYSLHRRKDLYGEDAEDFRPERWEEELGLFRDE
VTKTWGYLPFNGGPRICLGMDFALTEAAYTIVRMIQRFPVIKLPPNEPVVKTGKEKQIITLVISIKDGC
NVVLE*
>CYP6170-fragment1|1601|Grascr1
MQAASSGEAKKRSVDPSAFRGFGGGSTLCPGRHFSTTEILAVVIMFVIRYELRPAGGKWVFPITNKS
NIASAVMEPDFDIEVEVRPRTETHEGDWAFSLADSEMVFVVVAEDQAGRDS*

>CYP6456-fragment1|5963|Grascr1
MDSLLLPITIFCTILFTYLLLDHLYIPKHLPQEPPLLRPKVPYIGHILGLLSHGSKYLQLTRHTLPLLPPS
PLPNIPPSSSQSNYLPIYTLPVLSTKIYIITSPDLISAVNRNSKTLAFNPFIAQIGRRMTGHDEATSKIVQ
HNLNGENGPGYVTDVHDRTVAALAPGPDLEAMILPMVRGVDGYLSGLKKEKERGNEVYLFAWIR
RLVTMCSTDAIYGVGNPFERDEGLGEMFW*
>CYP548-fragment3|6661|Grascr1
MPSFYHAAEGDRRIWLWQNFQTYGDRVRVAPRTVVFNSSEAYKDIYGYKANVTRAKPCGLNKLL
NAITPPNVKVYYRFIERSISKPWESLKQTKNRTKRRKPREDMFHYLCQAEHPNTGGPAYSEQELIAE
GNLLFIAGSDTASLNRCGFFFCITHNERIYQKGPLRRFVAPSHLPARSSKASS*
>CYP552-fragment2|7094|Grascr1
MSIQEFTATAMDYAARSYLITAVCIFGIYQIARCFYNLFLHPLRHIPGPKLAAATYIPEFYHDVVRYG
RYTNRIKEMHDRYVRISPDETHCCDSRFIDEIYATGNRKRDKPIHQVRGSGTVETAIFSTVNHDIHR
MRRNALAKYFSRAQIVKLEPRLHGLAERLCDKILSLGEKAPFNVTTAYSLLSSDVISGYCFGESIGLV
AQQGWEPSLREPLYALLGAMYYLRFIPPLKYIGLAMSE*

>CYP5180F-fragment2|7697|Grascr1
MKALDYESIAEMPILDSFIKESVRLNPLDKMSIRRKALKPYTFVNGGYQVALGEIACAPAWELMHN
EAKYPNANSFDGLRFVKQAENVTKSGTQDETMTGTTFTDASKDFPIWGLGSKVWYANCSTFLRER
RFAARTLLTETQSRPMACVTSHEDGDRASSGKL*
>CYP50112A-fragment1|8029|Grascr1
MTVTKQSRRMEMTKSLYDKYGRTWQSSSFGKAIINTIDPLNIQSVMAHDFDSWGLESMRYPAAAPF
MGRGVFTSDAPFWQHARAQVKPI*
>CYP5285-fragment1|8876|Grascr1
MRRFILRPMTLSDGTTLSSGCSVAAPTQAVNLDPSVWDKPHEFDGFRFEKLRESEGKNAFQFSSSRY
EIKFAGDRSERPVNQCNGVLNGPDPAVMALFKSRKGDKSE*
>CYP5285-fragment2|8877|Grascr1
MDAMGSISSLSSLNKQDQALALSFIALLLSGLAAILVWRTPSQSRKKVPVFGQGTEDLTHPLLDGYN
KHPHSVFELATSDFPTLIVPRRIIREIGSMPEKSLSLDREVYERLMGKYTRLVKSSHFTNFVKLRILKNP
KKAVKLLQEEAEWALDVSVGDVDDWKPGNVSQIVLKVVSLQLGHSFVGYPLCRDQNWMKLAIQ
YAMKTVAVANKMMSYHWLQRPFIAWFTPEIAQLRSQFKEASVLLEHFLKKRLEQCSDPGFEKPDD
LMQWLIESILEQRGDLQLHTGIQMEAVQAGTFNLSFQVCLSR*
>CYP5105E-fragment1|9549|Grascr1
MTSAINNPSKWELLPTLLFEPANIVIATEIWHELSIGLMLXXXXXXXXXXXXXXXXXXXXXXXGLSPL
AKIPGPKLAALTSWYEIYYDIILPAQYVWKIKEMHTKYGPIVRVTPWEIHISDPTFLDEIYAGASRNR
GKYEFQLRTLPVPMSMGGARTHDLHRRRREALNLFFSQKRVVNLESNIKTKGGQLAEVFETHRKA
RTIVNLYNIYYAFANEYNPLPLPKRNRDSLMAAASSCNIASDKTTISGNPVFRVTFRDPLQR*
>CYP5168-fragment3|11212|Grascr1
MIRDKRAFSPSSQGTIFPRIPIDSNSETQTLTVNGTGRYNCVGKQLAMMELRYVVALLVRRFHVAFA
PGEDGHRVEAEMKDQFAAAPGHVQLTFTRRKDKVNIGPCIP*

>CYP503-fragment1|11214|Grascr1
MLERKNAENKRSRNDMLQWMLDSARGEDADPDMLIRRMLFLNMASIDTTTLTATNVILDLLDRP
EYMESLRSELLESIEANKGINQGALNQAHKTDSFMKEA*
>CYP548-fragment2|11878|Grascr1
RWIVSSSGFDVAGGTDIGNAASVTLDRSAFCAFSSGPWGCAGKGIAYKEIGLIVARLVWLYEWRIAE
GTHVGEGNPALGETTLRNHVKELQGKDRFVLCTDGPIVQFKARK
>CYP65V-fragment2|12099|Grascr1
MLDLHNKYGEVVRIGPDELAFSNPAAWKDVYGHRTTGGDDFAKQMRFYRPVEAEKINIVNAGRE
EHSMLRRQLAHGFSDKSMHDQESLIGSYIDLLIKRLHENCAGGSKPADLNAWYNFTTFDIIGDLAF
GEPFGCLSSSDYHPWVKMIFATAQIGTFLQSAGYYPRLKSLLLRMVPNSMKEKRKHHLDLTKAKLL
RRMEAGSRPDLIEGLLKKKDEWVSNKSCTTLRT*
>CYP611B-fragment1|1600|Grascr1
MSFYKILQPSLAPGPALDAMNRIMIQNVAASLEKLSDANSRLHRIELSKWLRHKVTMATTNAVYG
PMNPFKDPEIENAFWDFEDDFTMILMNNMPSITAKKGHRARELMAAAFDSYFRQKHHEKGSILVQ
NRYKNSAENKIPINDIARYEVGGSIAILVNITPAAFWSLLYIYAHEDVLSAVREETASILTTSSSSAGKP
VRSLDITKVKTHCRLLTSVFIEVLRQKTSGISVRKVMHDTMLDGKLLKAGKTLLMPARVLHTDANV
*
>CYP596B-fragment1|1637|Grascr1
MAFTDVPSGFTLVSDIRTNHSMTAWHTIKLKPEITHLVARLSSRVFLGKDLCRNERWLEITKGYTIDS
FAGAYILRLFPAITRPFVHWFIPHLRRLRKAIKDAHNLIDPEVERRRARVQKALDAGQKPPKVSDTI
GWMHEVSRGRKVDYVAGQLSLSLAAIHTTTEVTTQALFDICEHPEVVQPLRDEIIQVISEHGWAKTS
LYKLKFMDSFLKESQRINSMSEGSMNRSVEEDLELSDGTVLPKGSRILVASKFMDPQTYPEPGKFDA
ERFLKKRQEPGQENNFQFVSTSPEHILFGHGMHACPGRFFAANEIKIALCHLLLKYDWSFPLGVTKP
KYRLFETVLSATQDSEVLLRRRQEEITL*
>CYP6702-fragment1|3803|Grascr1

MNKLMQPLITERMAAMKAGKDVPSDMTTWNIQNARPHVKFDPEWQAHAQLLVSLAAIHTTTMT
SSHTFLDLAAHPECITELRAEIEEVLKSEPEGILGKTSMPKLKKLDSFIKESQRINPLGMTTFDRKVTSD
LTLPDGTLLPKGTIITVATDEIAHDPNHFKDPYTFDPFRFSKLRTEPGNGNKYQFVTTGVESMHFGH
GKHSCPGRFFAANEIKLFLVHLIMGFDVKLPEGEGRPRNIEGMSGNRPDHSKPILLRRRDVKW*
>CYP65-fragment8|3919|Grascr1
MSRQMSRREILTNSSNLIVAGSETTASALAGVTYLLLKHPEKMAKAVSEVRSTFSIEKEITMLRINGLE
YIAACCEETLRMYPTVPIGLPREVPSPGATVCGQFVPAKTAVAMTHLATYRSATNFKDPDSFVPER
WMNDAHLAYTELKWILTRVLWNFDLKLMKEGEDWMDQSHFMTWQRGPLMIQLSSRSIQ*
>CYP51200A-fragment2|3921|Grascr1
MAATGITTGQSFTVLLHVYFSSVLASTFVYRTFFHPLKNFPGPFGAKVSKFWNVLKAYDSSNFRLMD
SLYHEYGDFVRTGPNEIAVFRPEVIRVLDGPGTKCSKAPWYDILQPRVSLATTRDKILHDQRRKTWE
QALAVYSFQDYEQRLLRFTEGLHAHIASRVMEGVDVSKMFYYYSFDVMSDLAFGESLHMLVEDKN
HFALRDTVASTLVSIFWHLIDDAQQLAKLRRELDECPDLASPTALQSLQHLNAIINEALRLHPALPS
VESCYTHAEKFIPERWYSKPDLIKDRSAFAPFSIGRYNCIGKRIALLQVRSVVARLIMHFDVAFAPDE
DGIAVWRDLKDQFNSHPGQLKLVFTARK*

>CYP6259-fragment3|5938|Grascr1
MRLFLLAAPLTNPAQFLDTSTGFLFGKSTNSLAPQGDPETNEFMAAFDRSLLGLALLLITGPIRWPLY
LDPIWKAAYTKVHTFVDSRIQAALVRKQKATDNGSTAHSGKYILLDEMASMSQDPYELRNQILNV
FFPARDTAAIGFADIMFELARHPQEWKKLRQESLKYTKAIINETLRLHPAASRIGKCALRDTVLPKG
GGIDEQSPLFVPKGRVIELDLYCVQRDPSIWGHDADEFVPDRWIGDRPLWESK*
>CYP655-fragment1|6199|Grascr1
MENCVLPSGGGESSKQPLFVEKGTEINMAFRSTHRACNIWGEDAAEFRPERWESDKWSVWQYAPF
SAGARPCPAQQVALVDCGYVLARLAQTFMRIENRDLELKFIEERMAMQSRNGVLVALI*
>CYP552-fragment1|7093|Grascr1
MPNRVKKAQAEKEAGIMQEDRTIFESVLASDLPPSEKSLRRLTDEATSLFAAGTETISWALAVITFHV
LSKPDMLAKLTAEVTQAIDGSGQMPNWAELEKLPYLTSTIYEGLRLSYGVASRTSRIPTGEDLVYRQ
DWKPKGTLRIRSAERIRDRHVLCHNASR*
>CYP5328-fragment3|7408|Grascr1
MVMNNSIFMMVAPPRLLSLPFVPKRWAQVGQATNDFRDYMMSMLNEERRLSDEGKPGTGGLMT
SLLRASESHKKSDMDGPKPLSVSEILGNVFVINFAGQDTTANTLAYAIMLLPAHPEIQALIKVQSSET
WEYDSLFPRLKRCQAVLTLNINGRMVSIHPHSYVVPSLLAFHTHPRYWTPDPLTWRPIRWITCPATE
SINFSLDQRLEREEIVVPMKGTYFPWSDGSQYCLGKKFAQVEFVALPQSPGTAIA*
>CYP5415-fragment1|7901|Grascr1
MHLRVAFKDTTLPLGGGPSCTSPIFIPAGTRFEANFVALHRRKEIFGANVNSFRPSRWSEISPKPWEFI
PFGGGPRVCVGKPKALAETAYFLCTLVRKFEKIEPRDWREWNGQVMITAKNVNGCLVGMIPAK*
>CYP6334-fragment1|8006|Grascr1
MISFLRHQPQQRHRYPSDKSQVEKLREEQRNVDLEDFQQLQSLPHLNGVINETLRLYPAVPTGGFR
ETPSEGAMIGDYKIPGNVKICAPRWSIGRLESCFEEPEKFIPER*
>CYP65-fragment3|8617|Grascr1
MKDPSSGTCVDIVQWLNSTTFDIVGDLGFGESFNCLENSCYHPWVALIFNHFKASALVASVRFYPL
VESLLMSMLPKSVMKIQKDHFQQVVDKVHRRMNLEVQRPDFMSHVIRHNDDKGMSLSEIEATFNI
VTVAGSETTATVLSGTLNYLTANPSTLEKLVDEVRTSFSKDEDIKFDKLRSLPYLNAVLNEGMRLCP
PIPAGLPRLAPEGGDTVSGIPVPGGTHVSVHARSLSRDPEAFNGPLEFVPERWLDSERCKASSPFAKD
QLQAVQVFSVGPRQCIGKNLAWAELRLVLARLVRTFDIDSVEPKGRSNPKWDDQKTWMLVEKQP
LHLRLKVH*
>CYP65-fragment7|9616|Grascr1
MLKDLKKDQNFSIERVHERIAQGVIRNDFMSPVLNGQVTLPIVNRKWLIELLGSETAAVTISCALYH
LALNPAAMQKLVEEICTTFKTADQVTMAATQDLPWLNAVIEESLRIHPPSAFNQARIVPPGGAVIC
NESLPAGTAVGVGTYAAFLSPANWIKPNSFMPERWLGEGFPGDDRRSMQAFIIDFQIDKGDSLAYA
ELRLILTLLILNFEWELCEESRDWKYGQRFYLLWERPPLMFKISKRGLW*

>CYP6174-fragment1|9742|Grascr1
MVGSELCRNDTFIRESAAYNQSMFLFGFALLKLPLGPLRERLIKPLSYFQRRRQAKVIKMVAPEVAK
RLDERNKGTTPKLPRHDFIEWTLNILDEHPPSPNHTPEPQRISHEAMLVGLALNQNPSGTVVQMLF
QILEEPQYLQSLRQEAENALQEYGWTDKLTSALPLQDSFIREIHRLYPTLTHINKVSSQRTVVGNPYT
FSDGMVLPESTRICFPASPLQTDPTHFPNPLSFHGYRFLKLAEESKNNSNDSNTLHKGVNTWAAHH
PSPTNMTFGYGNHVCPGRFLAIRLIKIIFTKVVTEYDFWWDREAGKGQPSRFEFEGTKIPCPRQRIWL
RKRGVEGEER*
>CYP6259-fragment1|9869|Grascr1
MDVYCVQHDRAIWGEDADEFNPQRWLREDRPAYETKWQYEPFLGGIRMCPAQNQVVTQLAYLL
VRWALCFERMECRDQVEGYFEREVDYDC*
>CYP5168-fragment1|11213|Grascr1
MNVRVESKNHPSDTIALTLVFIFYELALHPEVQYRLFSELCEGPLNDFNALRSLPYLTAVINEALRLH
PAVPSGGYRVTGPGGLLVAGEHIPPETTISGPRWSLARHS*
>CYP65V-fragment1|12098|Grascr1
MILIIGGSETTATLLSGATYYLLSNPAKLAKLMEEVRSTFSSEKDITINSVSRLNYMLACLNEALRMYP
PVPIGLPRIVPKGGARIAGHFVPENTTVAIHHWATYHVAENFSHPFEFHPERFLDDPEFAEDRLDTL
QPFSVGGRNCLGRNLAYTEMRLILARVIFNFDLKLAEDSKGWAEKQKIFNMWQRGPLNIYLTPASP
RGGAN*
>CYP65-fragment11|12318|Grascr1
MEAVIANITWSKTIWAVGIACFVYSLGIAFYNAFLHPLSKVPGPKLRAAFYFPEYWNIIRENTVPATK
ALHDQYGPVVRLSPEAVSFVTPQAWKESNDEDHARIRKLLSHAFSETALREQESLLKEYFDLLVHQ
LRRQVDGPESGEVDLNKFMKLITYDVVADLALGESFDALQSGQYHPWMQAFLKSFRMLRFARLG
HQYPSFGLLLKIMSKNPRVARMRNMIFGFTQAKTEKRLESPTQRKDFMSYIQGYNDERGMTKAEIL
PTAAILVNAGGGTTSDSLAALLFFVLSNPSVHSRLKKEIRDTFQSEQDINLSSVGQLEYLKAVIEETFRI
HPPAAGVFERRTDTASELIDGWVMPCNTSLGVHQWSINHSPDYWHRPDEFIPERWLSNTPKEYQK
DVKAAMQPFSTGPRNCIGKNLAYFNIRSIAARLFWNFDMELMAQSLDWDKRGTFGTVWEHPPLY
AKLSHRKI*
>CYP52J-fragment1|12675|Grascr1
MARLRHEINEVIGDSDQPTREDIRKMPFLSYVIKESLRLYPPVPLNNRTAVKTTVLPTGGGPDGNSP
MLVRKGEMVTFSPYLNSRRKNIYGADVDDFRPERWETGELAKIGWAYFPFNGGPRQCLGEDFAIM
EISYTIVRLLQAFPVIKLPKGAIVEPVGTERQRLTLVLSSADGCKVTVAQS*
>CYP5987-fragment1|6198|Grascr1
MADEFAVGPIRHDALAPLLGDGVFLTDGPDHTRSRNLLKGIFGNVYSRDAAQLKKHVQRLINQIP
CDGNTIDMQPLLQVATHESSDRANETSRFLDAFNKALQMAGLRMFLGKFAFLLGSGKSMRKSCGQ
VHRVVDKLIDEALDPRGLKSESQSKRKSFVQDLAKEVPDRVKLRYELLNVFLPARDTTFIALSDVLF
QIARHPRVWTKLKLEVLALNEPLSFQAVKSLVYLRCVMNEGEDPIPIPFKVEVADVLLRSPLDRSFW
*
>CYP52K-fragment1|6707|Grascr1
MFGDGIFTQGDEAWRHSREMLRPQFHFKQYADLEVFRQSVDDLIGDIPQKGGVIDLQPLFFLNTFG
QSIQSLKTPESAGEDTFAASFNLAQEYVVKRFRLQDFYWLIGWKRFRNACKHVYDFADQIIDRNLS
KESNDTADDKRYVFLRTLAGNIPSWDVLRGQIINILAAGRDTTACLLSWTFFSLVRYPRVMEKLRAE
ISKNVLKEILRLYPSVPVNTRTLVRRTILPAGGGPDRKSPVLIPKGTAVAYSVYTMQWRPDLFGMDA
EIFRPERRDEEDLPLNRNETYAKWGYLPFNGGPRICLGLDFALTEAAYTVSSSQE*
>CYP52P-fragment2|10180|Grascr1
MVVLCGKSLEELQLTESDDGEAFAGAFSYARHLHETVDRIVASALHQHNTSKSVQQDSDRYVSLDS
LISSTQDPTVLRDQLINILLAGRDTTASLLSWTFKCLARHQHTQSALRRECLNLPSFRTNSLPTASEIK
RMRLLNNVLSETLRLYPSVPLNSRAALKTTTLPVGGGADGRSRIIVRKGEAVGYSLHSMHRHRDIYG
EDAEEFRPERWDCEDEGDEGDGGGGGGGGGGKFAKKLGWAYLPFHGGPRACPGREFALLEAGY
AVVRMLQVFERVEEVEDEPQWDRHTLTLVLAPKNGCRVRLGR*
>CYP5042-fragment2|7370|Grascr1

MNSMVPNSNPDADAFIKAFDYAMIGLGQRVRLGRLRFLHRDPKWHASIHVVHRQVDRYIDNAFA
QIDAEKGYHYAEKIQAEPQRYILLHEMAKQTRDKEDLRSQILAVFMPGRDSVGFALSNVFHALARR
PEVYKKLRDEVTTYAPASTPITFELLKSMKYTQWVINEGHRVHPTTAQAPRVCLRDTVLPTGGGPSE
TSPIYVRKGDHLMVSMWGLHQDTSIWGPDAAEFRPERWESTRPIWTYIPFLGGPRTCPAQQMVLT
QEAYVLVRLVQAFERIESRDPHPWTEARRIGFLSKYGVKVAVTPADGGLEGKREEMV*
>CYP65U-fragment1|99289|Umbpus1
MVTQVASFTCLASRKWLKTIKQAWSVALALRFAFFILTGCLDQDPKNTDFNSIRWIRSTKSSTTYFH
PLAQFPGPRSWSASRLPFIPSLLSGTLVKDIEKLHREYGPILHIAPNEITFAKAGTWADIFSPRPGYLHF
PKDPLWWARQPKQPELLLSVPTAEGHARMRKLLTPRFAECALNTQEPVVQKYVCLLLERLRERSA
APNMAGSEEGVVLDIVPWFDYTTFDIFGDLGYGESFNCLESSRFHPWIALLFNSVKVASFVIAARYY
PLIDFLLMKCILKSTMTITDGPLSADRGQGATPPQLGG*
>CYP631-fragment1|99305|Umbpus1
MKHCTQRPATAWTFSKATAGSDFVSSVMRTVIVNLLVQPHTPEKLYDKLRSVDVSHPCYKYNELH
KLAYACVREVWACIHCLCCRLSASYQREPSRSSGVIYLPATLLLEVYAINRHKLTFGQDAEFSRPER
WLENDDEHQKKLEQSMLTVSVMELFLSPFYIHSPLTFANALISCLAPAGAPLWAGTSGL*
>CYP59V-fragment1|102032|Umbpus1
MAYHILDLGHYRLYLAIIVVAVSVLARFLVSLYKTRRAFLRLKQQGLCMPPYNPLFGHLLEVQSILSK
IPSDAHPLYLPDQLRRKYPDMGRVFYLDMWPFSTPILFAESPSAAYQLIQEHQHPKADPVRKFMYPL
TKNKDLVTMEGQSWKDWRAVFNPGFSANHLTKLVPRLLKAVSTYSDILAEHAQAKDMFYLERITI
GEGYLFYPNKSQNWYYLSKMRPNEVFLMKQSDSRKERAFAVHAAVVDPSAPESVIPHKMSHSLQG
LGQIVKLKGAENYEAWYKSTRAIARLNGVWELFTGQTVKPTPPAKPTVPVDDPKYGPKYEDRQDL
YEQKLDKYNTKYGRTLRLLKMTLEEAPKDQLGELILESSKEQMDFLKQKYEVTGYTAVYQALGKIW
KNRTAKDYSTPIEFANKIKKAKT*
>CYP50334-fragment2|100088|Umbpus1
MDDVLTLLESRAGPNHRLVRAFEIDNAFTTTDKSFYRKFMSKARRLVAKDEPTWKLMSDLMRDHV
SFESQSTTSNQSLPLVPLVQAAVLKITMYTLFKSPAEKLEAAKIRLIAERINRLWIDSKSTHEPERFQED
RRELRETVHTILSVTRVDMDRNNPLNLILPAYETLWRVVLRGFLEVTFRGAEAGTEWRQLLKTFLA
DPTLSTFKRTNDLTGISVAFIVAETLRLYPPTRRIYRDTKPKVKDDPPAHFAADIEFLHRDAKIWGED
SLSFNPSRWKDVSKKCQDAYMPFGWKPFTCPTKDDFGPRMIGLAVAALVAEFEHGWTWRAARSE
DQIDVDGPLEAERDSYVTLQLAKRE*
>CYP682-fragment4|101500|Umbpus1
MRLSRIDPEKPVIYTDKATDKQYIIAPGTPMSMTGVLIHFDENIFPNPLAFKPERWLPSDPWSNDIVE
NRKKYLVPFTRGTRQCLGMNLARDVRMDGDRGYLELFEFDYERDLKIVGDGALPLYGVE
>CYP6421-fragment1|102031|Umbpus1
MLSYSISLLAAHPEVQEWVAEEVHYVLQGQGTSETWEYSLFPRLKRCLTVMLETLRLYPSVGSIPKY
TNDRAQSLNINGEVHIIPPKTLVFLNSFAMHKMPRYWGSDSLVWRPSRWILPSCPGTVSNGASLSSG
IIFDKETLLEPKKGTFMPWSDGPRVCPGKKFAQVEFVAVLSTLLRNHRVRPVARDGEKEEDAQQRIL
DVVADSGIHFTLQMRHPTRVTVSWVSV*
>CYP5076M-fragment1|102735|Umbpus1
MFIAADVAFRVQHSVIRLFVHKRLESIGYQDRQLAQLATIGRQPVNVTKWFNLYSFDIMGDLAFAT
SFNMLESTEEHFAVKVLDKGLEPVGWMLPTWMFRLLVAIPGLANDFVRFENTMTVNTPDVIATLL
GPWKEKTPTAGSDTTSATLTHLFYELAKNPDHVLKLRDELASHLSSGSDLSHQQIKNLDHLNAVIN
ETLRLYPVAPMGIARLTPREGIEIEETYIPGNMKVSCPQYVIGRREFSENIYADAEAFIPERWSKPEMIK
DNSAFAPFSTGTYDCICKQLALLSLRTVIAKLIMDFDVSFAPGDNGKYFAKNTTQHFTWGLAELNL
VLTKR*
>CYP5180F-fragment1|103830|Umbpus1
MVARLSLAIIPSKSGWEEDDELKSITLPYNMIKSSEGSSYWTPTTLTQAIAGSWFAGSHQPWINLHFV
FLELCNRPDYADLLRREIAAHSSLDVTTITLLPLLDSFIKECLRLNPLDEMGIRRKALEPFTFSNGGPSL
AVGDIACVSAYDILHNTAKYPSPHDFDGMRFVQNSTEAPATSEGMRGTYLTDASKDWPIWGLGSR
VCPGRWHAALVLKMAVVKLLANYEFRLKDEKARHKWWWETFQLPSGGTEVLFRKRGSE*
>CYP51F-fragment1|106667|Umbpus1

MVIGMLIAGQDTSSSIGGWILLRLASRPDVQEELYQEQLRVLGPDLPLPTQQACGRFSLHRMVVRET
LRLHAPIHSIMRAVKSPLAFPTRASGTFSE*
>CYP548AJ-fragment1|107345|Umbpus1
MGEFGFATTNKAGVYVQYPELANLKLEKLLYQRGLGMRERYLQLMSNLVRSRNSKEKDAKNDLL
YFLADAKDPETSLGFTEDEIWAESRFLLIAGADTTSTALTTAFCYLSAYPACYEKLVYEIRNTFSSGSEI
RSGPKLAQCQYLRACIDVSLRMSPPISGTLWREVCAGGYVIDNEYVPAGYDVSVNPYAVHHNEDC
FPDSFTFKPERWIPSADNPEKDIERARRAFSVYSIGSRACAGRNMAYTELGDSIARTMWWGRNICNT
RGPNIFVTAGFKICEASALLSIINTAAISSKAIISIASKYKDAREQIQAFGCEVSILGKLLDQLHRRLSNP
SWTVEEYVKLLTDQIIDECINIFTQLDTFKDNLYAKPADLEPSRVSLKGTTKWVFKSTELEFLRARMD
SMKINMVLIMAMAMPQRQEQPSAESNSEKREHVKQIQMLTAQSNTCLERLESLEQRLTVDDNFGS
DDTNSVATFQTTRTSQTIRDNLLQLSRAMPCALELGLTDNLPPKTAIGIDDSLNTSDLVERFLQQQE
EAASACAENDLVKRFLHQQDEATTTCSENDLVERLLDQQDEAATACSENEQACSRPEREVDLSTE
DDKTSSSVGPYRLKADSIAREDLQKGNGSKLRFIFCNPEAGILEAKSSIWYHKKLATRMADAGDQQ
YGIFPIPPRTLIDFQLYRRGAPVENGRVQLYRYYSVDLSKLGYAIAWRRYVGQIKHLVVEDERPGDLY
PVKSIFIATFS*

Partial P450s

>CYP50334C1-partial|121516|Acastr1
MHLLLALLTPVIFCIAVWLIQTLEYITIWKHANGSSKASARPKIFPTTQDISNSARKPLKSRAIPNQRL
VRAFGIDNAFTTTDGDYYRAFRRRVELLLKINDEQWTHLAHTATDLARSGLQACTPEYDGTEILLV
PFVQKMVLKFSMHVLFSVPVDELDDDVVATIAEKINSLWISSKSAQPTKSMRSDQKELRAALQRIFP
RVGKTARENPLNLILPAYETMWRVVLRCFLEVAFRPHNHQNTPVFRQILARFLADPSRATFGSTTPE
TGGLSAAFIVDEALRLYPPTRRIYRQQSIAGRRPALVAADIEHLHRDPRIWGEQSSSFHPSRWIDVSD
ECRRAFLPFGSKPFTCPAKGDTGPRMIGVLVAALLAVFGDDWVCGAEHPEDVIPCWEALSAGRDC
YGTLVLNGPFT*
>CYP5334A2-partial|125949|Acastr1
MELLQGFPTVMQNVLSTFKSGTLSQGRRISGPKWQFPNGQILDKFVNGRAKSDEWREKYGDVYRV
WGGPSPEIVITTPEDLRTFSLDGEKHGKHSNLGWFLDQLLGRCIGFLEGDEWKQKRQIFYPAFSHDG
TATRIDLTESAARKFIEDLPSMHSDGLGTNGGGKTISFHIVNGFRKFPLYVTASVIYGAMTNAEKDD
LWALAEKRLALWPYAVIGGPYRFSWGSWFDRTTYNLLNEYTTEWRNYNDRMVQTRRARGIKTPL
VLFYDAYEAEDVTLENLMHSLDEIILTNLDVMVHAITWVITLIADNKQVQQELHDEVDASWDRLH
DYLSKSDTHLHRSFMETIRLRPPASEDILIYILFNIGESSPSVKNFNGILVKPSTMVLVDVLGINVRNPF
WGSDS
>CYP51183A2-partial|6920|Dibbae1
MGDLIWLMLLIGVLVYLFPGPKLAALTDYWELYQDFFREESGYLFIELDNLHEEYGILPWNTSIDFR
MHFYNVHAGPVVRIRPNEVHVKDSNWMDVLYTGPSDPRDRDPLVAIGTGPHELHRIRKAAINPVF
STRSIVEMEPDVQAHAARLFEDLEKRVGEVLDMRIYFFAWTTDFITNTIFRNSIRIFWEPERATRWFM
IVWDFSGKFPLMKHMPWLVTTGLGLPLTVWKVLFPSLVPYISIYKDLLVMATKAFSDYSEEANRKLE
MPQEATTKDIFETILSSSLPFKEKQPKRMANEVFNLLIAGSLTTSKTAVVAMYHILDNPEVYKCLKAE
LLSEIRDKHALPNVKTLQKLPMLVMRCYPFERPIF*

>CYP5105E3-partial|1062|Dibbae1
MGGTLNFDLHRKRREALNPFFSKKGVLELEFMIGQKVQQVCQLLESHIKSRTPVNLSDVYYAYAND
FGHDNNLLEDEATSSTQRNNLSRLLLGVKVNQHFPWLFDLLDVIPFPIAKHIMPPGAIDMTHFVEM
CIILKITQNIRREVGQVLQDKDNKKTDRRSIFYELRDNPNLPPSEKSLSRLEQEGTLLVMAGTESTAKS
IAIAHFHLLNNPTCMVRLRAELRTLPDSATWSQLEQLPYLSGVIAEGNRLSFGVTARACRIAPDEAL
QYGKYTIPAGTPVSMTTLSVHTDESIFPDPWKFDPERWIGPEGTERRKYQMAFNKGGRICIGTNLAN
AEMFLVLAAVAQYDMELFRTDLSDVQFKHDFHIAYPELDSKGARVMVRGKAQMA*
>CYP6712B1-partial|5873|Dibbae1
MASVRRLQPVIQERIDVLLRRMKGYKDSGEVLNASCMFAALTNADIVQPDVVMTYSFARCDYRLE
APDFDPSSRDASLAGATSGAFLKHAPWFNDLMQALPESVASMLNPAIATFVAQKRNSRAQVLEIV

KGQNEKWKDREHPTIFHSVLGSNLPDEEKSMARLSDDAQMLVMAGTLTTSATLELIMFWLLSQPA
TLQKLKEELTAAIPNLEAVGSIPLPTLEGLPYLTAVIKEGLRLSYGVSCRLARIDPDNAIIFTDKDTGK
KWVIPAGTPVGSTSVLIHHDENIFPDSKKFSPERWLDGKGRELERYLVSFCVGSRKCLGVNLAYAEL
YLALSAVWRQWGSGGVDGIRGNDDMGVMSLWETGLRDVEIESDAFVPQPQKGSKGIRVLVQG*
>CYP676D1-partial|9763|Dibbae1
MDKGPWYDIMAIPDSRFPNQMSERNAKRHIQKQKNLAAGYSLSNIIKSEPYIDALLHLFRCRLSALA
STHSPVDWDYWLDYLAFDVVGEVTFSKQFGFLAEGRDIASAIANQTVLTIYLAVMAYFPWAHNYL
LANPLIEKLGLQPAMHILDTCTAAIKHRSSNDNVRKDMIEQWLDQHRAHPDRMPASEIFAAVVIT
VGAGADTVSAALQALLYYLLRTPDALALLRDELDAANLSDIPSFAETSTLPVLQACIKETLRIHAPIA
VSLPRVAPPGGITICDRYFPAGTILSVHPYALHNLPAIYGPDFATFRPQRWLPKDALKDASETLKAM
DACLIPFGTGYNGCPGQHLARIELCKTAALIVRDFEWQQVVPGRPFRCAYHFAAVPHGWPCFVRR
RQGGGRAVDDGEGMNGNAVMGNGSGGMGKS*
>CYP570H5-partial|10012|Dibbae1
MSLLPKVALFFDAAVQRSFQSPTISCMILLFCVAFYRLSTRRFLHLRKIPGPWWAPYTRLWLFKTLAS
EDSPNRYIQVNEQYGPLARIGPNHLLTNDPSVFRKILAARSNYLRGPWFDSLRLDPNKANLITEKDL
LAHNTLRQQMATSYDGKDIEGFEEGINESLQEWIQFVGEHGVSYPGSPRGEFEIGRSIQYLITDMICR
LCFGRPFGFVAKHADCYDFLKTLEERLPIVEKFSIYTEVSTLLSIISYIPLLNRVLPSVRDRNGIGKIIGIS
REVIDERFANGIHTKNDMLGSFLKHGLDRRQAELEITISLFAGSDTTATSIRAILLHIVTNPLIYNRLRL
EIDTHIANGTLSSPAREEQVRNLPYLQACIREGLRIFPPITYLRERVTPPAGDTLSGFDIPGGVNIGFNL
PGLLLNRVFNPDPTVFRPERWLDGVEPGHLREMERVMELVFGWGSTRCLGIRMANTNQSKFFVEV
SYLTRAGGSRGVDT*
>CYP51159A1-partial|10280|Dibbae1
MLAGPEVSRNEDWIMLQLDFAKEFVAAASQLLQYPIPLRPLITPFLASVRQTWKYQATMVSFLEEY
KLKTEAAVKGGAPPPNTLAQWLAEEHNDGDKQASVQHQANLMLITGLDCIYTVSQLCTQTLAPP
VTETRKDLDLTHILTAAFQRKALRPVTLSDGTTLPTGTLFFVPNCAYSTDPSAWESPSTFKGFRHISSS
TDNTTSSQAQSQSKEATDSQQHGQHFGVPHLRELTFGIGRHACPGRFYAVAEIKMVLAMLIRDYD
VKLVEGKTEAPKATADGWLVFVDESEELLFRNVVPN*
>CYP51156A1-partial|10704|Dibbae1
MVENEDKPASFGWISRLSEISVLVWVPLLLCFWIFIQLSLIVYSVYFGPLSDIPGPKFAALTDLYESYSA
GYRPDFYWRDVQELHKRYGPIVRISPNEIHLDDVGFSEGIYPTIKDLDKDRKSGGLLLRRDVQGSAA
RRLHRRVGNNVSSQNTRNKAASHDTETLWQMMWEFSRRKEAVLSTVVSKALARNIGAITDETVT
VIENVLHHVFHNHTVLHKIETELESVELADHALLDDEKLDQLPYLSAVISESLRLSIPMCVTEITTVL
DTDMIYKEIIIPTGVTISMTLADVHFDQQYFPAPRTFHPERWIGPDVILSREFDPKHCLGMLDLEFAR
PGISVLRLLLKDIILGLCIRSFFLLSEQ*

>CYP6845B1-partial|12083|Dibbae1
MSLEMFGHSWANFIGFAILFVAFLKFYCITLVAYPVVSDDITHARIRKLLAHAFSEAALQEQAPLLK
TRVKELIDQLDLHLDSIAKVNVDLNMWYTLLAFDVVTDLSFGEPLHAVKRAQRHPFIEDFFASCRM
YPMIPLSHAYVSMGLLMKLMLKIPAFKKVQDKGYMATKERVEKRIASHSPDRKDFMTYILRHNDE
RGMTKKEIIGSTAVLVNGGGESVAVCMTAATFYMLKNPAILTKAQMEVRNAFSEQDQITLRTAAR
LVYLNAIIEEALRIHPPTPGNFSRRTGPSGDMIDGHFVPPNTSVGVHQWSANHSPINFLKPDEFVPER
WLAKPPEEYRSDSRAASQPWSIGPRNCLGKNVAYAEIRSTLAYMLWNFDMELCSESKNWATNQRF
DITWDRPPMYVKLSRRQTA*
>CYP5589B2-partial|12084|Dibbae1
MYASDRMVDIVKKGINPQLPQLTPILMNEINYAFQKELGDFSGWKEITAMSFFGSVAHRTASRILIG
EELCRDEKFIRLSMDFVMSIFVTALVIVKLPLGPFRGLLAWPLSLLHRWKLNRCVTILLPIVKARMGA
RAATAEKDIAPPLDAIEWTLAFSKPDSPNNTPEYVTKELLHNLWAGSSAPGGLMTEMVYQLLLEPQ
YLEPLREEASNALKAHGWSEKTLDSLYLQDSFIREVNRLYPTGSVTCSRTILDKPFRFSDGLTLPIGSR
FGFPIKALQNDPDNFPNPDTFDGFRFARSSTSENHIDQNSRRWGAASMGTTNLAWGYGNHVCPG
RFYAVREVKMVFTKLILEYEIKWASPVAVRPPPVHVEGQFIPNMGQKIALRRRVE*
>CYP6267B1-partial|9134|Dibbae1

MHSGPIQLNKSIGTVSHSLHKTRRTALNPFFSKRSVVELVPFIQKLNDKLCGRFADAAKNREQVNL
KYAYAALTMDVMSEYCFSRDLKTVEKSDFGRKSFDDVDFFLEMSLIDWFSRLTSPAMAGILDLRDG
LAHQVEAIRAGKDRSYENSGHRTIFHDLLESKLPPEEMTRDRLRDEAFSLMTAGSGTSAFVLKSLSY
HISANPTIQETLHREICTVMPHPTSHADLSDLEKLPYLTACIQEGLRITHPVSHRISRAFPEKDLHYKQ
YTIPRGTIVHMTAMLIHENEDIFPEPKTFRPERWLSPSPPTKYLVSFSRGTRACVGINLSYAEMYLILA
SVYRRFEFDVSRVSRERDVEVHRDVIMAVARADSEGIVVGVKEITD*
>CYP6456C1-partial|5964|Grascr1
MSSXXXXXXXXXXXXXXSLLAPRGSHARSTLAAAFEKYFEAYEPTTSSSSAMIRARHHSATTYGITPL
NQGKLEVGTLLGILANTIPSSFYMLVHIYSDEDLLADVRAELERKCVEIEKYADEDAEKKEGENKRG
DGREKGIGSDSGPEEGSGSISESTRGKADGRDSPTRITSMRANKKRLLNMLSLRDKTPLLYSVWQELL
RVHAQGTSSRFVREDTLLDNGRYLLRKNNIVQMPMAVMHSDPKIWGDDAGVFRARRFLRGEGGA
GAGGGRGKEARKGGGGGGGGGGGKVGGREGGKGRAGGSGGGAAAGKIGSGKISAAYRPFGGG
ASMCPGRFFVAWEVMALVAYLVLRFDMVPLKDHLKGGEREEGKWEVPRQKQESLATNVFPPERD
FRVRVLERKGWEGVEWGFKVR*
>CYP5238C3-partial|1146|Grascr1
MGKVKIVPEYMCMDMGMISIVPEYMFLTPGVAAEIDFGFARSWGHDLEWKEVRVWTSALRIVAG
AANGAFCGPPLLYSLALGRDVAFLDKMKDHAMVIFAGALVLNSFPDSLHPLFGPVLSWASGFMG
KRAIENSLPIVEARLARTARWKAEPDCGWTPPNDALQWIIDECYASKVPAAQQEPKRNFILNLFST
DPTLGYVDVLRHECEEALSGSQGAWTYAAVKEMRLVDPAIRGSMRMNPFGTLSLPREVVHPAGIK
VPGFDSFIPNGTRVALPAEAIQYDGSIYEDAHRYNPFRFARTWDPESNQMKSTVTLDDSFLAFGVVG
RWACPGHFFALLELKIFVAEMLMKYDVAYIKTKAKTALFNVG*
>CYP5295A6- partial|9684|Grascr1
MPAPIGDDVLSRGMRMFTLPYGPKWRTYRNIVHQLVSSTMTATFIPAQEFETKQLIYDLSAGDDNQ
LAFIQHIRRFSFSIIMTSTYGNRVNSWDHPDVEFSGRSSRILGRLTRAGAFIVDELPFLANLPHWLQPG
RKQAEKIAVELLDIKMKFWQRLRDQVDAGKAPVCYAREILENSASWRKQGLTDEDAAWIAGGIVE
VGSHTSSTTLLHLIKHLAAYPEAQQAAHDELMRVVGPDRTPTYEDIKNLPYIRSCLKEVLRLCPTPIL
GIKHYSDADVQYKDYAIPKGTVLLANTSFMHYDPDRYPDAHAFKPDRYLAHTKYSADYAAGGDP
YERDHFSFGGGRRVCPGTRLAENTINISLANLLWAYEIRPPLVHGVEAKGMARRSCMVWKLRGWI
SVMTRTRRLVFVGRSLMLFALWRGARSGWGLLGGSGRRLVGRDTC*
>CYP51190A2-partial|9772|Grascr1
MVSISSPKDLSTVYDYSRKLKKSDLYGVFRPYSRGKVLTGMVALQDESYLQMLRQPFASIYSYHSLTL
VESHVTSTVRAFVARLQNFARRGKQFDFGFWLQLFVFDALCEATFSKRLGFLDEGKDVGDMLALV
WKQFHDGAPSNAMDGQYLEKESAHTSALAEFPDDTNSTICIGALITWTLGGIGAGGDTSVNVIRAL
FHNLLTHPLTMDLLRKDIHLGIERKGRDLTWQEARNLPYLSACLLEAARIHPSIGLHLERIVPPGGV
TVSEVYLPPGTIVGANAWVVNRNIEVFGTDADIWSPSRGVHRDG*
>CYP51162A1-partial|10339|Grascr1
MAAQFKEEFEHNRVSNFALHARRFMLGSGLSLLFGDNYFVQEGRYDPDTLLAAARGAAIPFCLTA
VFPYICVPLMRIHILKRLLIPHEEDARGFGVMMAHFDRSIKAHNKQSGECILTNILSAHNPTKELPVT
PADIKAESLLLMSASPDTTSPLASALLASLIQNPSSKTKVQSELNQALKSNTIQPNIPIPIPIPWTSLSHF
PYFTACLYETARLFPPVVSILPFSAPDSGLHLPDGRFVPGGTAIGAAAGVINRNKTVFGVDADMWR
PERWLGGREEVAEMHRYLFTWGFGARRCTGQKITVMLCGKLVLQMLRELEIEPAVGDLDAVRKE
GMGFALYDGFYVKCRPR*
>CYP5168M1-partial|10359|Grascr1
MLYAKRLESQVTAHTGRVIDASRLAYEFSFDLMGDFAFGRSIEDSEQQEWRQAIVALKQGMAMLG
PWSPVPWLVRLGFALFPFVDPIRSFNRELDWCKDRMKERLQRTVDKPDISHYLIEGALKHGASLDD
HHWQGDAIAMIVAGSDTVAPTIIMSLYLLATNPIEQIKLRQELRTVENPYEPELLRSLNHLNGCIEET
LRLYPPVPTGGYRKTGPEGLTIGQTHIPAHTTIVAPRYNLGRLRSCYKRPYDFVPERWYSSPEMIRDR
RGHVPFNRGRYSCVGKELALTEMRLVLALLVSRFKFKLADHSDGSNVLVEMKDYFTIQPGKLDLIF
EKI*
>CYP51169A1-partial|12959|Grascr1

MLKIVGQLTSRIFVGEELCRNDEWLKFLVEYAELAIIGTTALQKYPSWTHPFMAPFVPEIQDIHRAV
KAGKGMLWPVLRERQDRMTNEPDWVPPEDFLTWVAKSSKDGWDPSKHIQAQMGIGLASLHTTT
TVLTNTLFDLCVNPSLMSMLREEYTSVQNSGQDMKHMFTGMRKMDSVIRESMRMHPPLVLSIQRH
VSTDIRLSDGNVIPKNSFIGVPTYVMNFDPQVYDKPEVYDGLRFEKIRDEANQAGNAQKDTRAQLT
STSPEFLGFGHGKHACPGRFFAANEIKMIIGHLLTEYDFKLEHPEAGRPKSSTTKANISPDHKARLLF
RKRRETSF*
>CYP548AJ5-partial|13026|Grascr1
MVPSPGAYNIFTVVDKSMHRHKRKVLSQGFSDRCIRAFEPTILKHVDIFVEKLFARPSPLSPAWGLPF
ASKDDEGWSAPVDMTLACRHLGYDIMGAFGFGQSFDLQGSDKNRFLIEAVTATSRKAGVYVLYPA
LQKLQLEKLFYKKGLVMREKYLSLMAGLVKNRKQQEVKGAETRDLLGFLKDAKDAETGVGFSESE
LWAESRFLLIAGADTTSTALSAIFFYLTLPHSPFTPCPSPLARLTYEIRSTFPTHASINSSHLLSSCPYLR
ACIDESLRMSPPISGTLWRTLTPSPPDSSSITSQAHSPSLPHFCHPQNNSTHITIDNQPLPPNIDIGVSPY
ALHHNESLFPDHGSFRPERWLSDLAHTPEQKQQLARARQALI*
>CYP51183B1-partial|2704|Grascr1
MRKAAINPFFNAASVQRMEPDIYQHAEKLFENLENSIEAVIDIRIYFFAWTTDFIINTIFRDNTEIFWD
PIQASAWFQIVWNFSGKFPLMKHTPWLQLFGLARKAYADNEAEDGGDPKTEAEGSKDIFQTILTSN
LPEIEKLPKRMAAEVFNLLIAGSLTTSKTSAIAVFHILNNPEVLQSLQEELKQAIPDRQAIPSYRELQK
LPMLSAIIKETMRIADIVTTRFPMIAKDKVLEYKGAIIPAGVGASRKARSKMALTPGMYSLPSA*
>CYP51185A1-partial|3341|Grascr1
MPKPSATPRRRLMSPLLTKSTIQSSTSLNHLLSRILGARLLPLLRSSSTQSQPLNLLRQFRNLAMDLTS
TWTFGLNNGTDFLTDTAASDRYFRLFDESAYNAFWRTEFPWLTKVVGVTGLFAPPKVAEMARRET
VGWSRGLCERAGGERAGEKEKNLEGGNGGGEKSEKGGREEKETLYLRMRDGLKGIYAGSGKSEEY
VKQELAVELLDQLGAGHEPMGTLLSWVVMELSQRKEWQERLRREIRENPEVLSFLKVGEEGESLSE
EGKATQAARALDALPVLDAVLQETLRVYSVNPGPQPRLVPKGGYRLSMPSKLAEFGRVGYEGSVFT
GGHGSHGIELLLA*
>CYP5042F1-partial|4578|Grascr1
MTTLRMGESTWVLLNSDRVVHEIISKRASITHERPHLPVASGLMSQYQGTILRQSKGWADGRRLMH
RMLTGTAMKDYARIQESESVRLLTNYIQRPGAWYSHHYEFPYSIIHRIVAGDNVPRLDKQREEFARI
TKEFIRGINASLLDFFPTLTLLPEALQPGRRNWEKIGQNHQRVMESWWTSVKDNLTSNPSAHSFITD
ILLKDAKFANDDREAMYLATAIIAAGSDNIRMTLNVMVMAALSYPAVSQKARAEIDNICGSSDNL
HLPSLEDLPHLPYISAMIKECLRWRPVLPLIPQHRLTQDLNFEGHFFPAGTDFVVFPAGTDFVVQFT
GGEFRIRGSWGFQAGTMAGWPSA*
>CYP6259C1-partial|9868|Grascr1
MTNMQALVYAAFAALAIYLIWQYWSFQVRRNRLVRLKGCEPATRYPHKEPFLGLDFFYNLQVAD
AAGHRSPAYLDLHKKYGSTFEVKAFSKTQVETAHAENIQAICTTQFEDFGVEPFRGNVAAPFMDR
GVFMNDGEYWKQSRALIRPIFNKAGIADLDYFELHVARFLKLMATDGETFDVLPFAKRLFVDSASE
FIFGRSTNTLVPNPPKELIDFMAAFDRALLGTALTFMLGPLRWFVYIDPYWIQAYKTVHRFVDSRIQ
QILQERKACLSSNPENKPSAIAIDEKKTKAERYVLLNQLVEQTQDPRELRNQIINIFSPTRDTTALAFG
NILFYLSRNPSWYNKVRSEAVPLRHTHLTYDVVRSLKTIKAIVTEANRLHPAVSRLGRAALKDTTLP
RGGGAPP*
>CYP51186A2-partial|4571|Grascr1
MITPLFKKAKVRSAAIFKRHVDRFISQIPRDGSTIDVQPLLKKTNFDSTAKFIFATANFIEAFNYANDG
SLKRRRTGRLAFRYCFDSEYTRTISEVHAFVDKEVARLLNSPPPPPSTAEKTTKPRRYVLLTELASQIH
DPLALRSECLNLFAGGRDGVAAPLRTVALTIPEGADPDLDSKILRLFRNVIFETIRSTGPSATVTRTAF
KDTLLPRDGGRDGDIPCFVRKGDQVSVIGWGRNHIESVWGRDCYEFKPERWEGGERSERMLGGRI
PAEFVPFGGGRRMCPGYGQVYL*
>CYP584AW1-partial|10870|Grascr1
IRWSKQLLKPISFLTTDPQHIQAIYSTDFHSYGVGPLRHFPFEPLIGSGTMNTDGAAWEAPRALLRPIL
TRSQVNDLAMLERHVQNLIRKIPKDSSTVDLQPLFACMDLDYSTEFIFGESVASLTSESTLDAKTFLE
AYNTAQVGIAYRASIPYWNIFHRDKVFWYCCRIVHDYVNRAVAQALLNHKSGTRRPNLDITKHIL
VSSDPLQLRNQLMNVFLPGHDSTAILISNVFFALARHPATCSRLRDEVLSLDPSAAITADALKRLSYL

QAVINETLRLYPPVTAMSHAALRNVQLPTGSGPANAAPVLLPRGSVVQISYKALHRRRDLWGADA
DVFKPERRLADAGMDEKKPVPASWTFIPFSGGPRTCPGQQRALAECAYVVARLLRTFRAVENRDP
VWEYHDLYKLVTESKNGVKVALEWA*

P450s from the Literature

Grayanic Acid

>CYP682BG1(ADM79460.1)Cladonia grayi

MLGVIQYSILTIFWLPIAAAXLYGAGLAIYRLFLSPLAKFPGPKLAALTRKYESYYEAYQNYEYYWKIKE

LHKQYGELFTPLTASFXRXGPIVRVNPHELHIDDKDFYYKLNSFQGAWNKDPYTAHQFANPGSIVGTIDH

DIHRKRRAAIMPFFSKQKIYALESVIQGMVDKLCYRIEEYGKTGQPVNLRNASKCFAADVVGEYCFAESG

GLXDKPDFAIEEMNQQQQGLKAGLRARYLPSWWMPVVRGAPAWIRASIDPAAKHFEVWHRVSDSLFVRLY

DARKXGPVGRMEKRKNDEFYEKAGHRTIFHELINSPHLPPEEKGTGRIIQEAGAMVGAGGESTSQVITAF

VYCLLANPQVLSRLREELRSVIPNADSPAPTLRQLEALPYLVGPLLXSTYKYVGYLLTLVARLRTGKIAR

HQRVPRDRPLYFNEWEIPAGVSXHEDNIPMASTBTAQTICSMTPIFLQIDPEVYPNPHAFMPERWLNLDE

XQRQRLEHNLVPYSKGTRGCAGLTLANAELYMLIPALVTRFDLELFDSDAWDTEMAVDSHHHSPRPDSKG

VKVFVKKSTF

Atranorin

> CYP65FQ2(A0A8F4SN83.1) Stereocaulon alpinum
MALLDTIELFSNFSLSGVFAGLVLASLLLTTTYCIWNIFYNIYLHPLKGYPGPKFLTTSRLPYLKWMFSGT
LVPNFQRLHEQYGPVVRVAPNELSYINPEALKTIYGHRQPGEGFRKNPAFFQPATNGVHSILTSEGDAHS
SVRRKILPAFSDKALAEQQDILQHFTDLLIRKLRERVEASKSSEPVDMFEWYIWTTFDLIGDLAFGEPFN
CLEAASFTEWVALVFNAFKTFAFINISKQLAPLDKLVRLMIPKSMKARQDKVFSLNVAKVDRRIASKADR
PDFLSYIIKGKDGVAMALPELYANSTLLVLAGSESTASGLAGITFELLKHREAQKKAVEEIRSAFKTEDE
IVPESVKRLPYLAAMVSEGLRMYPPFPEGLPRLTPRQGAQICGQWVPGGTYVQFSTHAAHRASANFTDPN
VFAPERWLGDTKFASDIKEASQPFSIGPRSCIGRNLAYLEMRLILARMLWSFDMQLTPECEDWDDQNSWI
QWDKKPLMVKLSLVKR

usnic acid

>CYP6309A2(A0A0R8YXT5.1) Cladonia uncialis
MISPVSSILASIWDNSKLLLDHTSVLSIALIGVACAISIRSILYVRLACANYSILLLTHAQRLRLAYSTP
LRHVPGPWYAKFTALGLRANDVAGNRWYYVQGLHKKYGSIVRIAPEEVAISDPKVVSKVHALGTEFRKRQ
QPGTPFNIFSISDPKAHRTRQRFYAKAFSDETLKASTEPAVRQLIKTAVASIKRDAALRKDHTADVYKWC
MLFGSDVAFQVIYGNSNTEGLMATQKTTDEVIMGAYLQRMNAWAQFCFPVFLLGRWLSPLSPTLHNIFRV
EEKYGDFWQEGQRQREIAARTVFVQNTKYSKNDGVFSVSDEVKLSDVDIAHDITTFLGAGGEPVGASLVF
LIWQVLRMPDLQRELEAEVAGLTEPITDATTAQLPILNGVIYETLRLYGGGVTQMPRYAPIATELGGYVI
PPGTAVTTHTGALHRNPAAWDDPEK
> CYP6309A3(WBO01282.1)Cladonia rangiferina isolate MI-R-4
MVSAVASSLASIWDSGKVYLERTSLLSIASIGVICALFIRSTIYVRLFCAGRSILLLIHRQRLRLAYWTT
LRHVPGPWYAKFTGLVLKAHDVAGNRWYYVQALHKKYGCTVRIAPEEVAISDPKVVRKVHAFGTEFRKRQ
QPGTAFNIFSMSDPKLHRTRQRFYANVFSYETLKSCTEPDLRKLVEMAVAAVRRDATESKDHTADMFKWC
MLFGNDAAFQVIYGNINGLIANNGTTDEVIMGKYLQLMTSWANFCLPIFLLGRWLSPFSQYLRDTFCLHP
IYVDLWEEGQRQRDIAGRTVFVQNTKYTKDDGYFRVSDEVKLSDVEIAHDITTFLGAGGEAVGATLVFLI
WQVLQMPDLQRELEAEVAGLTEPLTDATAAQLPILNAVIYEALRLYGGGATLLPRYAPVATDLGGYIIPP
GTAVTTHTGALHRNPAAWDDPET

Physodic acid

>CYP682BG2_Pseudevernia_furfuracea

MLGLLKIGIFTILWLPLVAVGFYALALAVYRLFLSPLAKFPGPKLAALTRKYEFYYEAIENYEYLWKIKQMHEKY

GPIIRISPHELHVGDVEFFDRLNSFQGKWNKDPYTAHQFANPGSIVGTLDHDIHRKRRAILPFFSKQKIYALEP

VITSMVDKFCDRVEEYRKSGEVMPLRNGFHCFAADVIADYCFAESGGLLDKPGFALSNMQQHKHGLKSGLRARYF

PSWYMPVVRGAPEWITQSVDPAAKHFEVWHREVDGPVRRMEERKNDDFFKKAGHRTIFHEVINSPHLPPEEKQTI

RVIQEAGAMVGAGGESTTQALITMAYCLIASPEKLARLRDELRTIMPNAKSPLPSLRQLEQLPYLTGCVKEGLRR

```
LRTGKIARHQRLPRDRPLYYKEWEIPAGTIISMTPIFHQVDPDVFPSPHAFLPERWIDIEEEQRFRMEHHFMPYS
KGSRQCAGLSLANAELYMCIPALMTRFDFDLFETDEWDVDMAVDSHHHSPRADTQGVRVFAKASTF*
```

The above sequence is retrieved from genome accession number: JAIUPS010000069.1
Conting: Pseudevernia furfuracea strain Pfur_phy_TBG_2152 contig_31_arrow_pilon
Region: 29836-31974 (+) strand
